# Risk factors for the spread of vaccine-derived type 2 polioviruses after global withdrawal of trivalent oral poliovirus vaccine and the effects of outbreak responses with monovalent vaccine: a retrospective analysis of surveillance data for 51 countries in Africa

**DOI:** 10.1016/S1473-3099(21)00453-9

**Published:** 2022-02

**Authors:** Laura V Cooper, Ananda S Bandyopadhyay, Nicksy Gumede, Ondrej Mach, Pascal Mkanda, Modjirom Ndoutabé, Samuel O Okiror, Alejandro Ramirez-Gonzalez, Kebba Touray, Sarah Wanyoike, Nicholas C Grassly, Isobel M Blake

**Affiliations:** a Medical Research Council Centre for Global Infectious Disease Analysis, Department of Infectious Disease Epidemiology, Imperial College London, London, UK; b Bill & Melinda Gates Foundation, Seattle, WA, USA; c Regional Office for Africa, World Health Organization, Brazzaville, Republic of Congo; d Polio Eradication Department, World Health Organization, Geneva, Switzerland; e Expanded Programme on Immunization, Vaccines, and Biologicals Department, World Health Organization, Geneva, Switzerland

## Abstract

**Background:**

Expanding outbreaks of circulating vaccine-derived type 2 poliovirus (cVDPV2) across Africa after the global withdrawal of trivalent oral poliovirus vaccine (OPV) in 2016 are delaying global polio eradication. We aimed to assess the effect of outbreak response campaigns with monovalent type 2 OPV (mOPV2) and the addition of inactivated poliovirus vaccine (IPV) to routine immunisation.

**Methods:**

We used vaccination history data from children under 5 years old with non-polio acute flaccid paralysis from a routine surveillance database (the Polio Information System) and setting-specific OPV immunogenicity data from the literature to estimate OPV-induced and IPV-induced population immunity against type 2 poliomyelitis between Jan 1, 2015, and June 30, 2020, for 51 countries in Africa. We investigated risk factors for reported cVDPV2 poliomyelitis including population immunity, outbreak response activities, and correlates of poliovirus transmission using logistic regression. We used the model to estimate cVDPV2 risk for each 6-month period between Jan 1, 2016, and June 30, 2020, with different numbers of mOPV2 campaigns and compared the timing and location of actual mOPV2 campaigns and the number of mOPV2 campaigns required to reduce cVDPV2 risk to low levels.

**Findings:**

Type 2 OPV immunity among children under 5 years declined from a median of 87% (IQR 81–93) in January–June, 2016 to 14% (9–37) in January–June, 2020. Type 2 immunity from IPV among children under 5 years increased from 3% (<1–6%) in January–June, 2016 to 35% (24–47) in January–June, 2020. The probability of cVDPV2 poliomyelitis among children under 5 years was negatively correlated with OPV-induced and IPV-induced immunity and mOPV2 campaigns (adjusted odds ratio: OPV 0·68 [95% CrI 0·60−0·76], IPV 0·82 [0·68−0·99] per 10% absolute increase in estimated population immunity, mOPV2 0·30 [0·20−0·44] per campaign). Vaccination campaigns in response to cVDPV2 outbreaks have been smaller and slower than our model shows would be necessary to reduce risk to low levels, covering only 11% of children under 5 years who are predicted to be at risk within 6 months and only 56% within 12 months.

**Interpretation:**

Our findings suggest that as mucosal immunity declines, larger or faster responses with vaccination campaigns using type 2-containing OPV will be required to stop cVDPV2 transmission. IPV-induced immunity also has an important role in reducing the burden of cVDPV2 poliomyelitis in Africa.

**Funding:**

Bill & Melinda Gates Foundation, Medical Research Council Centre for Global Infectious Disease Analysis, and WHO.

**Translation:**

For the French translation of the abstract see Supplementary Materials section.

## Introduction

Rapid growth in circulating vaccine-derived type 2 poliovirus (cVDPV2) is threatening successful cessation of oral poliovirus vaccine (OPV) use and delaying global polio eradication. cVDPV2 has caused more cases of poliomyelitis than wild poliovirus every year since 2017, with 1057 cases reported in 2020.^1^ As a result of expanding cVDPV2 transmission, the overall number of cases of poliomyelitis has almost doubled each year since 2016 and is now at its highest level since 2009.^1^

By contrast with the parenteral inactivated poliovirus vaccine, OPV is inexpensive, easily administered by non-specialists, and replicates in the human intestine to induce mucosal immunity that prevents further infection and transmission. However, OPV is a genetically unstable live-attenuated vaccine containing Sabin poliovirus strains that can evolve during replication to regain characteristics of its parental wild type, including neurovirulence and transmissibility. Persistently low population immunity allows the vaccine virus to replicate and be transmitted from person to person, leading to outbreaks of cVDPV. To completely eradicate poliovirus, the world must stop using OPV. In April 2016, type 2 OPV was withdrawn from routine use, and a bivalent OPV containing types 1 and 3 replaced trivalent OPV in routine immunisation.^2^

**Research in context**

**Evidence before this study**

Vaccination is the key tool to stop poliovirus spread. Several papers have modelled poliovirus immunity trends, but few have used empirical data on vaccination history or immunisation coverage to estimate population immunity. We searched PubMed for relevant studies published between database inception and June 4, 2021, using the terms “serotype 2 vaccine-derived poliovirus” or “type 2 vaccine-derived poliovirus” or “VDPV2”, and “immunity”, with no language restrictions. Of 19 results, three studies estimated population immunity against type 2 poliomyelitis and quantified the effects of immunity on circulating type 2 vaccine-derived poliovirus (cVDPV2) transmission. Duintjer-Tebbins and colleagues used a dynamic model to show that cVDPV2 would be more likely to sustain transmission as population immunity decreased after OPV2 withdrawal. Blake and colleagues identified risk factors for cVDPV2 detection in the 15 months after type 2 oral poliovirus (OPV2) withdrawal in Nigeria, Pakistan, Syria, and the Democratic Republic of Congo, including routine immunisation coverage and population immunity. Blake and colleagues estimated that the odds of cVDPV2 detection after OPV2 withdrawal in a province increased by 2·6 times per 10% absolute decrease in pre-withdrawal immunity. A study by Pons-Salort and colleagues showed an increasing probability of cVDPV2 detection at lower levels of population immunity in Nigeria and Pakistan between 2004 and 2015. The study showed that the proportion of districts reporting at least one cVDPV2 case in a 6-month period increased from less than 1% where population immunity was over 80% to 13% in Nigeria and 30% in Pakistan for immunity less than 20%.

**Added value of this study**

This study covers a larger population and geographical area than have previous studies identified in our literature search (more than 196 million children under 5 years across all of continental Africa) over a key period (2015−20) during which type 2 immunity has declined dramatically and cVDPV2 has spread widely. We address the effects of inactivated poliovirus vaccine (IPV) and OPV on the incidence of polio and model the spread of cVDPV2 from affected populations to closely connected susceptible ones. Our results show that immunity from both IPV and OPV decrease the incidence of cVDPV2. Notably, our findings showing the role of IPV in preventing cVDPV2 paralysis contributed to the recent WHO recommendation that all countries currently administering one IPV dose in their routine immunisation schedule should introduce a second IPV dose in 2021−22.

This work quantitatively informs optimal outbreak response strategy, showing that only 11% of children at high risk are receiving OPV within 6 months and that the geographical scope of outbreak response needs to be larger to stop cVDPV2 transmission.

**Implications of all the available evidence**

Type 2 population immunity from OPV and IPV is a key driver of cVDPV2 incidence. Population immunity can reliably be estimated from dose reporting data and immunisation coverage estimates to characterise cVDPV2 risk. IPV coverage must be strengthened, and catchup campaigns should be used to protect children who did not receive IPV because of delayed or disrupted supply. The initial findings of this analysis, in addition to work from other research groups, informed outbreak response when activities restarted in June, 2020 after COVID-19-related disruptions. Further work is needed to quantify the effects of a second IPV dose in routine immunisation. Novel OPV2, which is a new vaccine that has been engineered to be more genetically stable and thus less likely to result in further vaccine-derived poliovirus emergences, holds promise for the future of polio eradication. Nevertheless, countries must respond with vaccination campaigns of adequate geographical scope and speed to reduce cVDPV2 transmission risk. Population immunity estimates, population movement, and measures of transmission intensity should be considered when planning outbreak response.

Although eradication of type 2 wild poliovirus was certified in 2015, most cases of vaccine-derived poliomyelitis reported in the past decade have been caused by cVDPV2, defined as vaccine strains that are at least 0·6% divergent from Sabin type 2 poliovirus in the viral protein 1 genomic region with genetically linked isolates consistent with circulation. It was predicted that if countries achieved high mucosal immunity before OPV withdrawal, circulation of type 2 polioviruses would eventually cease.^3^ Any transmission of cVDPV2 detected after withdrawal would be stopped by campaigns with monovalent type 2 OPV (mOPV2), released with the authorisation of the WHO Director General. However, use of mOPV2 in outbreak response carries with it a risk of further emergences of cVDPV2, particularly where mucosal immunity to type 2 poliovirus is low,^3^, ^4^ so WHO sought to restrict use of mOPV2 to populations exposed to cVDPV2 transmission.^5^

Between 2016 and 2017, cVDPV2 transmission was restricted to just five countries: the Democratic Republic of the Congo, Nigeria, Pakistan, Somalia, and Syria. However, use of mOPV2 in outbreak response campaigns with inadequate coverage in areas of declining type 2 mucosal immunity led to further emergences of cVDPV2, which have now spread widely, with 34 countries affected as of February 2021. Over 64% of cVDPV2 cases since OPV withdrawal have occurred in Africa.^1^ The persistence of wild poliovirus in Pakistan and Afghanistan, and the emergence and spread of new cVDPV2 outbreaks in Africa, have resulted in the continued categorisation of polio as a Public Health Emergency of International Concern by WHO since 2014.^6^ Furthermore, the COVID-19 pandemic resulted in temporary suspension of vaccination campaigns and diminished surveillance, probably contributing to further spread of cVDPV2.

In an effort to mitigate the risks of type 2 OPV withdrawal, inactivated poliovirus vaccine (IPV), which protects against paralysis but offers scarce transmission-blocking mucosal immunity, was added to routine immunisation from 2014 in countries previously using only OPV.^7^ The introduction has been hindered by shortfalls in vaccine supply and low routine immunisation coverage.^8^

With the variable use of mOPV2 in outbreak response and the introduction of IPV into routine immunisation, type 2 poliovirus immunity is now highly spatially heterogeneous in Africa. This spread is influenced by variation in the coverage of campaigns and routine immunisation, the timing and geographical scope of campaigns, and the timing of IPV introduction and vaccine supply interruptions. We aimed to characterise OPV-induced and IPV-induced type 2 poliovirus population immunity subnationally across the African continent, and to quantify the relationship between population immunity and cases of cVDPV2 poliomyelitis. We aimed to use this information to assess whether the geographical scope and timing of the outbreak response has been adequate to stop the spread of cVDPV2.

## Methods

### Study design and data sources

To estimate per-dose OPV effectiveness, we used data on seroconversion after administration of trivalent OPV or mOPV2 from 38 studies across Africa and Asia, collected in a systematic review conducted in 2018.^9^, ^10^, ^11^, ^12^, ^13^

Global surveillance for poliomyelitis is done through surveillance for acute flaccid paralysis.^14^ For each individual with acute flaccid paralysis, information recorded includes the first and second administrative region (hereafter province and district, respectively) in which the individual resides, the date of onset of paralysis, the age and sex of the individual, and the reported number of OPV doses received. cVDPV2 poliomyelitis cases are confirmed through isolation and sequencing of poliovirus from two stool samples collected 48 h apart within 14 days of paralysis onset from notified acute flaccid paralysis cases. We used data from individuals with acute flaccid paralysis with paralysis onset between Jan 1, 2015, and June 30, 2020, accessed through the Polio Information System (section S1.1.2).^1^

The Global Polio Eradication Initiative maintains a calendar of implemented and planned supplementary immunisation activities (SIAs) worldwide. The calendar includes district-level information on the dates of implementation, age groups targeted, and vaccine formulation. We obtained data for SIAs implemented or planned between Jan 1, 2010, and June 30, 2020, accessed through the Polio Information System (section S1.1.2).^1^

Institutional ethics approval for this study was granted by the Imperial College Research Governance and Integrity Team (reference ID 21IC6996).

### Outcomes

We estimated the following: trivalent OPV and mOPV2 per-dose effectiveness against type 2 poliomyelitis; subnational population immunity from OPV and IPV across continental Africa for children under 5 years and under 3 years for 6-month periods between January–June, 2015, and January–June, 2020; risk factors for cVDPV2 detection and incidence; subnational cVDPV2 risk for children under 5 years for July–December 2020; number of mOPV2 SIAs required in January–June, 2020, to reduce predicted risk in July–December, 2020 to low in all locations; subnational cVDPV2 risk for children under 5 years for 6-month periods between January–June, 2016, and January–June, 2020 assuming no mOPV2 use or actual mOPV2 use in the previous 6 months.

### Statistical analyses

We estimated OPV effectiveness as a function of vaccine formulation and the national mortality rate in children under 5 years, a proxy for sanitation, by fitting a logistic regression to trivalent OPV and mOPV2 seroconversion data.

We estimated population immunity from OPV and IPV against type 2 poliomyelitis for every country in Africa (except Cabo Verde, Seychelles, and Mauritius) at different administrative divisions for children under 5 years and under 3 years for discrete 6-month periods between January–June, 2015, and January–June, 2020. We estimated population immunity from OPV on the basis of the reported number of doses of OPV received by individuals with non-polio acute flaccid paralysis, estimated vaccine efficacy, and SIA history, using methods described previously.^15^

We could not estimate population immunity from IPV directly from non-polio acute flaccid paralysis data because these do not widely report IPV doses. We, therefore, used a cohort model with a 1-month time step to estimate population immunity from IPV, assuming 60% efficacy against type 2 from a single routine dose.^16^ We estimated subnational routine coverage of IPV by scaling national IPV coverage by district-level or province-level coverage for the third dose of the diphtheria–pertussis–tetanus vaccine (delivered at the same age as IPV), accounting for the timing of IPV introduction into routine immunisation, periods in which routine IPV immunisation was halted due to supply shortages, and catchup campaigns after reintroduction. We also accounted for SIAs using IPV, assuming per-SIA coverage of 50% and 60% efficacy.

We estimated population immunity from OPV and IPV at the province level for 38 countries. For Nigeria, where there were sufficient non-polio acute flaccid paralysis data, we estimated population immunity at the district level. We estimated immunity at the national level for 12 other countries Botswana, Comoros, Djibouti, Equatorial Guinea, Eritrea, Eswatini, The Gambia, Guinea-Bissau, Lesotho, Namibia, São Tomé and Príncipe, and Tunisia with incomplete non-polio acute flaccid paralysis data. None of these 12 countries have used mOPV2 since withdrawal.

To identify factors affecting the spread of cVDPV2, we fitted a series of mixed-effects logistic regression models to routine surveillance data reporting the presence or absence of one or more cVDPV2 cases in each province of 50 African countries and each district of Nigeria for discrete 6-month periods between January–June, 2016, and January–June, 2020. Provinces *j* in each country *i* (or districts in the case of Nigeria) were classified as reporting or not reporting cVDPV2 cases at time *t*. The log-odds of reporting cases were assumed to be a function of *K* covariates *X*, and country-level and province-level time-invariant random effects *t*_i_ and *μ*_ij_, respectively.

$$\begin{array}{c} Y_{ijt}∼\text{Bernouli}(p_{ijt})\\ \text{logit}(p_{ijt})=\alpha_{ij}+\sum_{k=1}^{k=K}\beta_{k}X_{k}(i,j,t)+\mu_{ij}+\tau_{i}\\ \mu_{ij}∼\text{normal}(0,\sigma_{ij})\\ \tau_{i}∼\text{normal}(0,\rho_{i}) \end{array}$$

The first case in a genetic lineage was excluded from *Y*_ijt_ to distinguish risk factors for geographical spread from those affecting the likelihood of emergence. Models were fitted to the data using the Integrated nested laplace approximation approach, implemented in the R-INLA package version 21.02.23.^17^

We considered the following time-variant covariates in constructing our model, all in the previous 6-month periods: estimated IPV and OPV immunity, number of mOPV2 SIAs, force of infection from other districts and provinces with varying levels of international movement (appendix 2 pp 10–11), and cVDPV2 emergences and cases. We also included time-invariant demographic factors and measures of faecal-oral transmission intensity.^18^, ^19^, ^20^, ^21^ The most parsimonious yet best-fitting model was selected based on the Watanabe-Akaike Information Criterion using a stepwise addition approach.^22^

We tested whether the variables in the final model had a non-linear relationship with the log-odds of cVDPV2 spread by comparing the Watanabe-Akaike Information Criterion of models with three to five categorical variables to the best-fitting model with linear variables.

To better understand the role of different sources of IPV in containing cVDPV2 geographical spread, we substituted the number of IPV SIAs (including catchup campaigns) and national routine IPV coverage for the estimated population immunity from IPV. We also analysed the effects of IPV on cVDPV2 incidence by fitting a mixed-effects negative binomial regression model to the incidence of cVDPV2 cases. We assumed the number of observed cVDPV2 cases followed a negative binomial distribution with dispersion parameter *k* and mean *q*:

$$\begin{array}{c} Z_{ijt}∼\text{negative binomial}(q_{ijt},k)\\ \text{log}(q_{ijt})=\alpha_{ij}+\sum_{k=1}^{k=K}\beta_{k}X_{k}(i,j,t)+\mu_{ij}+\tau_{i} \end{array}$$

We used the same covariates as in the best-fitting model for spread (table 1) but did not use model selection for this model.

**Table 1:** Risk factors associated with the spread of circulating vaccine-derived type 2 poliovirus based on the best-fitting multivariable mixed-effects lagged regression model for January–June, 2016, to January–June, 2020

	**Univariable**		**Multivariable**	
	Odds ratio	95% CrI	Odds ratio	95% CrI
Emergence (previous 6 months)	78·1	(37·2–165·0)	17·7	(6·03–53·8)
Log FOI (previous 6 months)	1·57	(1·50–1·64)	1·52	(1·43–1·63)
Type 2 immunity from IPV (previous 6 months, 10% decrease, under 5 years)	0·95	(0·87–1·04)	1·21	(1·01–1·48)
Type 2 immunity from OPV (previous 6 months, 10% decrease, under 5 years)	1·50	(1·42–1·59)	1·48	(1·32–1·67)
mOPV2 rounds (previous 6 months)	0·93	(0·69–1·20)	0·30	(0·20–0·44)
Diarrhoea prevalence (10% increase, under 5 years)	1·74	(1·60–1·91)	1·59	(1·26–2·03)
Log population size (under 5 years)	2·14	(1·91–2·39)	1·42	(1·09–1·86)

Fixed effects of variables are given in brackets. Random effects variables were province (median 1·72 [95% CrI 0·83–4·18]) and country (0·55 [0·30–1·04]), with a precision of 1/variance. 95% CrI=95% credible interval. FOI=force of infection. IPV=inactivated poliovirus vaccine. OPV=oral poliovirus vaccine. mOPV2=monovalent type 2 oral poliovirus vaccine.

To test the predictive ability of the best-fitting multivariable logistic model, we did 6-months-ahead, out-of-sample predictions from January–June, 2020, refitting the model each time, and calculated the area under the curve of the receiver operating characteristic curve. We calculated the specificity of our predictions by setting the probability threshold to give a sensitivity of 80%. Locations where the 97·5th, 50th, and 2·5th percentile of the probability distribution exceeded this threshold were classified as high, medium, and low risk, respectively; those where the threshold exceeded the 2·5th percentile were classified as very low risk.

Based on the best-fit logistic model and data up to January–June, 2020, we predicted the probability of cVDPV2 cases in each 6-month period between January–June, 2017, and July–December, 2020. We classified locations as very low, low, medium, or high risk as outlined. We calculated the expected number of locations reporting cases by first calculating the probability *E*_k_ of exactly *k* provinces or districts reporting cases, and then taking the cumulative probability of *E*_k_ over all *k*.

Assuming 80% SIA coverage and mOPV2 efficacy as discussed, we calculated the number of SIAs that would have been required in January–June, 2020, to reduce the predicted risk in July–December, 2020, to low in all locations. We compared the number and location of these SIAs to those that took place in January–June, 2020. We did three sensitivity analyses, assuming decreased SIA coverage (50%); a categorical effect of one, two, or three or more mOPV2 rounds in the previous 6 months; or an additional reduction in international movement January–June, 2020, due to COVID-19 restrictions. To contextualise our findings, we calculated the number of children under 5 years who were living in districts or provinces classified by the model as being medium or high risk for each 6-month period between January–June, 2017, and July–December, 2020, and isolated those that used mOPV2 within 6 months, within 7–12 months, or not within 12 months of the risk prediction to respond to cVDPV2 outbreaks.

Full details of our statistical analyses are available in appendix 2 (pp 4−15).

### Role of the funding source

The funders of the study had no role in study design, data collection, data analysis, and data interpretation. ASB, who works for the Gates Foundation, and OM, who works in the Polio Eradication Department at WHO, contributed to critical revisions of the final manuscript.

## Results

Using data from 38 seroconversion studies from Africa and Asia, estimated per-dose efficacy for type 2 poliomyelitis was 50–67% for trivalent OPV and 68–80% for mOPV2, with higher efficacy in places with low under-5 mortality (appendix 2 p 17). 111 507 non-polio acute flaccid paralysis cases were included in the population immunity estimates, 56% from male children (appendix 2 p 49). Adjusting for age, the number of OPV doses did not differ by sex.

**Figure 1:**
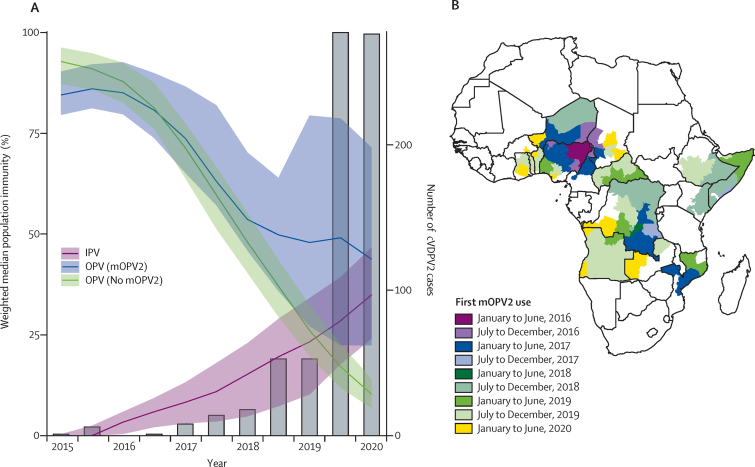
Population immunity induced by oral and inactivated poliovirus vaccine among children under 5 years and the incidence of poliomyelitis caused by cVDPV2 in Africa (A) Weighted median population immunity against type 2 poliovirus induced by OPV2 in provinces with and without mOPV2 use and IPV and annual cVDPV2 cases (grey bars) at 6-month intervals between January, 2015, and June, 2020. Shaded ribbons indicate population-weighted IQR. (B) Provinces (first-level administrative divisions) conducting mOPV2 campaigns between April 2016, and June 2020, with shading corresponding to the date of implementation of the first campaign. cVDPV2=circulating type 2 vaccine-derived poliovirus. IPV=inactivated poliovirus vaccine. mOPV2=monovalent type 2 oral poliovirus vaccine. OPV=oral poliovirus vaccine.

Type 2 population immunity from OPV (OPV2) declined from a population-weighted median of 87% (IQR 81−93) of children under 5 years in January–June, 2016, to 14% (9–37) in January–June, 2020. In provinces where no mOPV2 has been used, median population immunity from trivalent OPV declined from 88% (IQR 83−92) in children under 5 years in January–June, 2016 to 10% (7–14) in January–June, 2020 (figure 1). In provinces with mOPV2 use in the past 2 years, median OPV2 immunity was 70% (IQR 49−86). 42% of locations where mOPV2 has been used in the past 2 years (1390 of 3271) had OPV2 immunity less than 80%. At the time of OPV2 withdrawal, 79% (154 of 196 million) of children under 5 years lived in a region where OPV2 immunity was greater than 80%. In the first half of 2020, only 8% (16 of 196 million) of children under 5 years lived in a region where OPV2 immunity was greater than 80%, and 64% (125 of 196 million) lived in a region where OPV2 immunity was less than 20%.

**Figure 2:**
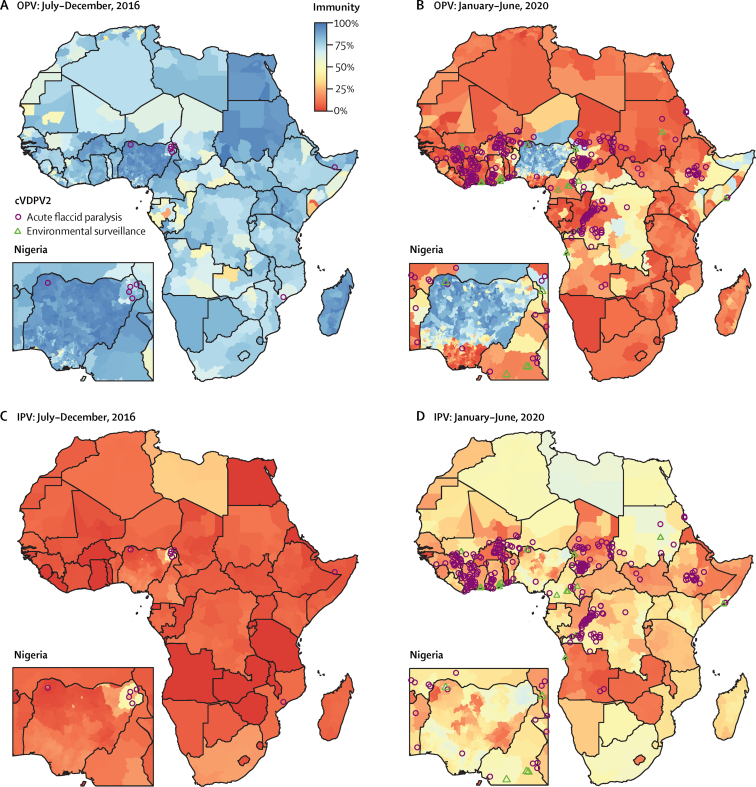
Estimated population immunity against type 2 poliomyelitis in children under 5 years induced by OPV (A–B) or IPV (C–D) Nigeria inset enlarged to show detail. cVDPV2=circulating type 2 vaccine-derived poliovirus. IPV=inactivated poliovirus vaccine. OPV=oral poliovirus vaccine.

Population immunity from IPV increased from a population-weighted median of 3% (IQR <1−6) of children under 5 years in January–June, 2016, to 35% (24−47) in January–June, 2020. The number of provinces or districts with cVDPV2 cases increased from just one of 1489 in July–December, 2016 (accounting for less than 0·03% of the 196 million of children aged under 5 years) to a maximum of 63 (16%) in January–June, 2020. Between January and June, 2020, there were 34 million children under 5 years living in Africa in provinces where cVDPV2 was detected (figure 2). We estimate that 27 million (80%) of these have no immunity to type 2 poliovirus from OPV and 24 million (70%) have no immunity from IPV.

The probability of cVDPV2 poliomyelitis among children under 5 years was negatively correlated with OPV-induced and IPV-induced immunity and mOPV2 campaigns (table 1). Adjusted odds ratios were 0·68 (95% CrI 0·60−0·76) for OPV and 0·82 (0·68−0·99) for IPV per 10% absolute increase in estimated population immunity, and 0·30 (0·20−0·44) for mOPV2 per campaign.

cVDPV2 cases were more common in densely populated provinces with a high prevalence of diarrhoeal disease, with a novel cVDPV2 emergence in the previous 6 months, and if exposed to a high force of infection from other provinces (table 1). The estimated force of infection based on a 75% relative decrease in movement across international borders produced the best fit to the data. Increased population immunity from IPV and OPV reduced the odds of a case being reported. The number of mOPV2 campaigns done in the preceding 6 months was also associated with an additional reduction in the odds of a case being reported. Thus, a single mOPV2 round achieving 70% coverage would result in a predicted 15·1-times decrease in the odds of cVDPV2 detection as a result of an absolute increase in OPV immunity of 39% (4·6-times decrease) plus the additional effects of an mOPV2 round not measured by the OPV immunity estimate (additional 3·3-times decrease).

We did not find any difference in the effects of IPV campaigns compared with routine coverage (appendix 2 p 19). Using a categorical variable for mOPV2 rounds improved the fit of the model but did not change our overall findings (appendix 2 p 32). The best-fitting model predicted the presence of cVDPV2 cases with 97% specificity and a positive predictive value of 31%, when fixing the sensitivity at 80%. The model also performed well in sequential cross-validation, with the area under the curve increasing from 0·71 to 0·89 between July–December, 2018, and January–June, 2020 (appendix 2 p 46).

We also showed that immunity from IPV has a significant effect on reducing the incidence of cVDPV2 cases, with a 1·3-fold decrease in incidence per 10% absolute increase in population immunity (adjusted incidence rate ratio 0·79, 95% CrI 0·64−0·95; table 2). This is similar in magnitude to the effect of immunity from OPV (1·5-times decrease or aIRR 0·65 [95% CrI 0·59−0·72]).

**Table 2:** Risk factors associated with incidence of circulating vaccine-derived poliovirus type 2 cases based on multivariable mixed-effects lagged regression model for January–June, 2016, to January–June, 2020

	**Univariable**		**Multivariable**	
	IRR	95% CrI	IRR	95% CrI
Emergence (previous 6 months)	26·70	(6·37–181·00)	9·32	(3·35–31·20)
Log FOI (previous 6 months)	1·58	(1·43–1·96)	1·49	(1·26–1·67)
Type 2 immunity IPV (previous 6 months, 10% increase)	1·65	(1·42–1·93)	0·787	(0·644–0·950)
Type 2 immunity OPV (previous 6 months, 10% increase)	0·433	(0·197–0·992)	0·654	(0·588–0·724)
mOPV2 rounds (previous 6 months)	0·710	(0·486–1·020)	0·364	(0·235–0·571)
Diarrhoea prevalence (10% increase)	1·61	(1·27–2·07)	1·73	(1·41–2·14)

Fixed effects of variables are given in brackets. Random effects variables were province (median 3·39 [95% CrI 1·51–9·54]) and country (1·36 [0·86–2·23]), with a precision of 1/variance. The median dispersion parameter (1/k) was 0·195 (95% CrI 0·140–0·264). 95% CrI=95% credible interval. FOI=force of infection. IPV=inactivated poliovirus vaccine. IRR=incidence rate ratio. mOPV2=monovalent type 2 oral poliovirus vaccine. OPV=oral poliovirus vaccine.

**Figure 3:**
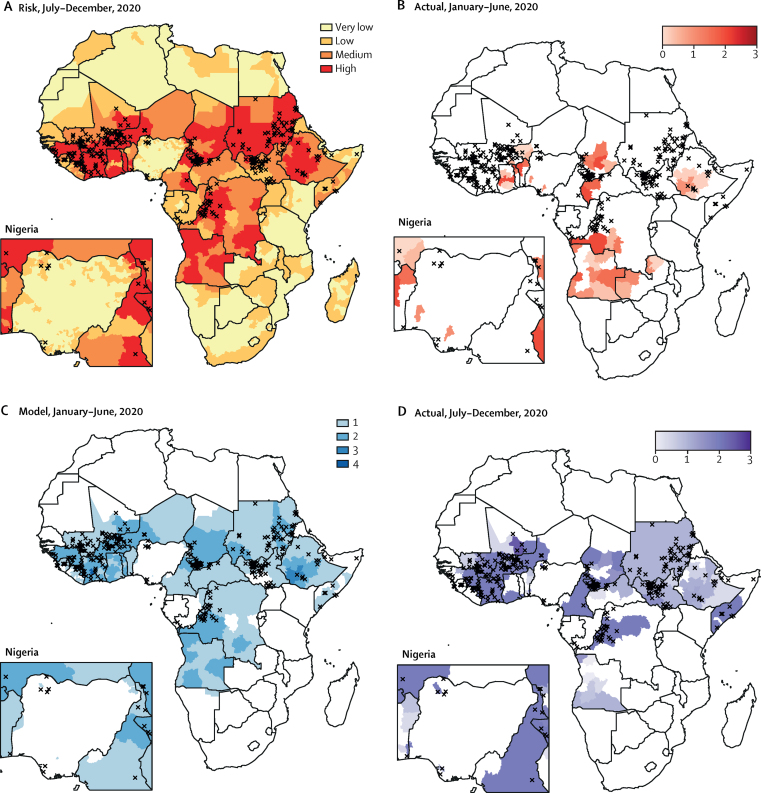
cVDPV2 risk (A) and number of mOPV2 SIAs required to reduce risk to low levels (B–D) in 2020 (A) Predicted risk of detecting a cVDPV2 case between July 1 and Dec 31, 2020. Risks were categorised as follows: very low, 97·5th percentile of risk less than threshold (probability 0·07); low, 50th percentile of risk less than threshold; medium, 50th percentile of risk greater than threshold; and high, 2·5th percentile of risk greater than threshold. (B) Number of mOPV2 SIAs (proportion of all children under 5 years targeted) delivered between Jan 1 and June 30, 2020. (C) Number of mOPV2 SIAs (single campaign covering all children under 5 years) required between Jan 1 and June 30, 2020, to reduce predicted risk in all locations to low or very low levels. (D) mOPV2 SIAs (proportion of all children under 5 years targeted) completed or planned between July 1 and Dec 31, 2020. Cases of circulating vaccine-derived type 2 poliomyelitis between July 1 and Dec 31, 2020 are shown as black crosses. Nigeria inset enlarged to show detail. cVDPV2=circulating type 2 vaccine-derived poliovirus. mOPV2=monovalent type 2 oral poliovirus vaccine. SIAs=supplementary immunisation activities.

Using the model for cVDPV2 spread summarised in table 1, we predicted the probability of observing cVDPV2 cases in 1489 districts or provinces in Africa between July and December, 2020. Using these probabilities, we classified 1011 districts or provinces as very low risk for cVDPV2 spread, 240 as low risk, 88 as medium risk, and 150 as high risk (figure 3A). The districts and provinces at very low risk tended to have lower population densities, containing 37% of the 196 million children under 5 years, with those at low, medium, and high risk accounting for 21%, 13% and 28% of the population, respectively. 19 million doses of mOPV2 were delivered in the first half of 2020 in response to cVDPV2 outbreaks (figure 3B). For this level of response, our model predicts 121 districts or provinces (95% CrI 107−136) would report cVDPV2 spread in the second half of 2020. We predict that 129 million doses would have been required to reduce the risk to low levels across the entire continent (figure 3C), reducing the expected number of districts or provinces with spread to 20 (95% CrI 12−29). Sensitivity analysis to model assumptions showed the required number of doses to vary between 104 million (assuming non-linear impact of SIAs) and 140 million doses (assuming a lower SIA coverage of 50%; appendix 2 p 49). Assuming decreased international movement January–June, 2020 due to COVID-19 had a negligible effect on required doses (appendix 2 p 49). An additional 79 million doses of mOPV2 were delivered between July and December, 2020 (figure 3D).

**Figure 4:**
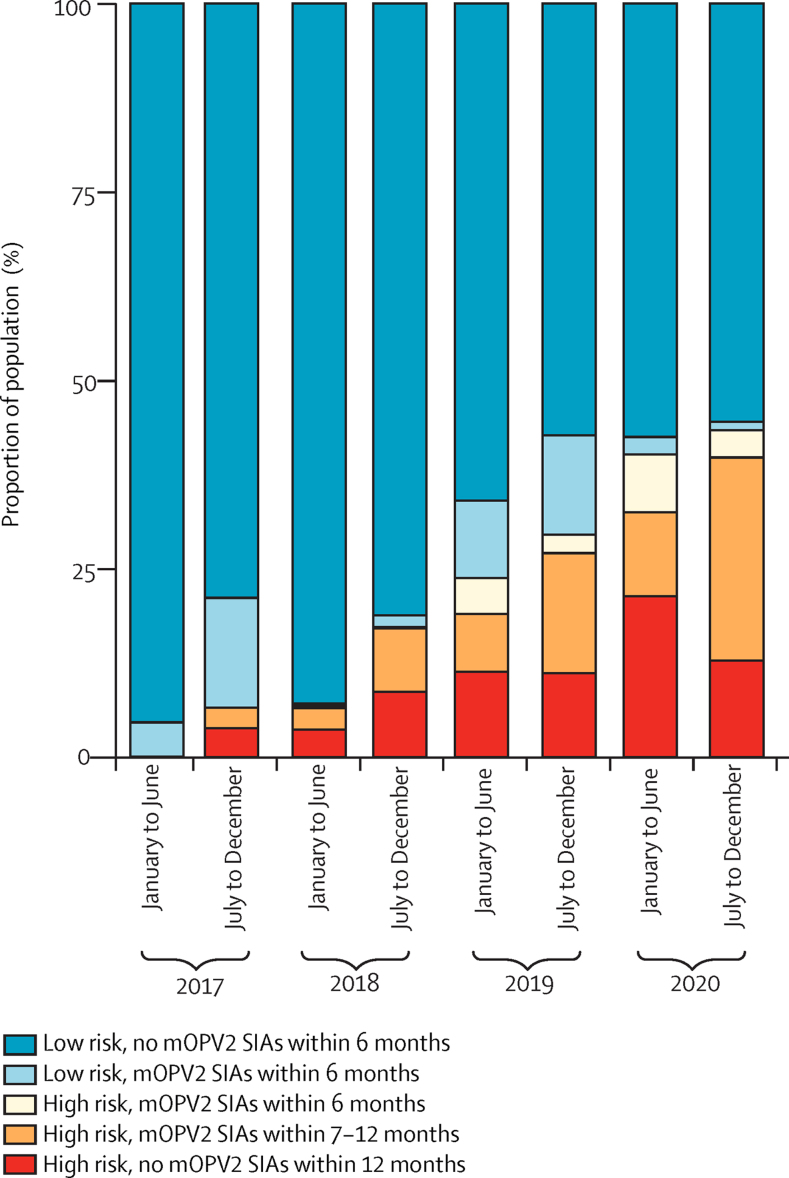
Comparison of mOPV2 SIAs and predicted circulating vaccine-derived type 2 risk in children under 5 years, 2017–2020 cVDPV2=circulating type 2 vaccine-derived poliovirus. mOPV2=monovalent type 2 oral poliovirus vaccine. SIAs=supplementary immunisation activities. Low risk=low or very low risk. High risk=high or medium risk.

The proportion of the children under 5 years living in locations of high or medium risk of reporting cVDPV2 cases increased from 0% of 196 million in January–June, 2017, to 42% (82 million of 196 million) in July–December, 2020. Between January, 2017, and December, 2020, we estimated that 408 district-level or province-level 6-month periods were at high risk of reporting cVDPV2 cases and 509 were at medium risk. Of these 917 at-risk periods, only 138 used mOPV2 within 6 months (accounting for 11% of 164 million at-risk child-years in children aged under 5 years across the 6-month periods) and 413 used mOPV2 within 7–12 months (45% of 164 million child-years; figure 4). This shortfall was consistent over time; in every 6-month period, the proportion of provinces at high or medium risk of reporting cVDPV2 cases using mOPV2 within 6 months was less than 20%.

## Discussion

The rapid expansion of cVDPV2 transmission is threatening the goal of global poliovirus eradication. If transmission of cVDPV2 is not interrupted and becomes too widespread, type 2 containing live vaccine might have to be reintroduced into routine immunisation, representing a major step backwards from the goal of eradicating all polioviruses. Previous studies have quantified the relationship between low population immunity from OPV2 and increased risk of cVDPV2 outbreaks.^15^, ^23^ Our analysis showed that more places in Africa are at risk of cVDPV2 transmission than ever before and that outbreak response activities have been too small and too slow to stop the spread of cVDPV2. As anticipated, type 2 immunity from OPV drastically declined after OPV withdrawal.^3^, ^4^ Although immunity from IPV has increased since 2016, it is not high enough to completely offset the decrease in OPV immunity and does not induce the mucosal immunity that is effective against poliovirus transmission. Even in places where mOPV2 has recently been used in outbreak response, OPV immunity is still not as high as it was before withdrawal.

We estimated that 129 million doses of mOPV2 would have been required in the first half of 2020; however, only 19 million were delivered. An additional 79 million doses of mOPV2 were delivered in the second half of 2020. Although the total number of doses (98 million) is closer to the level indicated by our model, most of these doses were delivered in late 2020, by which time the virus might have spread outside the response zones. This delay was in part the unavoidable result of restrictions designed to mitigate the COVID-19 pandemic, but this trend is consistent with previous years, with 43% of the 164 million child-years at risk of cVDPV2 between July, 2016, and December, 2020 not receiving mOPV2 SIAs within 12 months.

As predicted by theoretical models, responding to cVDPV2 outbreaks has become more challenging after the withdrawal of OPV2.^4^, ^24^ As population immunity declines, cVDPV2 spreads rapidly and outbreak response campaigns need to be faster and geographically larger to contain transmission. However, larger campaigns increase the number of children shedding OPV2, which might cause further emergences of cVDPV2. The outbreak response has been too conservative in scope or too slow (or both), in part due to shortages in the mOPV2 stockpile.^25^ In November, 2020, emergency use licensure was granted to a novel type 2 oral poliovirus vaccine (nOPV2), which is designed to be more genetically stable than the Sabin strain and is expected to have a reduced risk of causing new cVDPV2 outbreaks.^26^ If effectiveness and genetic stability in the field are consistent with the indications of clinical trials,^27^ nOPV2 will facilitate more robust responses to outbreaks, with reduced risk of new emergences.

The COVID-19 pandemic presents additional challenges to the cVDPV2 outbreak response. When the pandemic was declared in March, 2020, all outbreak response activities were suspended by the Global Polio Eradication Initiative for several months to reduce the risk of spreading SARS-CoV-2. Although there was initial speculation that movement and contact restrictions might reduce poliovirus transmission intensity, there is no evidence to this effect. Border closures delayed the confirmation of cVDPV2 isolates via sequencing in international reference laboratories. This disruption allowed for the further spread of cVDPV2. In Sudan, for example, a case of cVDPV2 paralysis occurred in South Darfur in March, 2020, but this case was not confirmed until August due to shipping delays and the vaccination response did not begin until October. In Cameroon, three cVDPV2 cases occurred in early 2020, with confirmation by April, but outbreak response was delayed until September due to the pandemic.

Despite initial challenges with supply, all countries globally have now introduced IPV into routine immunisation and most have implemented catchup campaigns where needed.^8^ However, we showed that immunity is low in many settings because of low routine immunisation coverage and the delivery of just a single dose. Routine delivery of two doses of IPV would provide substantially higher immunogenicity, especially in delayed schedules.^16^, ^28^ Our findings showing the additional role of IPV in preventing cVDPV2 paralysis contributed to the WHO recommendation in April, 2020 that all countries administering only one IPV dose in their routine immunisation schedule should introduce a second IPV dose in 2021−22.^25^ Further analysis is needed to quantify the effects and cost-effectiveness of a second IPV dose. In addition, routine immunisation coverage must be strengthened, and catchup campaigns should be implemented for children who did not receive IPV because of poor coverage or delayed and disrupted IPV supply.

Although IPV primarily induces a humoral response, administration of IPV after previous immunisation with OPV can boost waned mucosal immunity.^29^ We did not consider this effect in our analysis. We expect the boosting effects from IPV on type 2 mucosal immunity in Africa in this time period to be negligible for three reasons. First, doses delivered via routine immunisation from April, 2016 targeted OPV2-naive children. Second, IPV catchup campaigns were scarce in this time period and targeted mostly OPV2-naive children, such that less than 2% of the 196 million study population (aged <5 years) would be expected to have received OPV2 and also receive an IPV catchup dose in any given 6-month period. Third, other SIAs with IPV that might have reached some children previously vaccinated with mOPV2 or trivalent OPV were restricted to six local government areas in northern Nigeria, targeting less than 4% of the study population in any given 6-month period. Our analysis does not provide evidence to suggest that IPV coverage has any effect on the transmission of cVDPV2. More widespread environmental sampling could show whether cVDPV2 continues to circulate in settings with moderate type 2 IPV immunity but low type 2 OPV immunity. This information is particularly relevant to countries such as Egypt and Iran, where cVDPV2 has been detected in sewage and type 2 population immunity from OPV is low but IPV coverage is relatively high.

Our analysis has some limitations. First, estimates of OPV immunity rely on recall of the number of doses received by children with non-polio acute flaccid paralysis, which could be inaccurate or biased.^30^ Furthermore, in locations where the non-polio acute flaccid paralysis rate is low or campaigns occurred late in the 6-month period, immunity estimates might not capture the effects of recent campaigns. For this reason, we have included the number of mOPV2 campaigns in the previous 6 months as an independent variable in our regression model. We did not include any data on SIA coverage, which can vary between campaigns and countries, because coverage surveys are not routinely done across Africa. In places where the non-polio acute flaccid paralysis rate is particularly low, immunity estimates could also be affected by sampling noise. Second, we used a single value for the relative decrease in movement across international borders. In reality, intracontinental migration varies in space and over time.^31^ Finally, we were unable to use non-polio acute flaccid paralysis dose reporting data to inform our IPV immunity estimates because IPV dose reporting in Africa regions is still too incomplete. Our IPV immunity estimates, therefore, rely heavily on subnational estimates of diphtheria–pertussis–tetanus vaccine coverage.

The COVID-19 pandemic still threatens to disrupt the polio outbreak response in Africa. Another extended pause would allow cVDPV2 to spread to new populations with little to no immunity to type 2 poliomyelitis. Nonetheless, nOPV2 holds promise for the future of polio eradication. Future studies are needed to understand the effectiveness and stability of nOPV2, but even under optimal conditions, cVDPV2 transmission will only be stopped if countries respond with high quality vaccination campaigns of adequate geographical scope and speed.

## Data sharing

The aggregated immunity estimates, other model covariates, and estimated risk of cVDPV2 used in this analysis will be available to download upon publication from the corresponding author's GitHub page at https://github.com/lvcooper/polio-st2immunity.

**This online publication has been corrected. The corrected version first appeared at thelancet.com/infection on January 26, 2022**

## Declaration of interests

We declare no competing interests.

## Supplementary Materials

French translation of the abstract
