# Correction to *Lancet Infect Dis* 2022; 22: 284–94

**DOI:** 10.1016/S1473-3099(22)00010-X

**Published:** 2022-02

*Cooper LV, Bandyopadhyay AS, Gumede N, et al. Risk factors for the spread of vaccine-derived type 2 polioviruses after global withdrawal of trivalent oral poliovirus vaccine and the effects of outbreak responses with monovalent vaccine: a retrospective analysis of surveillance data for 51 countries in Africa.* Lancet Infect Dis *2022; **22:** 284–94*—In this Article, “10% increase” on rows 2 and 3 of table 1 has been corrected to “10% decrease”, the labels of panels B and C in the legend of figure 3 have been swapped, and reference 26 has been replaced to correct a citation error. These corrections have been made to the online version as of Jan 26, 2022, and the printed article is correct.

