# Supplementary material for: Risk factors for the spread of vaccine-derived type 2 polioviruses after global withdrawal of trivalent oral poliovirus vaccine and the effects of outbreak responses with monovalent vaccine: a retrospective analysis of surveillance data for 51 countries in Africa
Source: Lancet Infect Dis. 2022 Feb;22(2):284–94. doi: 10.1016/S1473-3099(21)00453-9 (PMC8799632; doi:10.1016/S1473-3099(21)00453-9)
Supplement: French translation of the abstract [file mmc1.pdf]

THE LANCET
Infectious Diseases

Supplementary appendix 1

This translation in French was submitted by the authors and we reproduce it as supplied. It has not been peer reviewed. *The Lancet*'s editorial processes have only been applied to the original in English, which should serve as reference for this manuscript.

Supplement to: Cooper LV, Bandyopadhyay AS, Gumede N, et al. Risk factors for the spread of vaccine-derived type 2 polioviruses after global withdrawal of trivalent oral poliovirus vaccine and the effects of outbreak responses with monovalent vaccine: a retrospective analysis of surveillance data for 51 countries in Africa. *Lancet Infect Dis* 2021; published online Oct 11. https://doi.org/10.1016/S1473-3099(21)00453-9.

Cette traduction en français a été proposée par les auteurs et nous l'avons reproduite telle quelle. Elle n'a pas été examinée par des pairs. Les processus éditoriaux du *Lancet* n'ont été appliqués qu'à l'original en anglais et c'est cette version qui doit servir de référence pour ce manuscrit.

Sommaire

Contexte

L'expansion des épidémies de poliovirus circulants dérivés d'une souche vaccinale type 2 (PDSVc2) en Afrique après le retrait mondial du vaccin antipoliomyélitique oral (VPO) trivalent en 2016 retarde l'éradication mondiale de la poliomyélite. Notre objectif était d'évaluer l'effet des campagnes de riposte aux flambées avec le VPO monovalent type 2 (VPOm2) et l'ajout du vaccin antipoliomyélitique inactivé (VPI) à la vaccination systématique.

Méthodes

Nous avons utilisé les données d'historique de vaccination d'enfants de moins de 5 ans atteints de paralysie flasque aiguë non poliomyélitique provenant d'une base de données de surveillance routine (le Polio Information System) et les données d'immunogénicité du VPO de la littérature pour estimer l'immunité de la population conférée par VPO et VPI contre la poliomyélite type 2 entre le 1er janvier 2015 et le 30 juin 2020 dans 51 pays Africains. Nous avons étudié les facteurs de risque de poliomyélite PDSVc2 rapportée, notamment l'immunité de la population, les activités de riposte aux flambées et les corrélats de la transmission du poliovirus à l'aide de la régression logistique. Nous avons utilisé le modèle pour estimer le risque de PDSVc2 pour chaque période de 6 mois entre le 1er janvier 2016 et le 30 juin 2020, avec différents nombres de campagnes de VPOm2 et comparé le moment et l'emplacement des campagnes de VPOm2 réelles et le nombre de campagnes de VPOm2 requis pour réduire le risque de PDSVc2 au niveau faible.

Résultats

L'immunité VPO type 2 chez les enfants de moins de 5 ans est passée d'une médiane de 87 % (IQR 81-93) en janvier-juin 2016 à 14 % (9-37) en janvier-juin 2020. L'immunité VPI type 2 chez les enfants de moins de 5 ans est passé de 3 % (<1 à 6 %) en janvier-juin 2016 à 35 % (24-47) en janvier-juin 2020. La probabilité de poliomyélite PDSVc2 chez les enfants de moins de 5 ans était négativement corrélée avec l'immunité conférée par le VPO et le VPI et campagnes VPOm2 (rapport de cotes ajusté : VPO 0,68 [ICr à 95 % 0,60−0·76], VPI 0,82 [0,68−0,99] pour 10 % augmentation absolu de l'immunité estimée de la population, VPOm2 0,30 [0,20−0,44] par campagne). Les campagnes de vaccination en réponse aux épidémies de PDSVc2 ont été plus petites et plus lentes que ce que notre modèle montre serait nécessaire pour réduire le risque à des niveaux faibles, couvrant seulement 11% des enfants de moins de 5 ans qui devraient être à risque dans les 6 mois et seulement 56% dans les 12 mois.

Interprétation

Nos résultats suggèrent qu'à mesure que l'immunité muqueuse diminue, des réponses plus grandes ou plus rapides avec des campagnes de vaccination utilisant le VPO type 2 seront nécessaires pour arrêter la transmission du PDSVc2. L'immunité conférée par le VPI joue également un rôle important dans la réduction du fardeau de la poliomyélite PDSVc2 en Afrique.

Financement

La Fondation Bill & Melinda Gates, le Centre du Conseil de Recherches Médicales pour l'Analyse Mondiale des Maladies Infectieuses (MRC-GIDA) et de l'OMS.
