# Supplementary material for: Risk factors for the spread of vaccine-derived type 2 polioviruses after global withdrawal of trivalent oral poliovirus vaccine and the effects of outbreak responses with monovalent vaccine: a retrospective analysis of surveillance data for 51 countries in Africa
Source: Lancet Infect Dis. 2022 Feb;22(2):284–94. doi: 10.1016/S1473-3099(21)00453-9 (PMC8799632; doi:10.1016/S1473-3099(21)00453-9)
Supplement: Supplementary appendix 2 [file mmc2.pdf]

THE LANCET
Infectious Diseases

Supplementary appendix 2

This appendix formed part of the original submission and has been peer reviewed. We post it as supplied by the authors.

Supplement to: Cooper LV, Bandyopadhyay AS, Gumede N, et al. Risk factors for the spread of vaccine-derived type 2 polioviruses after global withdrawal of trivalent oral poliovirus vaccine and the effects of outbreak responses with monovalent vaccine: a retrospective analysis of surveillance data for 51 countries in Africa. *Lancet Infect Dis* 2021; published online Oct 11. https://doi.org/10.1016/S1473-3099(21)00453-9.

S1 Text - Supplementary Information
Spread of vaccine-derived type 2 polioviruses in Africa following global withdrawal of trivalent oral poliovirus vaccine: risk factors, short-term projections, and impact of outbreak response with monovalent vaccine

Laura V Cooper, Ananda Bandyopadhyay, Nicksy Gumede-Moeletsi,
Ondrej Mach, Pascal Mkanda, Modjirom Ndoutabé, Samuel O Okiror,
Alejandro Ramirez-Gonzalez, Kebba Touray, Sarah Wanyoike, Nicholas C Grassly, Isobel Blake

Contents

1 Supplementary methods **4**
- 1.1 Estimating population immunity to type 2 poliomyelitis . . . 4
  - 1.1.1 Global meta-analysis of tOPV and mOPV2 per-dose efficacy . . . 4
  - 1.1.2 Crude estimates of population immunity by location and 6-month period . . . 5
  - 1.1.3 Hierarchical temporal models of population immunity . . . 6
  - 1.1.4 Accounting for IPV . . . 8
  - 1.1.5 Mucosal and humoral population immunity . . . 9
- 1.2 Spatial regression analysis . . . 10
  - 1.2.1 Model structure . . . 10
  - 1.2.2 Covariates . . . 10
  - 1.2.3 Model selection . . . 11
  - 1.2.4 Sensitivity to model priors . . . 14
  - 1.2.5 Non-linear effects . . . 14
  - 1.2.6 Sensitivity to administrative unit . . . 14
  - 1.2.7 Sensitivity and specificity . . . 14
  - 1.2.8 Expected cumulative probability of cVDPV2 detection . . . 15
  - 1.2.9 Model cross-validation . . . 15
  - 1.2.10 Projections for 1 July - 31 December 2020 . . . 15
  - 1.2.11 Retrospective cVDPV2 risk for 1 January 2017 - 30 June 2020 . . . 16

2 Supplementary results **16**

3 Appendix: OPV immunity time series **51**

List of Tables

- S1 Covariates in model selection. . . . 13
- S2 Summary of model selection process. . . . 13
- S3 Odds ratios of seroconversion by vaccine formulation. . . . 16
- S4 Probability of seroconversion by vaccine formulation. . . . 17
- S5 Seroprevalence against type 2 poliovirus by age, time period, and location. . . . 18
- S6 Risk factors for cVDPV2 spread using IPV rounds and routine IPV coverage. . . . 19
- S7 Risk factors for cVDPV2 spread province-level averages for observations in Nigeria. . . . 19
- S8 Risk factors for cVDPV2 spread using a categorical variable for the force of infection. . . . 20
- S9 Risk factors for cVDPV2 spread using a categorical variable for type 2 OPV immunity. . . . 20

S10 Risk factors for cVDPV2 spread using a categorical variable for mOPV2 rounds. 32
S11 Risk factors for cVDPV2 spread using a categorical variable for type 2 IPV immunity. 47
S12 Sensitivity analysis for model predictions, July to December 2020. 47
S13 Comparison of actual outbreak response January-June 2020 and July-December 2020 and model-defined optimal response. 49
S14 Mean reported OPV doses by sex and age. 49

List of Figures

S1 mOPV2 campaigns by six-month period, January 2015 to June 2020. 21
S2 IPV campaigns by six-month period, January 2015 to June 2020. 22
S3 IPV catch-up campaigns by six-month period, January 2015 to June 2020. 23
S4 Routine IPV coverage by six-month period, January 2015 to June 2020. 24
S5 Under-three type 2 population immunity from OPV by six-month period, January 2015 to June 2020. 25
S6 Under-five type 2 population immunity from OPV by six-month period, January 2015 to June 2020. 26
S7 Under-three type 2 population immunity from IPV by six-month period, January 2015 to June 2020. 27
S8 Under-five type 2 population immunity from IPV by six-month period, January 2015 to June 2020. 28
S9 Estimated humoral and mucosal population immunity against type 2 poliomyelitis in children under five years. 29
S10 Estimated humoral, mucosal, OPV and IPV population immunity against type 2 poliomyelitis in children under five years, 2016 to 2020. 30
S11 Seroprevalence against type 2 poliovirus by age group and location. 31
S12 Time-invariant covariates used in model selection. 32
S13 cVDPV2 cases by six-month period, January 2015 to June 2020. 33
S14 Force of infection with 0% reduction in international movement. 34
S15 Force of infection with 0% reduction in international movement using urban population. 35
S16 Force of infection with 25% reduction in international movement. 36
S17 Force of infection with 25% reduction in international movement using urban population. 37
S18 Force of infection with 50% reduction in international movement. 38
S19 Force of infection with 50% reduction in international movement using urban population. 39
S20 Force of infection with 75% reduction in international movement. 40
S21 Force of infection with 75% reduction in international movement using urban population. 41
S22 Force of infection with 99.9% reduction in international movement. 42
S23 Force of infection with 99.9% reduction in international movement using urban population. . . . 43
S24 Prior sensitivity. 44
S25 Province- and country-level random effects. 44
S26 Predicted cVDPV2 risk, January 2016 to December 2020. 45
S27 Six-month-ahead out-of-sample predicted cVDPV2 risk, July 2018 to June 2020. 46
S28 Sensitivity of fixed effects to Jan-Jun 2020 period. 47
S29 Comparison of mOPV2 SIAs and predicted cVDPV2 risk, July 2016 to December 2020. 48
S30 Type 2 population immunity from OPV - Abia, Nigeria. 52
S31 Type 2 population immunity from OPV - Adamawa, Nigeria. 53
S32 Type 2 population immunity from OPV - Akwa Ibom, Nigeria. 54
S33 Type 2 population immunity from OPV - Anambra, Nigeria. 55
S34 Type 2 population immunity from OPV - Bauchi, Nigeria. 56
S35 Type 2 population immunity from OPV - Bayelsa, Nigeria. 57
S36 Type 2 population immunity from OPV - Benue, Nigeria. 58
S37 Type 2 population immunity from OPV - Borno, Nigeria. 59
S38 Type 2 population immunity from OPV - Cross River, Nigeria. 60
S39 Type 2 population immunity from OPV - Delta, Nigeria. 61
S40 Type 2 population immunity from OPV - Ebonyi, Nigeria. 62
S41 Type 2 population immunity from OPV - Edo, Nigeria. 63
S42 Type 2 population immunity from OPV - Ekiti, Nigeria. 64
S43 Type 2 population immunity from OPV - Enugu, Nigeria. 65
S44 Type 2 population immunity from OPV - Federal Capital Territory, Nigeria. 66
S45 Type 2 population immunity from OPV - Gombe, Nigeria. 67
S46 Type 2 population immunity from OPV - Imo, Nigeria. 68

S47 Type 2 population immunity from OPV - Kaduna, Nigeria. 69
S48 Type 2 population immunity from OPV - Kano, Nigeria. 70
S49 Type 2 population immunity from OPV - Katsina, Nigeria. 71
S50 Type 2 population immunity from OPV - Kebbi, Nigeria. 72
S51 Type 2 population immunity from OPV - Kogi, Nigeria. 73
S52 Type 2 population immunity from OPV - Kwara, Nigeria. 74
S53 Type 2 population immunity from OPV - Lagos, Nigeria. 75
S54 Type 2 population immunity from OPV - Nasawara, Nigeria. 76
S55 Type 2 population immunity from OPV - Niger, Nigeria. 77
S56 Type 2 population immunity from OPV - Ogun, Nigeria. 78
S57 Type 2 population immunity from OPV - Ondo, Nigeria. 79
S58 Type 2 population immunity from OPV - Osun, Nigeria. 80
S59 Type 2 population immunity from OPV - Oyo, Nigeria. 81
S60 Type 2 population immunity from OPV - Plateau, Nigeria. 82
S61 Type 2 population immunity from OPV - Rivers, Nigeria. 83
S62 Type 2 population immunity from OPV - Sokoto, Nigeria. 84
S63 Type 2 population immunity from OPV - Taraba, Nigeria. 85
S64 Type 2 population immunity from OPV - Yobe, Nigeria. 86
S65 Type 2 population immunity from OPV - Zamfara, Nigeria. 87
S66 Type 2 population immunity from OPV - Algeria. 88
S67 Type 2 population immunity from OPV - Angola. 89
S68 Type 2 population immunity from OPV - Benin. 90
S69 Type 2 population immunity from OPV - Burkina Faso. 91
S70 Type 2 population immunity from OPV - Burundi. 92
S71 Type 2 population immunity from OPV - Cameroon. 93
S72 Type 2 population immunity from OPV - Central African Republic. 94
S73 Type 2 population immunity from OPV - Chad. 95
S74 Type 2 population immunity from OPV - Congo. 96
S75 Type 2 population immunity from OPV - Cote d'Ivoire. 97
S76 Type 2 population immunity from OPV - Democratic Republic of the Congo. 98
S77 Type 2 population immunity from OPV - Egypt. 99
S78 Type 2 population immunity from OPV - Ethiopia. 100
S79 Type 2 population immunity from OPV - Gabon. 101
S80 Type 2 population immunity from OPV - Ghana. 102
S81 Type 2 population immunity from OPV - Guinea. 103
S82 Type 2 population immunity from OPV - Kenya. 104
S83 Type 2 population immunity from OPV - Liberia. 105
S84 Type 2 population immunity from OPV - Libya. 106
S85 Type 2 population immunity from OPV - Madagascar. 107
S86 Type 2 population immunity from OPV - Malawi. 108
S87 Type 2 population immunity from OPV - Mali. 109
S88 Type 2 population immunity from OPV - Mauritania. 110
S89 Type 2 population immunity from OPV - Morocco. 111
S90 Type 2 population immunity from OPV - Mozambique. 112
S91 Type 2 population immunity from OPV - Niger. 113
S92 Type 2 population immunity from OPV - Rwanda. 114
S93 Type 2 population immunity from OPV - Senegal. 115
S94 Type 2 population immunity from OPV - Sierra Leone. 116
S95 Type 2 population immunity from OPV - Somalia. 117
S96 Type 2 population immunity from OPV - South Africa. 118
S97 Type 2 population immunity from OPV - South Sudan. 119
S98 Type 2 population immunity from OPV - Sudan. 120
S99 Type 2 population immunity from OPV - Togo. 121
S100 Type 2 population immunity from OPV - Uganda. 122
S101 Type 2 population immunity from OPV - Tanzania. 123
S102 Type 2 population immunity from OPV - Zambia. 124
S103 National-level estimates of type 2 population immunity from OPV. 125

1 Supplementary methods

1.1 Estimating population immunity to type 2 poliomyelitis

1.1.1 Global meta-analysis of tOPV and mOPV2 per-dose efficacy

It is well established that oral poliovirus vaccine (OPV) effectiveness varies across countries, whereby it is generally lower in lower-income settings although the reasons for this phenomenon have not been fully established.[1,2] We sought to estimate effectiveness of trivalent OPV (tOPV) and monovalent type 2 OPV (mOPV2) against type 2 poliomyelitis as a function of national under-five mortality (a proxy measure for the level of country development and sanitation). We compiled a review of studies that assessed seroconversion against type 2 poliovirus following three doses of tOPV based on three already published reviews.[3–5] Studies were selected in which the first dose of tOPV was administered at 4 weeks of age or later, and those that were performed in Africa, Asia, or Latin America (N = 35). Seroconversion after three doses was recorded as this was the most common dose regime across multiple countries for this vaccine formulation.

A review of studies measuring seroconversion after mOPV2 administration was also performed. This vaccine has had comparatively limited use compared to tOPV and fewer seroconversion studies have been conducted where the first dose was administered at 4 weeks of age or later.[6–8] Additional studies have been performed but were not included in our analysis as either the study participants were not randomised to a particular vaccine schedule[9] or a non-standard dose of mOPV2 was administered.[10] The studies measuring mOPV2 seroconversion administered either 1 or 2 doses of mOPV2. OPV is considered to induce an all-or-nothing response and therefore we converted the observed seroconversion after 1 P_1 or 2 P_2 doses of mOPV2 into the expected seroconversion P_3 after three doses of mOPV2 to ensure the seroconversion data was comparable to that of tOPV:

$$P_3 = 1 - (1 - P_1)^3$$

where for the study which measured seroconversion after two doses:

$$P_1 = 1 - \sqrt[2]{1 - P_2}$$

For each seroconversion study, i, the country the study population resided in, C, the year the study was performed, Y, the number of study participants, N, the vaccine formulation administered (V = tOPV or mOPV2), and the number of participants seroconverting after 3 doses was recorded, S. We then recorded the estimated under-five mortality rate (number of deaths per 1000 live births) corresponding to study country and year, $M_{C,Y}$.[11]

To quantify the relationship between seroconversion after 3 doses of either tOPV or mOPV2 and the under-five mortality rate we used logistic regression whereby:

$$S_{C,Y,i} \sim Binomial(N_i, x_{C,Y,i})$$
$$logit(x_{C,Y,i}) = \beta_0 + \beta_1 V_i + \beta_2 M_{C,Y}$$

We examined inclusion of the under-five mortality rate as a continuous variable on either the natural scale or the log scale, or as a categorical variable (<25, 25-75, >75 deaths per 1000 live births) and the most parsimonious yet best fitting model was chosen based on the AIC.[12] Inclusion of the under-five mortality rates as a categorial resulted in the lowest AIC (AIC=325) whilst the variable on the natural continuous scale or log scale resulted in a poorer fit to the data (AIC = 379, 402 respectively).

As expected, seroconversion after three doses of tOPV was much less likely than the expected equivalent seroconversion rate after mOPV2 administration (Table S3). There was also evidence that seroconversion was far greater in areas with lower child mortality.

Assuming an all-or-nothing response, the effectiveness of a given vaccine (i =tOPV or mOPV2) in a certain country, $z_{C,i}$, can be calculated from the predicted probability of seroconversion in a given country and year, $x_{c,i}$, as:

$$z_{C,i} = 1 - \sqrt[3]{1 - x_{C,i}}$$

The corresponding predicted effectiveness per single dose of each vaccine against type 2 poliovirus for each category of the under-five mortality rate is given in Table S4.

Note that the estimated effectiveness of tOPV in a country with the highest category of under 5 mortality, 50%, aligns closely with the estimated tOPV effectiveness in Nigeria (48%), using a case control approach comparing vaccination histories from VDPV2 cases and controls (non-polio acute flaccid paralysis cases).[13] We use Table S4 to determine the per-dose effectiveness of tOPV and mOPV2 by country using the under-five mortality rate of 2013 as this was the most recent available year of mortality data. 28 of 51 countries on the African continent fell within the highest level of under-five mortality and 19 countries fell within the medium level of under-five mortality.

1.1.2 Crude estimates of population immunity by location and 6-month period

Global surveillance for poliomyelitis is conducted through surveillance for acute flaccid paralysis (AFP) cases.[14] For each AFP case, information recorded includes the first and second administrative region (hereafter province and district, respectively) in which the individual resides, the date of onset of paralysis, the age and sex of the individual, and the reported number of OPV doses received. cVDPV2 poliomyelitis cases are confirmed through isolation and sequencing of poliovirus from two stool samples collected 48 hours apart within 14 days of paralysis onset from notified AFP cases. We used data from AFP cases with onset between 1 January 2015 and 30 June 2020, accessed through the Polio Information System.[15] Institutional ethics approval for this study was not sought because the databases are free of personally identifiable information. The Global Polio Eradication Initiative maintains a calendar of implemented and planned supplementary immunisation activities (SIAs) worldwide.[14] The calendar includes district-level information on the dates of implementation, age groups targeted, and vaccine formulation. We obtained data for SIAs implemented or planned from 1 January 2010 to 30 June 2020.

We estimated population immunity against type 2 poliomyelitis induced by OPV for 51 countries on the African continent at different administrative divisions depending on the number of non-polio AFP cases reported. Immunity was estimated at the national level (administrative level 0) for Botswana, Comoros, Djibouti, Equatorial Guinea, Eritrea, Eswatini, the Gambia, Guinea-Bissau, Lesotho, Namibia, Sao Tome and Principe, and Tunisia, and at the district level (administrative level 2) Nigeria. For all remaining countries, we estimated immunity at the province level (administrative level 1). We estimated population immunity for both children under 60 months and under 36 months for 6-month periods from Jan-Jun 2015 to Jul-Dec 2020 based on the number of doses of OPV reported by non-polio AFP cases, estimates of vaccine efficacy, and the SIA calendar. We define the "crude" estimate of population immunity for a given geography and 6-month period as the mean of the probability of protection for each child with non-polio AFP, weighted to ensure equal representation of each single year age group (since the age distribution of children with AFP differs from the underlying population distribution, which is approximately uniform).

The probability that an individual child is protected against type 2 is obtained using the number of tOPV and mOPV2 doses received, x_t, x_m, respectively, and a per-dose efficacy against type 2, e_t, e_m, as in previously published methods.[13,16,17] The method assumes an all-or-nothing protective response to vaccination where the per-dose probability of inducing an immune response is independent of the number of previous doses. The probability that a child is protected is thus given by $1 - (1 - e_t)^{x_t}(1 - e_m)^{x_m}$. These estimates do not account for immunity resulting from transmission of OPV among contacts of vaccinated children, maternally-derived antibodies, or naturally-acquired immunity following exposure to circulating vaccine-derived type 2 poliovirus (cVDPV2).

The reported number of OPV doses received by a non-polio AFP case includes those received through routine immunisation (RI) and supplementary immunisation activities (SIAs). Only tOPV or bOPV is used in RI (before or after OPV2 withdrawal) whereas SIAs use different formulations of OPV (tOPV, mOPV1, mOPV2, mOPV3, bOPV) at different times depending on the setting, but the reported

number of doses do not specify the vaccine type. To obtain the number of tOPV or mOPV2 doses received through SIAs, the number of reported OPV doses received through SIAs was multiplied by the proportion of SIAs with tOPV or mOPV2 that the child was exposed to based on the SIA calendar (using the child's date of birth and district of residence and the age groups targeted in the SIA). Therefore, the number of tOPV doses that an individual has received, x_r, corresponds to the sum of tOPV doses from RI and tOPV doses from SIAs. The distribution of mOPV2 SIAs between January 2015 and June 2020 is shown in Figure S1.

African countries reporting data to the World Health Organization Eastern Mediterranean Regional Office (EMRO) report the number of OPV doses received through RI and SIAs separately. These countries are Sudan, Egypt, Libya, Tunisia, Morocco, Somalia, and Djibouti. In countries in the African Region (AFRO), only the total number of OPV doses (RI+SIA) is reported. Therefore, for AFRO countries, we estimated routine OPV coverage using district-specific coverage estimates for the first and third doses of Diphtheria-Tetanus-Pertussis (DTP1 and 3) using data from Mosser and colleagues[18] and national-level skilled birth attendance rates from the World Health Organization (WHO) and United Nations International Children's Emergency (UNICEF) Fund Joint Skilled Birth Attendant database.[19] For countries in AFRO administering an additional birth dose of OPV (OPV0), we used the national skilled birth attendance rate scaled by district-level immunisation coverage to reflect geographic heterogeneity in coverage. For each non-polio AFP case, we generated 1000 possible vaccination histories by defining a binary variable indicating whether the child received a birth dose and a multinomial variable indicating whether the child received a further 0, 1, 2, or 3 doses of OPV in RI. The binary variable indicating whether the child received a birth dose was drawn from a Bernoulli distribution with probability of success given by the estimated birth attendance rate in that district. The multinomial variable indicating whether the child received a 0, 1, 2, or 3 doses of OPV was drawn from a categorical distribution with probability of success given by DPT1, 2, and 3 coverage in the district. If the child was born before OPV2 withdrawal, the routine doses were assumed to be tOPV. The remaining doses were assumed to be received though SIAs. Using OPV efficacy estimates and modelled vaccination histories, we therefore generated 1000 estimates of the probability of type 2 immunity per child, which were used to obtain 1000 estimates of immunity for each geography and 6-month period by averaging across all children, weighting by single-year age group. The median of those 1000 estimates were taken as the final crude estimates.

1.1.3 Hierarchical temporal models of population immunity

We then fitted random-effects temporal models to the crude estimates to temporally smooth our estimates. This was important because the number of non-polio AFP cases by geography and 6-month period is relatively small, leading to geographic periods with no crude estimates or crude estimates strongly affected by the presence of outliers.

In the case of Nigeria, let p_{ijt} be the crude estimate of population immunity in a district i in a province j at a 6-month period t, and n_{ijt} the number of non-polio AFP cases used to estimate p_{ijt}. We define the following random-effects model for p_{ijt}:

$$p_{ijt} \sim Normal(\eta_{ijt}, \frac{\sigma^2}{n_{ijt}})$$

$$logit(\eta_{ijt}) = \alpha + \gamma_{it} + \delta_{jt}$$

where α is the intercept, and γ_{it} and δ_{jt} are two space-time interactions at two different spatial levels (the district, γ, and the province, δ). With this formulation, η_{ijt} will therefore be the smoothed estimate of population immunity for the district i in the province j at a 6-month period t.

Having the province-level effect in addition to the district-level effect is important because most of the SIAs are organised at the province level. The district level accounts for difference in vaccine coverage within provinces. The interactions with time are necessary because the number of tOPV and mOPV2 SIAs in each district 6-month period changed over the study period and because changes in coverage are

also likely to have occurred. We assume that both interactions have a structured temporal effect and an unstructured spatial effect. We assume an autoregressive time structure of second order: a second-order random walk across time for each area (district or state) independently of all other areas. If T denotes the total number of time periods, I the total number of districts, and J the total number of states, the densities of the two space-time random effects can be written as

$$\pi(\gamma|\sigma_\gamma^2) \propto exp\left(-\frac{1}{2\sigma_\gamma^2}\sum_{t=3}^{T}\sum_{i=1}^{I}\left(\gamma i,t - 2\gamma_{i,t-1} + \gamma_{i,t-2}\right)^2\right)$$

and

$$\pi(\delta|\sigma_\delta^2) \propto exp\left(-\frac{1}{2\sigma_\delta^2}\sum_{t=3}^{T}\sum_{j=1}^{J}\left(\delta j,t - 2\delta_{j,t-1} + \delta_{j,t-2}\right)^2\right)$$

respectively.

For countries with province-level smoothing and mOPV2 SIAs, we use a province-level space-time interaction term and an additional space-time interaction term that groups together provinces without mOPV2 SIAs (β)

$$logit(\eta_{ijt}) = \alpha + \delta_{jt} + \beta_{jt}$$

In Somalia, we use an additional space-time interaction term that groups together provinces into six federated states (γ). Awdal, Galbeed, Sahil, Togdher, Sanag, and Sool are grouped as Somaliland; Bari and Nugal as Puntland; Mudug and Galgadud as Galmudug; Hiran and Middle Shabelle as Hirshabelle; Banadir as Mogadishu; Bakol, Bay and Lower Shabelle as Koofur Galbeed; and Gedo, Middle Juba, and Lower Juba as Jubaland.

$$logit(\eta_{ijt}) = \alpha + \delta_{jt} + \beta_{jt} + \gamma_{jt}$$

For some countries with limited non-polio AFP data in a few small or sparsely populated provinces (Ethiopia, Chad, Central African Republic, Niger), we combine proximate provinces with low AFP rates (δ'):

$$logit(\eta_{jt}) = \alpha + \delta'_{jt} + \beta_{jt}$$

In Ethiopia, the small provinces of Dire Dawa and Harari are combined with the larger surrounding province of Oromiya. In Chad, the northern desert provinces of Borkou, Ennedi Est, Ennedi Ouest, and Tibesti are combined to form the province of Borkou-Ennedi-Tibesti as it existed prior to 2016. In Central African Republic, the urban interior province of RS7 is combined with the larger surrounding province of RS1. In Niger, the urban interior province of Niamey is combined with the larger surrounding province of Tillabery.

For countries with province-level smoothing and no mOPV2 SIAs, an additional national-level time trend ω_t is modelled as a second-order random walk:

$$logit(\eta_{jt}) = \alpha + \omega_t + \delta_{jt}$$

where

$$\Delta^2\omega_t = \omega_t - 2\omega_{t+1} + \omega_{t+2} \sim Normal(0, \sigma_\omega^2)$$

For some countries with limited non-polio AFP data in a few provinces (Egypt, Mali, Algeria, Morocco), we combine proximate provinces with very low AFP rates (δ'):

$$logit(\eta_{jt}) = \alpha + \omega_t + \delta'_{jt}$$

In Egypt, the provinces of Northern Sinai, Southern Sinai, Ismailia, Port Said, Suez, and Sharkia are combined to form the Suez Canal regional unit (http://gopp.gov.eg/eg-map/). In Mali, the provinces of Gao and Menaka are combined and the provinces of Taoudeni and Tombouctou are combined to form the provinces as they existed prior to 2019. In Morocco, the southern desert provinces of Dakhla-Ou. Eddahab, Guelmim-Oued Noun, and Laayoune-S. Hamra are combined. In Algeria, the south-eastern desert provinces of Ilisii, Tamanghasset, Ouargla, El Oued, Ghardaia, Laghouat, and Biskra, and the south-western desert provinces of Tindouf, Adrar, Bechar, El Bayadh, and Naama are combined to form the health regions (regions sanitaires) of Sud-Est and Sud-Ouest.

In Madagascar, we use an additional space-time interaction term that groups together modern provinces into their previous divisions prior to 2008 (γ).

$$logit(\eta_{jt}) = \alpha + \omega_t + \delta_{jt} + \gamma_{jt}$$

For countries with very limited non-polio AFP data (Botswana, Comoros, Djibouti, Equatorial Guinea, Eritrea, Eswatini, the Gambia, Guinea-Bissau, Lesotho, Namibia, Sao Tome and Principe, and Tunisia), we use only a national-level term:

$$logit(\eta_t) = \alpha + \omega_t$$

None of these countries have had any mOPV2 SIAs, so the assumption of uniform decline in type 2 population immunity following OPV2 withdrawal is not unreasonable. The crude and smoothed time series are provided in Figures S30-S103. The median smoothed estimates for January 2015 to June 2020 are mapped in Figures S5 and S6.

The Normal approximation is used to model population immunity because it is unknown if non-polio AFP cases are immune, and instead only the probability that each case is immune can be derived based on their reported vaccination history and vaccine efficacy. This allows uncertainty in the crude estimates to be scaled to the number of non-polio AFP cases informing those estimates. A similar approach has previously been used.[17] A caveat to this approximation is that this does not hold for very low numbers of non-polio AFP cases and in these instances the model may slight overestimate low values of true immunity and underestimate high values of true immunity. The models were fitted to the data using the Integrated Nested Laplace Approximation (INLA) approach, implemented in the R-INLA package.[20] The INLA algorithm is a computation efficient method for Bayesian inference with latent Gaussian models, providing accurate approximations of the posterior distributions in lower computational time than Markov Chain Monte Carlo methods.

1.1.4 Accounting for IPV

It is not possible to accurately estimate population immunity from IPV delivered in routine immunisation or SIAs directly from non-polio AFP data because AFP cases in AFRO and EMRO do not widely report doses of IPV. We use a cohort model with a one month time step to estimate population immunity from IPV, assuming a per-dose efficacy of IPV against type 2 of 60% ($e_I = 0.6$), based on data from Grassly 2014.[21] The efficacy for a second dose of IPV is likely to be higher, but we did not account for this difference here.

1.1.4.1 IPV in routine immunisation

We approximate subnational routine coverage of IPV in a given district or province j by scaling the annual WHO and UNICEF Estimates of National Immunization Coverage (WUENIC) estimate of national IPV coverage[22] (Figure S4) in a given year y (W_y) by a spatial heterogeneity term derived from local DTP3 coverage estimates (D_j):

$$c_{j,y} = W_y \frac{D_j}{\frac{1}{J}\sum_{j=1}^{J} D_j}$$

We also account for the timing of introduction into national routine immunisation programs (m_{intro}), periods in which routine IPV immunisation was halted due to supply shortages ($M_{disrupt}$), and catch-up campaigns following reintroduction ($m_{catch-up}$, $A_{catch-up}$), where m refers to months and a refers to monthly age cohorts (Figure S3). The coverage of catch-up campaigns is assumed to be the same as routine coverage for the year that the campaign is implemented. This gives the proportion of individuals in month m of age a receiving a routine dose of IPV ($c_{j,m,a}$).

$$c_{j,m,a} = \begin{cases} c_{j,y} & a = 0, m \geq m_{intro}, m \notin M_{disrupt} \\ c_{j,y} & a \in A_{catch-up}, m = m_{catch-up} \\ 0 & else \end{cases}$$

Population immunity from routine IPV immunisation is calculated as

$$R_{j,m,a} = c_{j,m,a} e_I$$

1.1.4.2 IPV in SIAs

From 2014 onwards, a few districts in Nigeria, Cameroon, Kenya, and Somalia have performed SIA campaigns with IPV (Figure S2). Overall immunity from IPV is calculated using population immunity from routine IPV immunisation and the coverage of IPV SIA campaigns

$$I_{j,m,a} = f_2(1-(1-R_{j,m,a})(1-ce_I)^2) + f_1(1-(1-R_{j,m,a})(1-ce_I)) + (1-(f_1+f_2))R_{j,m,a}$$

where f_1 is the proportion of the population in the district or province j targeted with 1 round of IPV in month m and f_2 is the proportion of the population targeted with 2 rounds, e_I is the per-dose efficacy of IPV against type 2, and c is the SIA coverage. There was insufficient data to estimate coverage for IPV SIAs, so we assumed a per-SIA coverage of 60%.

We then take the average value of population immunity from IPV across all ages to obtain the monthly population immunity from IPV.

$$I_{j,m} = \frac{1}{A} \sum_{a=1}^{a=A} I_{j,m,a}$$

The resulting immunity estimates are shown in Figures S7 and S8.

1.1.5 Mucosal and humoral population immunity

We assume that mucosal immunity (M) comes from OPV only:

$$M_{j,m} = O_{j,m}$$

Humoral immunity is induced by either IPV or OPV. We calculate two versions of humoral population immunity (H,H'). The first assumes random distribution of immunity from IPV and OPV, such that children may receive immunity from only IPV or only OPV:

$$H_{j,m} = 1 - (1 - O_{j,m})(1 - I_{j,m})$$

The second assumes assortative distribution of immunity from IPV and OPV, such that only children who receive OPV also receive IPV:

$$H'_{j,m} = maximum(O_{j,m}, I_{j,m})$$

Mucosal and humoral immunity estimates are shown in Figures S9 and S10. We use assortative humoral immunity estimates to validate our results against seroprevalence surveys (Table S5, Figure S11).

1.2 Spatial regression analysis

1.2.1 Model structure

Provinces or districts i in each country j were classified as either reporting or not reporting circulating VDPV2 cases at time t, shown in Figure S13. The log odds or logit probability of reporting cases was assumed to be a function of covariates X and country- and province-level time-invariant random effects, μ:

$$Y_{ijt} \sim Bernoulli(p_{ijt})$$

$$logit(p_{ijt}) = \alpha + \sum_{k=1}^{k=I} \beta_k X_k(i,j,t) + \mu_{ij} + \mu_j$$

$$\mu_{ij} \sim Normal(0, \sigma_{ij}^2)$$

$$\mu_j \sim Normal(0, \sigma_j^2)$$

Because we wanted to identify risk factors for spread of cVDPV2 separate from emergence of cVDPV2, Y_{ijt} excludes the first case in an emergence chain, hereafter referred to as an "emergence". The models were fitted to the data using the Integrated Nested Laplace Approximation (INLA) approach, implemented in the R-INLA package.

1.2.2 Covariates

We considered the following covariates in constructing our final regression model: population immunity from OPV in children under three and children under five years old (Figures S5 and S6), population immunity from IPV in children under three and children under five years old (Figures S7 and S8), effective number of mOPV2 SIAs (Figure S1), force of infection from other districts and provinces (Figures S14-S23), detection of cVDPV2 cases (Figure S13), detection of a new cVDPV2 emergence (Figure S13), average household size (Figure S12A), proportion of population living in urban areas (Figure S12B), prevalence of diarrheal disease (Figure S12C), access to improved sanitation (Figure S12D), population size (Figure S12E), and population density. Population immunity, number of mOPV2 SIAs, force of infection and detection of cases and new cVDPV2 emergences were variable over time and we used the value for the previous six month period. Further details about all the covariates are summarised in Table S1.

The force of infection captures transmission to district or province j from all other districts or provinces. We used the radiation model to approximate population movement across the African continent. The radiation model predicts the commuting flux $R_{i,j}$ from district or province i to district or province j and is dependent on the population sizes P_i and P_j and the population within a circle of radius equal to the Euclidean distance between the two populations $S_{i,j}$.[23] The number of individuals moving from i to j is defined by:

$$R_{i,j} = C_i \frac{P_i P_j}{(P_i + S_{i,j})(P_i + S_{i,j} + P_j)}$$

where C_i is the number of individuals moving out of i. Estimating C_i is not possible in this context. Assuming $C_i = 1$, $R_{i,j}$ represents the probability of movement between districts or provinces i and j. The population size of each geographical area was calculated by summing over a 1 kilometer raster of under-five population, modelled by Tatem and colleagues.[24] The median population size for all provinces outside Nigeria is 131,000 (IQR 50,300-263,000). The median population size for Nigerian districts is 36,800 (IQR 25,000-54,000) whereas the median population size for Nigerian provinces is 750,000 (IQR 603,000-1,110,000).

We calculated a modified version of the radiation model using only population in urban areas, P':

$$R'_{i,j} = \frac{P'_i P'_j}{(P'_i + S'_{i,j})(P'_i + S'_{i,j} + P'_j)}$$

P' was calculated as UP, where U is the proportion of population living in urban areas (Figure S12B), accessed from the Subnational Human Development Database.[25]

We adapted the equations above to decrease the probability of movement across international borders as follows:

$$R_{i,j} = \frac{nP_i P_j}{(P_i + S_{i,j})(P_i + S_{i,j} + P_j)}$$

where $n = 1$ if districts i and j are in the same country, and $n = m$ otherwise. We calculate modified radiation matrices using values for m of 0.01, 0.25, 0.5, and 0.75, reducing cross-border transmission by 99%, 75%, 50%, and 25%. The radiation matrix R was then normalised and used to calculate the force of infection F on district i from all other districts using the presence or absence of cases C at time t :

$$F_{i,t} = \sum_{j,j \neq i} C_{j,t} R_{i,j}$$

Each variant of the force of infection is shown in Figures S14-S23.

We also included newly detected VDPV emergences in the current period as a covariate to adjust for the circulation of novel cVDPV2 strains, which would not be captured in the external force of infection or previous local cases (shown in Figure S13).

Because some SIAs only targeted particular age groups or a single district in a province, we calculated the effective number of rounds of IPV, mOPV2 or tOPV SIAs in a six-month period in province i with N SIAs across K districts as

$$\Theta_{t,i} = \frac{\sum_{n=1}^{N} \sum_{k=1}^{K} a_n P_k}{P_i}$$

where P_k is the population size of district k, a_n is the proportion of children under five years of age targeted in the nth SIA, and P_i is the population size of province i.

The local prevalence of diarrheal disease was calculated using a raster of modelled prevalence of diarrheal disease smoothed to a 5 by 5 kilometer resolutionn (shown in Figure S12C).[26] The model uses covariates (access to road, aridity, ratio of children dependents to working adults, distance from rivers or lakes, night-time lights, diphtheria-tetanus-pertussis immunisation coverage, elevation, number of children under 5 per woman of childbearing age, urbanicity, number of people whose daily vitamin A needs could be met, irrigation, prevalence of under-5 stunting or wasting, and population) associated with diarrheal disease, measured by surveys at a limited subset of locations, to predict prevalence of diarrheal disease across wider geographies. The mean prevalence at province or district level was extracted from the raster in R, weighting for population distribution.

The local prevalence of access to improved sanitation was calculated using a raster of modelled prevalence of access smoothed to a 5 by 5 kilometer resolution (shown in Figure S12D).[27] The model uses covariates (travel time to nearest settlement, aridity, elevation, urbanicity, length of growing season, irrigation, and time) associated with access to sanitation, measured by household surveys at a limited subset of locations, to predict access across wider geographies. The mean prevalence at province (or district level in Nigeria) was extracted from the raster in R, weighting by population.

1.2.3 Model selection

We constructed our final multivariable model by stepwise forward selection. We started with all possible models with a single fixed-effects covariate, including random effects of province i and country j *a priori*:

$$logit(p_{ijt}) = \alpha + \beta_1 X_1(i,j,t) + \mu_{ij} + \mu_j$$

and selected the model with the lowest Watanabe-Akaike information criterion (WAIC).[28] We then fitted all possible models with the first covariate and one additional covariate, selecting the model with the lowest WAIC. We continued this process until the WAIC could not be decreased by more than two by adding an additional covariate. Where the candidate covariate was of the same category as one already present in the reference model (OPV or IPV immunity, force of infection), it was allowed to replace the reference variable. At the end of forward selection, we again tested alternative versions of population immunity and force of infection variables, swapping immunity in the under five population for immunity in the under three population and testing each of the ten variants of the external force of infection. Details of the model selection process are given in Table S2. Our final model included seven fixed effects:

$$\begin{aligned} logit(p_{i,j,t}) = \alpha + \beta_1 O_{i,j,t-1} + \beta_2 I_{i,j,t-1} + \beta_3 M_{i,j,t-1} + \beta_4 F^{75\%}_{i,j,t-1} \\ + \beta_5 E_{i,j,t-1} + \beta_6 D_{i,j} + \beta_7 P_{i,j} + \mu_{i,j} + \mu_j \end{aligned} \tag{1}$$

Table S1: Covariates considered in model selection. OPV = oral poliovirus vaccine; IPV = inactivated poliovirus vaccine; POLIS = Polio Information System; mOPV2 = monovalent type 2 oral poliovirus vaccine; cVDPV2 = circulating type 2 vaccine-derived poliovirus; SHDD = Subnational Human Development Database.

Covariate	Time period	Age group	Variable	Source
Immunity from OPV	Previous six months	Under 3-year-olds; under 5-year-olds	O'; O	Estimated from POLIS data[15]
Immunity from IPV	Previous six months	Under 3-year-olds; under 5-year-olds	I'; I	Estimated from POLIS data[15]
Number of mOPV2 SIAs	Previous six months	Under 5-year-olds	M	Calculated from POLIS data[15]
External force of infection	Previous six months	Under 5-year-olds	F	Calculated from POLIS data,[15] WorldPop,[24] SHDD[25]
cVDPV2 emergences	Previous six months	Under 15-year-olds	E	Retrieved from POLIS[15]
cVDPV2 cases	Previous six months	Under 15-year-olds	C	Retrieved from POLIS[15]
Diarrheal disease (prevalence)	Estimated 2016	Under 5-year-olds	D	Reiner 2020[26]
Access to improved sanitation (prevalence)	Estimated 2016	Under 5-year-olds	K	Deshpande 2020[27]
Average household size	Interpolated 2016		H	SHDD[25]
Proportion of population living in urban area	Interpolated 2016		U	SHDD[25]
Population size	Estimated 2019	Under 5-year-olds	P	WorldPop[24]

Table S2: Summary of model selection process. WAIC = Watanabe Akaike Information Criterion; OPV = oral poliovirus vaccine; IPV = inactivated poliovirus vaccine; mOPV2 = monovalent type 2 oral poliovirus vaccine; FOI = force of infection.

WAIC	Fixed-effects variables	Notes
1229.0	$\beta_1 O_{i,j,t-1}$	Start with OPV immunity in under five year olds
1171.0	$\beta_1 O_{i,j,t-1} + \beta_2 M_{i,j,t-1}$	Add mOPV2 SIAs
1105.6	$\beta_1 O_{i,j,t-1} + \beta_2 M_{i,j,t-1} + \beta_3 F^{75\%}_{i,j,t-1}$	Add FOI (75% reduction)
1094.0	$\beta_1 O_{i,j,t-1} + \beta_2 M_{i,j,t-1} + \beta_3 F^{75\%}_{i,j,t-1} + \beta_4 E_{i,j,t-1}$	Add emergences
1088.6	$\beta_1 O_{i,j,t-1} + \beta_2 M_{i,j,t-1} + \beta_3 F^{75\%}_{i,j,t-1} + \beta_4 E_{i,j,t-1} + \beta_5 P_{i,j}$	Add population size
1083.9	$\beta_1 O_{i,j,t-1} + \beta_2 M_{i,j,t-1} + \beta_3 F^{75\%}_{i,j,t-1} + \beta_4 E_{i,j,t-1} + \beta_5 P_{i,j} + \beta_6 I_{i,j,t-1}$	Add IPV immunity in under five year olds
1078.8	$\beta_1 O_{i,j,t-1} + \beta_2 M_{i,j,t-1} + \beta_3 F^{75\%}_{i,j,t-1} + \beta_4 E_{i,j,t-1} + \beta_5 P_{i,j} + \beta_6 I_{i,j,t-1} + \beta_7 D_{i,j}$	Add diarrhea prevalence

1.2.4 Sensitivity to model priors

We used penalized-complexity (PC) priors for the precision of the Gaussian random effects for country (τ) and province (μ). The PC prior for the precision τ has density

$$\pi(\tau) = \frac{\lambda}{2}\tau^{-3/2} \exp\left(-\lambda\tau^{-1/2}\right), \quad \tau > 0$$

for $\lambda > 0$ where

$$\lambda = -\frac{\ln(\alpha)}{u}$$

and (u, α) are the parameters to this prior. The interpretation of (u, α) is that

$$\text{Prob}(\sigma > u) = \alpha, \qquad u > 0, \quad 0 < \alpha < 1,$$

where the standard deviation is $\sigma = 1/\sqrt{\tau}$.[29]

We used $u = 0.5$ and $\alpha = 0.1$. We varied the value of u to assess the sensitivity of the model to prior specifications for $u \in 1, 0.5, 0.1, 0.01$. The influence of this on the fixed effects estimates in shown in Figure S24.

1.2.5 Non-linear effects

We also tested whether the variables in the final model had a non-linear relationship with the log-odds of cVDPV2 spread. We fitted a model using a categorical variable for the number of mOPV2 rounds, independently estimating the impact of one, two, or three or more rounds of mOPV2 relative to zero rounds (Table S10). We also tested categorical variables for the level of OPV population immunity (20-40%, 40-60%, 60-80%, 80-100%, compared to 0-20%, Table S9), the level of IPV population immunity (15-30%, 30-45%, 45-60%, 60-75%, compared to 0-15%, Table S11), and the magnitude of the force of infection (2nd, 3rd, 4th, 5th quantile compared to 1st, Table S8). We compared the WAIC of these models to the best-fitting model without categorical variables.

1.2.6 Sensitivity to administrative unit

We tested whether the choice of district as the population unit for Nigeria made a significant difference to our findings by fitting the same model in Equation 1 to a data set with population-weighted provincial averages for Nigeria, rather than district-level observations. We found that the fixed effects were largely the same (Table S7).

1.2.7 Sensitivity and specificity

In order to assess the sensitivity and specificity of our model in predicting the observed outcomes between 1 January 2016 and 30 June 2020 and to make projections for the period 1 July to 31 December 2020 we set a threshold to classify the median posterior probabilities $p_{i,j,t}$ (Equation 1) as true positives, true negatives, false positives, or false negatives.

We selected a threshold q_{80} which yielded a sensitivity of greater than 80% . $q_{80} \approx 0.0707$ yielded a sensitivity of 80.1% and a specificity of 97.4% .

We used the posterior probability distributions to create risk tiers: locations in which the full 95% CrI of the probability exceeded a given threshold were classified as high risk; those in which the median and the 2.5th percentile exceeded the threshold were classified as medium risk; those in which only the 2.5th percentile of the credible interval exceeded the threshold were classified as low risk, and those in which the 95% CrI was less than the threshold were classified as very low risk. In other words, locations classified as high risk are expected to exceed the threshold with greater than 97.5% probability; locations classified as medium risk are expected exceed the threshold with greater than 50% probability but less than 97.5% probability; locations classified as low risk are expected exceed the threshold with less than 50% probability but greater than 2.5% probability; locations classified as very low risk are expected exceed the threshold with less than 2.5% probability.

1.2.8 Expected cumulative probability of cVDPV2 detection

We also used the individual median probability estimates, $p_{i,j,t}$, to calculate the expected number of districts or provinces reporting cVDPV2 cases across the entire continent. We first calculated the probability E of exactly $k = 1, 2, 3, ..., 1489$ districts or provinces reporting cVDPV2 cases, where S is the collection of all subsets of size k of the estimates $p_{i,j,t}$ for t = July-December 2020:

$$E_k = \sum^{S} \prod_{n \in S} p_n \prod_{n \notin S} 1 - p_n$$

E_k can be calculated by expanding the joint probability polynomial:

$$\prod_{i,j=1}^{i,j=1489} 1 - p_{i,j,t} + p_{i,j,t} x$$

where the probability E_k of observing exactly k events is the coefficient of x^k in the expanded polynomial.

We then calculated the cumulative probability of k or fewer events occurring by taking the cumulative sum of E_k. The median expected number of districts or provinces reporting cVDPV2 cases is thus the kth event for which the cumulative probability function E_k exceeds 0.5, with lower and upper confidence intervals calculated as the event for which E_k exceeds 0.025 or 0.975, respectively.

1.2.9 Model cross-validation

To test the predictive ability of the best-fitting multivariable model, we performed 6-month ahead out-of-sample predictions from the period 1 January to 30 June 2019 onward, re-fitting the model each time but keeping the variables selected in the model selection process described in section 1.2.3. The predicted median posterior probability of observing a case in a particular location was compared with the observation of cases in that location and evaluated by calculating the area under the curve (AUC) of the receiver operating characteristic curve. We also calculated the sensitivity and specificity of our out-of-sample predictions by setting a minimum threshold giving a sensitivity of more than 80%, following the same methods described in section 1.2.7. The results of the cross-validation are given in Figures S27 and S28.

1.2.10 Projections for 1 July - 31 December 2020

Using data from the period 1 January 2016 to 30 June 2020 and the model described in section 1.2.3, we estimated the probability of observing 1 or more cVDPV2 cases in each province (or district, in the case of Nigeria) between 1 July and 31 December 2020.

In order to accurately characterise the risk of cVDPV2 detection following 0, 1, 2, or 3 rounds of mOPV2 Jan-Jun 2020, we first approximate immunity from OPV Jan-Jun 2020 if 0 rounds had taken place. We calculate the proportion of individuals with any type 2 immunity Jul-Dec 2019 ($t - 1$) as the proportion of individuals born before the most recent mOPV2 round, assuming uniform age distribution 0 to 60 months:

$$q_{t-1} = \frac{10 - (t - 1 - t_{mOPV2})}{10}$$

such that if, for example, $t_{mOPV2} = t - 2$, $q_{t-1} = 0.9$: individuals above 6 months of age at the end of $t - 1$ have type 2 immunity.

Estimated OPV immunity in $t - 1$ is the weighted sum of the immunity in the vaccinated (O^V) and unvaccinated (O^U) age cohorts:

$$O_{t-1} = O^V_{t-1} q_{t-1} + O^U_{t-1}(1 - q_{t-1})$$

where $O^U = 0$:

$$O_{t-1} = O^V_{t-1} q_{t-1}$$

Thus immunity in the vaccinated age cohort is

$$O^V_{t-1} = \frac{O_{t-1}}{q_{t-1}}$$

In the absence of any further vaccination, we assume that OPV immunity Jan-Jun 2020 (t) is the product of immunity in the vaccinated population at $t-1$ (O^V_{t-1}) and the proportion of the population with immunity at t (q_t):

$$q_t = \frac{10 - (t - t_{mOPV2})}{10}$$

$$O_t = O^V_{t-1} q_t$$

We calculate the new OPV immunity after m rounds of mOPV2, assuming a coverage of c and per-dose efficacy of e:

$$O^*_{m,t} = 1 - (1 - O_t)(1 - ce)^m$$

We then calculate the probability p for all locations i, j for Jul-Dec 2020 ($t+1$) for $m \in \{0, 1, 2, 3\}$:

$$\begin{aligned} logit(p_{i,j,m,t+1}) = \alpha + \beta_1 O^*_{m,i,j,t} + \beta_2 I_{i,j,t} + \beta_3 m + \beta_4 F^{75\%}_{i,j,t} \\ + \beta_5 E_{i,j,t} + \beta_6 D_{i,j} + \beta_7 P_{i,j} + \mu_{i,j} + \mu_j \end{aligned} \tag{2}$$

We performed three sensitivity analyses, assuming 1) lower SIA coverage (50%) 2) a categorical effect of one, two, or three or more mOPV2 rounds in the previous six months, or 3) an additional reduction in international movement Jan-Jun 2020 due to COVID-19 restrictions (changing $F^{75\%}_{i,j,2020}$ to $F^{99\%}_{i,j,2020}$). These results are shown in Tables S12 and S13.

1.2.11 Retrospective cVDPV2 risk for 1 January 2017 - 30 June 2020

Using data from the period 1 January 2016 to 30 June 2020 and the model described in section 1.2.3, we estimated the probability of observing 1 or more cVDPV2 cases in each province (or district, in the case of Nigeria) between 1 July and 31 December 2020 for two scenarios: first, assuming the number of mOPV2 SIAs reported, and second, a counterfactual assuming no mOPV2 SIAs, adjusting OPV immunity estimates as in section 1.2.10. Results of this analysis are shown in Figures S26 and S29.

2 Supplementary results

Table S3: Multivariable odds ratios of seroconversion against type 2 poliovirus by vaccine formulation and national under-five mortality rate across 38 populations in Africa, Asia and Latin America. 95% CI = 95% profile confidence intervals.

Factor	Odds ratio	95% CI	
Vaccine			
mOPV2	1.00		
tOPV	0.25	(0.11-	0.48)
Under-five mortality rate			
≥ 75	1.00		
25 - 75	4.59	(2.53-	9.36)
< 25	3.08	(2.43 -	3.94)

Table S4: Predicted probability of seroconversion against type 2 poliovirus following three OPV doses as a function of vaccine formulation and national under-five mortality rate, and the corresponding per dose effectiveness estimates assuming an all-or-nothing response.

Vaccine	Under-five mortality rate	Probability of seroconversion after three doses	Per-dose effectiveness
tOPV	< 25	0.97	0.67
	25 - 75	0.96	0.65
	≥ 75	0.88	0.50
mOPV2	< 25	0.99	0.80
	25 - 75	0.99	0.78
	≥ 75	0.97	0.68

Table S5: Seroprevalence against type 2 poliovirus by age, time period, and location. Comparison of model estimates (H' as defined in section 1.1.5) and observed seroprevalence from seroprevalence surveys by age group in months.

Country	Model estimate		Seroprevalence by age		Seroprevalence survey			Seroprevalence by age					
	Location	Date	0-35	0-59	Study	Location	Date	6-9	6-11	12-15	19-22	36-47	36-59
Nigeria	Jere, Borno	Jan-Jun 2016	0.89	0.90	[30]	Borno, primarily Jere and Maiduguri	Jan-Feb 2016	0.86				0.95	
Nigeria	Maiduguri, Borno	Jan-Jun 2016	0.86	0.83									
Nigeria	Damaturu, Yobe	Jan-Jun 2016	0.89	0.88		Yobe, primarily Nguru and Damaturu	Jan-Feb 2016	0.74				0.93	
Nigeria	Nguru, Yobe	Jan-Jun 2016	0.87	0.87									
Guinea	Kankan	Jul-Dec 2016	0.79	0.77	[31]	Kankan and Siguiri	Dec 2016-Jan 2017		0.77			0.97	
Mali	Koulikoro	Jul-Dec 2016	0.72	0.75		Kenieroba and Selingue	Jun-Jul 2016		0.97			1.00	
Cote d'Ivoire	Poro	Jul-Dec 2016	0.85	0.82		Korogho	Nov 2016		0.79			0.98	
Madagascar	Analamanga	Jul-Dec 2016	0.98	0.96	[32]	Antananarivo	May-Sep 2016		0.93				0.98
Madagascar	Boeni	Jul-Dec 2016	0.93	0.91		Mahajanga	May-Sep 2016		0.95				1.00
Madagascar	Melaky	Jul-Dec 2016	0.90	0.91		Antsalova	May-Sep 2016		0.92				1.00
Madagascar	Sud-Est	Jul-Dec 2016	0.91	0.88		Midongy-atsimo	May-Sep 2016		0.75				1.00
Nigeria	Kano Municipal, Kano	Jul-Dec 2015	0.77	0.77	[33]	Kano Metropolitan Area	Oct-Nov 2015	0.83		0.85	0.86		

Table S6: Risk factors associated with the spread of circulating vaccine-derived type 2 poliovirus based on multivariable mixed-effects lagged regression model for January-June 2016 to January-June 2020 using IPV rounds and routine IPV coverage in place of IPV population immunity, WAIC 1082.4. cOR = crude odds ratio; aOR = adjusted odds ratio; 95% CrI = 95% credible interval.

	Univariable			Multivariable		
Variable (fixed effects)	cOR	95% CrI		aOR	95% CrI	
Emergence (previous 6 months)	78.1	(37.2-	165)	18.5	(6.25-	57.3)
Log FOI (previous 6 months, external)	1.57	(1.5-	1.64)	1.51	(1.42-	1.62)
IPV rounds (previous 6 months)	0.669	(0.224-	1.48)	0.609	(0.165-	1.7)
Routine IPV coverage (10% increase)	1.13	(0.617-	2.12)	0.791	(0.17-	4.86)
OPV immunity (previous 6 months, under 5 years, 10% increase)	0.667	(0.629-	0.705)	0.697	(0.616-	0.78)
mOPV2 rounds (previous 6 months)	0.931	(0.69-	1.2)	0.305	(0.2-	0.444)
Diarrhea prevalence (10% increase)	1.74	(1.6-	1.91)	1.67	(1.32-	2.13)
Log population size	2.14	(1.91-	2.39)	1.41	(1.08-	1.85)
Variable (random effects)				Median	95% CrI	
Province (precision)				1.66	(0.812-	4.05)
Country (precision)				0.555	(0.293-	1.08)

Table S7: Risk factors associated with the spread of circulating vaccine-derived type 2 poliovirus based on multivariable mixed-effects lagged regression model for January-June 2016 to January-June 2020, using province-level averages for observations in Nigeria. cOR = crude odds ratio; aOR = adjusted odds ratio; 95% CrI = 95% credible interval.

	Univariable			Multivariable		
Fixed effects variable	cOR	95% CrI		aOR	95% CrI	
Emergence (previous 6 months)	78.1	(37.2-	165)	19.6	(7.00-	58)
Log FOI (previous 6 months, external)	1.57	(1.50-	1.64)	1.50	(1.39-	1.62)
IPV immunity (previous 6 months, under 5 years, 10% increase)	1.06	(0.965-	1.15)	0.925	(0.740-	1.15)
OPV immunity (previous 6 months, under 5 years, 10% increase)	0.667	(0.629-	0.705)	0.672	(0.589-	0.755)
mOPV2 rounds (previous 6 months)	0.931	(0.690-	1.20)	0.385	(0.249-	0.566)
Diarrhea prevalence (10% increase)	1.74	(1.60-	1.91)	1.40	(1.11-	1.78)
Log population size	2.14	(1.91-	2.39)	1.56	(1.20-	2.07)
Variable (random effects)				Median	95% CrI	
Province (precision)				8.08	(1.50-	204)
Country (precision)				0.618	(0.327-	1.19)

Table S8: Risk factors associated with the spread of circulating vaccine-derived type 2 poliovirus based on multivariable mixed-effects lagged regression model for January-June 2016 to January-June 2020 using a categorical variable for the external force of infection (FOI) based on quantiles, WAIC 1096.1. cOR = crude odds ratio; aOR = adjusted odds ratio; 95% CrI = 95% credible interval.

Fixed effects variable	cOR	95% CrI		aOR	95% CrI	
Diarrhea prevalence (under 5 years, 10% increase)	1.74	(1.60-	1.91)	1.69	(1.34-	2.16)
Emergence (previous 6 months)	78.1	(37.2-	165)	12.1	(4.50-	34.3)
Log FOI (previous 6 months, external)						
1st quantile, -23.1 to -14.1	1.00		1.00			
2nd quantile, -14.1 to -12.7	2.71	(0.402-	36.1)	2.53	(0.368-	34.3)
3rd quantile, -12.7 to -10.6	2.71	(0.402-	36.2)	0.920	(0.133-	12.6)
4th quantile, -10.6 to -7.95	18.0	(3.63-	191)	3.67	(0.698-	41.2)
5th quantile, -7.95 to 0.958	161	(33.9-	1640)	38.5	(7.59-	421)
IPV immunity (previous 6 months, under 5 years, 10% increase)	1.06	(0.965-	1.15)	0.850	(0.696-	1.03)
Log population size	2.14	(1.91-	2.39)	1.47	(1.14-	1.93)
mOPV2 rounds (previous 6 months)	0.931	(0.690-	1.20)	0.499	(0.340-	0.698)
OPV immunity (previous 6 months, under 5 years, 10% increase)	0.667	(0.629-	0.705)	0.643	(0.566-	0.722)
Variable (random effects)				Median	95% CrI	
Province (precision)				1.92	(0.907-	5.06)
Country (precision)				0.388	(0.222-	0.685)

Table S9: Risk factors associated with the spread of circulating vaccine-derived type 2 poliovirus based on multivariable mixed-effects lagged regression model for January-June 2016 to January-June 2020 using a categorical variable for type 2 OPV population immunity, WAIC 1089.2. cOR = crude odds ratio; aOR = adjusted odds ratio; 95% CrI = 95% credible interval.

Fixed effects variable	cOR	95% CrI		aOR	95% CrI	
Diarrhea prevalence (under 5 years, 10% increase)	1.74	(1.60-	1.91)	1.54	(1.22-	1.95)
Emergence (previous 6 months)	78.1	(37.2-	165)	17.7	(6.03-	54.0)
IPV immunity (previous 6 months, under 5 years, 10% increase)	1.06	(0.965-	1.15)	0.831	(0.684-	0.998)
Log FOI (previous 6 months, external)	1.57	(1.50-	1.64)	1.54	(1.44-	1.65)
Log population size	2.14	(1.91-	2.39)	1.38	(1.07-	1.82)
mOPV2 rounds (previous 6 months)	0.931	(0.690-	1.20)	0.303	(0.199-	0.440)
OPV immunity (previous 6 months, under 5 years)						
0-20%	1.00			1.00		
20-40%	0.680	(0.482-	0.96)	0.559	(0.335-	0.920)
40-60%	0.271	(0.174-	0.413)	0.216	(0.110-	0.406)
60-80%	0.110	(0.062-	0.182)	0.103	(0.0437-	0.228)
80-100%	0.0365	(0.0187-	0.0646)	0.0756	(0.0276-	0.187)
Variable (random effects)				Median	95% CrI	
Province (precision)				1.72	(0.828-	4.25)
Country (precision)				0.574	(0.307-	1.10)

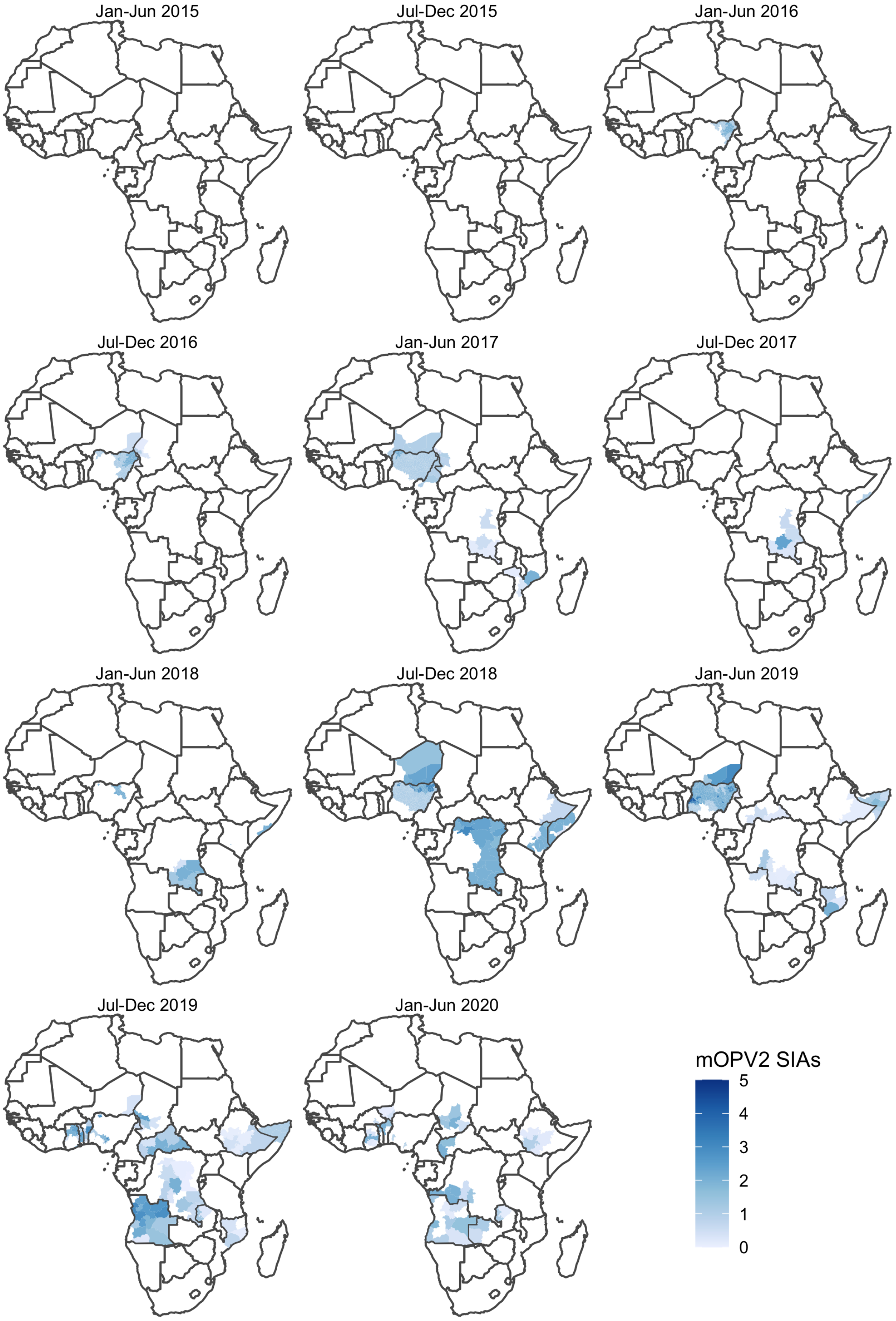

Figure S1: Number of mOPV2 campaigns by six-month period from January 2015 to June 2020, proportion of under-five population targeted per district (Nigeria) or province (all other countries). The publication of this map does not imply the expression of any opinion whatsoever on the part of WHO concerning the legal status of any territory, city or area or of its authorities, or concerning the delimitation of its frontiers or boundaries.

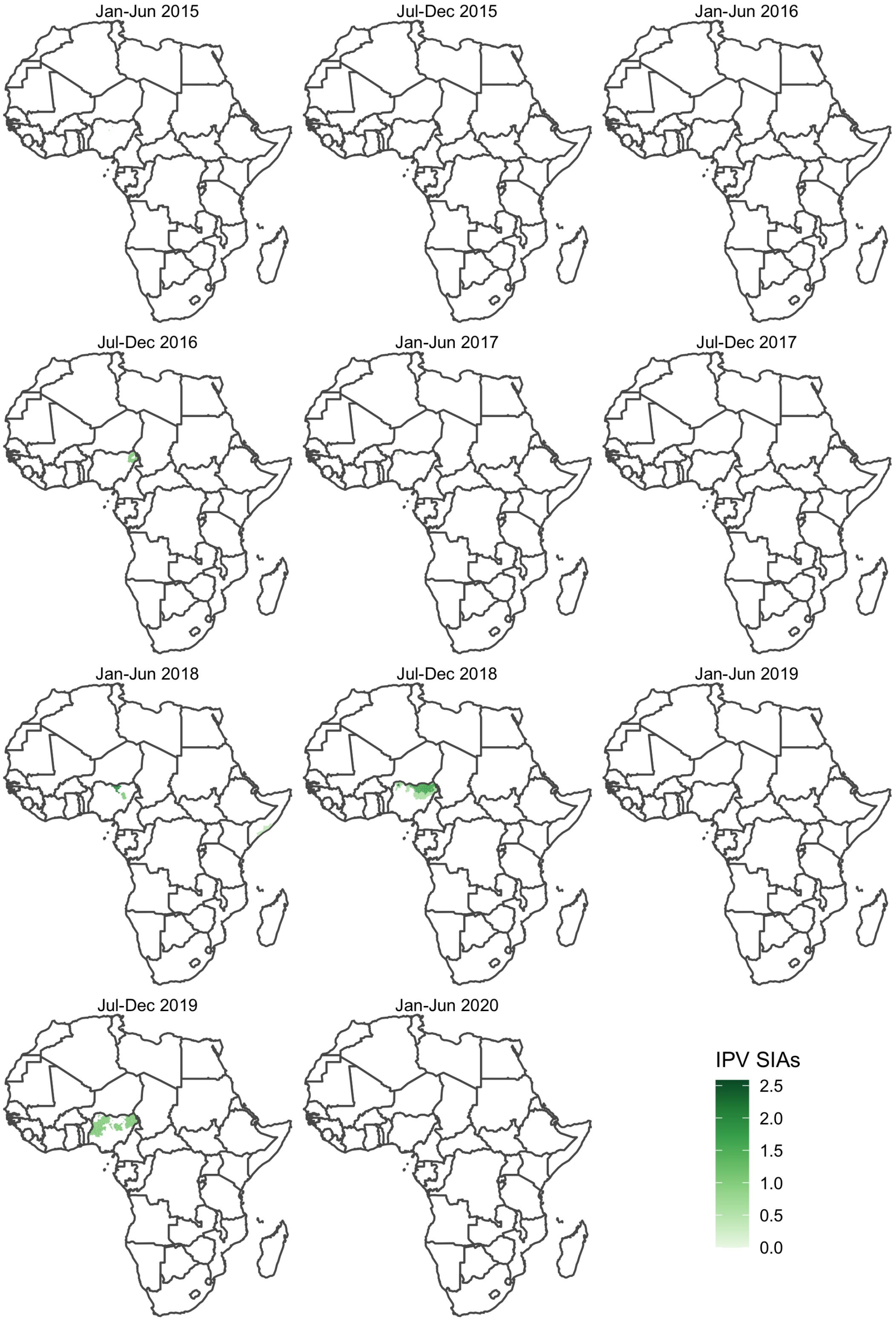

Figure S2: Number of IPV campaigns by six-month period from January 2015 to June 2020, proportion of under-five population targeted per district (Nigeria) or province (all other countries). The publication of this map does not imply the expression of any opinion whatsoever on the part of WHO concerning the legal status of any territory, city or area or of its authorities, or concerning the delimitation of its frontiers or boundaries.

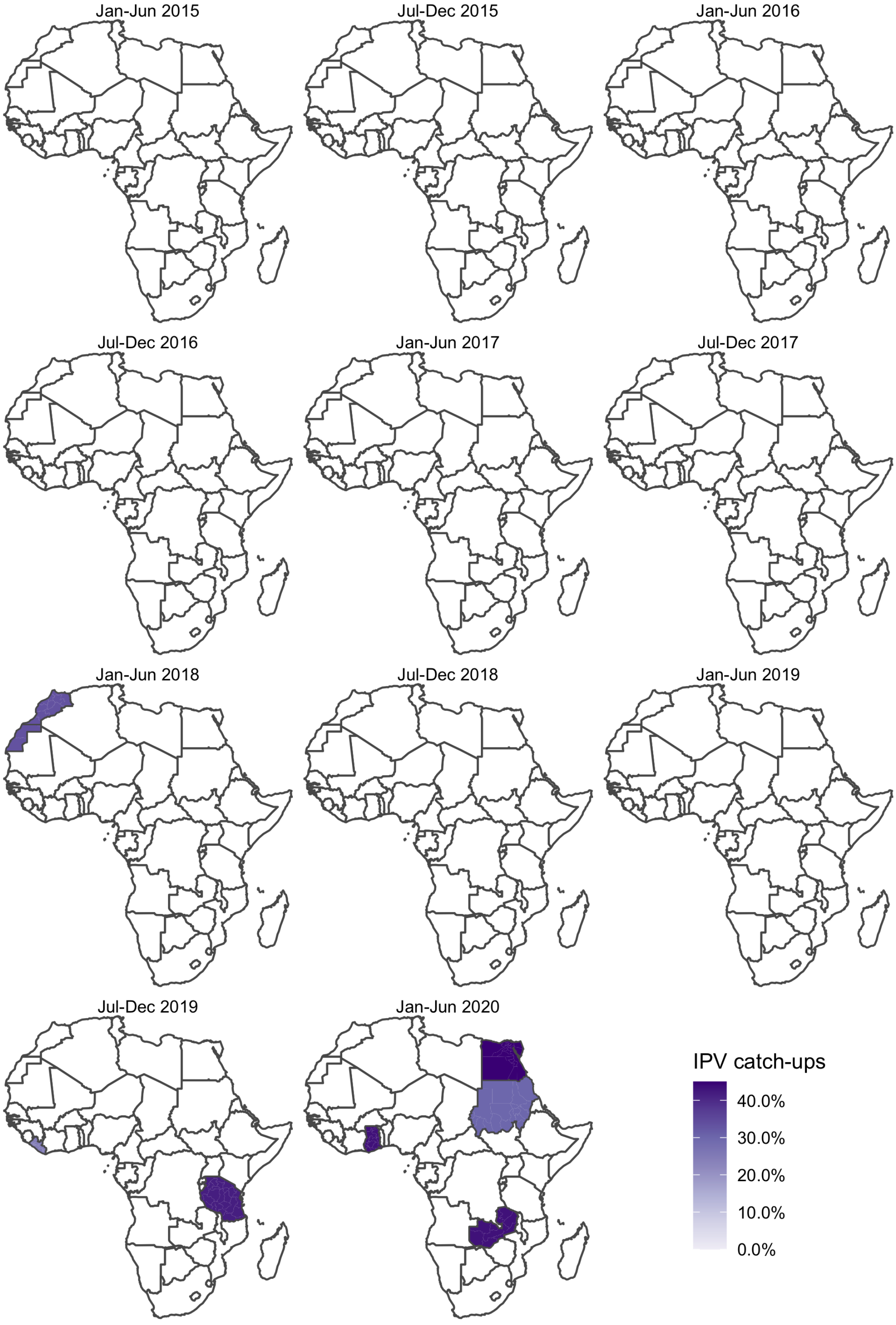

Figure S3: IPV routine immunisation catch-up campaigns by six-month period from January 2015 to June 2020, proportion of under-five population targeted per district (Nigeria) or province (all other countries). The publication of this map does not imply the expression of any opinion whatsoever on the part of WHO concerning the legal status of any territory, city or area or of its authorities, or concerning the delimitation of its frontiers or boundaries.

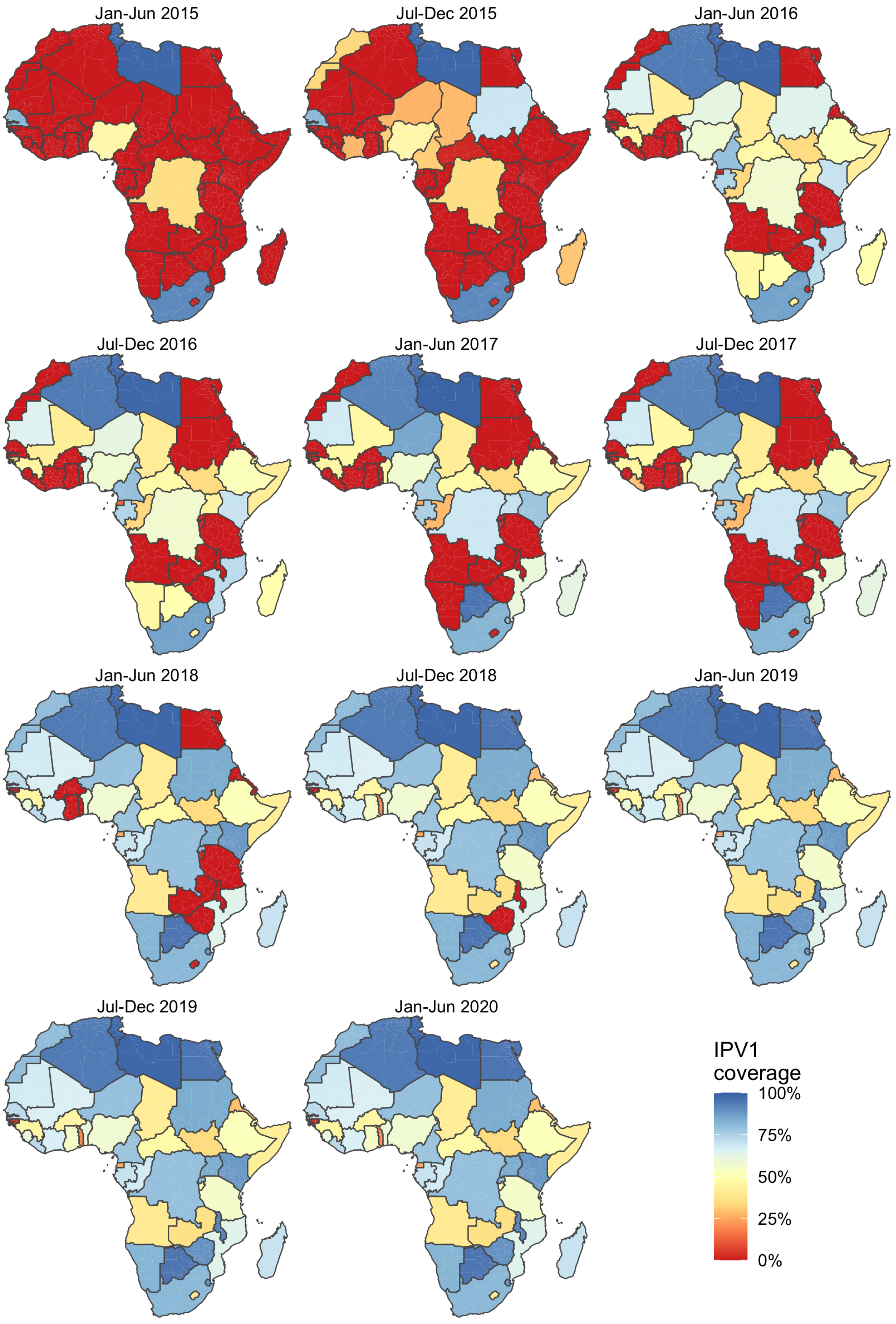

s

Figure S4: Proportion of children receiving one dose IPV in routine immunisation by six-month period from January 2015 to June 2020. The publication of this map does not imply the expression of any opinion whatsoever on the part of WHO concerning the legal status of any territory, city or area or of its authorities, or concerning the delimitation of its frontiers or boundaries.

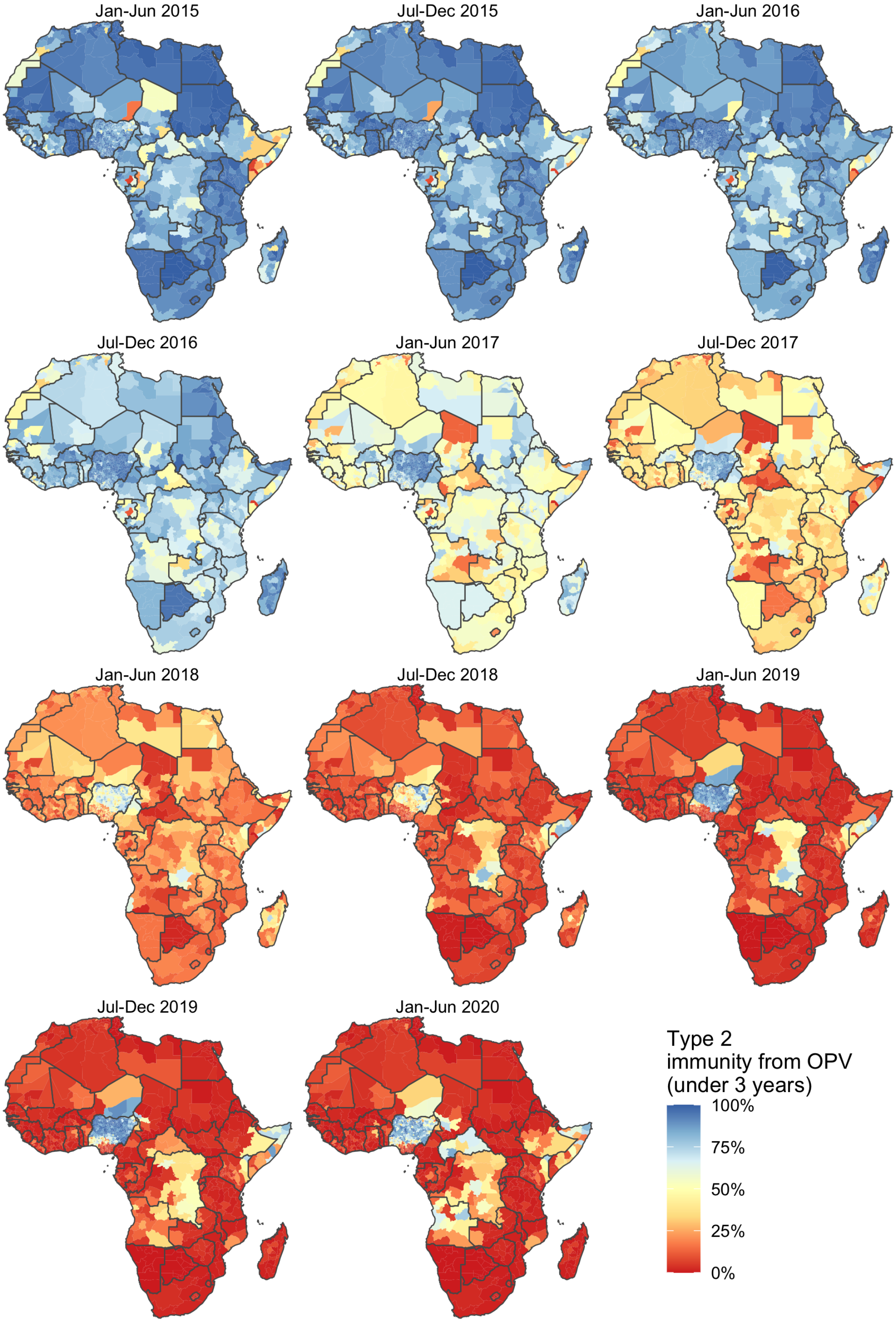

Figure S5: Proportion of under-three population with type 2 immunity from OPV by six-month period from January 2015 to June 2020. The publication of this map does not imply the expression of any opinion whatsoever on the part of WHO concerning the legal status of any territory, city or area or of its authorities, or concerning the delimitation of its frontiers or boundaries.

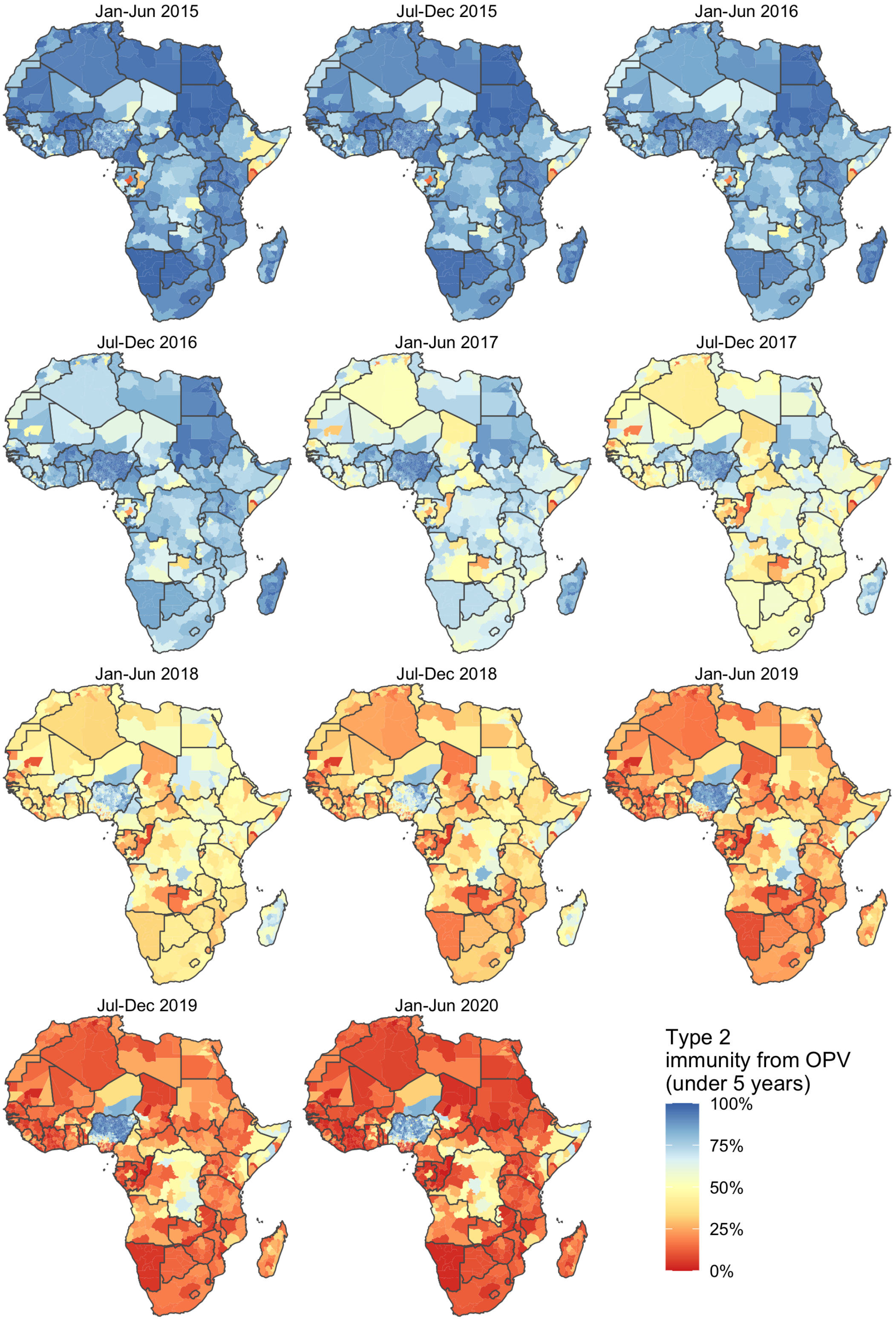

Figure S6: Proportion of under-five population with type 2 immunity from OPV by six-month period from January 2015 to June 2020. The publication of this map does not imply the expression of any opinion whatsoever on the part of WHO concerning the legal status of any territory, city or area or of its authorities, or concerning the delimitation of its frontiers or boundaries.

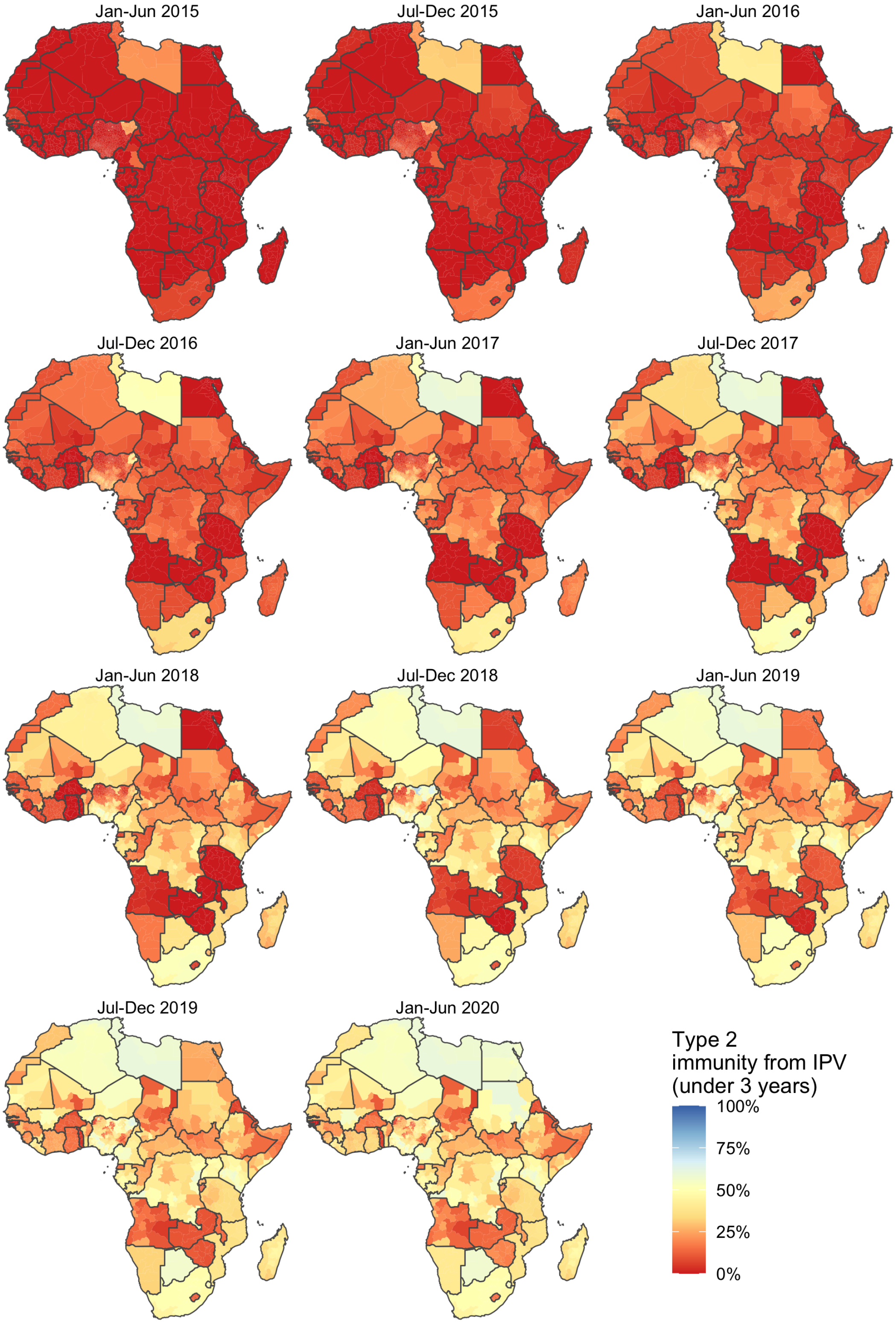

Figure S7: Proportion of under-three population with type 2 immunity from IPV by six-month period from January 2015 to June 2020. The publication of this map does not imply the expression of any opinion whatsoever on the part of WHO concerning the legal status of any territory, city or area or of its authorities, or concerning the delimitation of its frontiers or boundaries.

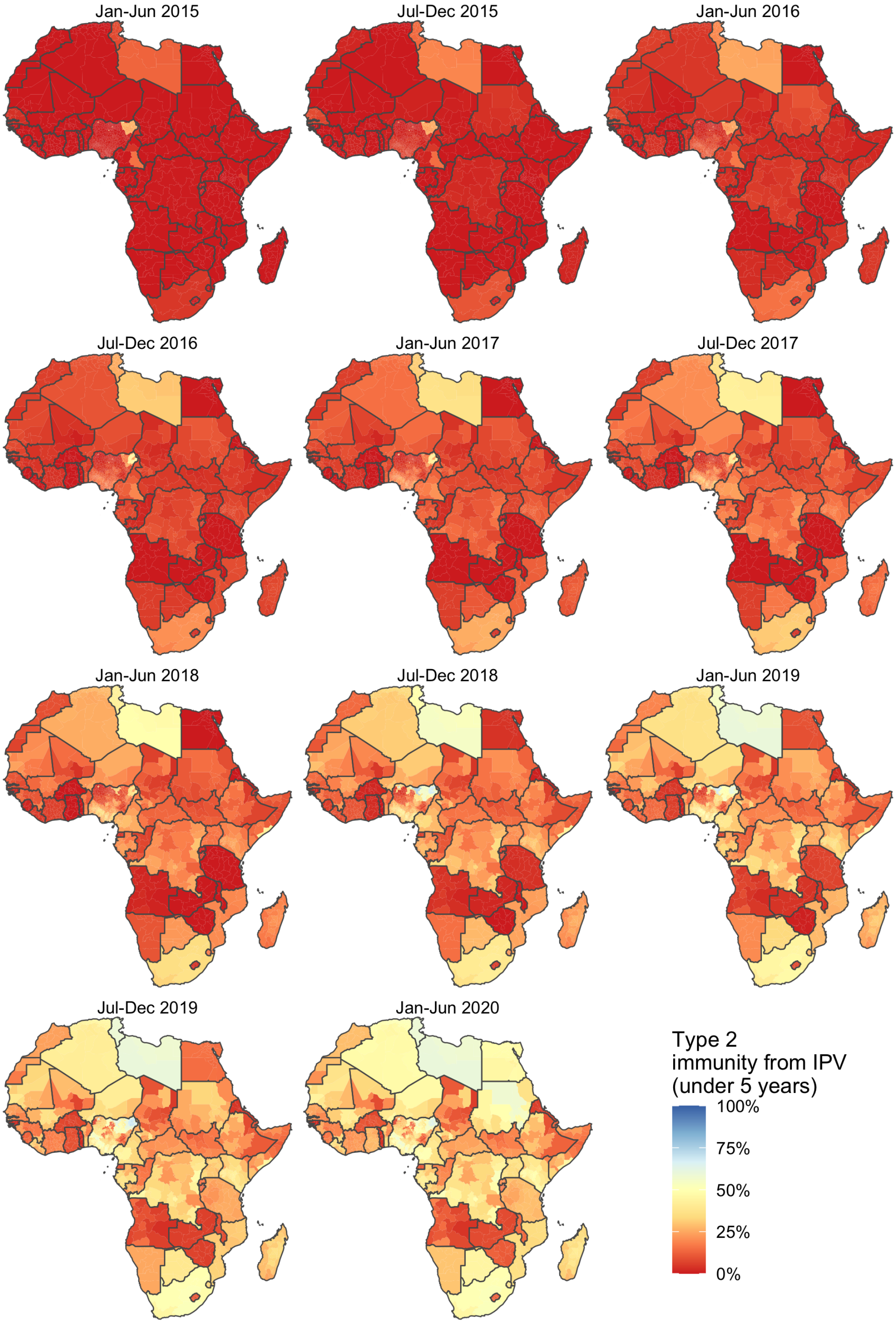

Figure S8: Proportion of under-five population with type 2 immunity from IPV by six-month period from January 2015 to June 2020. The publication of this map does not imply the expression of any opinion whatsoever on the part of WHO concerning the legal status of any territory, city or area or of its authorities, or concerning the delimitation of its frontiers or boundaries.

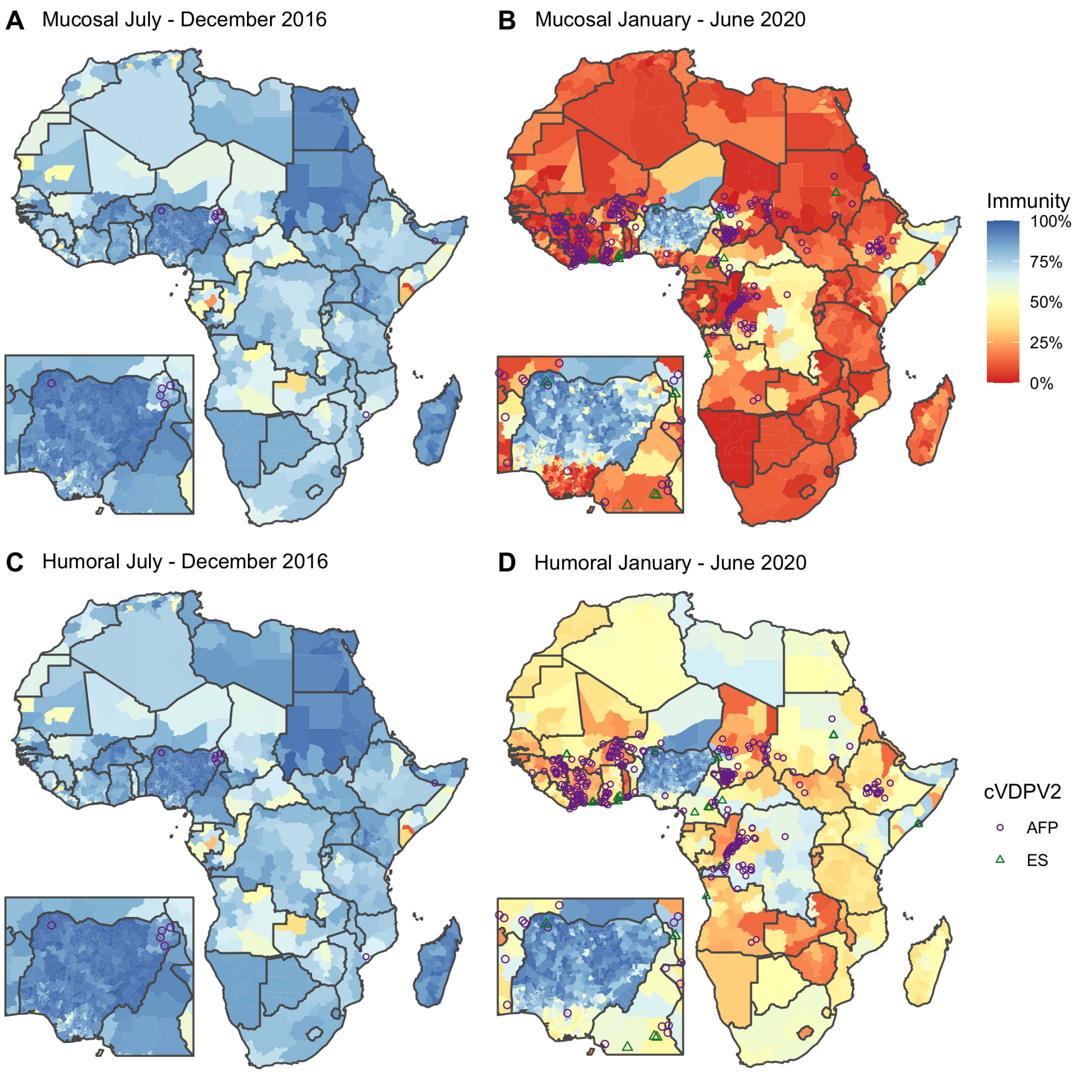

Figure S9: Estimated humoral and mucosal population immunity against type 2 poliomyelitis in children under five years. Estimates are shown for Jul-Dec 2016 (A, C) and Jan-Jun 2020 (B, D) for mucosal immunity (A, B) and humoral immunity (C, D). Cases of circulating vaccine-derived type 2 poliomyelitis (AFP) and detections of circulating vaccine-derived type 2 poliovirus in the environment (ES) shown as purple circles and green triangles. The publication of this map does not imply the expression of any opinion whatsoever on the part of WHO concerning the legal status of any territory, city or area or of its authorities, or concerning the delimitation of its frontiers or boundaries.

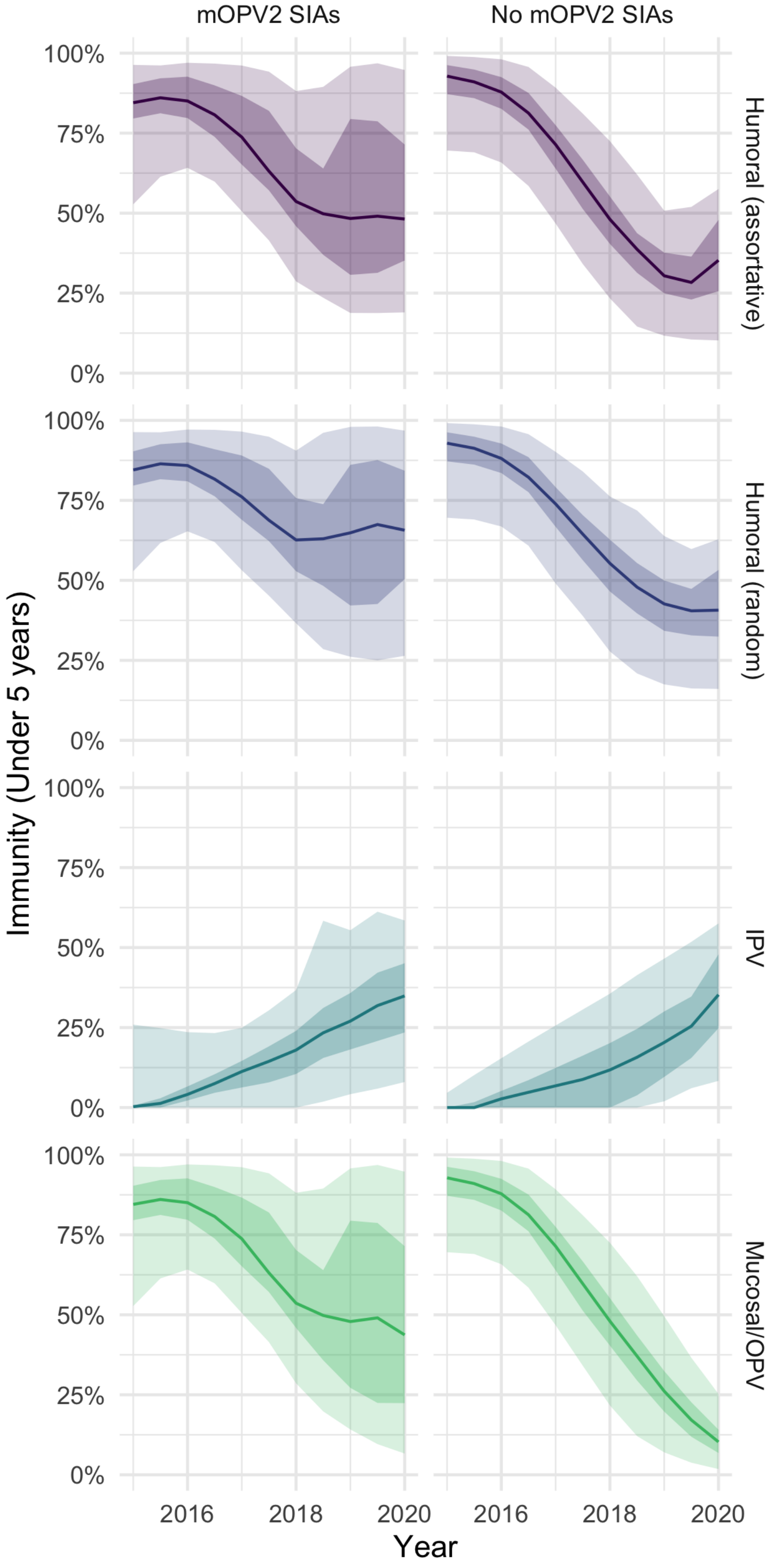

Figure S10: Weighted median population immunity against type 2 poliovirus in provinces with and without monovalent type 2 oral poliovirus vaccine (mOPV2) use at six-month intervals from January 2105 to June 2020. Each panel shows proportion of population with humoral immunity (assuming either assortative or random distribution of IPV and OPV immunity), immunity from IPV, and immunity from OPV. The darker shaded ribbons indicate population-weighted interquartile range and lighter shaded ribbons indicate population.

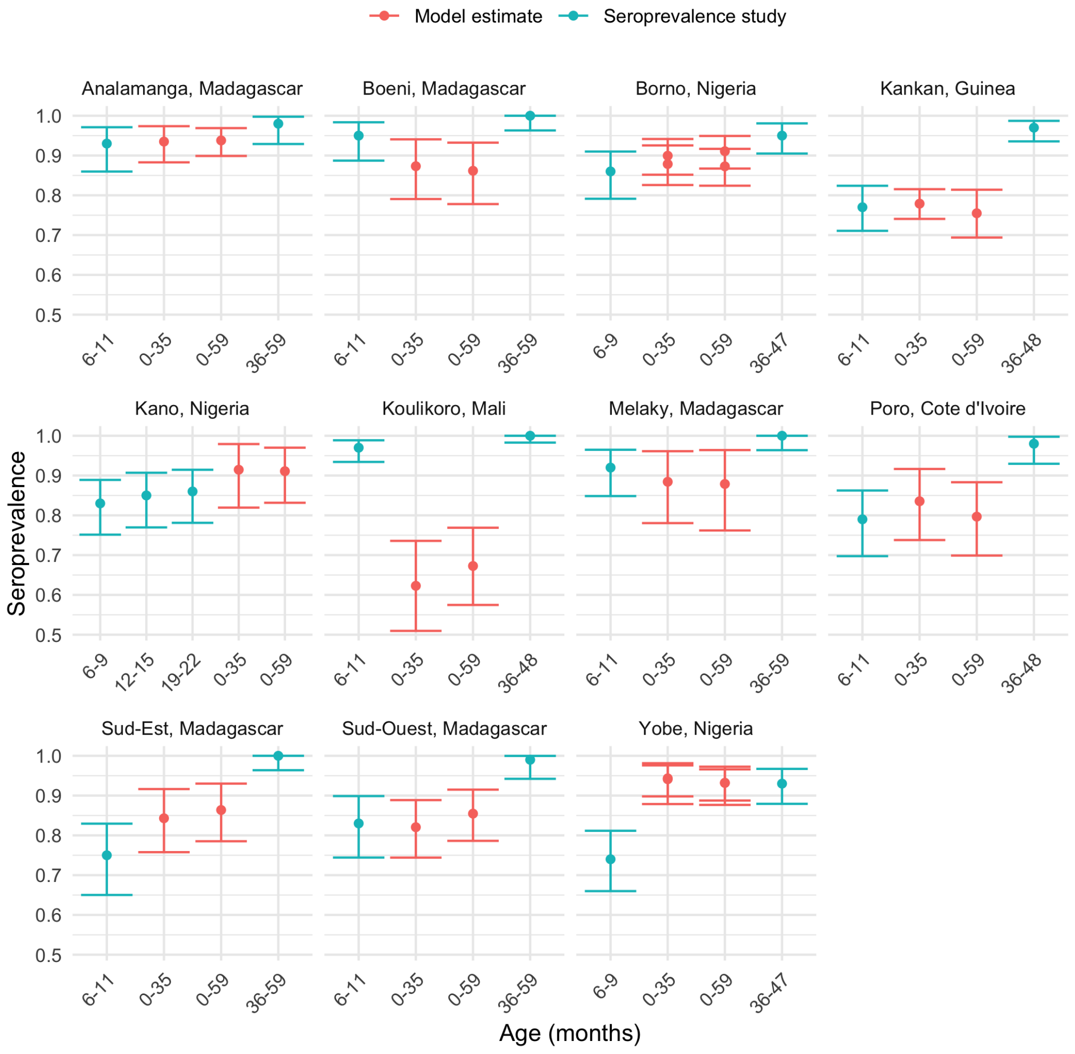

Figure S11: Seroprevalence against type 2 poliovirus by age group and location. Comparison of model-estimated seroprevalence (red, H' as defined in section 1.1.5) and seroprevalence survey results (blue) for 11 locations across 4 seroprevalence studies. Error bars for model estimates come from 95% CrI for OPV immunity and error bars for seroprevalence study observations come from binomial 95% confidence intervals.

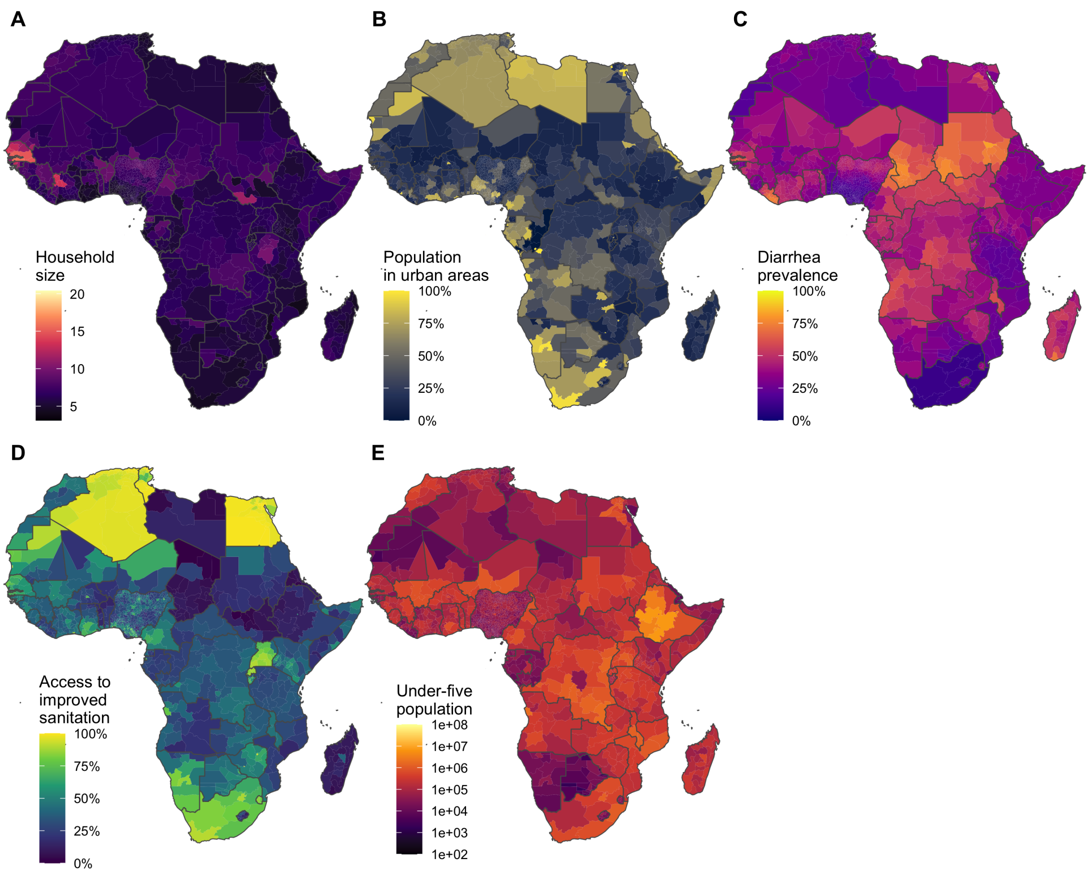

Figure S12: Time-invariant covariates used in model selection. A) Average household size. B) Proportion of population living in urban areas. C) Median estimated prevalence of diarrheal disease in children under five. D) Median estimated prevalence of access to improved sanitation. E) Size of under-five population. The publication of this map does not imply the expression of any opinion whatsoever on the part of WHO concerning the legal status of any territory, city or area or of its authorities, or concerning the delimitation of its frontiers or boundaries.

Table S10: Risk factors associated with the spread of circulating vaccine-derived type 2 poliovirus based on multivariable mixed-effects lagged regression model for January-June 2016 to January-June 2020 using a categorical variable for mOPV2 rounds, WAIC 1074.1. cOR = crude odds ratio; aOR = adjusted odds ratio; 95% CrI = 95% credible interval.

Fixed effects variable	cOR	95% CrI		aOR	95% CrI	
Emergence (previous 6 months)	78.1	(37.2-	165)	25.7	(8.63-	78.4)
Log FOI (previous 6 months, external)	1.57	(1.50-	1.64)	1.53	(1.43-	1.64)
IPV immunity (previous 6 months, under 5 years, 10% increase)	1.06	(0.965-	1.15)	0.809	(0.661-	0.977)
OPV immunity (previous 6 months, under 5 years, 10% increase)	0.667	(0.629-	0.705)	0.668	(0.590-	0.749)
Diarrhea prevalence (10% increase)	1.74	(1.60-	1.91)	1.63	(1.28-	2.08)
Log population size	2.14	(1.91-	2.39)	1.42	(1.09-	1.88)
mOPV2 rounds (previous 6 months)						
Less than 0.5	1.00			1.00		
0.5 to 1.5	0.587	(0.29-	1.05)	0.122	(0.0462-	0.282)
1.5 to 2.5	0.929	(0.422-	1.76)	0.117	(0.0426-	0.281)
2.5 or more	1.34	(0.260-	4.32)	0.0429	(0.00559-	0.204)
Variable (random effects)				Median	CrI	
Province (precision)				1.38	(0.688-	3.06)
Country (precision)				0.460	(0.247-	0.878)

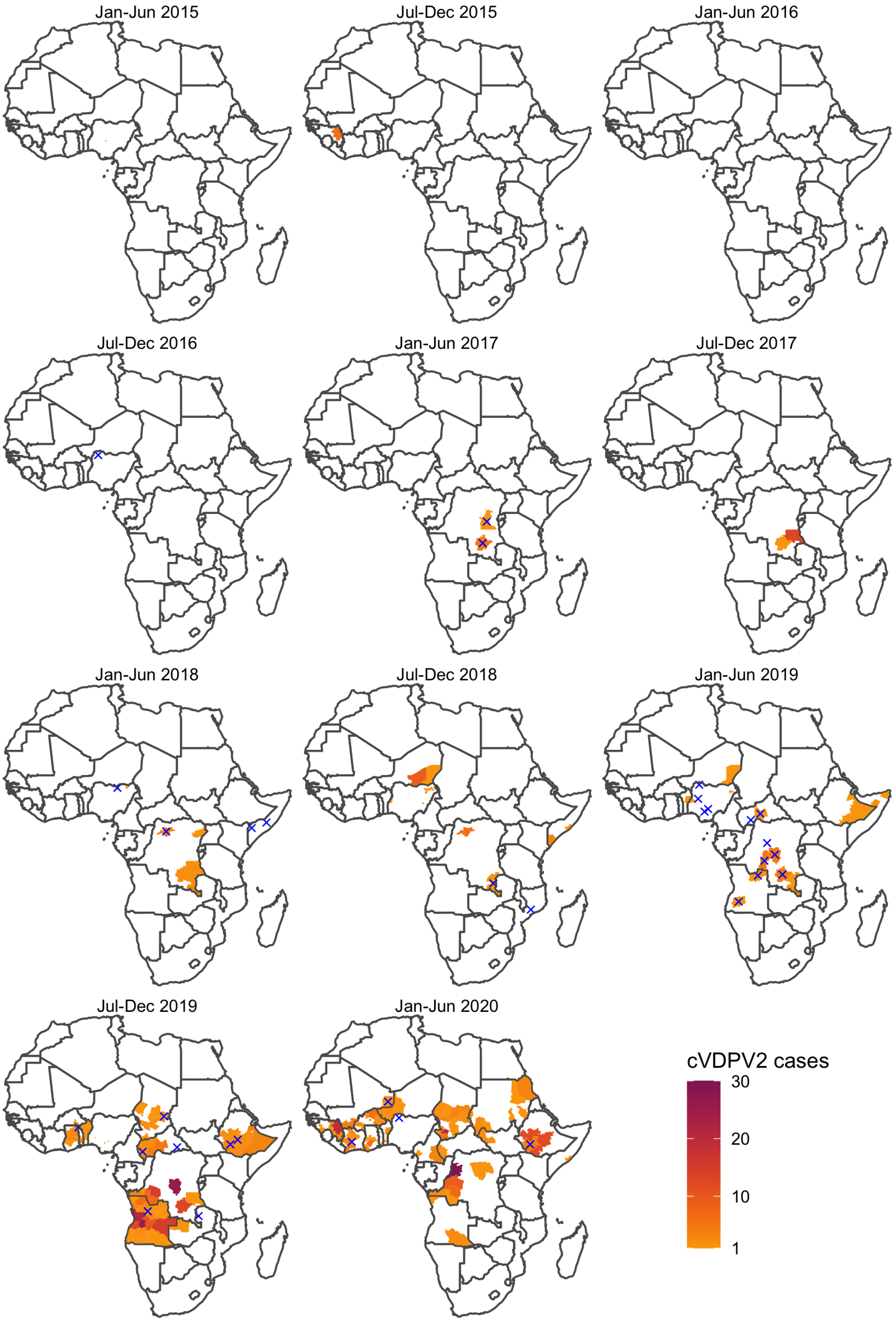

Figure S13: Number of cVDPV2 cases in children under fifteen years of age per six-month period from January 2015 to June 2020. Blue crosses indicate the locations of new cVDPV2 emergence lineages. The publication of this map does not imply the expression of any opinion whatsoever on the part of WHO concerning the legal status of any territory, city or area or of its authorities, or concerning the delimitation of its frontiers or boundaries.

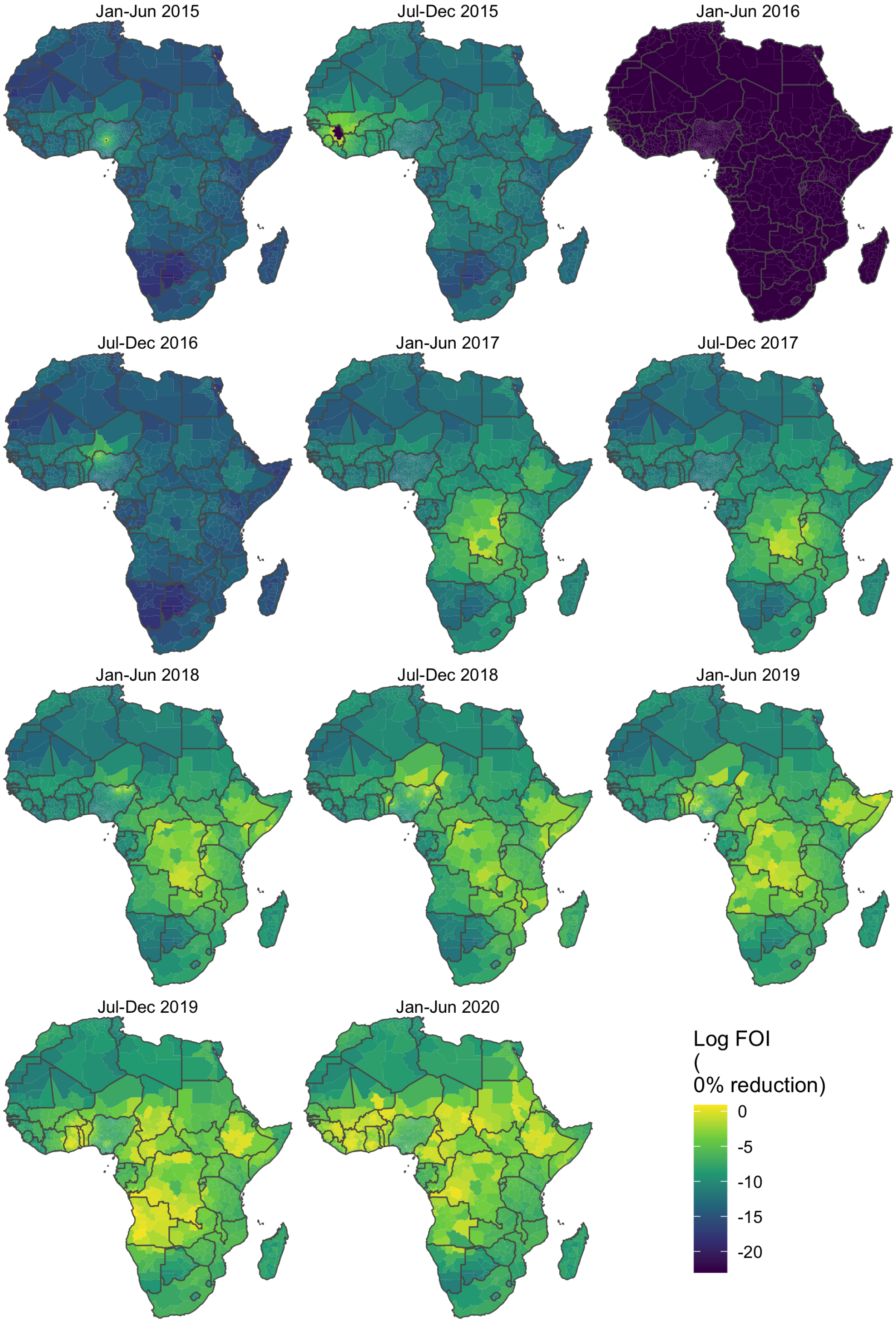

Figure S14: Natural log of the force of infection from external provinces and districts with 0% reduction in movement across international borders. The publication of this map does not imply the expression of any opinion whatsoever on the part of WHO concerning the legal status of any territory, city or area or of its authorities, or concerning the delimitation of its frontiers or boundaries.

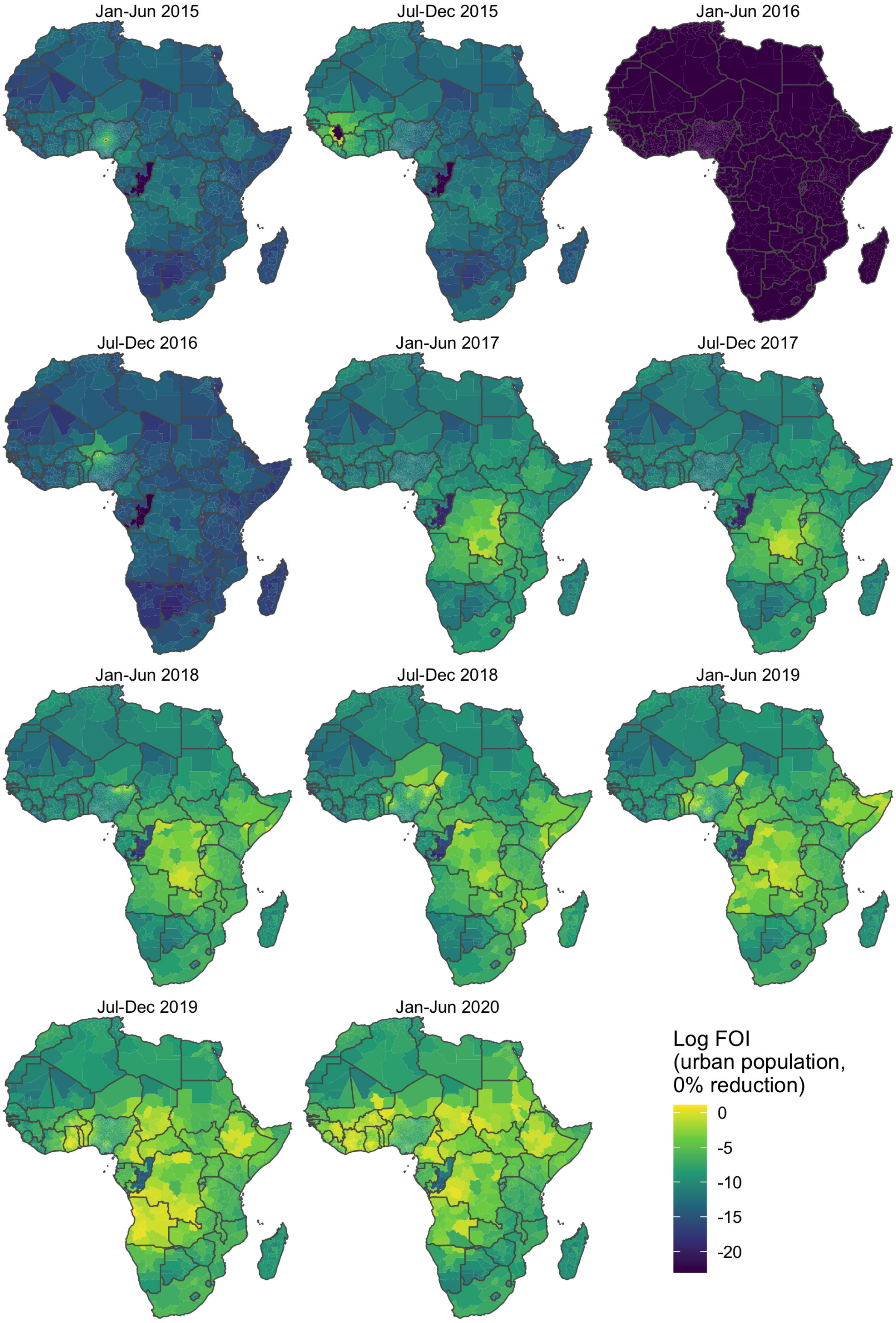

Figure S15: Natural log of the force of infection from external provinces and districts with 0% reduction in movement across international borders using population in urban areas only. The publication of this map does not imply the expression of any opinion whatsoever on the part of WHO concerning the legal status of any territory, city or area or of its authorities, or concerning the delimitation of its frontiers or boundaries.

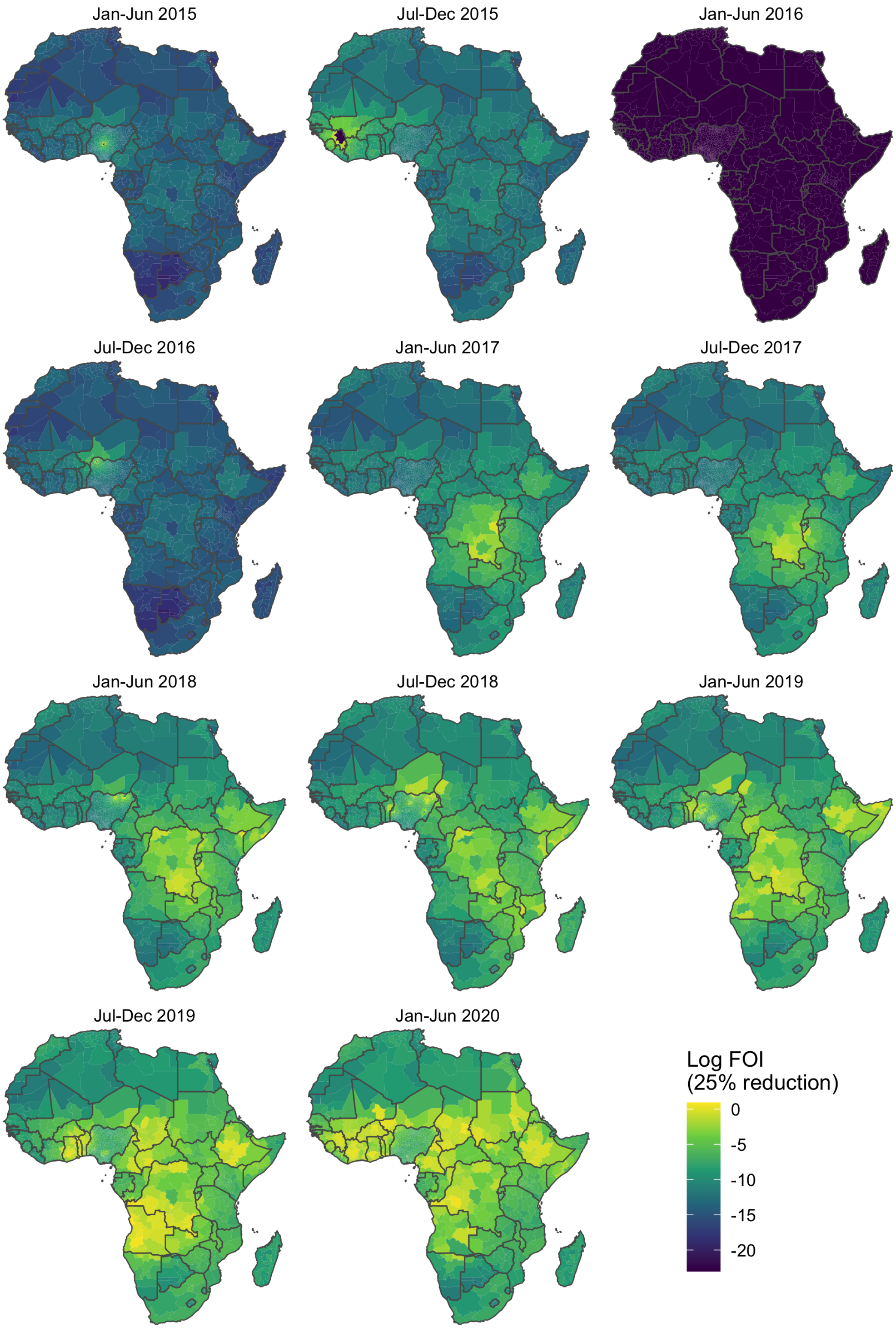

Figure S16: Natural log of the force of infection from external provinces and districts with 25% reduction in movement across international borders. The publication of this map does not imply the expression of any opinion whatsoever on the part of WHO concerning the legal status of any territory, city or area or of its authorities, or concerning the delimitation of its frontiers or boundaries.

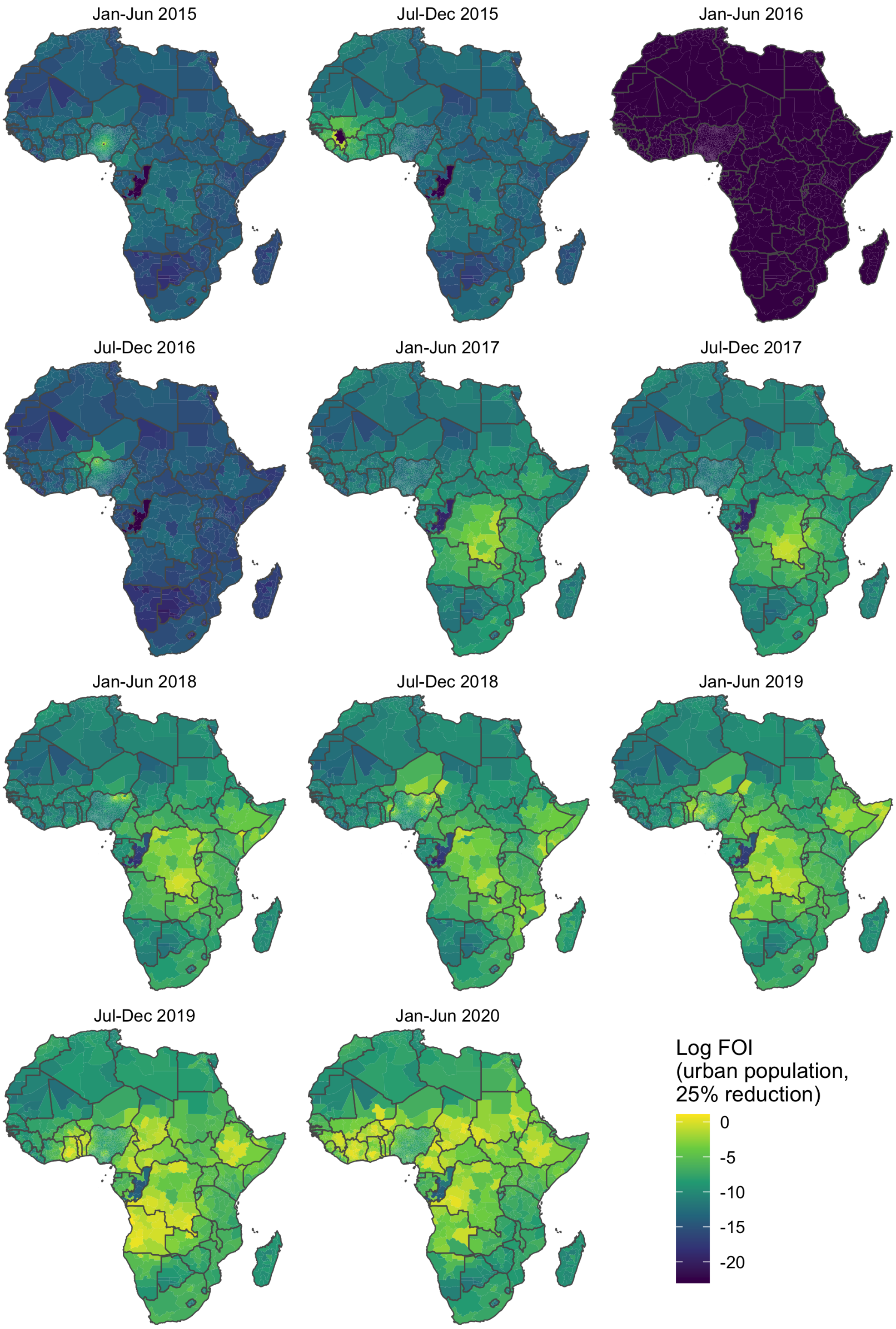

Figure S17: Natural log of the force of infection from external provinces and districts with 25% reduction in movement across international borders using population in urban areas only. The publication of this map does not imply the expression of any opinion whatsoever on the part of WHO concerning the legal status of any territory, city or area or of its authorities, or concerning the delimitation of its frontiers or boundaries.

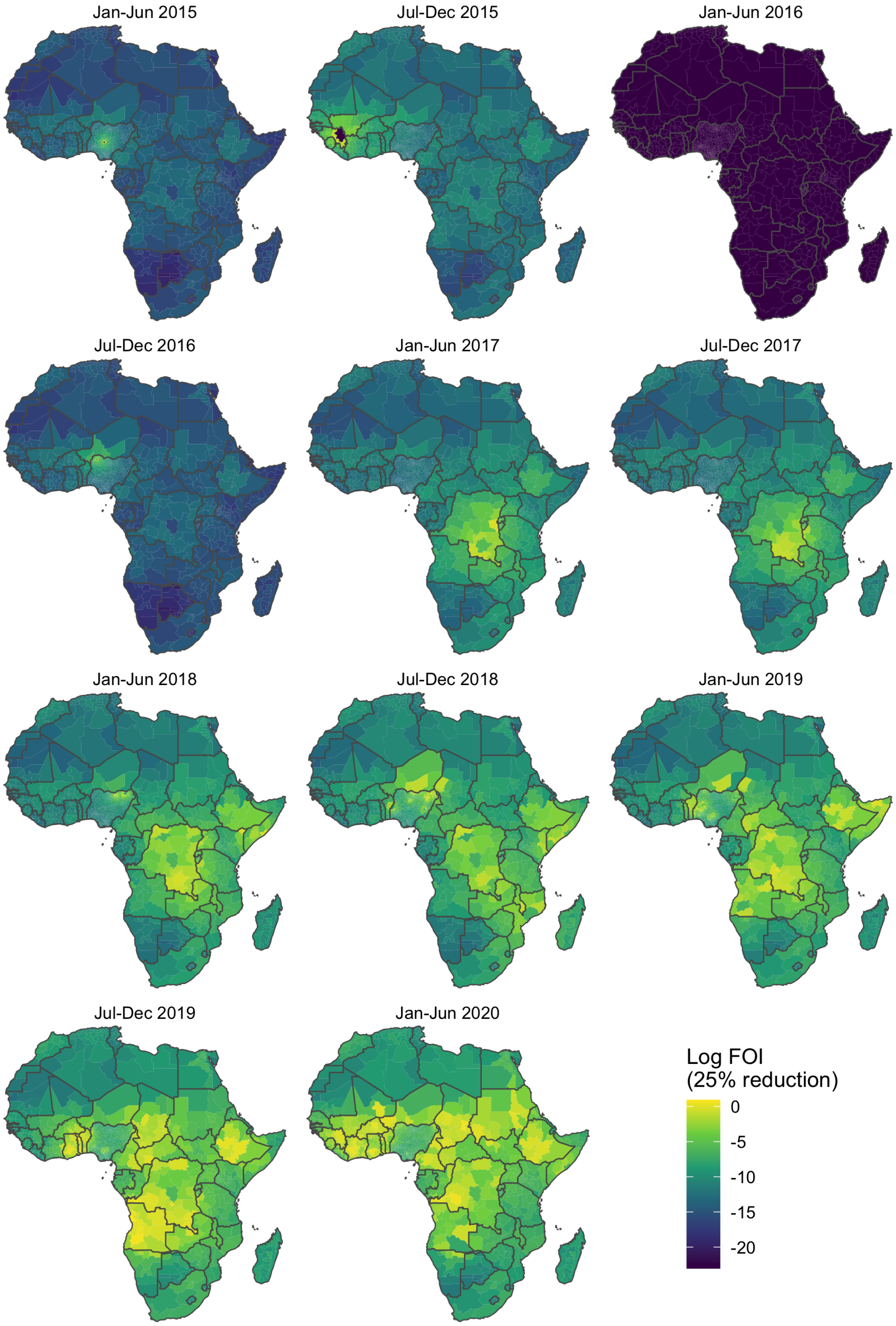

Figure S18: Natural log of the force of infection from external provinces and districts with 50% reduction in movement across international borders. The publication of this map does not imply the expression of any opinion whatsoever on the part of WHO concerning the legal status of any territory, city or area or of its authorities, or concerning the delimitation of its frontiers or boundaries.

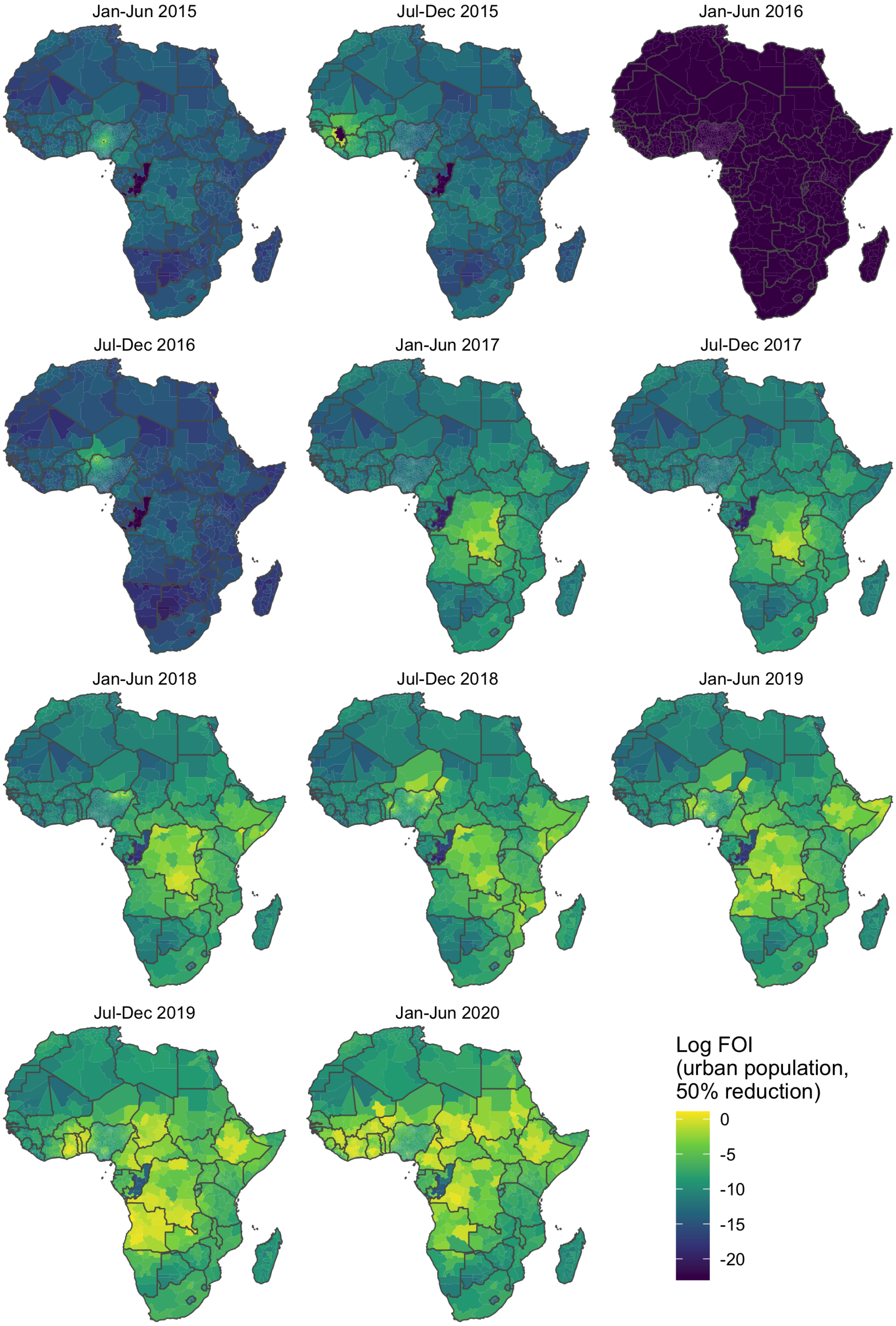

Figure S19: Natural log of the force of infection from external provinces and districts with 50% reduction in movement across international borders using population in urban areas only. The publication of this map does not imply the expression of any opinion whatsoever on the part of WHO concerning the legal status of any territory, city or area or of its authorities, or concerning the delimitation of its frontiers or boundaries.

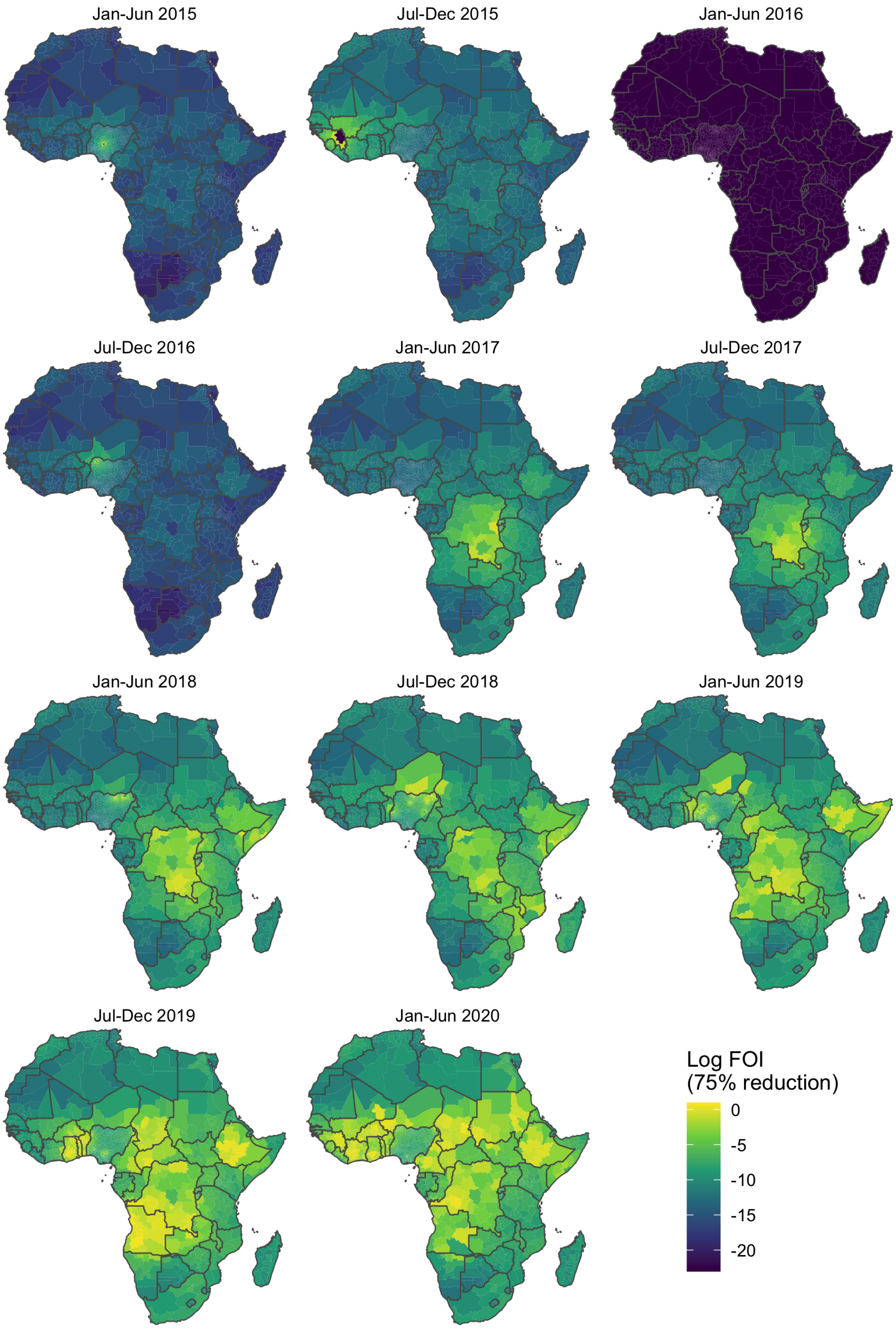

Figure S20: Natural log of the force of infection from external provinces and districts with 75% reduction in movement across international borders. The publication of this map does not imply the expression of any opinion whatsoever on the part of WHO concerning the legal status of any territory, city or area or of its authorities, or concerning the delimitation of its frontiers or boundaries.

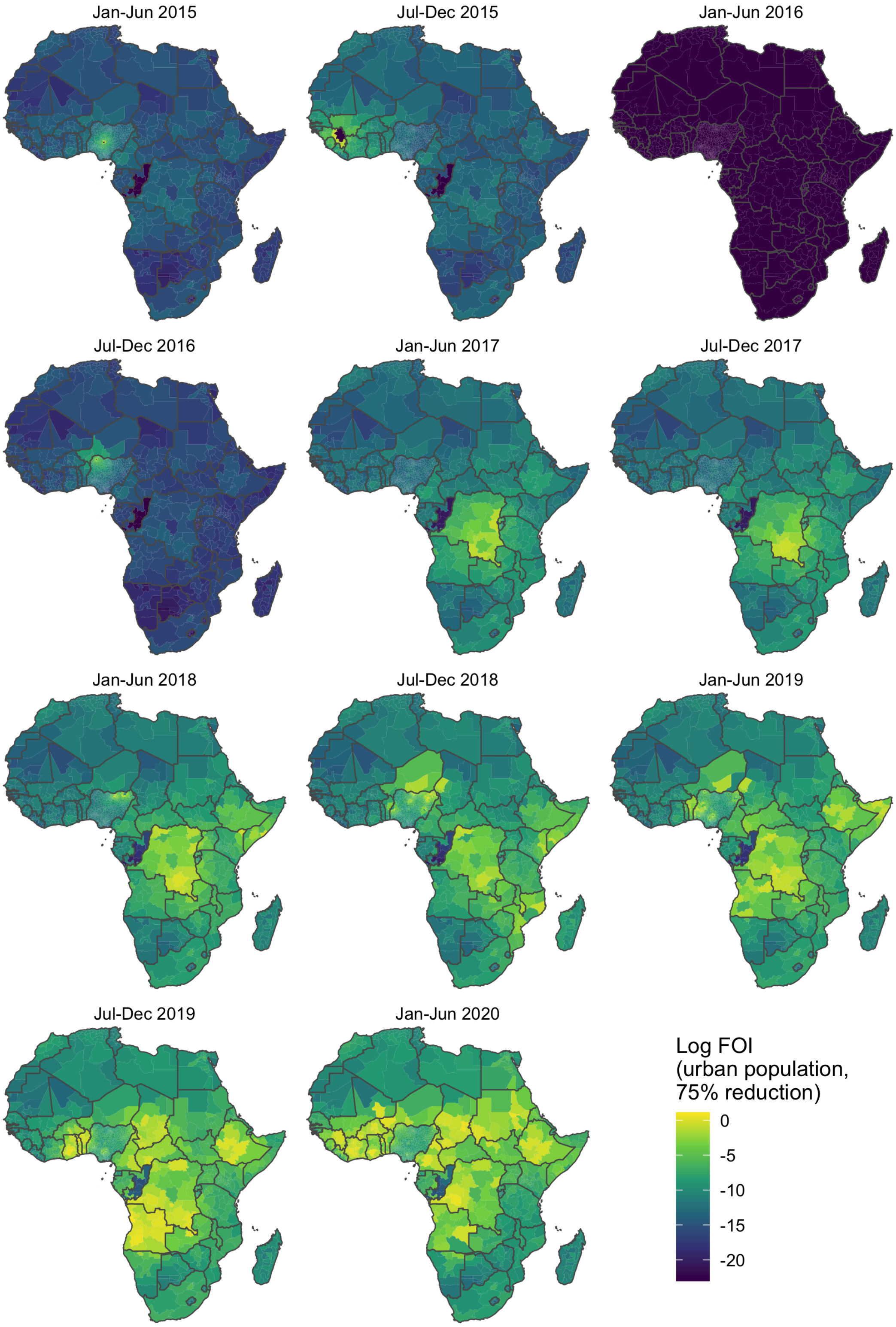

Figure S21: Natural log of the force of infection from external provinces and districts with 75% reduction in movement across international borders using population in urban areas only. The publication of this map does not imply the expression of any opinion whatsoever on the part of WHO concerning the legal status of any territory, city or area or of its authorities, or concerning the delimitation of its frontiers or boundaries.

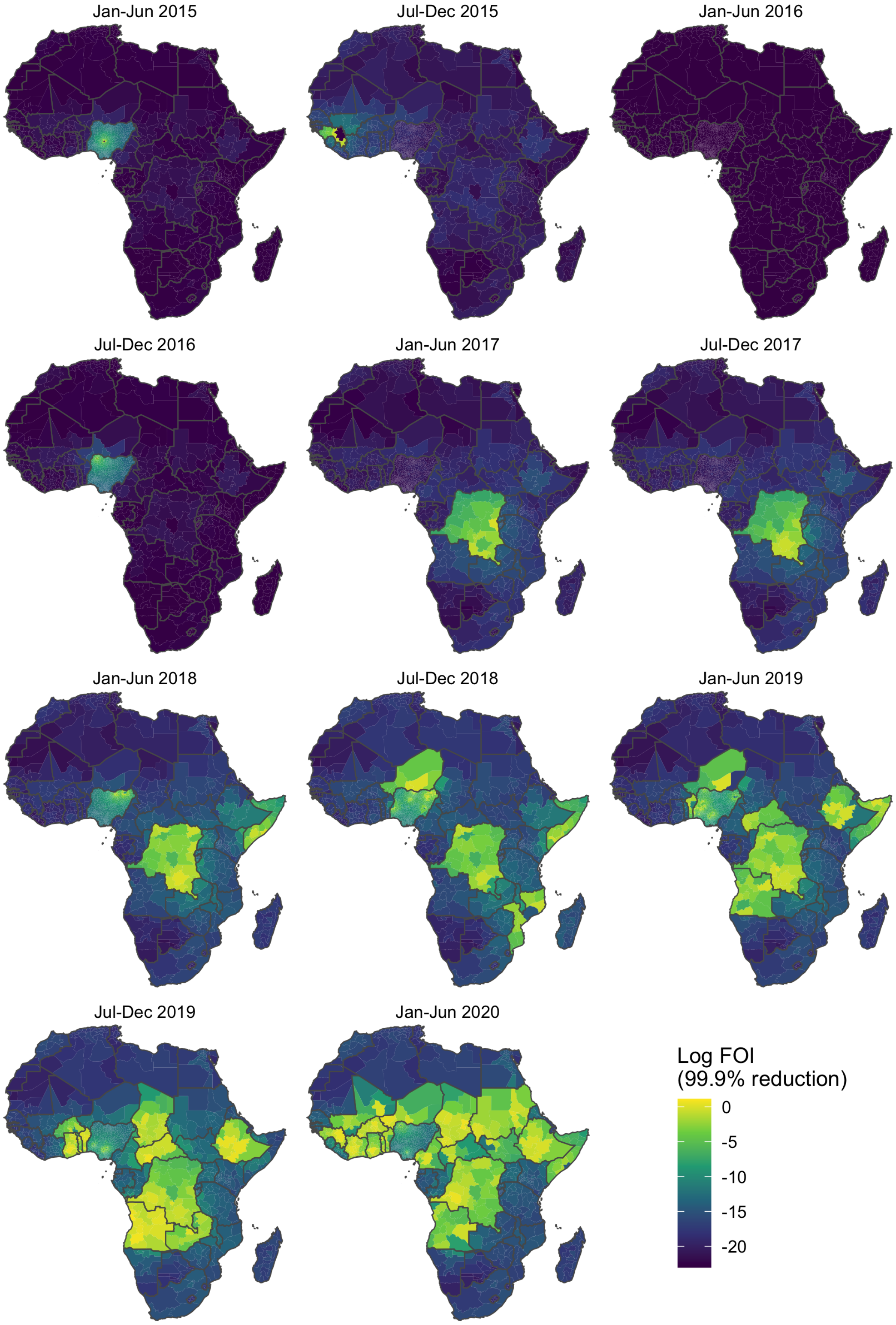

Figure S22: Natural log of the force of infection from external provinces and districts with 99.9% reduction in movement across international borders. The publication of this map does not imply the expression of any opinion whatsoever on the part of WHO concerning the legal status of any territory, city or area or of its authorities, or concerning the delimitation of its frontiers or boundaries.

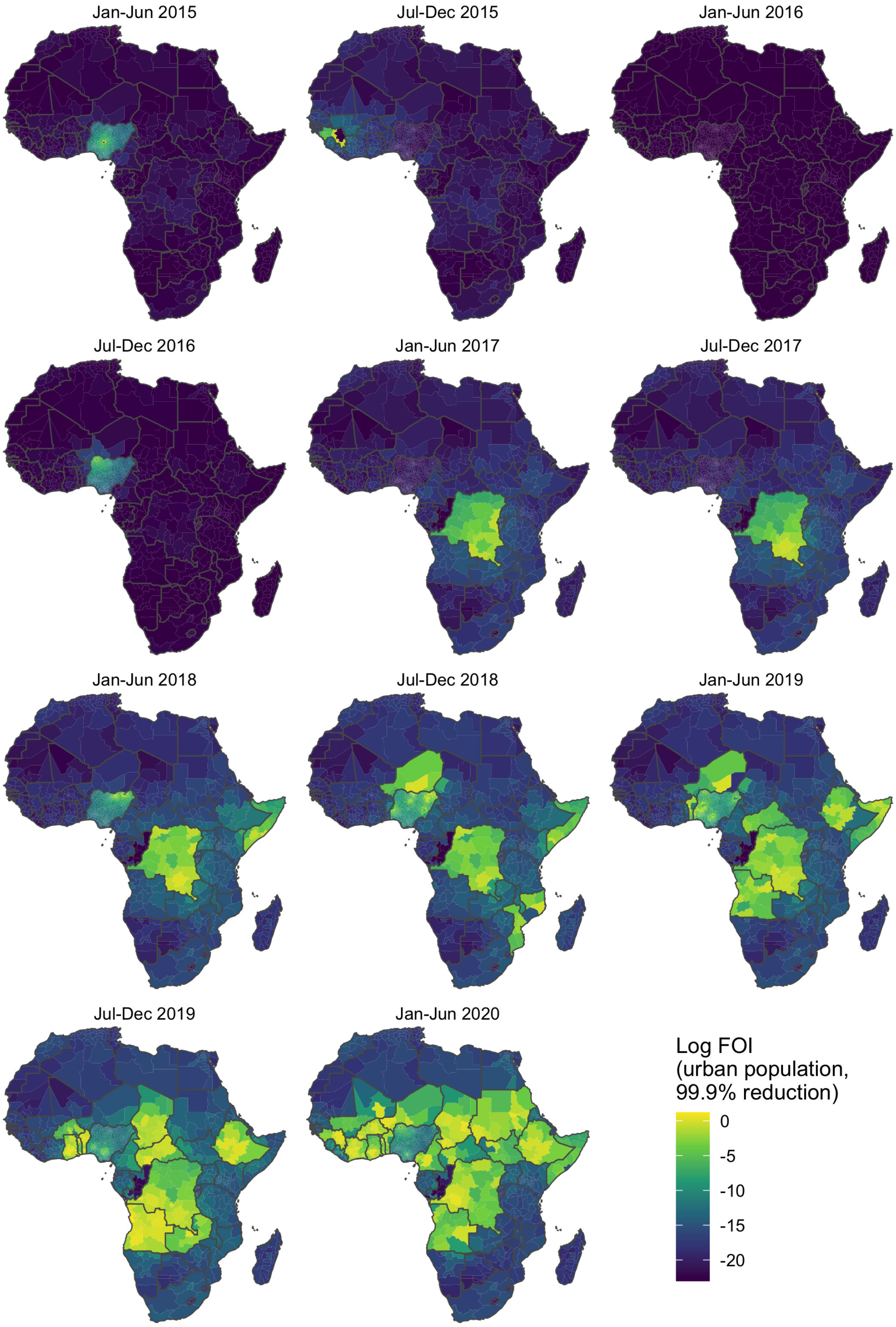

Figure S23: Natural log of the force of infection from external provinces and districts with 99.9% reduction in movement across international borders using population in urban areas only. The publication of this map does not imply the expression of any opinion whatsoever on the part of WHO concerning the legal status of any territory, city or area or of its authorities, or concerning the delimitation of its frontiers or boundaries.

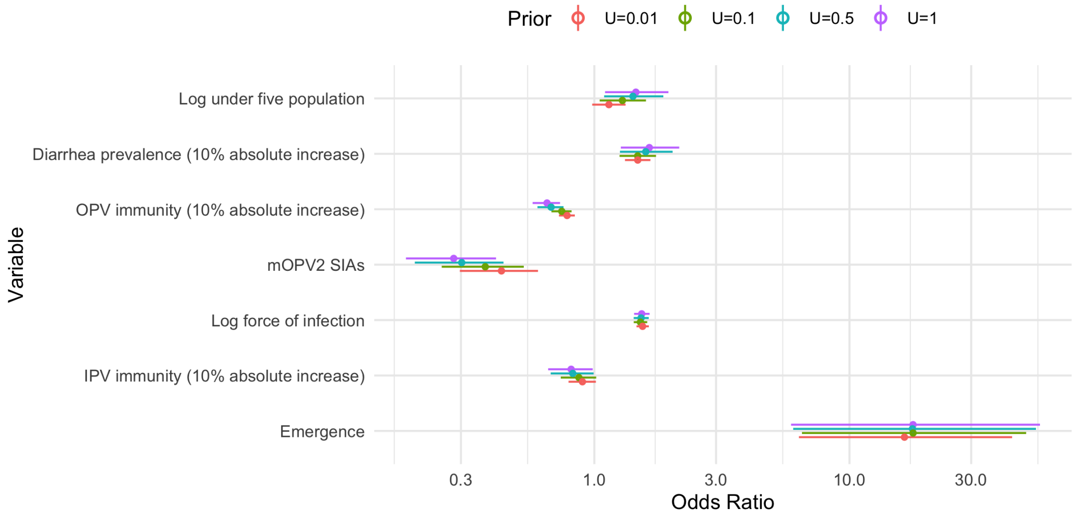

Figure S24: Value of fixed-effects variables in the final mixed effects model of cVDPV2 spread using different priors.

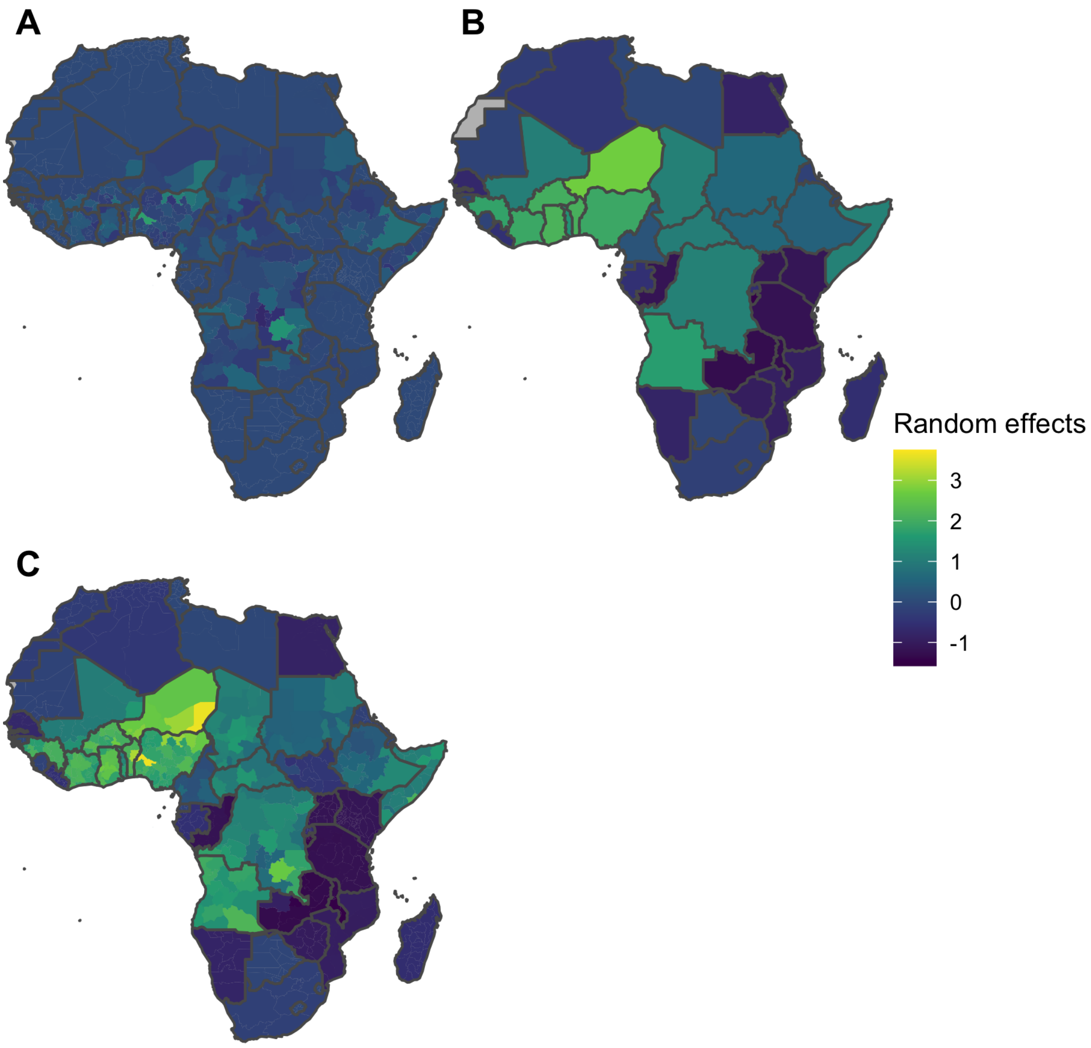

Figure S25: Value of A) province- and B) country-level time-invariant random effects μ and τ and sum of province- and country-level effects C) in the final mixed effects model of cVDPV2 spread. The publication of this map does not imply the expression of any opinion whatsoever on the part of WHO concerning the legal status of any territory, city or area or of its authorities, or concerning the delimitation of its frontiers or boundaries.

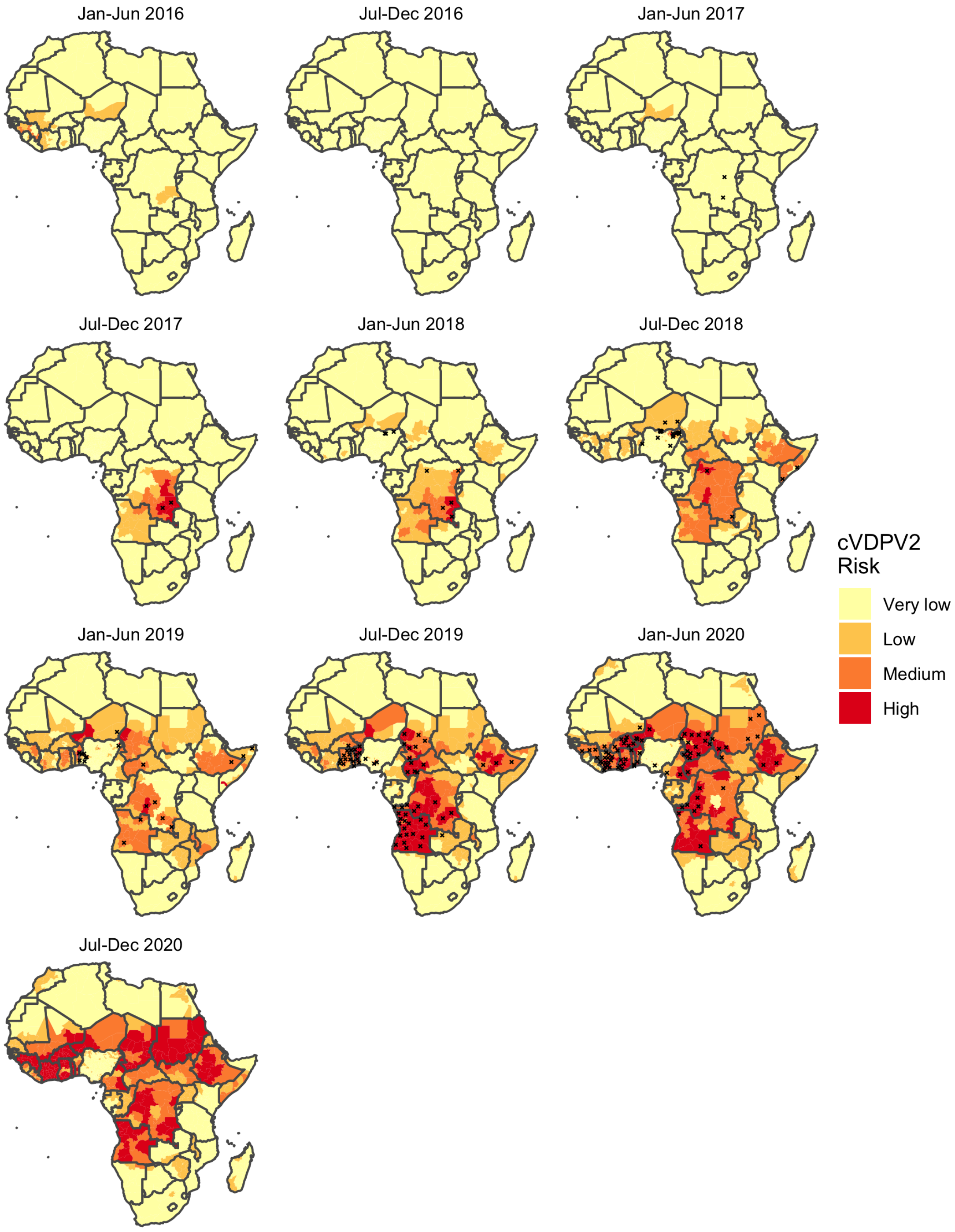

Figure S26: Predicted risk of detecting a cVDPV2 case between 1 January 2016 and 31 December 2020. Very low: 97.5th percentile of risk less than threshold (probability 0.07). Low: 50th percentile of risk less than threshold. Medium: 50th percentile of risk greater than threshold. High: 2.5th percentile of risk greater than threshold. Black crosses indicate provinces or districts where one or more cVDPV2 cases were reported (not shown for July-December 2020 period). The publication of this map does not imply the expression of any opinion whatsoever on the part of WHO concerning the legal status of any territory, city or area or of its authorities, or concerning the delimitation of its frontiers or boundaries.

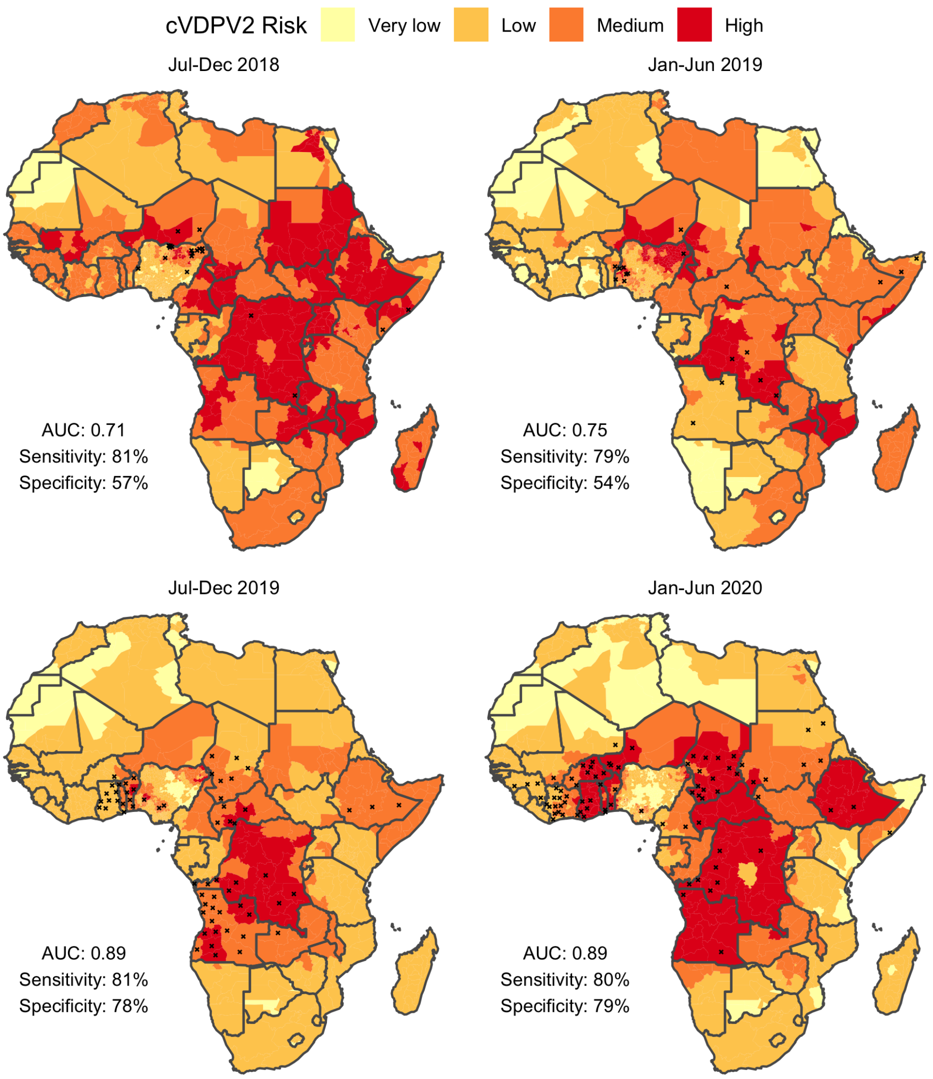

Figure S27: Six-month-ahead out-of-sample predicted risk of detecting a cVDPV2 case from July-December 2018 to January-June 2020. Very low: 97.5th percentile of risk less than threshold. Low: 50th percentile of risk less than threshold. Medium: 50th percentile of risk greater than threshold. High: 2.5th percentile of risk greater than threshold. Black crosses indicate provinces or districts where one or more cVDPV2 cases were reported. AUC: area under the curve. The publication of this map does not imply the expression of any opinion whatsoever on the part of WHO concerning the legal status of any territory, city or area or of its authorities, or concerning the delimitation of its frontiers or boundaries.

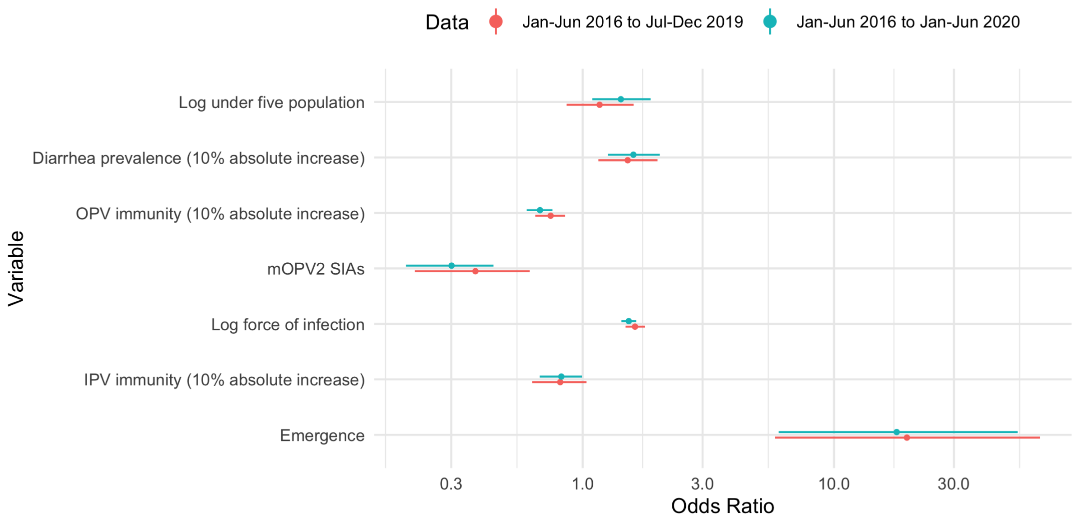

Figure S28: Sensitivity of fixed effects to Jan-Jun 2020 period. Values of fixed-effects variables in the final mixed effects model of cVDPV2 spread fitted to data Jan 2016-Dec 2019 (red) and Jan 2016-Jun 2020 (blue).

Table S11: Risk factors associated with the spread of circulating vaccine-derived type 2 poliovirus based on multivariable mixed-effects lagged regression model for January-June 2016 to January-June 2020 using a categorical variable for type 2 IPV population immunity, WAIC 1079.3. cOR = crude odds ratio; aOR = adjusted odds ratio; 95% CrI = 95% credible interval.

Fixed effects variable	cOR	95% CrI		aOR	95% CrI	
Diarrhea prevalence (under 5 years, 10% increase)	1.74	(1.60-	1.91)	1.60	(1.26-	2.05)
Emergence (previous 6 months)	78.1	(37.2-	165)	17.4	(5.88-	53.5)
IPV immunity (previous 6 months, under 5 years, 10% increase)						
0-15%	1.00			1.00		
15-30%	2.03	(1.49-	2.78)	1.15	(0.700-	1.92)
30-45%	1.32	(0.855-	1.99)	0.818	(0.418-	1.58)
45-60%	0.620	(0.224-	1.37)	0.35	(0.101-	1.00)
60-75%	0.698	(0.0662-	3.39)	0.393	(0.0301-	2.43)
Log FOI (previous 6 months, external)	1.57	(1.5-	1.64)	1.51	(1.42-	1.62)
Log population size	2.14	(1.91-	2.39)	1.41	(1.09-	1.86)
mOPV2 rounds (previous 6 months)	0.931	(0.690-	1.20)	0.298	(0.195-	0.434)
OPV immunity (previous 6 months, under 5 years, 10% increase)	0.667	(0.629-	0.705)	0.691	(0.611-	0.772)
**Variable (random effects)**				**Median**	**95% CrI**	
Province (precision)				1.63	(0.800-	3.88)
Country (precision)				0.536	(0.289-	1.02)

Table S12: Sensitivity analysis for model predictions, July to December 2020. WAIC = Watanabe Akaike Information Criterion. Sensitivity, specificity using threshold of q_{80}. Number of districts or provinces at very, low, medium, or high risk for July-December 2020 using threshold q_{80}. Expected number of districts or provinces with cVDPV2 cases reported July-December 2020. 95% CI = 95% confidence interval, calculated as described in section 1.2.8.

					Number of locations at risk				Expected locations with cases (95%CI)		
Name	WAIC	q_{80}	Sens.	Spec.	Very low	Low	Medium	High			
Main model	1078.8	0.070	0.80	0.97	1011	240	88	150	121	(107 -	136)
Sensitivity analysis: categorical variable for mOPV2 SIAs	1074.1	0.068	0.80	0.97	973	274	97	145	121	(107 -	136)
Sensitivity analysis: decreased international movement	1078.8	0.070	0.80	0.97	1225	56	71	137	107	(95 -	121)

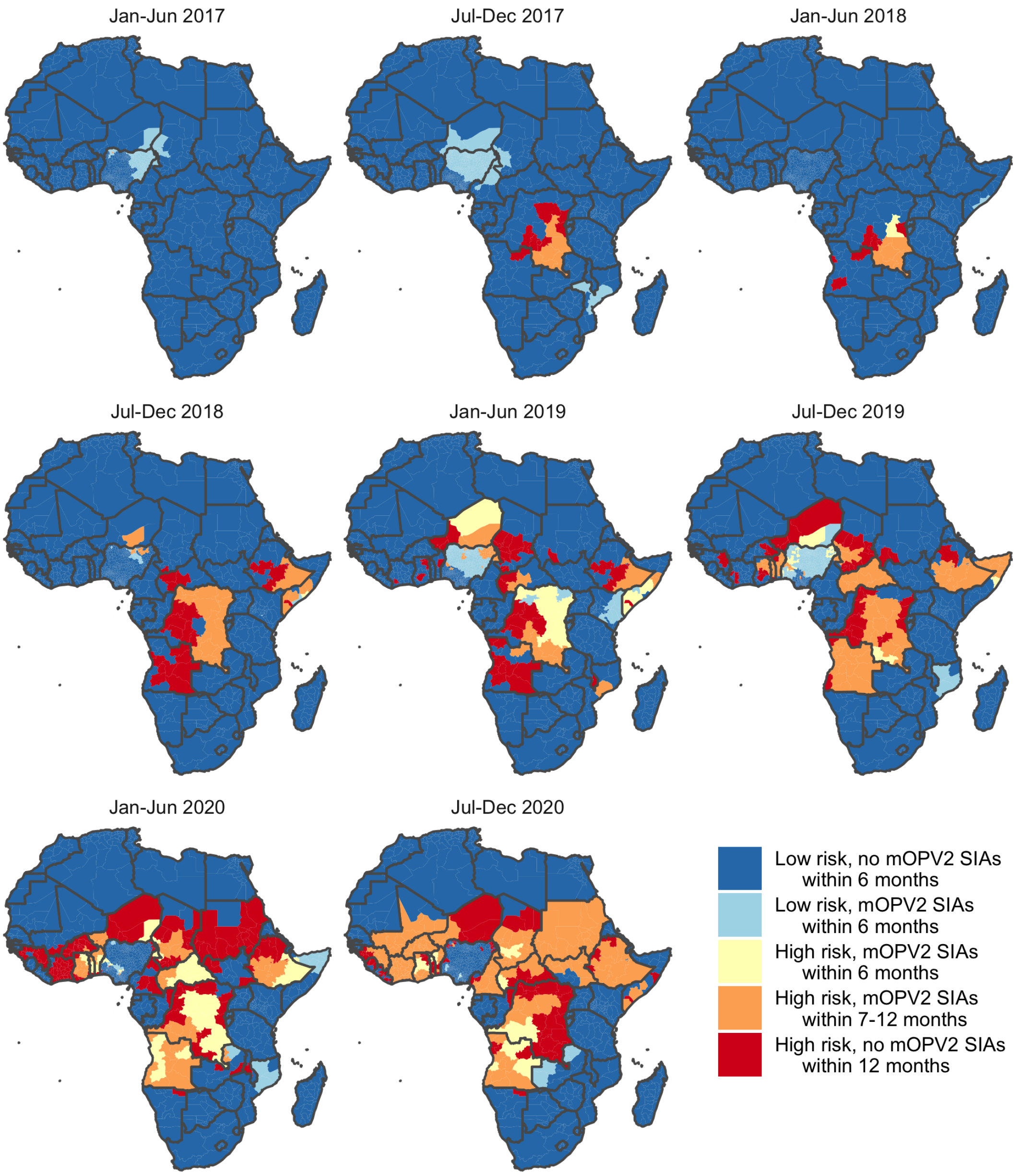

Figure S29: Comparison of mOPV2 SIAs and predicted cVDPV2 risk between July 2016 and December 2020. Dark blue shows districts and provinces at low or very low cVDPV2 risk and no mOPV2 SIAs took place within 6 months. Light blue shows districts and provinces at low or very low cVDPV2 risk and mOPV2 SIAs took place within 6 months. Yellow shows districts and provinces at high or medium cVDPV2 risk and mOPV2 SIAs took place within 6 months. Orange shows districts and provinces where model predicted high or medium risk and mOPV2 SIAs took place within 7-12 months. Red shows districts and provinces at high or medium cVDPV2 risk and mOPV2 SIAs did not take place within 12 months. The publication of this map does not imply the expression of any opinion whatsoever on the part of WHO concerning the legal status of any territory, city or area or of its authorities, or concerning the delimitation of its frontiers or boundaries.

Table S13: Comparison of actual outbreak response January-June 2020 and July-December 2020 and model-defined optimal response. Number of districts or provinces with 0, 1, 2, 3 or 4 or more SIAs per six-month period. Expected number of districts or provinces with cVDPV2 cases reported, 95% CI = 95% confidence interval, calculated as described in section 1.2.8. Total doses mOPV2 required (million children).

Category	Locations with mOPV2 SIAs					Locations with cVDPV2			Doses mOPV2 (million)
	0	1	2	3	>3	Median (95%CI)			
Actual use									
Jan-Jun 2020	1413	59	17	0	0	121	(107 -	136)	19.20
Jul-Dec 2020	1317	51	118	2	0				78.81
Targeting high and medium risk Jul-Dec 2020									
Main model	1239	139	109	1	1	20	(12 -	29)	129.42
Sensitivity analysis: 50% coverage of mOPV2 SIAs	1239	106	131	12	1	20	(12 -	29)	140.55
Sensitivity analysis: categorical variable for mOPV2 SIAs	1238	204	42	4	1	20	(12 -	29)	103.71
Sensitivity analysis: decreased international movement	1268	115	104	1	1	10	(5 -	16)	123.63

Table S14: Mean reported OPV doses by sex and age and p-value of Student t-test for difference in two sample means.

Age	Number of npAFP		Mean OPV doses		t-test
(months)	Female	Male	Female	Male	p-value
0-11	6925	8065	4.10	4.17	0.15
12-23	15604	19301	6.59	6.60	0.85
24-35	12885	16751	8.30	8.37	0.25
36-47	8189	10639	9.59	9.73	0.21
48-59	5559	7589	10.75	10.72	0.89
0-59	49162	62345	7.66	7.80	<0.001

References

1. Church JA, Parker EP, Kirkpatrick BD, Grassly NC, and Prendergast AJ. Interventions to improve oral vaccine performance: a systematic review and meta-analysis. The Lancet Infectious Diseases 2019;19:203–14.
2. Cáceres VM and Sutter RW. Sabin monovalent oral polio vaccines: Review of past experiences and their potential use after polio eradication. 2001. DOI: 10.1086/321905. URL: https://academic.oup.com/cid/article-lookup/doi/10.1086/321905.
3. Macklin GR, Grassly NC, Sutter RW, et al. Vaccine schedules and the effect on humoral and intestinal immunity against poliovirus: a systematic review and network meta-analysis. The Lancet Infectious Diseases 2019;19.
4. McBean AM, Thoms ML, Albrecht P, Cuthie JC, and Bernier R. Serologic Response to Oral Polio Vaccine and Enhanced-Potency Inactivated Polio Vaccines. American Journal of Epidemiology 1988;128:615–28.
5. Patriarca PA, Wright PF, and John TJ. Factors affecting the immunogenicity of oral poliovirus vaccine in developing countries: Review. 1991. DOI: 10.1093/clinids/13.5.926.
6. Asturias EJ, Bandyopadhyay AS, Self S, et al. Humoral and intestinal immunity induced by new schedules of bivalent oral poliovirus vaccine and one or two doses of inactivated poliovirus vaccine in Latin American infants: an open-label randomised controlled trial. The Lancet 2016;388:158–69.
7. Deus N de, Capitine IPU, Bauhofer AFL, et al. Immunogenicity of Reduced-Dose Monovalent Type 2 Oral Poliovirus Vaccine in Mocuba, Mozambique. The Journal of Infectious Diseases 2020.
8. Zaman K, Estívariz CF, Morales M, et al. Immunogenicity of type 2 monovalent oral and inactivated poliovirus vaccines for type 2 poliovirus outbreak response: an open-label, randomised controlled trial. The Lancet Infectious Diseases 2018;18:657–65.
9. Saleem AF, Yousafzai MT, Mach O, et al. Evaluation of vaccine derived poliovirus type 2 outbreak response options: A randomized controlled trial, Karachi, Pakistan. Vaccine 2018;36:1766–71.
10. Sutter RW, John TJ, Jain H, et al. Immunogenicity of bivalent types 1 and 3 oral poliovirus vaccine: A randomised, double-blind, controlled trial. The Lancet 2010.
11. United Nations Development Programme. Under-five mortality rate (per 1,000 live births) | Human Development Reports. 2020. URL: http://hdr.undp.org/en/content/under-five-mortality-rate-1000-live-births (visited on 02/08/2021).
12. Anderson D and Burnham K. Model selection and multi-model inference. Second. New York: Springer-Verlag, 2004.
13. Mangal TD, Aylward RB, Mwanza M, et al. Key issues in the persistence of poliomyelitis in Nigeria: a case-control study. The Lancet Global Health 2014;2:e90–e97.
14. Levitt A, Diop OM, Tangermann RH, et al. Surveillance systems to track progress toward global polio eradication—worldwide, 2012–2013. MMWR. Morbidity and mortality weekly report 2014;63:356.
15. World Health Organization. Polio Information System. URL: https://extranet.who.int/polis/public/CaseCount.aspx (visited on 03/11/2021).
16. Grassly NC, Fraser C, Wenger J, et al. New strategies for the elimination of polio from India. Science (New York, N.Y.) 2006;314:1150–3.
17. Pons-Salort M, Molodecky NA, O'Reilly KM, et al. Population Immunity against Serotype-2 Poliomyelitis Leading up to the Global Withdrawal of the Oral Poliovirus Vaccine: Spatio-temporal Modelling of Surveillance Data. PLOS Medicine 2016;13. Ed. by Viboud C:e1002140.
18. Mosser JF, Gagne-Maynard W, Rao PC, et al. Mapping diphtheria-pertussis-tetanus vaccine coverage in Africa, 2000–2016: a spatial and temporal modelling study. The Lancet 2019;393:1843–55.

19. World Health Organization. WHO and UNICEF Joint Skilled Birth Attendant (SBA) database. Tech. rep. Geneva, Switzerland: World Health Organization, 2019.

20. Rue H, Martino S, and Chopin N. Approximate Bayesian inference for latent Gaussian models by using integrated nested Laplace approximations. Journal of the Royal Statistical Society: Series B (Statistical Methodology) 2009;71:319–92.

21. Grassly NC. Immunogenicity and Effectiveness of Routine Immunization With 1 or 2 Doses of Inactivated Poliovirus Vaccine: Systematic Review and Meta-analysis. The Journal of Infectious Diseases 2014;210:S439–S446.

22. Burton A, Monasch R, Lautenbach B, et al. WHO and UNICEF estimates of national infant immunization coverage: Methods and processes. 2009. DOI: 10.2471/BLT.08.053819.

23. Simini F, González MC, Maritan A, and Barabási AL. A universal model for mobility and migration patterns. Nature 2012;484:96–100.

24. Tatem AJ, Garcia AJ, Snow RW, et al. Millennium development health metrics: Where do Africa's children and women of childbearing age live? Population Health Metrics 2013;11:11.

25. Smits J and Permanyer I. Subnational Human Development Database. GDL Working Paper Series 2019:1–16.

26. Reiner RC, Wiens KE, Deshpande A, et al. Mapping geographical inequalities in childhood diarrhoeal morbidity and mortality in low-income and middle-income countries, 2000-17: Analysis for the Global Burden of Disease Study 2017. The Lancet 2020;395:1779–801.

27. Deshpande A, Miller-Petrie MK, Lindstedt PA, et al. Mapping geographical inequalities in access to drinking water and sanitation facilities in low-income and middle-income countries, 2000–17. The Lancet Global Health 2020;8:e1162–e1185.

28. Watanabe S and Opper M. Asymptotic equivalence of Bayes cross validation and widely applicable information criterion in singular learning theory. Journal of machine learning research 2010;11.

29. Simpson D, Rue H, Riebler A, Martins TG, and Sørbye SH. Penalising model component complexity: A principled, practical approach to constructing priors. Statistical Science 2017;32:1–28.

30. Gofama MM, Verma H, Abdullahi H, et al. Survey of poliovirus antibodies in Borno and Yobe States, North-Eastern Nigeria. PLOS ONE 2017;12. Ed. by Borrow R:e0185284.

31. Guindo O, Mach O, Doumbia S, et al. Assessment of poliovirus antibody seroprevalence in polio high risk areas of West Africa. Vaccine 2018;36:1027–31.

32. Razafindratsimandresy R, Mach O, Heraud JM, et al. Assessment of poliovirus antibody seroprevalence in high risk areas for vaccine derived poliovirus transmission in Madagascar. Heliyon 2018;4.

33. Verma H, Iliyasu Z, Craig KT, et al. Trends in Poliovirus Seroprevalence in Kano State, Northern Nigeria. Clinical Infectious Diseases 2018;67:S103–S109.

3 Appendix: OPV immunity time series

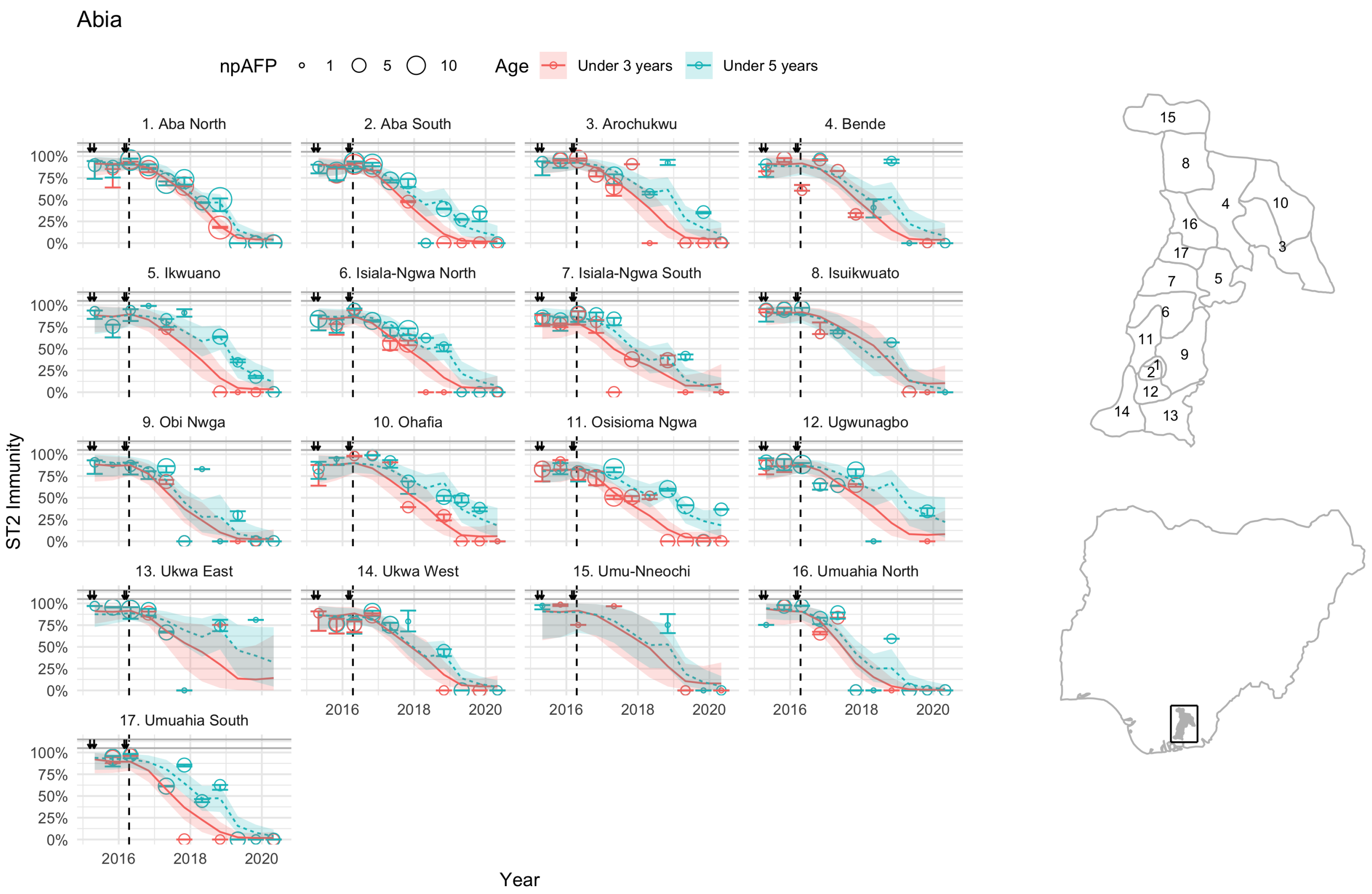

Figure S30: Type 2 population immunity from OPV in each district of Abia, Nigeria in children under five (blue) and under three (red). Circles show median of bootstrapped crude immunity estimates, error bars show 2.5th and 97.5th percentiles of bootstrapped estimates. Size of circles indicate the number of non-polio AFP cases that each crude estimate is based on. Lines show median smoothed immunity estimate, transparent ribbons show 95% credible interval. Arrows show timing of tOPV (before withdrawal, dotted line) or mOPV2 SIAs (after withdrawal, dotted line). Height of arrows should the proportion of under-five population targeted in SIA. The publication of this map does not imply the expression of any opinion whatsoever on the part of WHO concerning the legal status of any territory, city or area or of its authorities, or concerning the delimitation of its frontiers or boundaries.

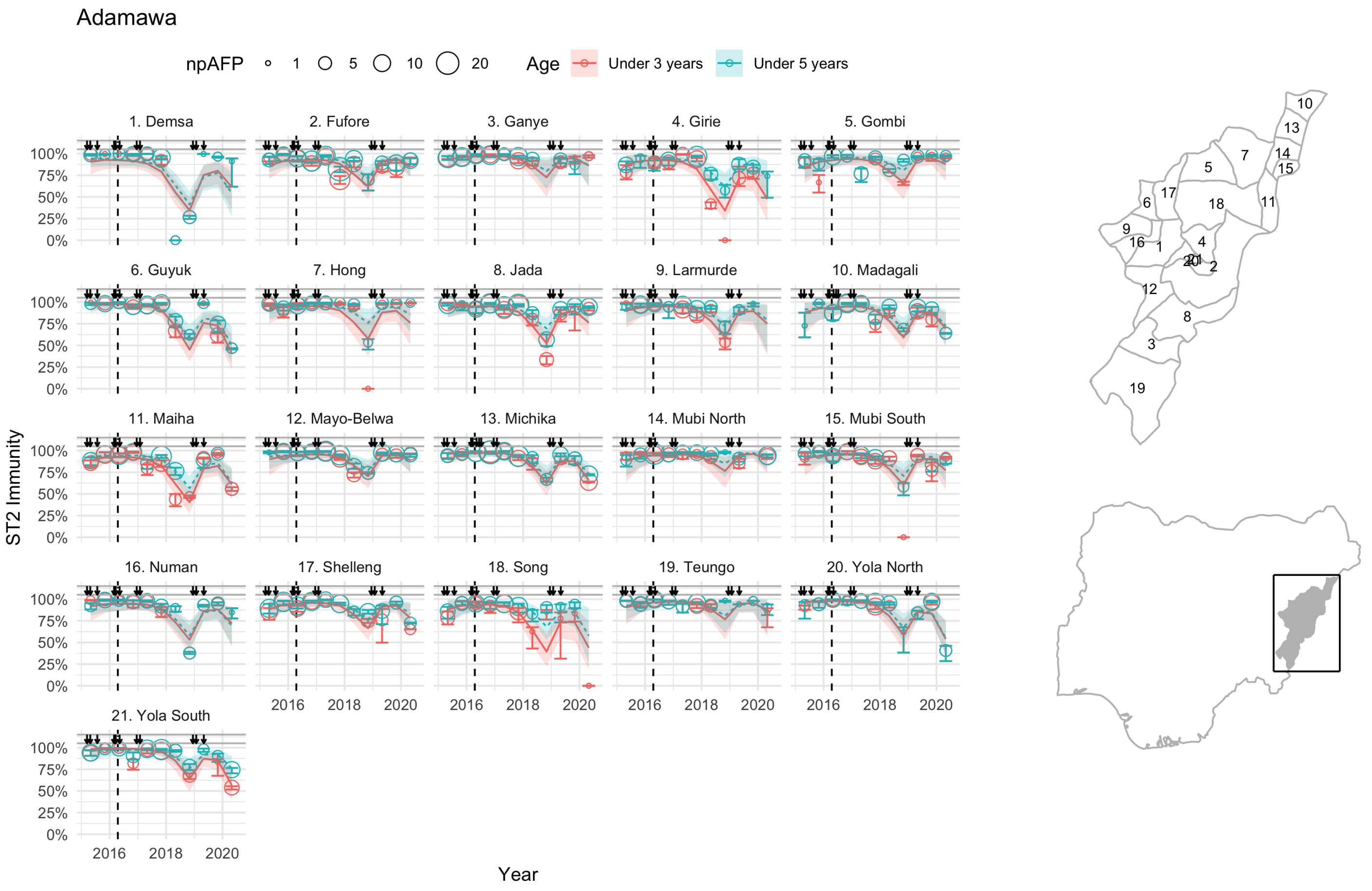

Figure S31: Type 2 population immunity from OPV in each district of Adamawa, Nigeria in children under five (blue) and under three (red). Circles show median of bootstrapped crude immunity estimates, error bars show 2.5th and 97.5th percentiles of bootstrapped estimates. Size of circles indicate the number of non-polio AFP cases that each crude estimate is based on. Lines show median smoothed immunity estimate, transparent ribbons show 95% credible interval. Arrows show timing of tOPV (before withdrawal, dotted line) or mOPV2 SIAs (after withdrawal, dotted line). Height of arrows should the proportion of under-five population targeted in SIA. The publication of this map does not imply the expression of any opinion whatsoever on the part of WHO concerning the legal status of any territory, city or area or of its authorities, or concerning the delimitation of its frontiers or boundaries.

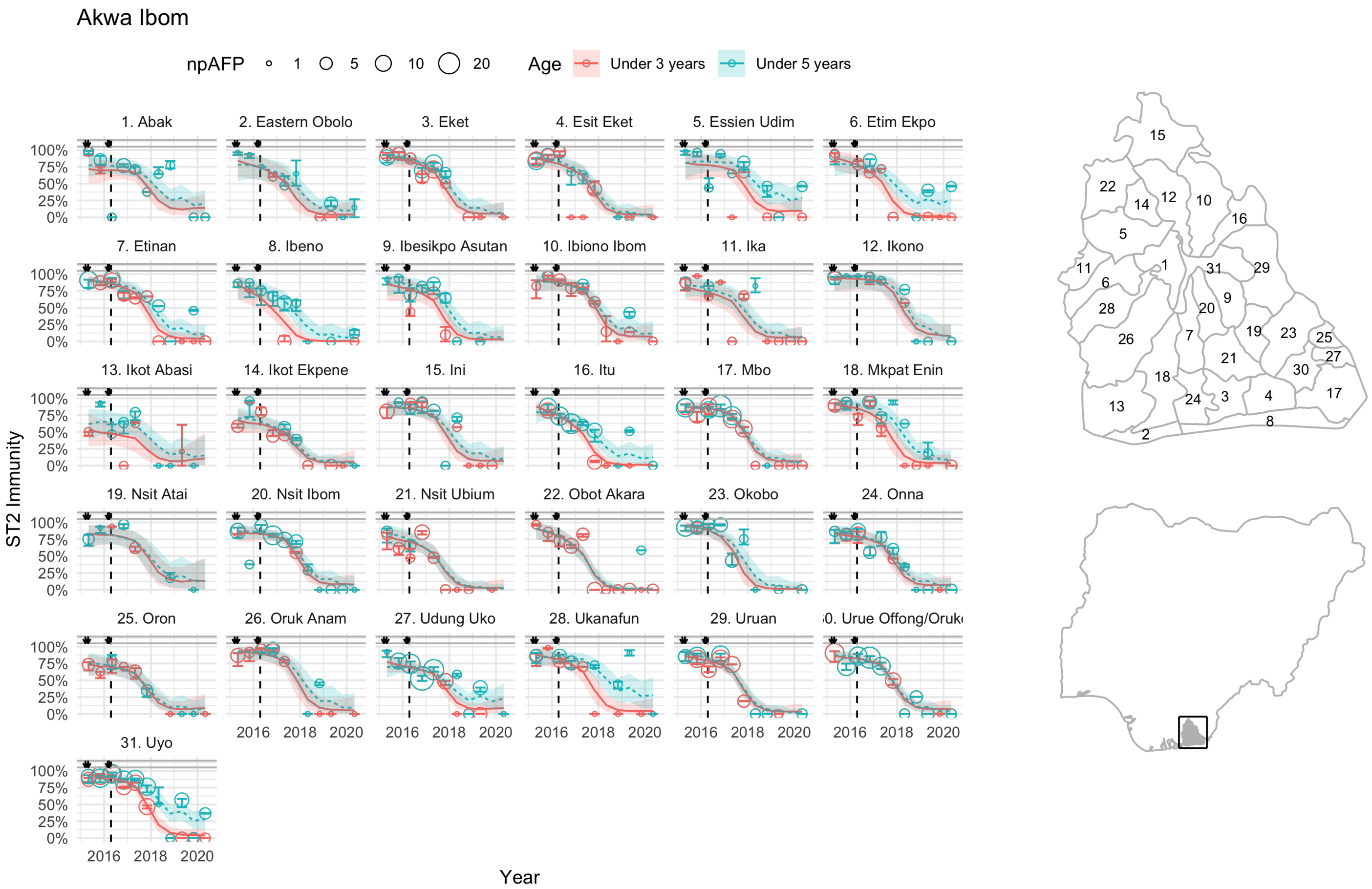

Figure S32: Type 2 population immunity from OPV in each district of Akwa Ibom, Nigeria in children under five (blue) and under three (red). Circles show median of bootstrapped crude immunity estimates, error bars show 2.5th and 97.5th percentiles of bootstrapped estimates. Size of circles indicate the number of non-polio AFP cases that each crude estimate is based on. Lines show median smoothed immunity estimate, transparent ribbons show 95% credible interval. Arrows show timing of tOPV (before withdrawal, dotted line) or mOPV2 SIAs (after withdrawal, dotted line). Height of arrows should the proportion of under-five population targeted in SIA. The publication of this map does not imply the expression of any opinion whatsoever on the part of WHO concerning the legal status of any territory, city or area or of its authorities, or concerning the delimitation of its frontiers or boundaries.

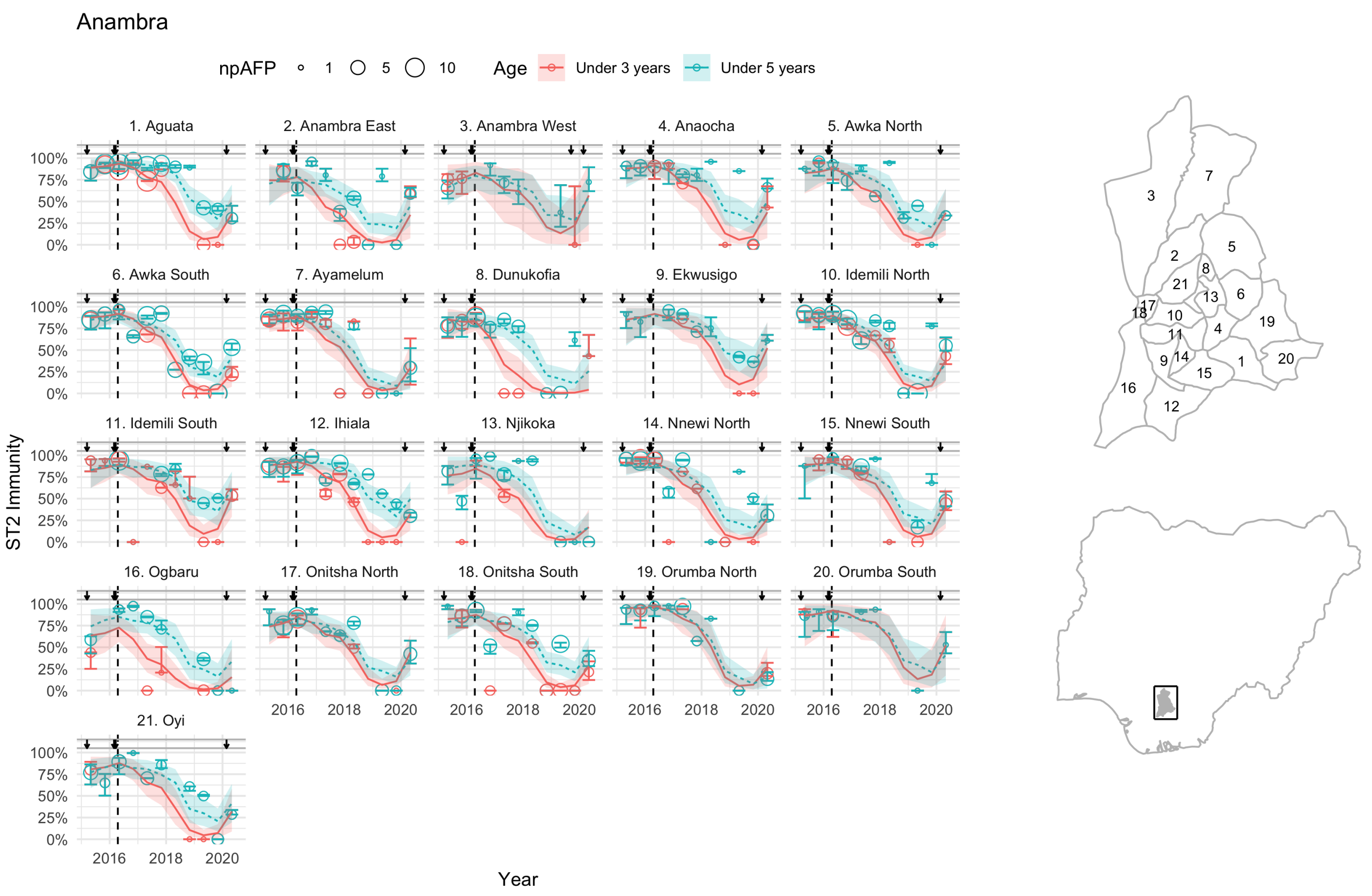

Figure S33: Type 2 population immunity from OPV in each district of Anambra, Nigeria in children under five (blue) and under three (red). Circles show median of bootstrapped crude immunity estimates, error bars show 2.5th and 97.5th percentiles of bootstrapped estimates. Size of circles indicate the number of non-polio AFP cases that each crude estimate is based on. Lines show median smoothed immunity estimate, transparent ribbons show 95% credible interval. Arrows show timing of tOPV (before withdrawal, dotted line) or mOPV2 SIAs (after withdrawal, dotted line). Height of arrows should the proportion of under-five population targeted in SIA. The publication of this map does not imply the expression of any opinion whatsoever on the part of WHO concerning the legal status of any territory, city or area or of its authorities, or concerning the delimitation of its frontiers or boundaries.

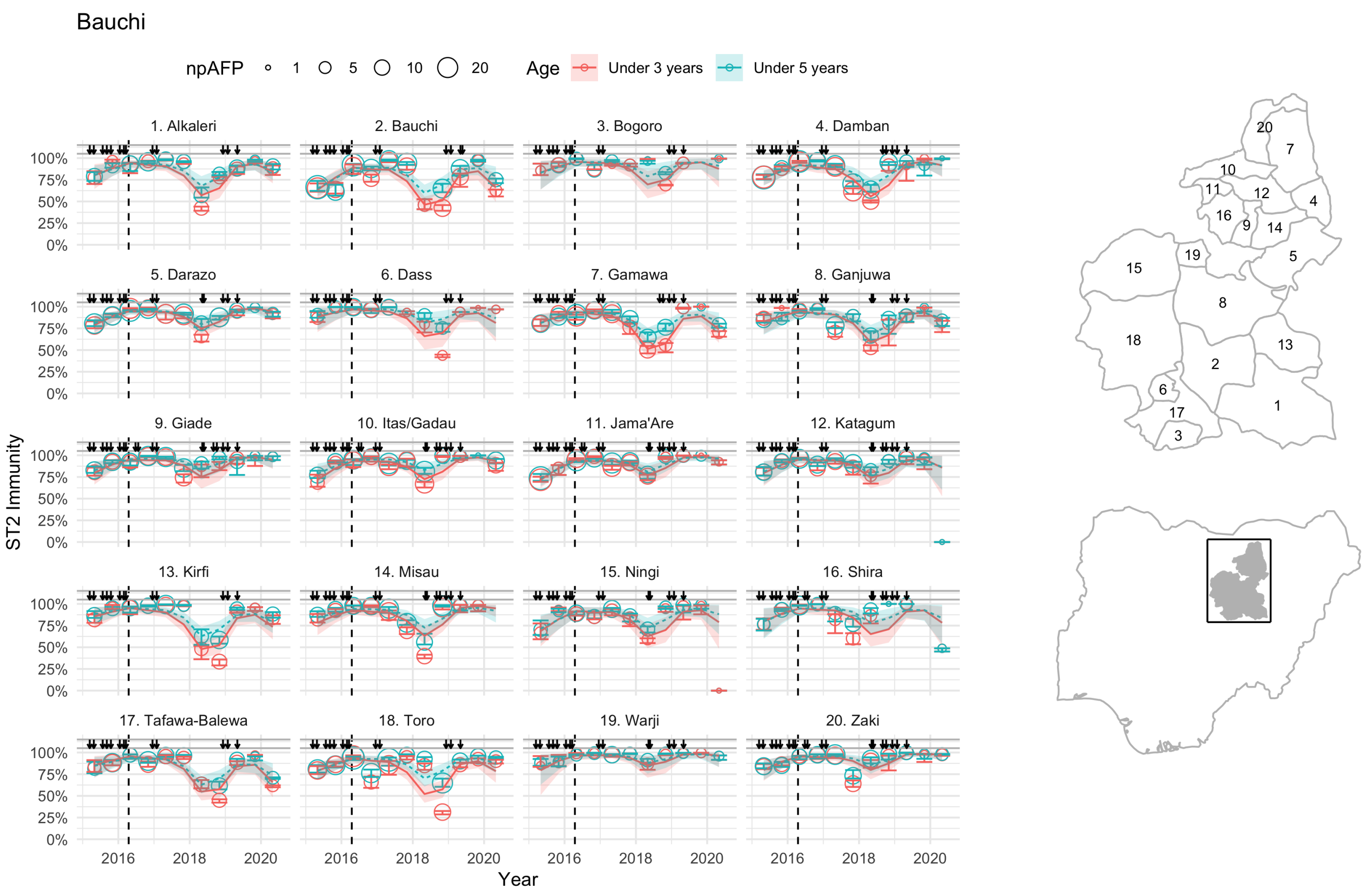

Figure S34: Type 2 population immunity from OPV in each district of Bauchi, Nigeria in children under five (blue) and under three (red). Circles show median of bootstrapped crude immunity estimates, error bars show 2.5th and 97.5th percentiles of bootstrapped estimates. Size of circles indicate the number of non-polio AFP cases that each crude estimate is based on. Lines show median smoothed immunity estimate, transparent ribbons show 95% credible interval. Arrows show timing of tOPV (before withdrawal, dotted line) or mOPV2 SIAs (after withdrawal, dotted line). Height of arrows should the proportion of under-five population targeted in SIA. The publication of this map does not imply the expression of any opinion whatsoever on the part of WHO concerning the legal status of any territory, city or area or of its authorities, or concerning the delimitation of its frontiers or boundaries.

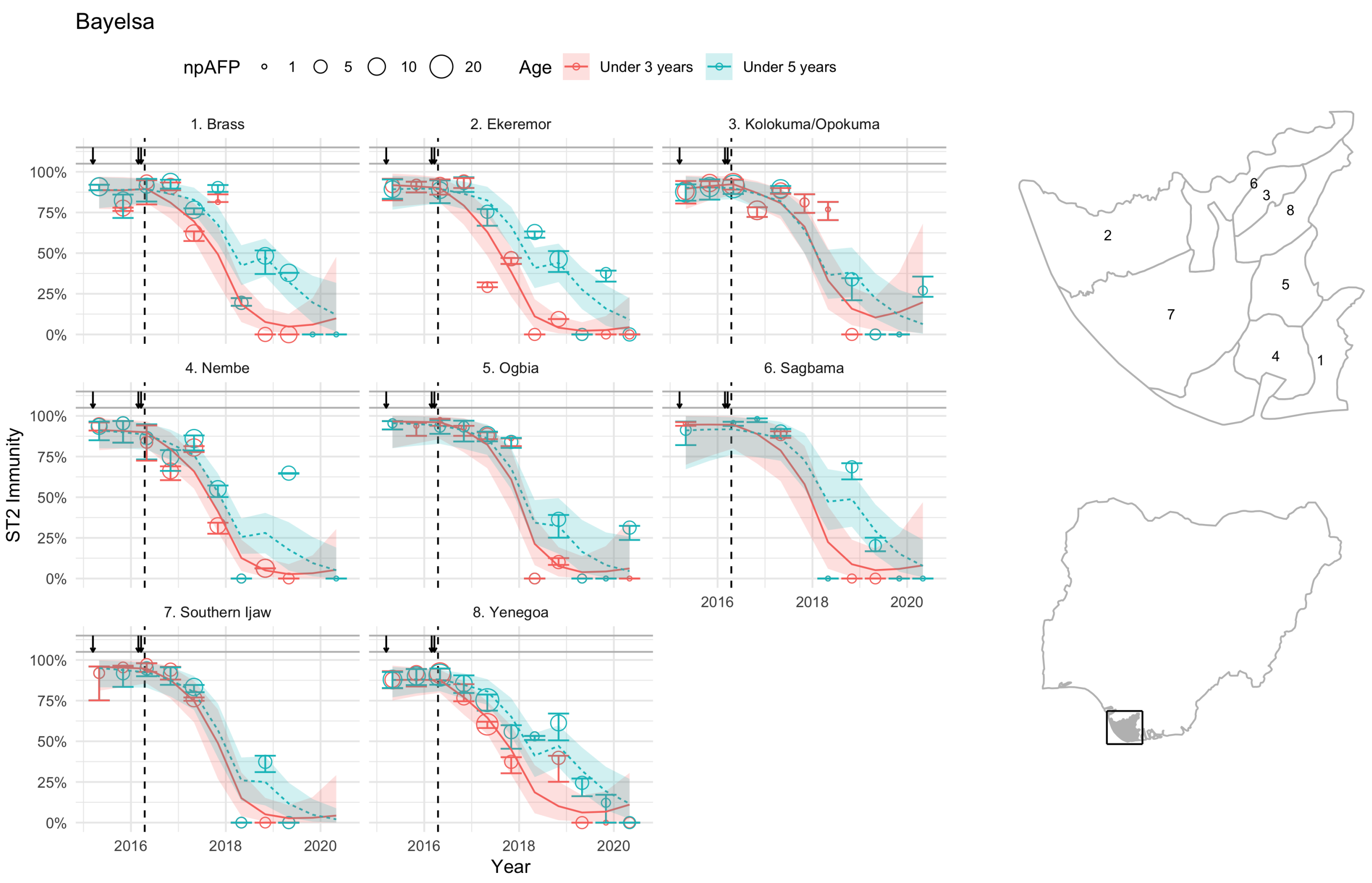

Figure S35: Type 2 population immunity from OPV in each district of Bayelsa, Nigeria in children under five (blue) and under three (red). Circles show median of bootstrapped crude immunity estimates, error bars show 2.5th and 97.5th percentiles of bootstrapped estimates. Size of circles indicate the number of non-polio AFP cases that each crude estimate is based on. Lines show median smoothed immunity estimate, transparent ribbons show 95% credible interval. Arrows show timing of tOPV (before withdrawal, dotted line) or mOPV2 SIAs (after withdrawal, dotted line). Height of arrows should the proportion of under-five population targeted in SIA. The publication of this map does not imply the expression of any opinion whatsoever on the part of WHO concerning the legal status of any territory, city or area or of its authorities, or concerning the delimitation of its frontiers or boundaries.

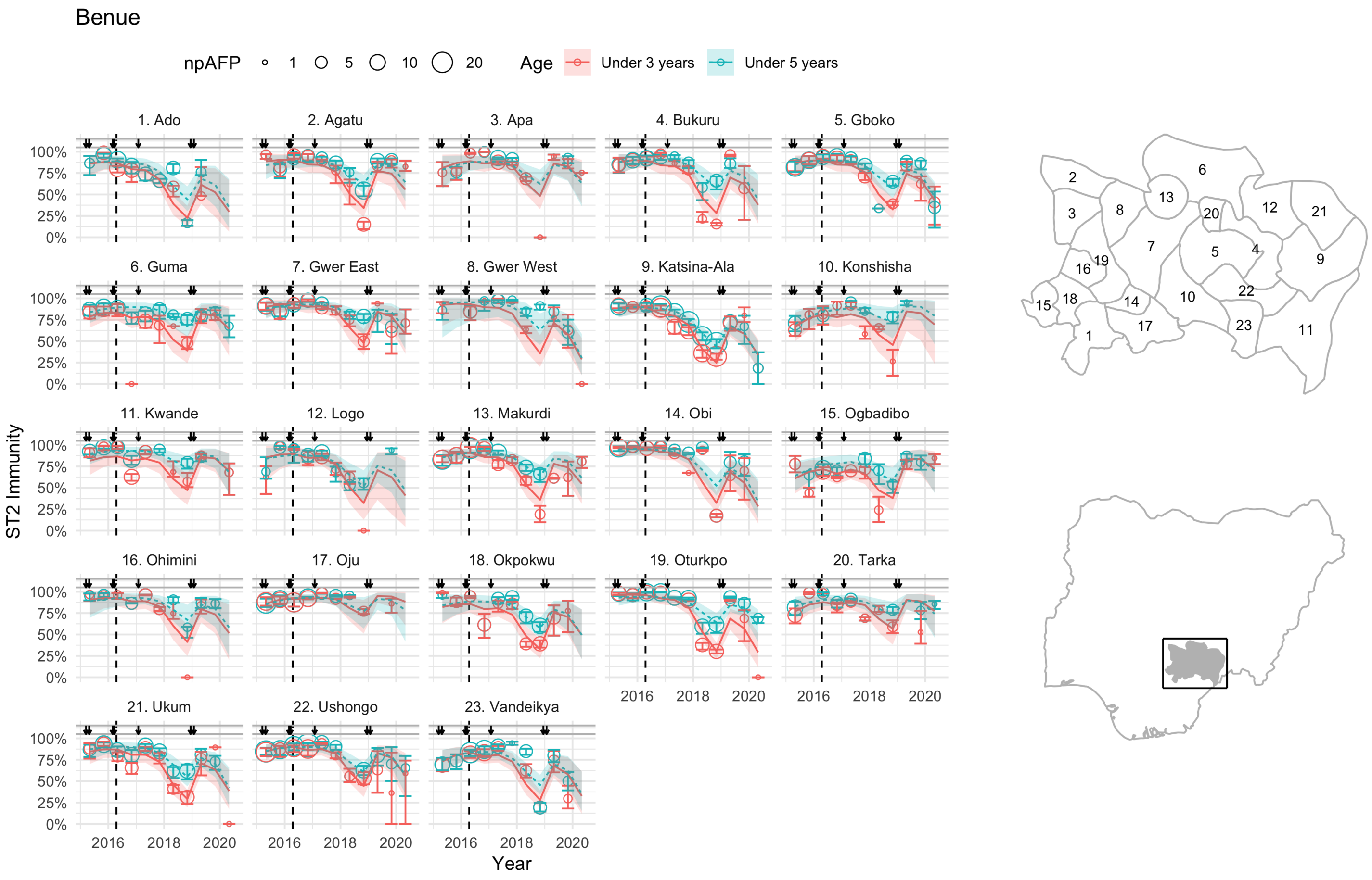

Figure S36: Type 2 population immunity from OPV in each district of Benue, Nigeria in children under five (blue) and under three (red). Circles show median of bootstrapped crude immunity estimates, error bars show 2.5th and 97.5th percentiles of bootstrapped estimates. Size of circles indicate the number of non-polio AFP cases that each crude estimate is based on. Lines show median smoothed immunity estimate, transparent ribbons show 95% credible interval. Arrows show timing of tOPV (before withdrawal, dotted line) or mOPV2 SIAs (after withdrawal, dotted line). Height of arrows should the proportion of under-five population targeted in SIA. The publication of this map does not imply the expression of any opinion whatsoever on the part of WHO concerning the legal status of any territory, city or area or of its authorities, or concerning the delimitation of its frontiers or boundaries.

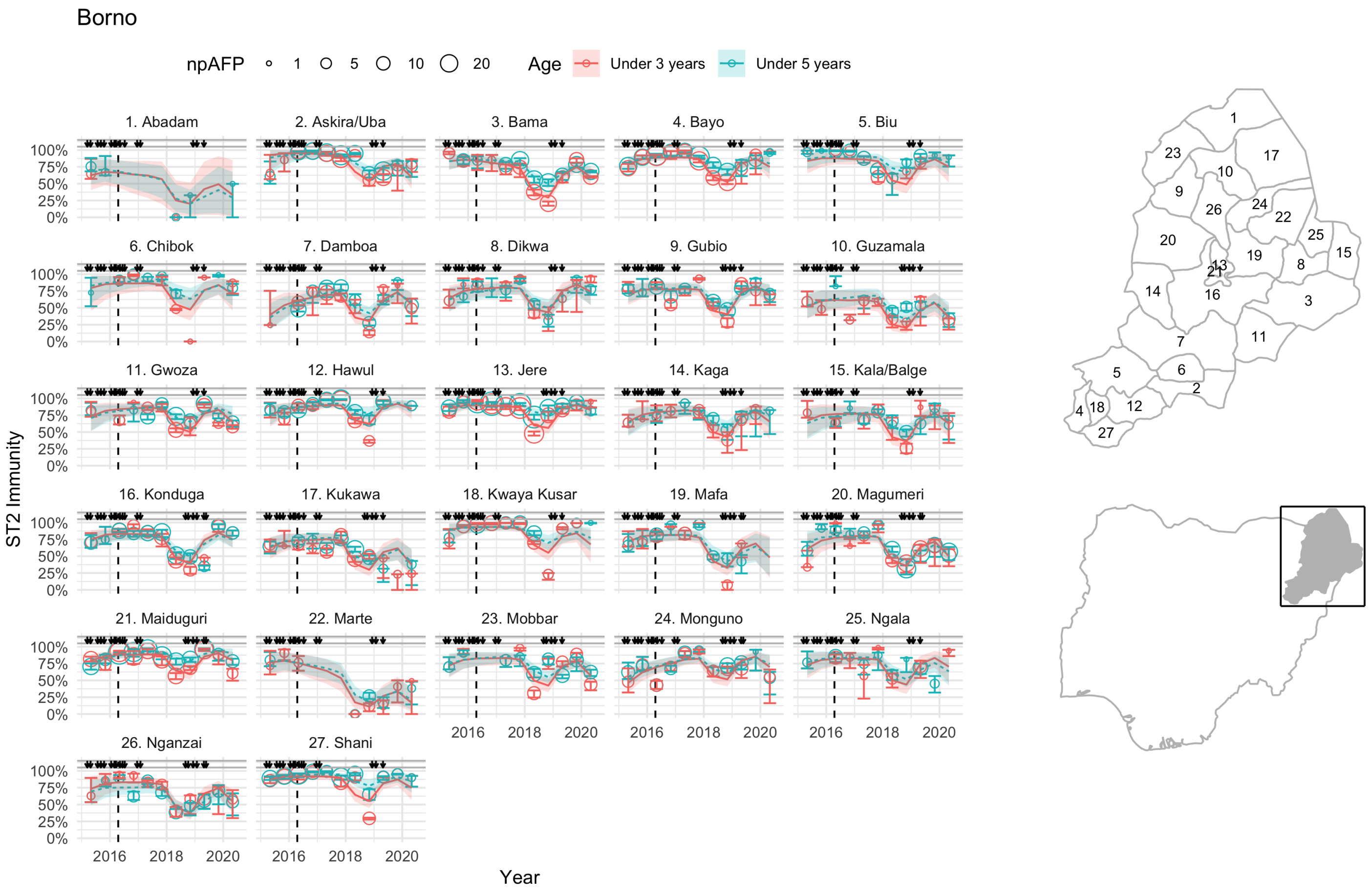

Figure S37: Type 2 population immunity from OPV in each district of Borno, Nigeria in children under five (blue) and under three (red). Circles show median of bootstrapped crude immunity estimates, error bars show 2.5th and 97.5th percentiles of bootstrapped estimates. Size of circles indicate the number of non-polio AFP cases that each crude estimate is based on. Lines show median smoothed immunity estimate, transparent ribbons show 95% credible interval. Arrows show timing of tOPV (before withdrawal, dotted line) or mOPV2 SIAs (after withdrawal, dotted line). Height of arrows should the proportion of under-five population targeted in SIA. The publication of this map does not imply the expression of any opinion whatsoever on the part of WHO concerning the legal status of any territory, city or area or of its authorities, or concerning the delimitation of its frontiers or boundaries.

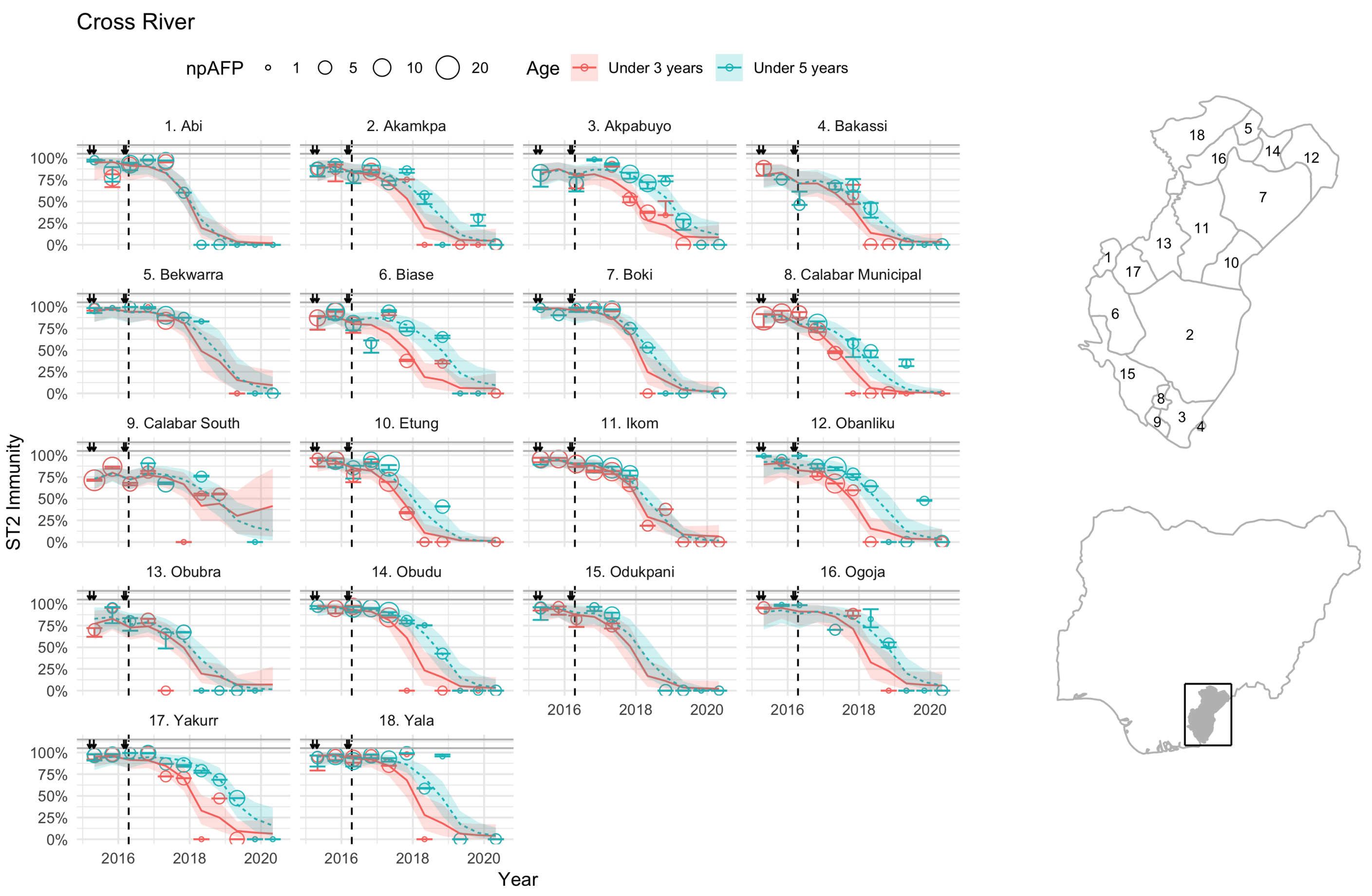

Figure S38: Type 2 population immunity from OPV in each district of Cross River, Nigeria in children under five (blue) and under three (red). Circles show median of bootstrapped crude immunity estimates, error bars show 2.5th and 97.5th percentiles of bootstrapped estimates. Size of circles indicate the number of non-polio AFP cases that each crude estimate is based on. Lines show median smoothed immunity estimate, transparent ribbons show 95% credible interval. Arrows show timing of tOPV (before withdrawal, dotted line) or mOPV2 SIAs (after withdrawal, dotted line). Height of arrows should the proportion of under-five population targeted in SIA. The publication of this map does not imply the expression of any opinion whatsoever on the part of WHO concerning the legal status of any territory, city or area or of its authorities, or concerning the delimitation of its frontiers or boundaries.

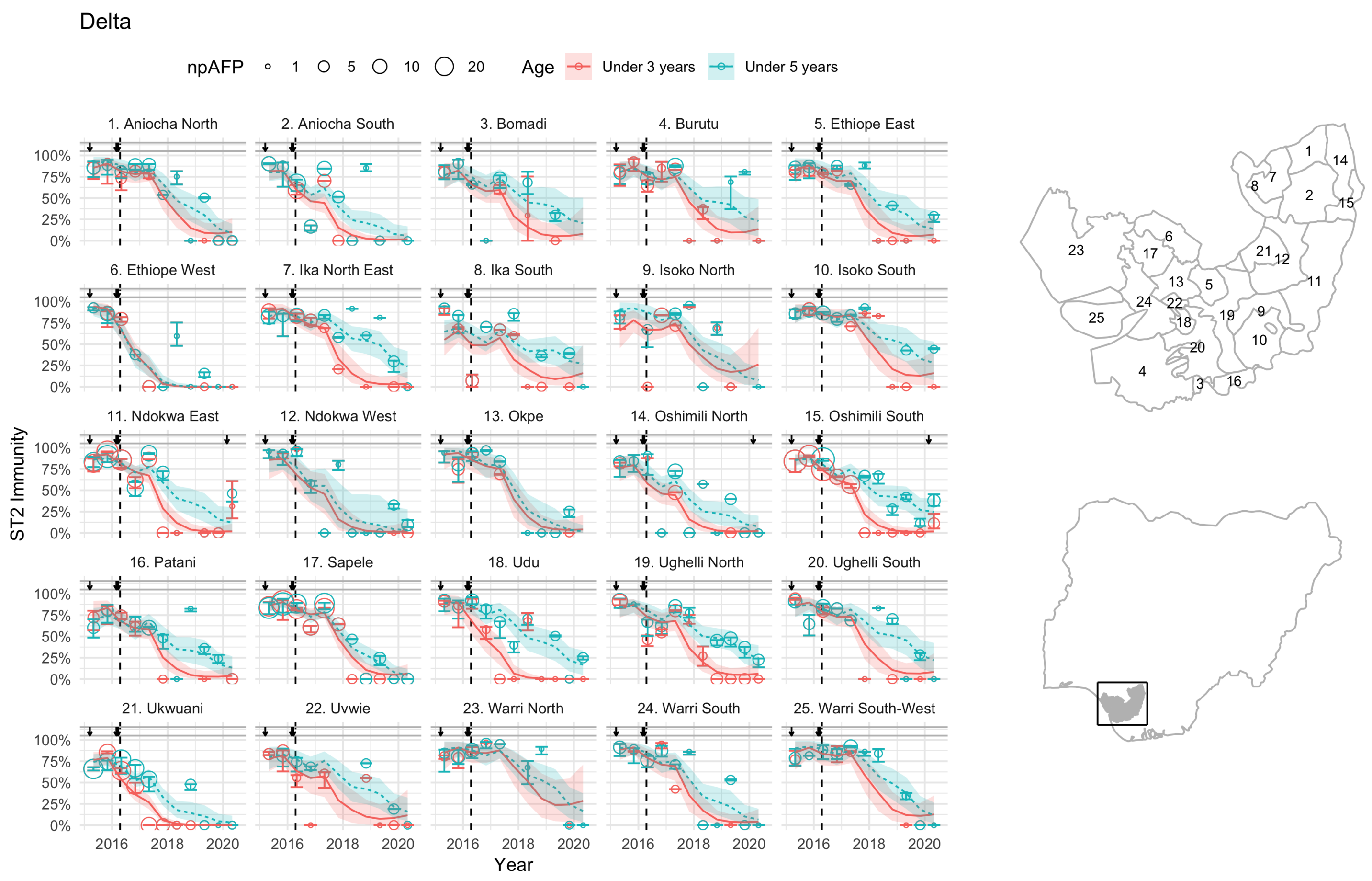

Figure S39: Type 2 population immunity from OPV in each district of Delta, Nigeria in children under five (blue) and under three (red). Circles show median of bootstrapped crude immunity estimates, error bars show 2.5th and 97.5th percentiles of bootstrapped estimates. Size of circles indicate the number of non-polio AFP cases that each crude estimate is based on. Lines show median smoothed immunity estimate, transparent ribbons show 95% credible interval. Arrows show timing of tOPV (before withdrawal, dotted line) or mOPV2 SIAs (after withdrawal, dotted line). Height of arrows should the proportion of under-five population targeted in SIA. The publication of this map does not imply the expression of any opinion whatsoever on the part of WHO concerning the legal status of any territory, city or area or of its authorities, or concerning the delimitation of its frontiers or boundaries.

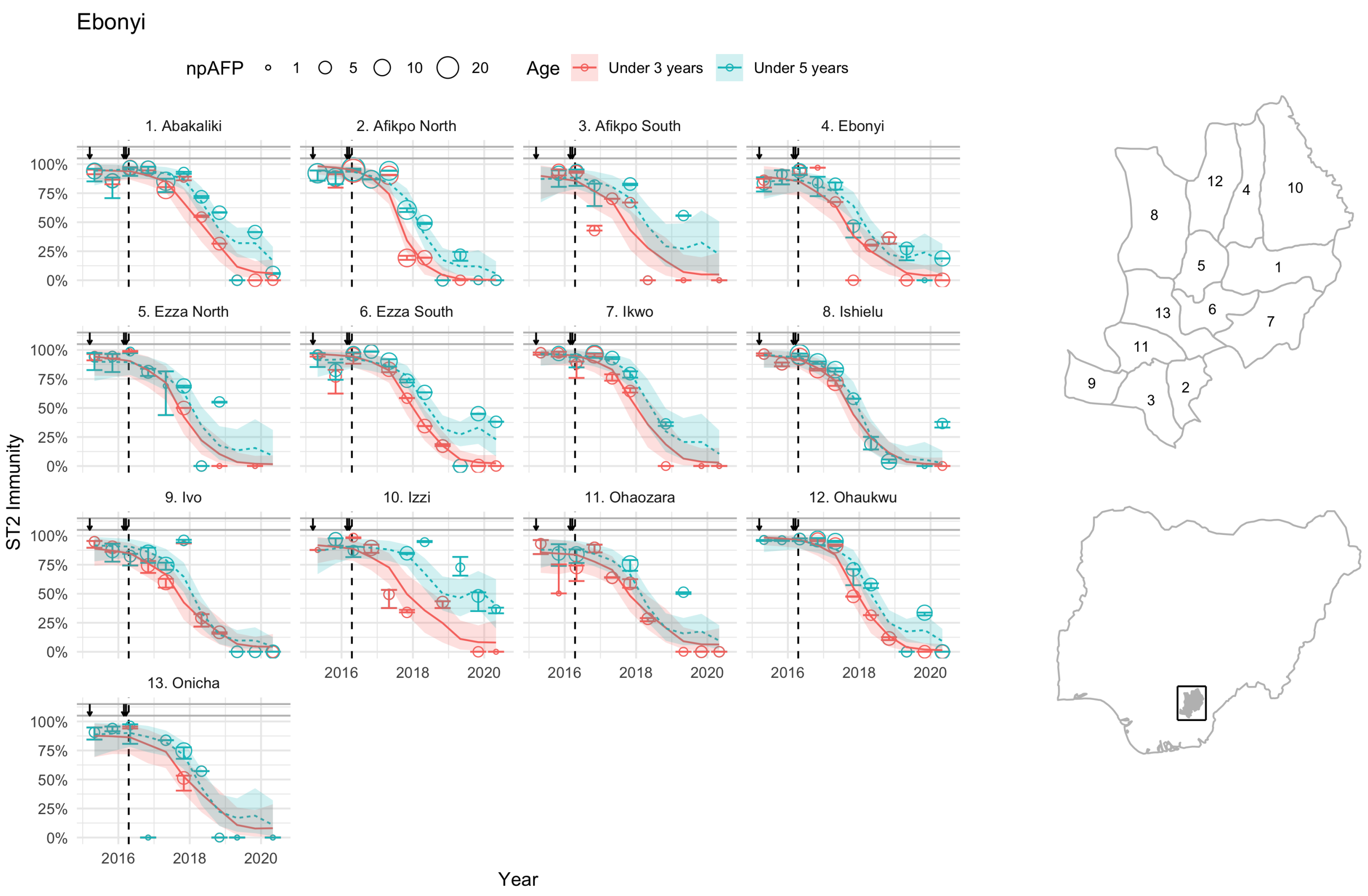

Figure S40: Type 2 population immunity from OPV in each district of Ebonyi, Nigeria in children under five (blue) and under three (red). Circles show median of bootstrapped crude immunity estimates, error bars show 2.5th and 97.5th percentiles of bootstrapped estimates. Size of circles indicate the number of non-polio AFP cases that each crude estimate is based on. Lines show median smoothed immunity estimate, transparent ribbons show 95% credible interval. Arrows show timing of tOPV (before withdrawal, dotted line) or mOPV2 SIAs (after withdrawal, dotted line). Height of arrows should the proportion of under-five population targeted in SIA. The publication of this map does not imply the expression of any opinion whatsoever on the part of WHO concerning the legal status of any territory, city or area or of its authorities, or concerning the delimitation of its frontiers or boundaries.

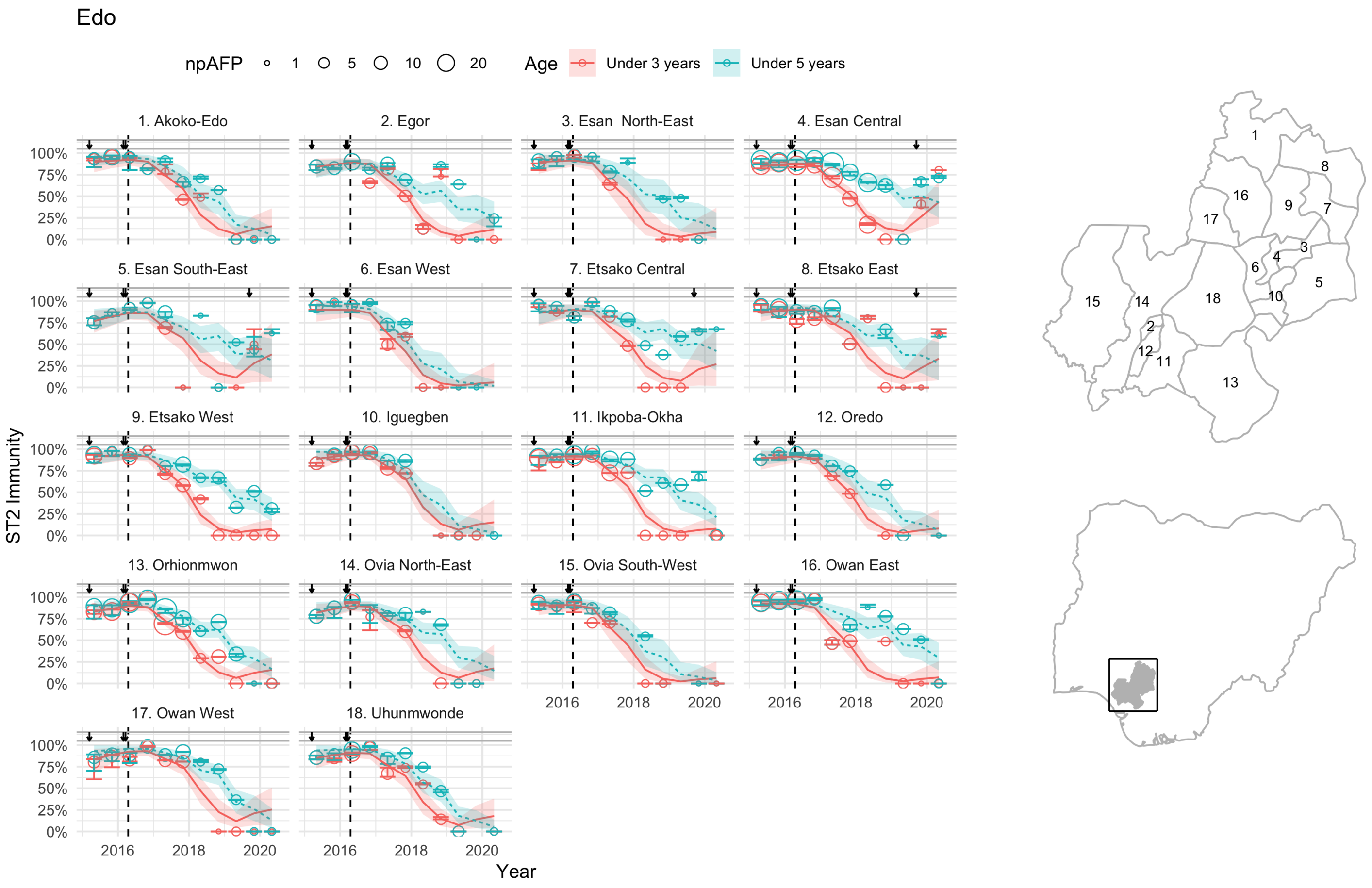

Figure S41: Type 2 population immunity from OPV in each district of Edo, Nigeria in children under five (blue) and under three (red). Circles show median of bootstrapped crude immunity estimates, error bars show 2.5th and 97.5th percentiles of bootstrapped estimates. Size of circles indicate the number of non-polio AFP cases that each crude estimate is based on. Lines show median smoothed immunity estimate, transparent ribbons show 95% credible interval. Arrows show timing of tOPV (before withdrawal, dotted line) or mOPV2 SIAs (after withdrawal, dotted line). Height of arrows should the proportion of under-five population targeted in SIA. The publication of this map does not imply the expression of any opinion whatsoever on the part of WHO concerning the legal status of any territory, city or area or of its authorities, or concerning the delimitation of its frontiers or boundaries.

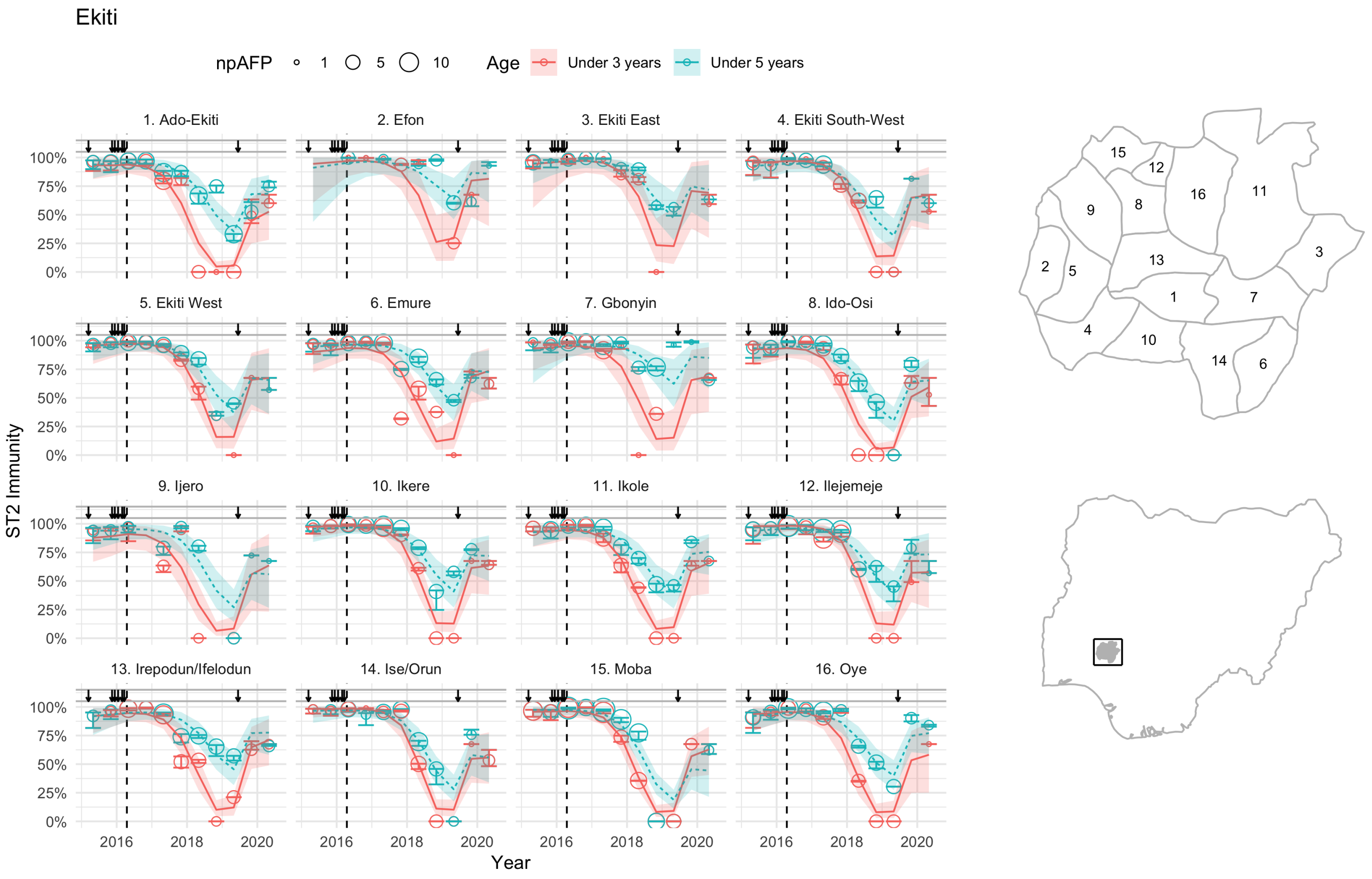

Figure S42: Type 2 population immunity from OPV in each district of Ekiti, Nigeria in children under five (blue) and under three (red). Circles show median of bootstrapped crude immunity estimates, error bars show 2.5th and 97.5th percentiles of bootstrapped estimates. Size of circles indicate the number of non-polio AFP cases that each crude estimate is based on. Lines show median smoothed immunity estimate, transparent ribbons show 95% credible interval. Arrows show timing of tOPV (before withdrawal, dotted line) or mOPV2 SIAs (after withdrawal, dotted line). Height of arrows should the proportion of under-five population targeted in SIA. The publication of this map does not imply the expression of any opinion whatsoever on the part of WHO concerning the legal status of any territory, city or area or of its authorities, or concerning the delimitation of its frontiers or boundaries.

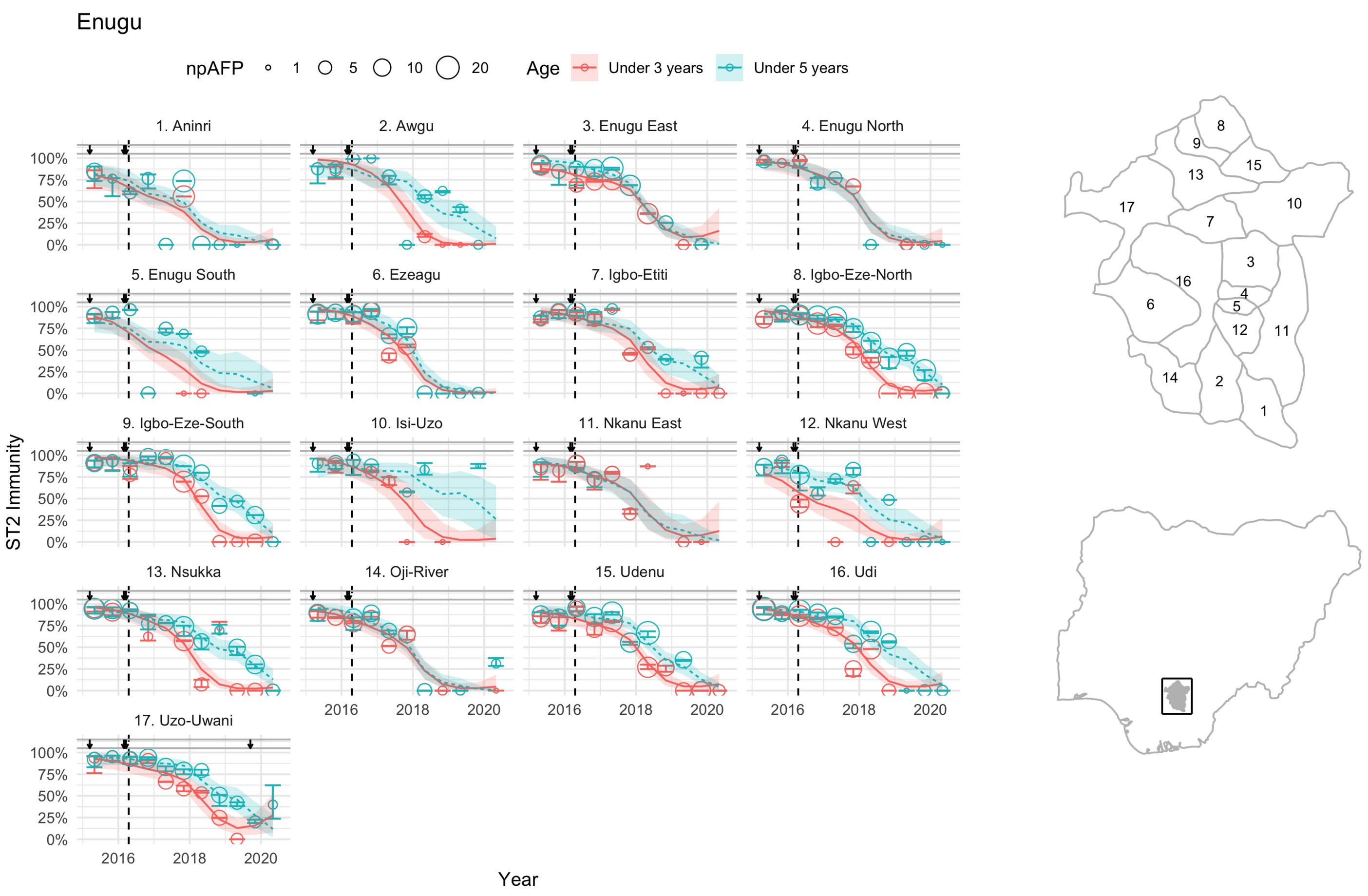

Figure S43: Type 2 population immunity from OPV in each district of Enugu, Nigeria in children under five (blue) and under three (red). Circles show median of bootstrapped crude immunity estimates, error bars show 2.5th and 97.5th percentiles of bootstrapped estimates. Size of circles indicate the number of non-polio AFP cases that each crude estimate is based on. Lines show median smoothed immunity estimate, transparent ribbons show 95% credible interval. Arrows show timing of tOPV (before withdrawal, dotted line) or mOPV2 SIAs (after withdrawal, dotted line). Height of arrows should the proportion of under-five population targeted in SIA. The publication of this map does not imply the expression of any opinion whatsoever on the part of WHO concerning the legal status of any territory, city or area or of its authorities, or concerning the delimitation of its frontiers or boundaries.

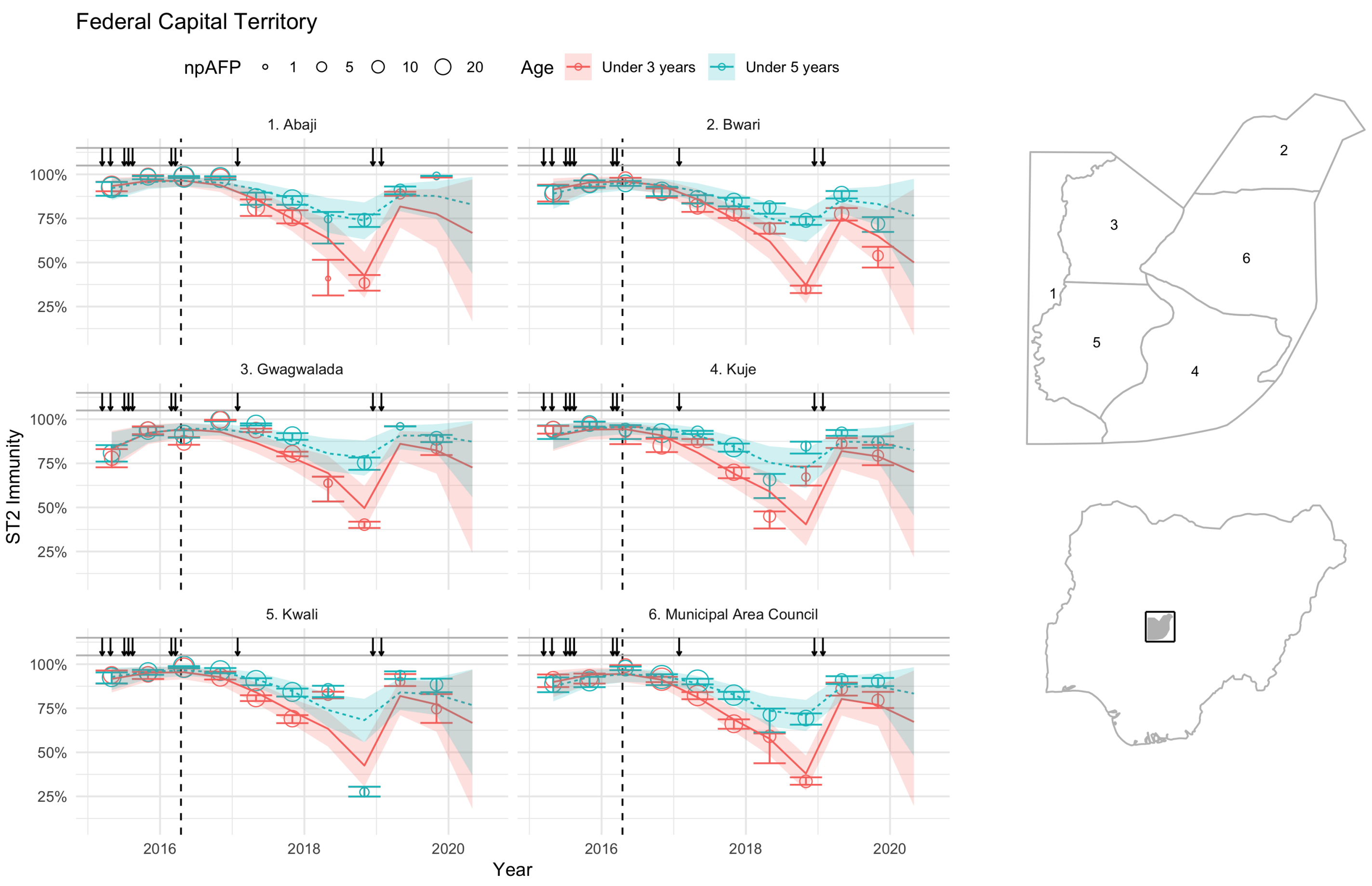

Figure S44: Type 2 population immunity from OPV in each district of Federal Capital Territory, Nigeria in children under five (blue) and under three (red). Circles show median of bootstrapped crude immunity estimates, error bars show 2.5th and 97.5th percentiles of bootstrapped estimates. Size of circles indicate the number of non-polio AFP cases that each crude estimate is based on. Lines show median smoothed immunity estimate, transparent ribbons show 95% credible interval. Arrows show timing of tOPV (before withdrawal, dotted line) or mOPV2 SIAs (after withdrawal, dotted line). Height of arrows should the proportion of under-five population targeted in SIA. The publication of this map does not imply the expression of any opinion whatsoever on the part of WHO concerning the legal status of any territory, city or area or of its authorities, or concerning the delimitation of its frontiers or boundaries.

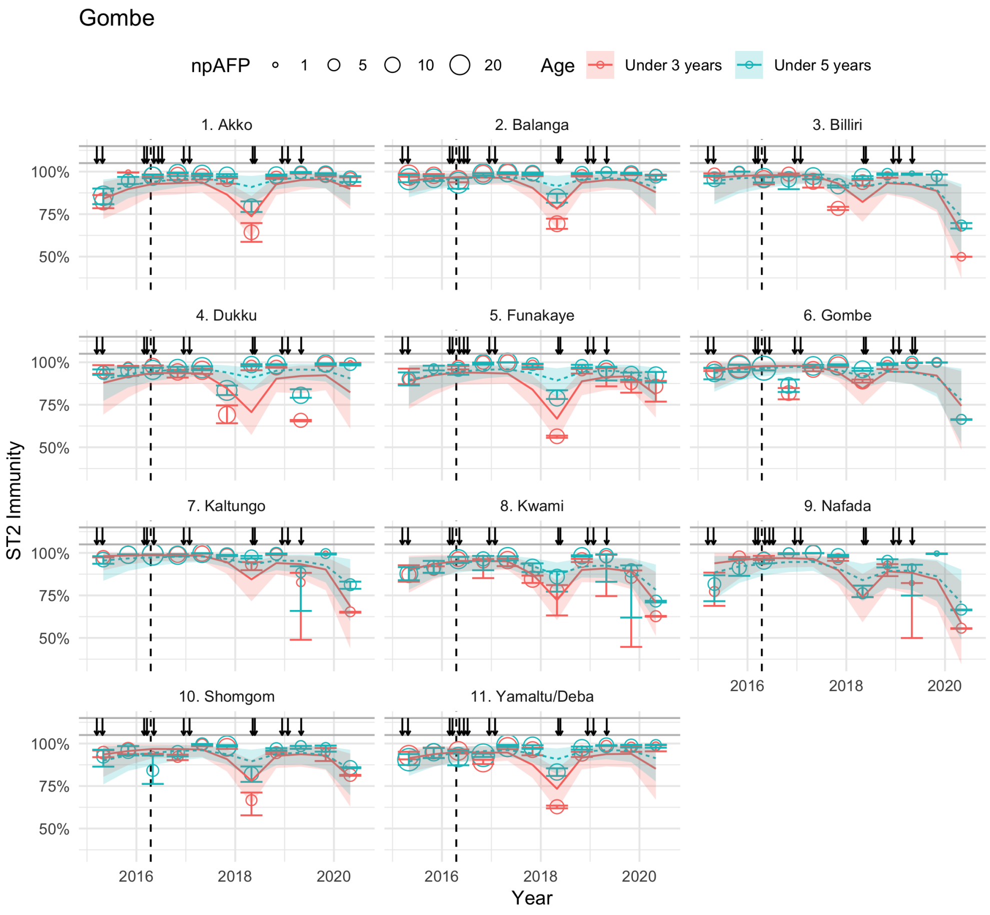

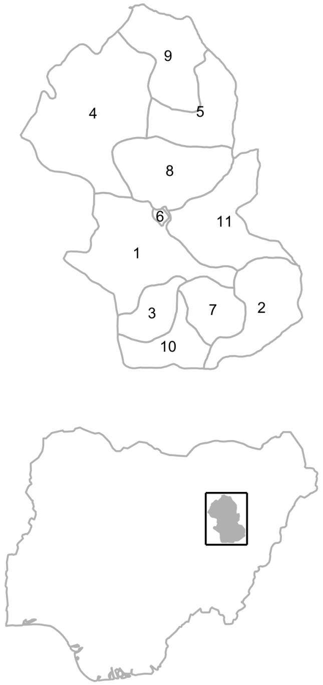

Figure S45: Type 2 population immunity from OPV in each district of Gombe, Nigeria in children under five (blue) and under three (red). Circles show median of bootstrapped crude immunity estimates, error bars show 2.5th and 97.5th percentiles of bootstrapped estimates. Size of circles indicate the number of non-polio AFP cases that each crude estimate is based on. Lines show median smoothed immunity estimate, transparent ribbons show 95% credible interval. Arrows show timing of tOPV (before withdrawal, dotted line) or mOPV2 SIAs (after withdrawal, dotted line). Height of arrows should the proportion of under-five population targeted in SIA. The publication of this map does not imply the expression of any opinion whatsoever on the part of WHO concerning the legal status of any territory, city or area or of its authorities, or concerning the delimitation of its frontiers or boundaries.

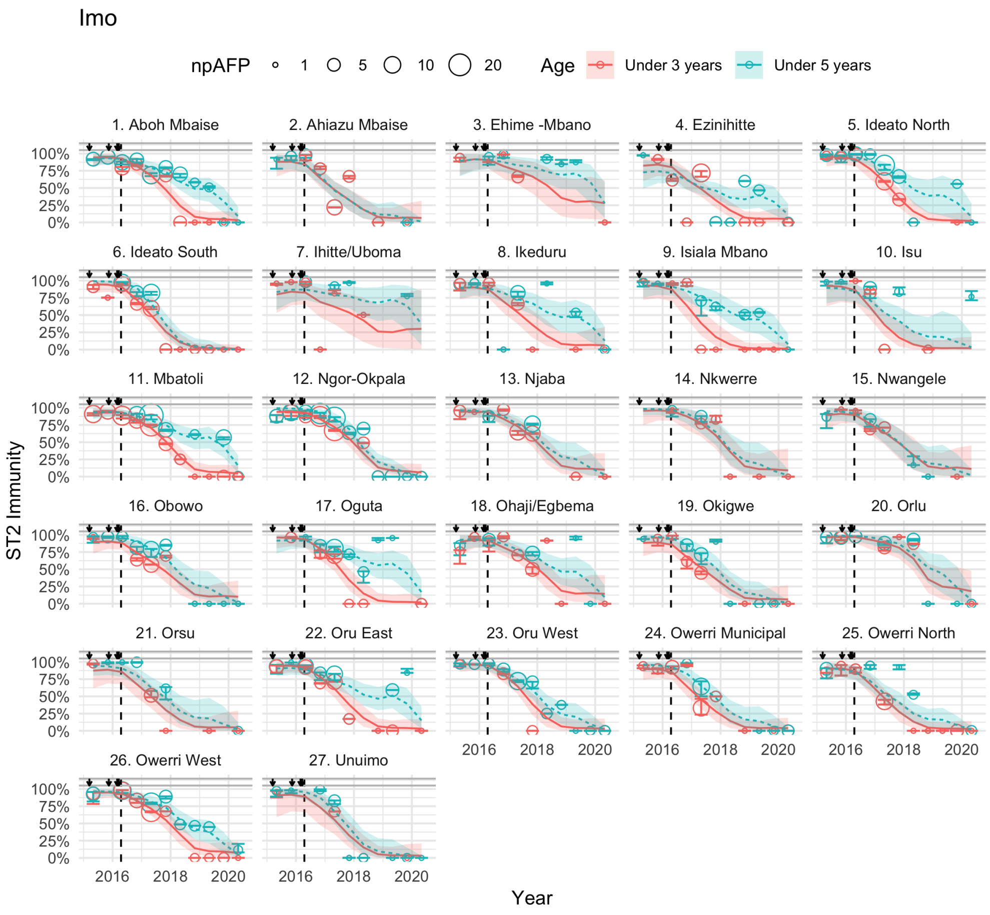

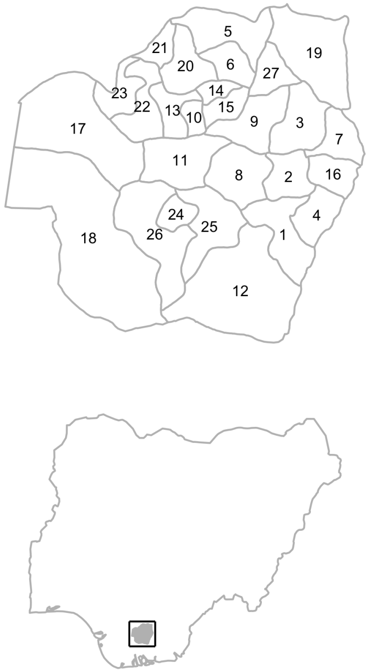

Figure S46: Type 2 population immunity from OPV in each district of Imo, Nigeria in children under five (blue) and under three (red). Circles show median of bootstrapped crude immunity estimates, error bars show 2.5th and 97.5th percentiles of bootstrapped estimates. Size of circles indicate the number of non-polio AFP cases that each crude estimate is based on. Lines show median smoothed immunity estimate, transparent ribbons show 95% credible interval. Arrows show timing of tOPV (before withdrawal, dotted line) or mOPV2 SIAs (after withdrawal, dotted line). Height of arrows should the proportion of under-five population targeted in SIA. The publication of this map does not imply the expression of any opinion whatsoever on the part of WHO concerning the legal status of any territory, city or area or of its authorities, or concerning the delimitation of its frontiers or boundaries.

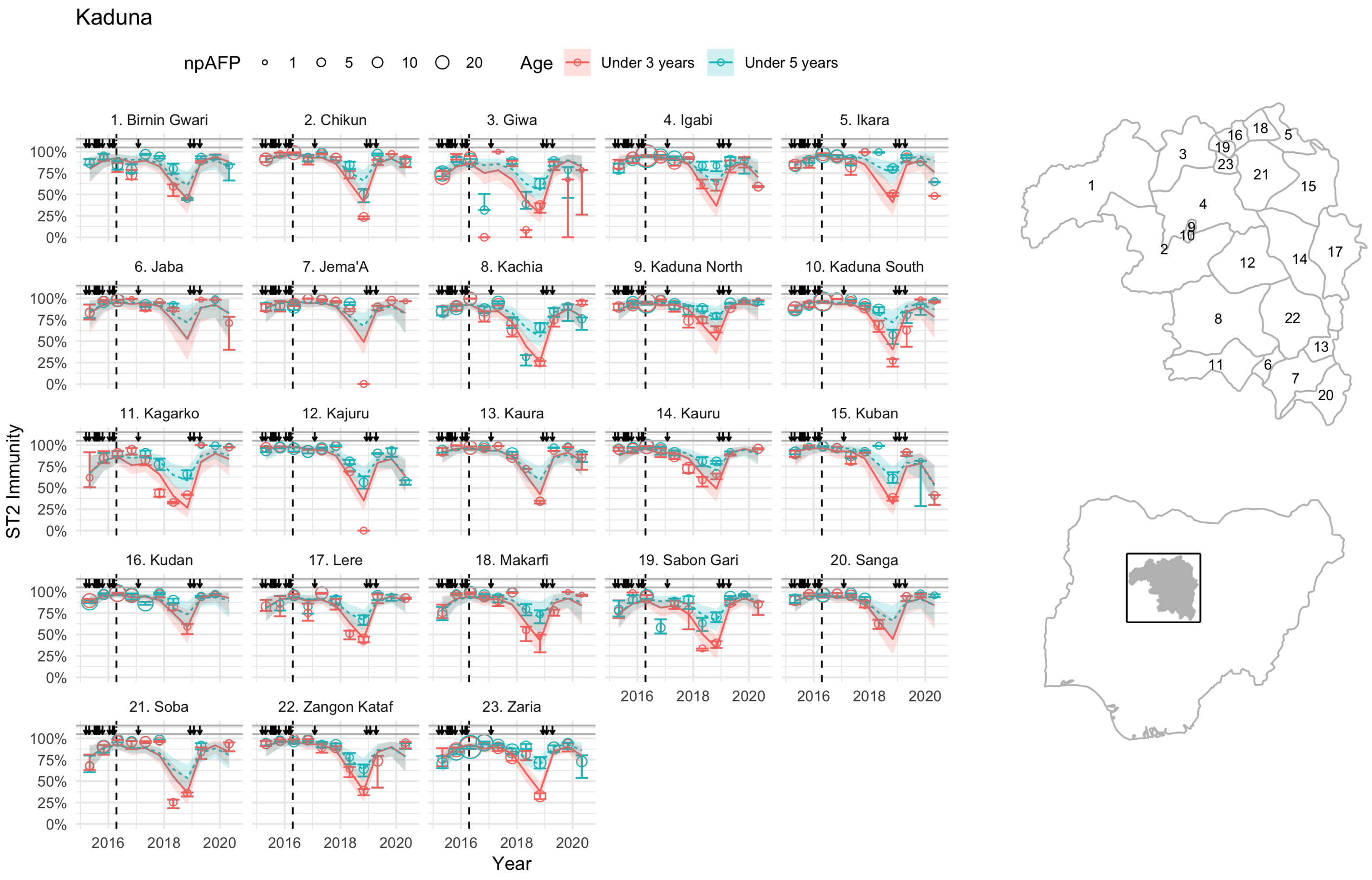

Figure S47: Type 2 population immunity from OPV in each district of Kaduna, Nigeria in children under five (blue) and under three (red). Circles show median of bootstrapped crude immunity estimates, error bars show 2.5th and 97.5th percentiles of bootstrapped estimates. Size of circles indicate the number of non-polio AFP cases that each crude estimate is based on. Lines show median smoothed immunity estimate, transparent ribbons show 95% credible interval. Arrows show timing of tOPV (before withdrawal, dotted line) or mOPV2 SIAs (after withdrawal, dotted line). Height of arrows should the proportion of under-five population targeted in SIA. The publication of this map does not imply the expression of any opinion whatsoever on the part of WHO concerning the legal status of any territory, city or area or of its authorities, or concerning the delimitation of its frontiers or boundaries.

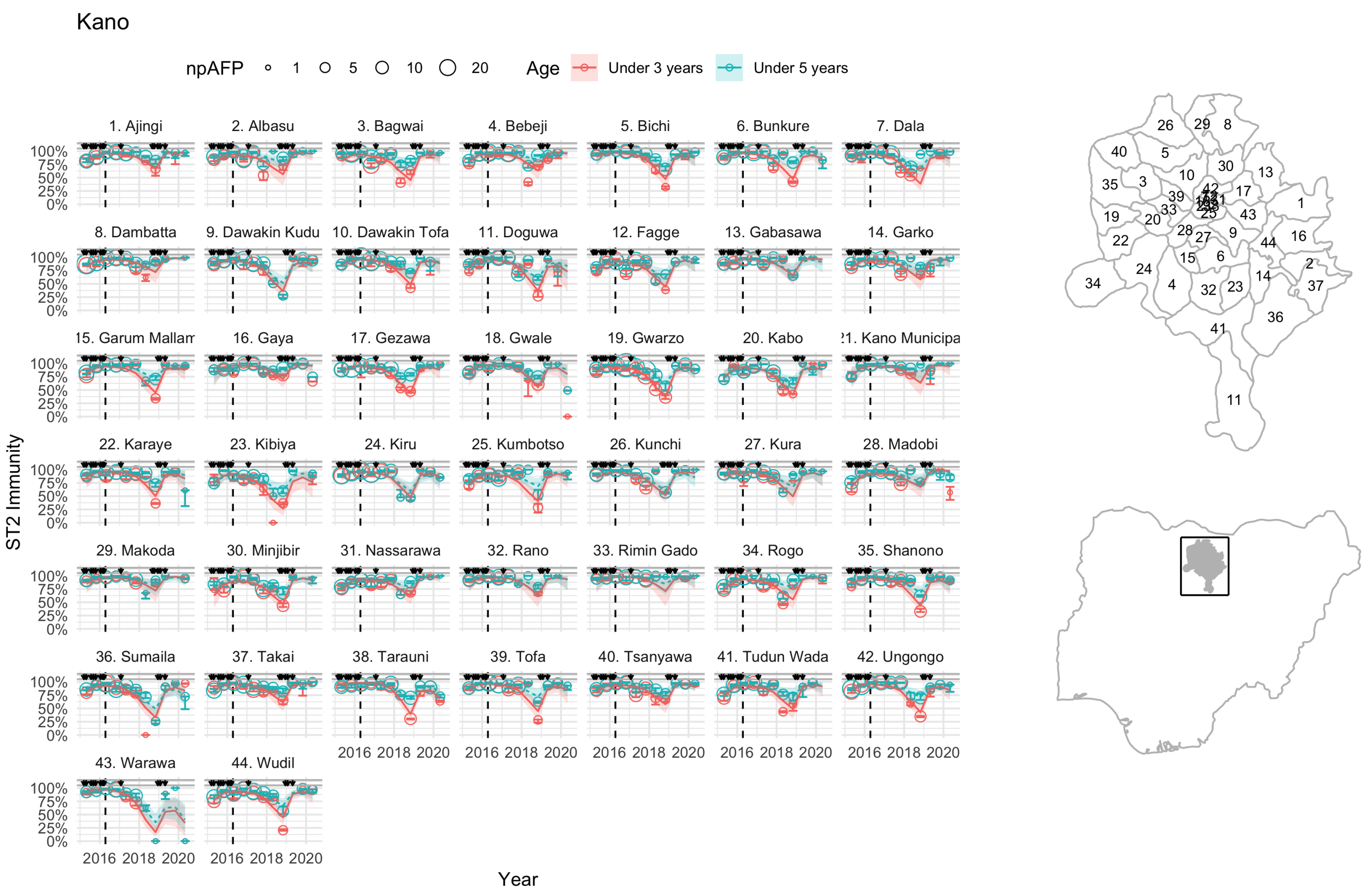

Figure S48: Type 2 population immunity from OPV in each district of Kano, Nigeria in children under five (blue) and under three (red). Circles show median of bootstrapped crude immunity estimates, error bars show 2.5th and 97.5th percentiles of bootstrapped estimates. Size of circles indicate the number of non-polio AFP cases that each crude estimate is based on. Lines show median smoothed immunity estimate, transparent ribbons show 95% credible interval. Arrows show timing of tOPV (before withdrawal, dotted line) or mOPV2 SIAs (after withdrawal, dotted line). Height of arrows should the proportion of under-five population targeted in SIA. The publication of this map does not imply the expression of any opinion whatsoever on the part of WHO concerning the legal status of any territory, city or area or of its authorities, or concerning the delimitation of its frontiers or boundaries.

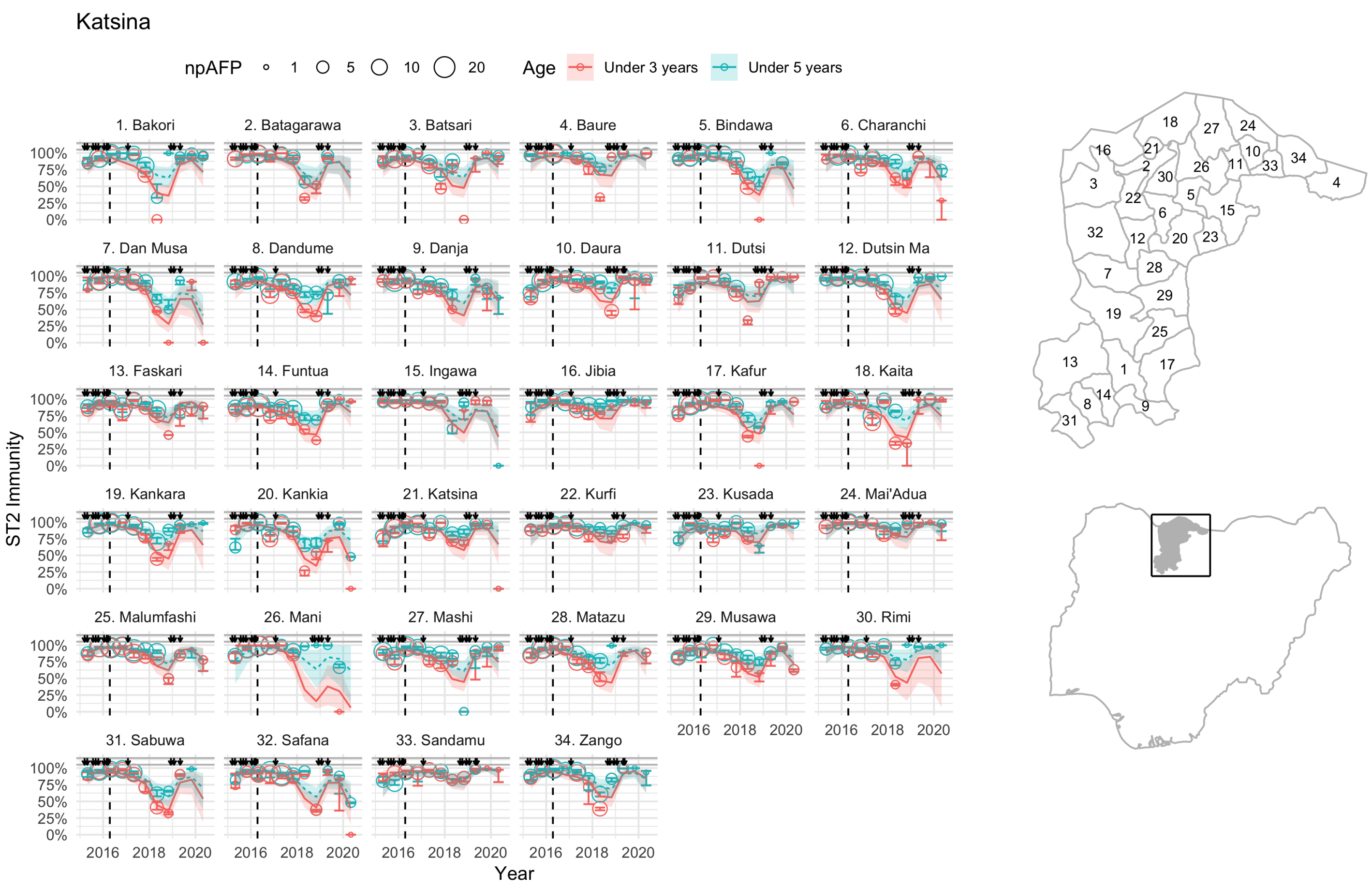

Figure S49: Type 2 population immunity from OPV in each district of Katsina, Nigeria in children under five (blue) and under three (red). Circles show median of bootstrapped crude immunity estimates, error bars show 2.5th and 97.5th percentiles of bootstrapped estimates. Size of circles indicate the number of non-polio AFP cases that each crude estimate is based on. Lines show median smoothed immunity estimate, transparent ribbons show 95% credible interval. Arrows show timing of tOPV (before withdrawal, dotted line) or mOPV2 SIAs (after withdrawal, dotted line). Height of arrows should the proportion of under-five population targeted in SIA. The publication of this map does not imply the expression of any opinion whatsoever on the part of WHO concerning the legal status of any territory, city or area or of its authorities, or concerning the delimitation of its frontiers or boundaries.

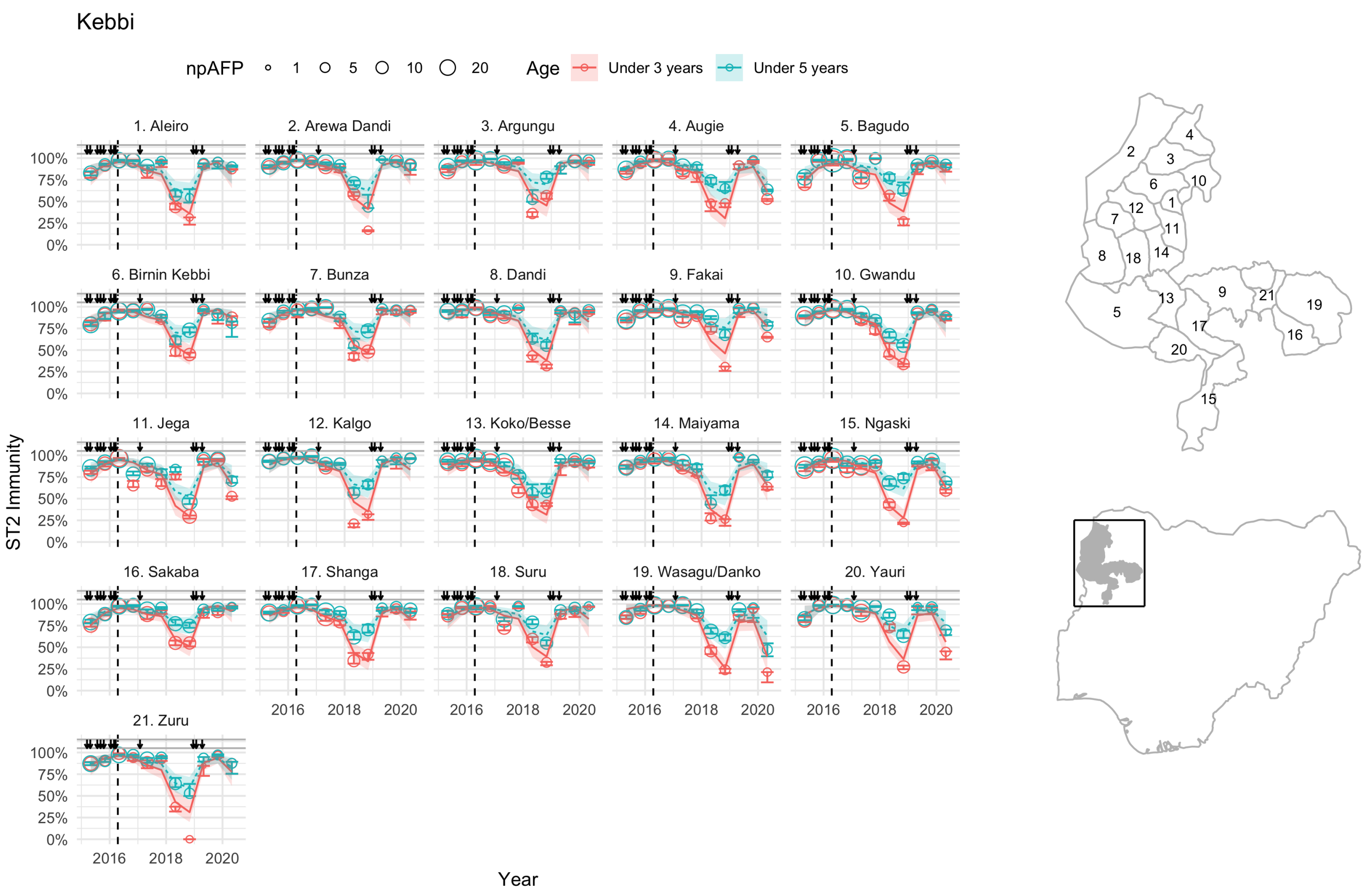

Figure S50: Type 2 population immunity from OPV in each district of Kebbi, Nigeria in children under five (blue) and under three (red). Circles show median of bootstrapped crude immunity estimates, error bars show 2.5th and 97.5th percentiles of bootstrapped estimates. Size of circles indicate the number of non-polio AFP cases that each crude estimate is based on. Lines show median smoothed immunity estimate, transparent ribbons show 95% credible interval. Arrows show timing of tOPV (before withdrawal, dotted line) or mOPV2 SIAs (after withdrawal, dotted line). Height of arrows should the proportion of under-five population targeted in SIA. The publication of this map does not imply the expression of any opinion whatsoever on the part of WHO concerning the legal status of any territory, city or area or of its authorities, or concerning the delimitation of its frontiers or boundaries.

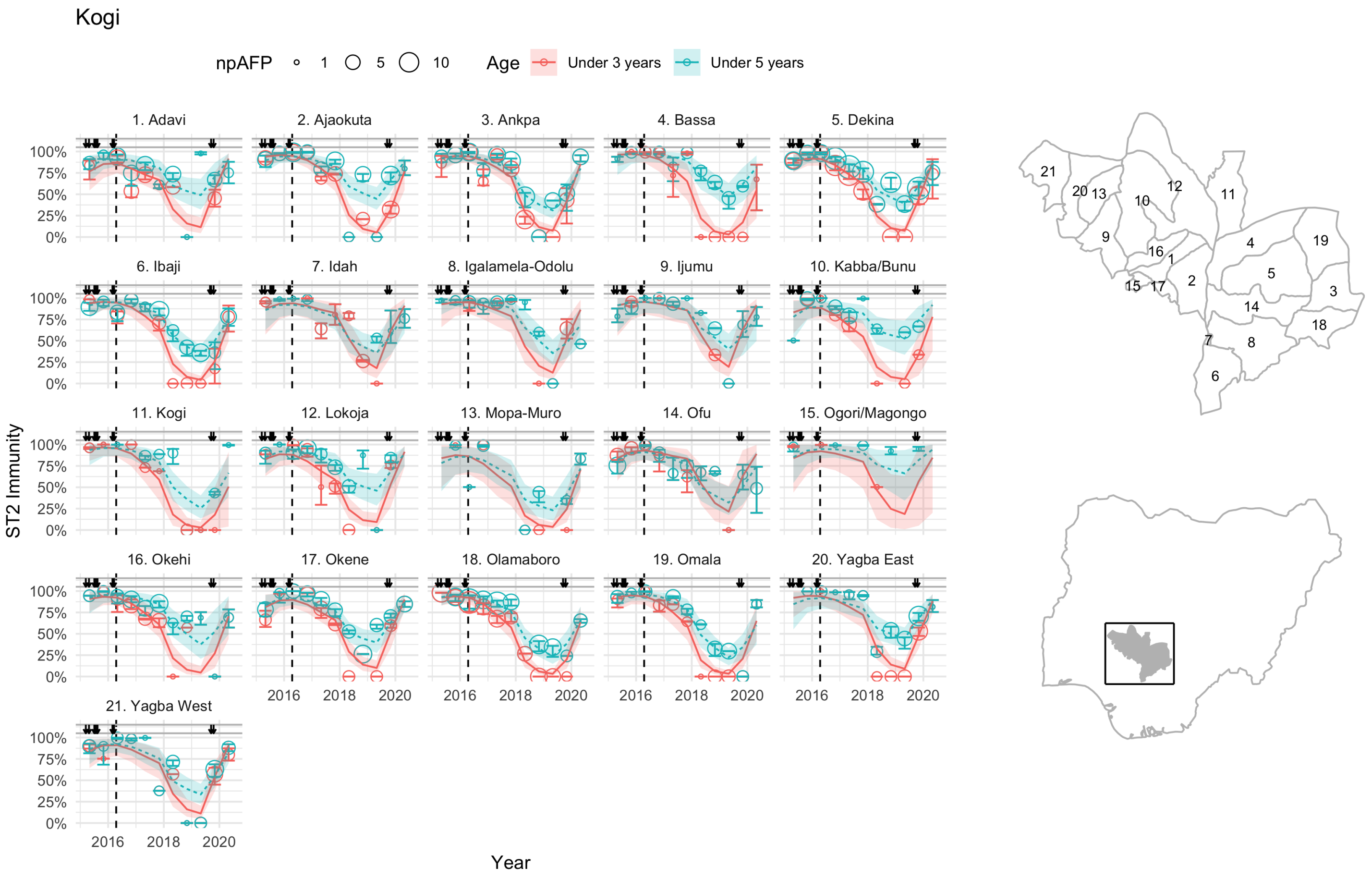

Figure S51: Type 2 population immunity from OPV in each district of Kogi, Nigeria in children under five (blue) and under three (red). Circles show median of bootstrapped crude immunity estimates, error bars show 2.5th and 97.5th percentiles of bootstrapped estimates. Size of circles indicate the number of non-polio AFP cases that each crude estimate is based on. Lines show median smoothed immunity estimate, transparent ribbons show 95% credible interval. Arrows show timing of tOPV (before withdrawal, dotted line) or mOPV2 SIAs (after withdrawal, dotted line). Height of arrows should the proportion of under-five population targeted in SIA. The publication of this map does not imply the expression of any opinion whatsoever on the part of WHO concerning the legal status of any territory, city or area or of its authorities, or concerning the delimitation of its frontiers or boundaries.

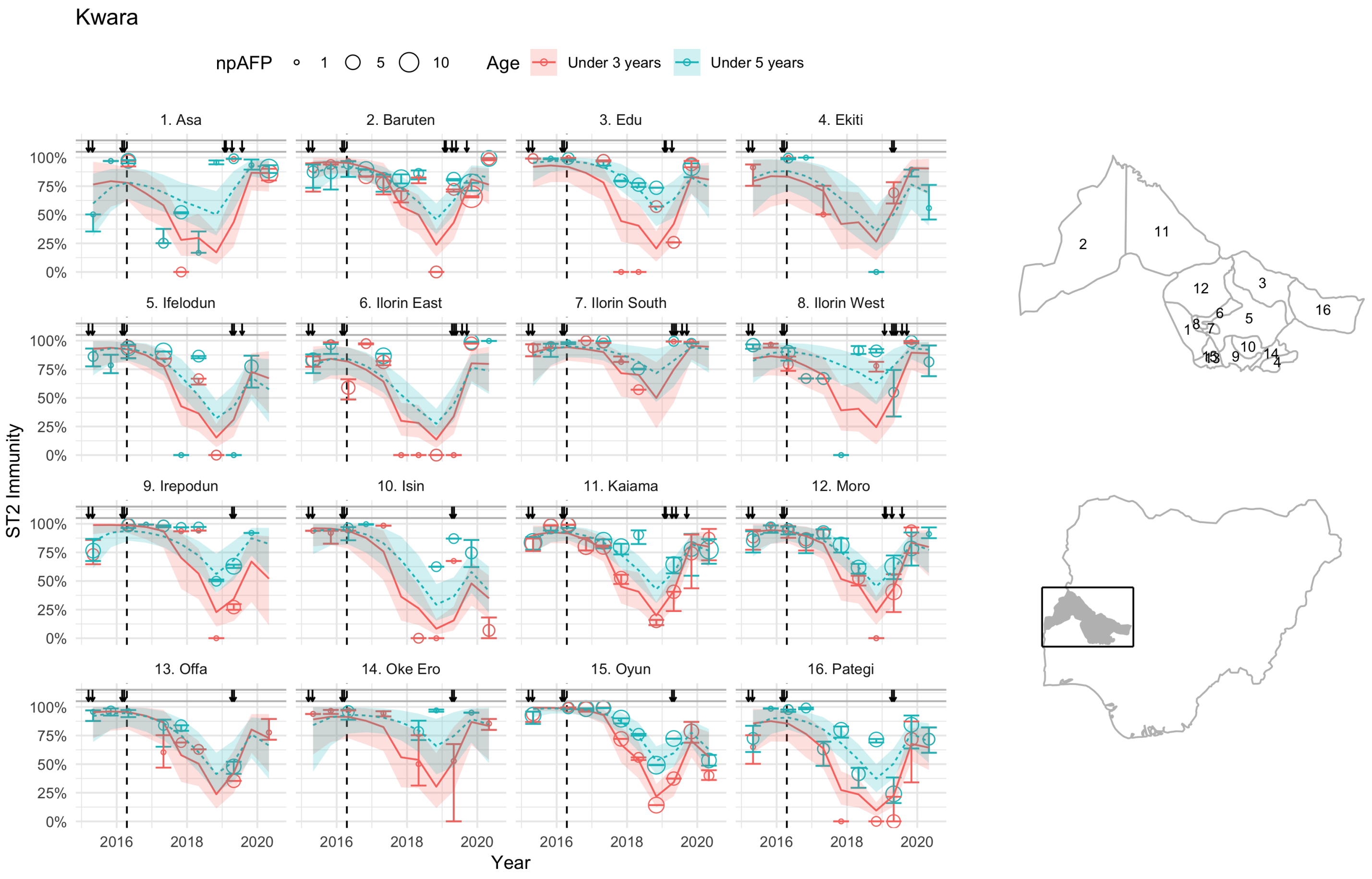

Figure S52: Type 2 population immunity from OPV in each district of Kwara, Nigeria in children under five (blue) and under three (red). Circles show median of bootstrapped crude immunity estimates, error bars show 2.5th and 97.5th percentiles of bootstrapped estimates. Size of circles indicate the number of non-polio AFP cases that each crude estimate is based on. Lines show median smoothed immunity estimate, transparent ribbons show 95% credible interval. Arrows show timing of tOPV (before withdrawal, dotted line) or mOPV2 SIAs (after withdrawal, dotted line). Height of arrows should the proportion of under-five population targeted in SIA. The publication of this map does not imply the expression of any opinion whatsoever on the part of WHO concerning the legal status of any territory, city or area or of its authorities, or concerning the delimitation of its frontiers or boundaries.

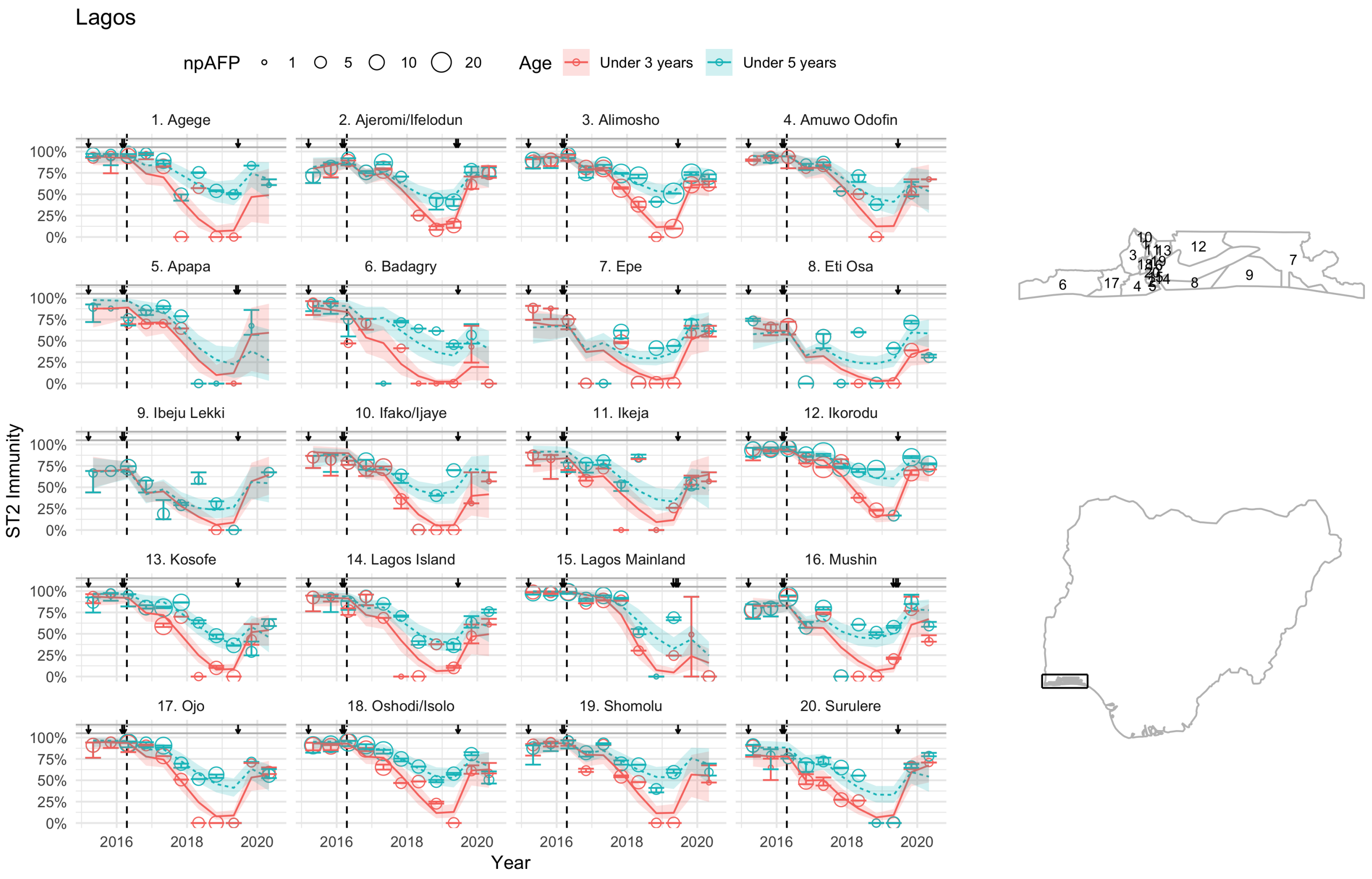

Figure S53: Type 2 population immunity from OPV in each district of Lagos, Nigeria in children under five (blue) and under three (red). Circles show median of bootstrapped crude immunity estimates, error bars show 2.5th and 97.5th percentiles of bootstrapped estimates. Size of circles indicate the number of non-polio AFP cases that each crude estimate is based on. Lines show median smoothed immunity estimate, transparent ribbons show 95% credible interval. Arrows show timing of tOPV (before withdrawal, dotted line) or mOPV2 SIAs (after withdrawal, dotted line). Height of arrows should the proportion of under-five population targeted in SIA. The publication of this map does not imply the expression of any opinion whatsoever on the part of WHO concerning the legal status of any territory, city or area or of its authorities, or concerning the delimitation of its frontiers or boundaries.

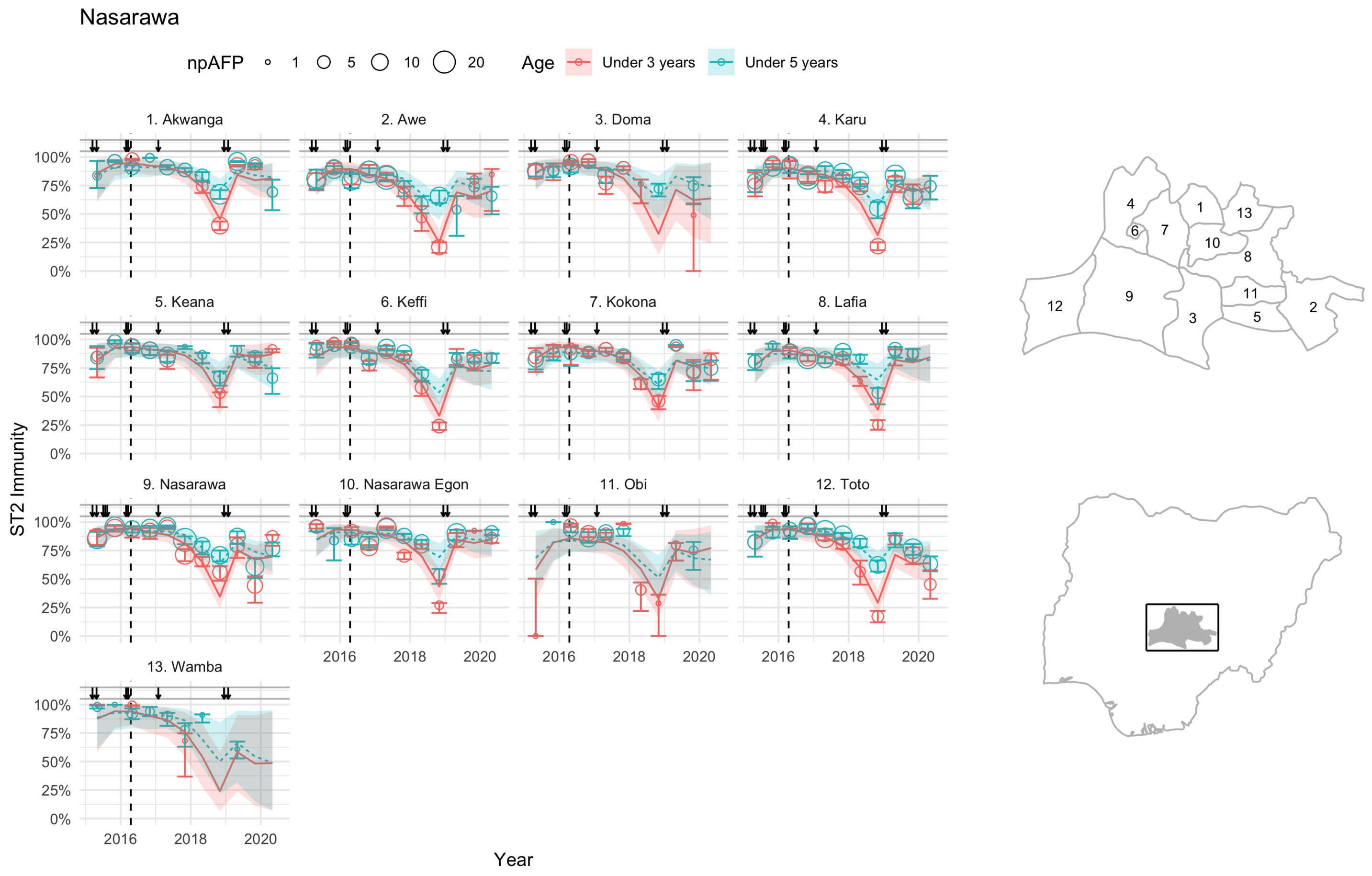

Figure S54: Type 2 population immunity from OPV in each district of Nasawara, Nigeria in children under five (blue) and under three (red). Circles show median of bootstrapped crude immunity estimates, error bars show 2.5th and 97.5th percentiles of bootstrapped estimates. Size of circles indicate the number of non-polio AFP cases that each crude estimate is based on. Lines show median smoothed immunity estimate, transparent ribbons show 95% credible interval. Arrows show timing of tOPV (before withdrawal, dotted line) or mOPV2 SIAs (after withdrawal, dotted line). Height of arrows should the proportion of under-five population targeted in SIA. The publication of this map does not imply the expression of any opinion whatsoever on the part of WHO concerning the legal status of any territory, city or area or of its authorities, or concerning the delimitation of its frontiers or boundaries.

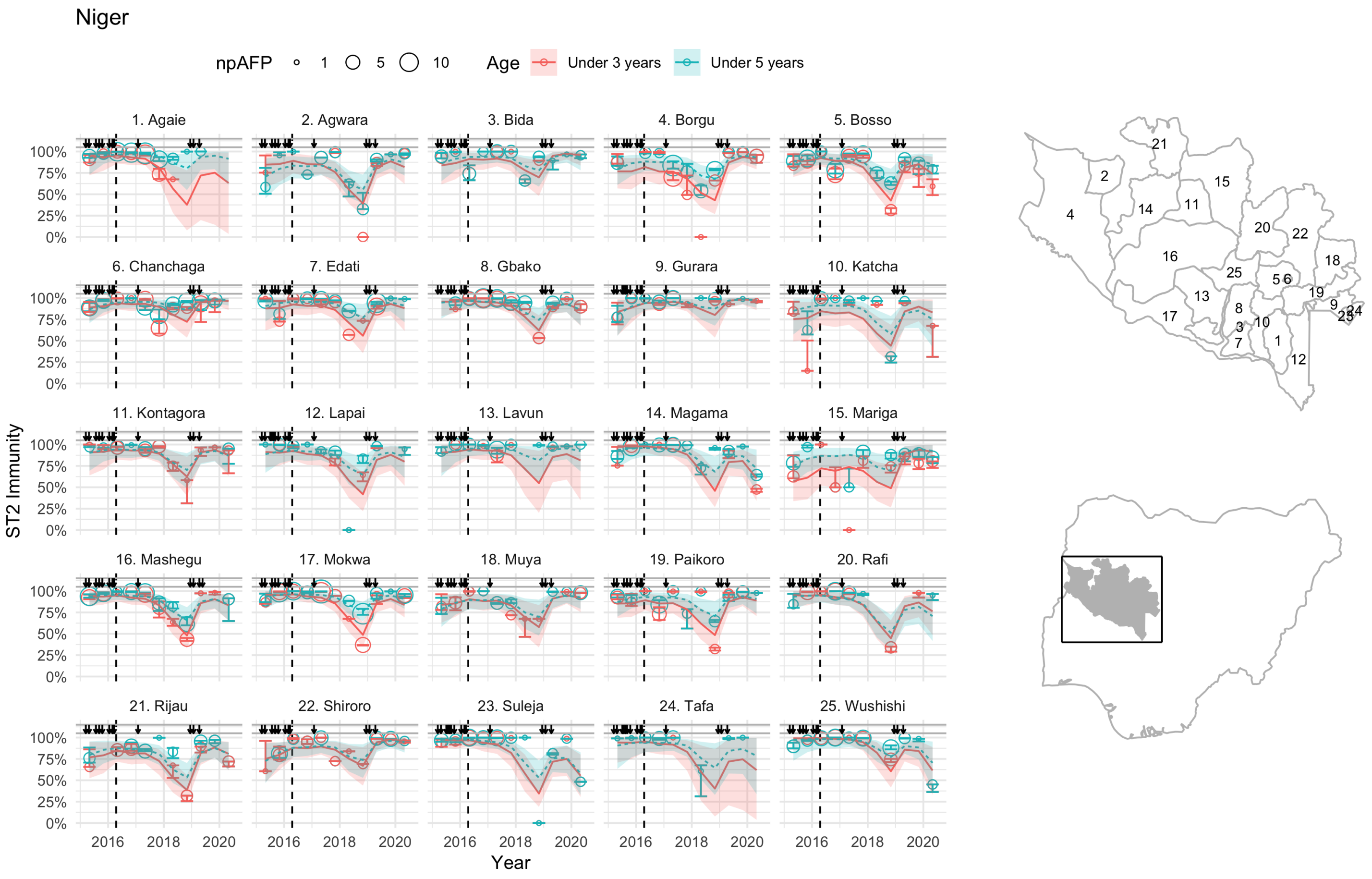

Figure S55: Type 2 population immunity from OPV in each district of Niger, Nigeria in children under five (blue) and under three (red). Circles show median of bootstrapped crude immunity estimates, error bars show 2.5th and 97.5th percentiles of bootstrapped estimates. Size of circles indicate the number of non-polio AFP cases that each crude estimate is based on. Lines show median smoothed immunity estimate, transparent ribbons show 95% credible interval. Arrows show timing of tOPV (before withdrawal, dotted line) or mOPV2 SIAs (after withdrawal, dotted line). Height of arrows should the proportion of under-five population targeted in SIA. The publication of this map does not imply the expression of any opinion whatsoever on the part of WHO concerning the legal status of any territory, city or area or of its authorities, or concerning the delimitation of its frontiers or boundaries.

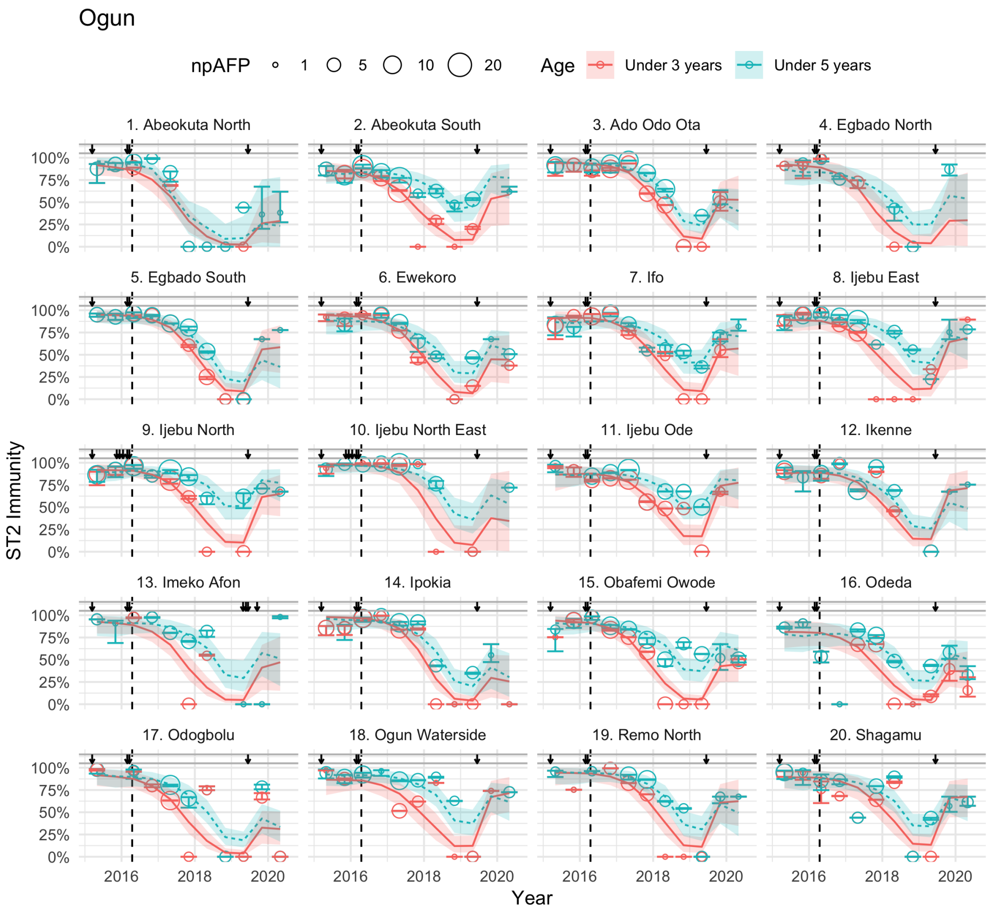

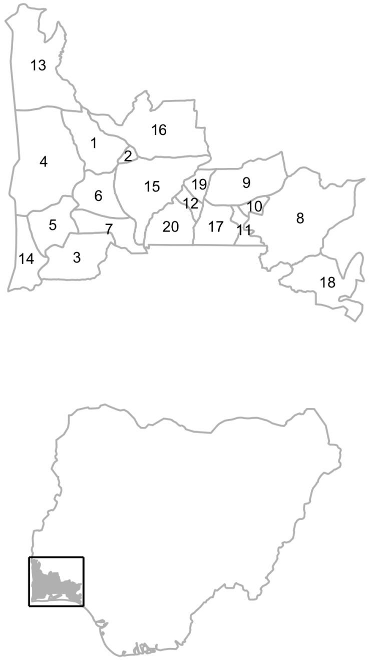

Figure S56: Type 2 population immunity from OPV in each district of Ogun, Nigeria in children under five (blue) and under three (red). Circles show median of bootstrapped crude immunity estimates, error bars show 2.5th and 97.5th percentiles of bootstrapped estimates. Size of circles indicate the number of non-polio AFP cases that each crude estimate is based on. Lines show median smoothed immunity estimate, transparent ribbons show 95% credible interval. Arrows show timing of tOPV (before withdrawal, dotted line) or mOPV2 SIAs (after withdrawal, dotted line). Height of arrows should the proportion of under-five population targeted in SIA. The publication of this map does not imply the expression of any opinion whatsoever on the part of WHO concerning the legal status of any territory, city or area or of its authorities, or concerning the delimitation of its frontiers or boundaries.

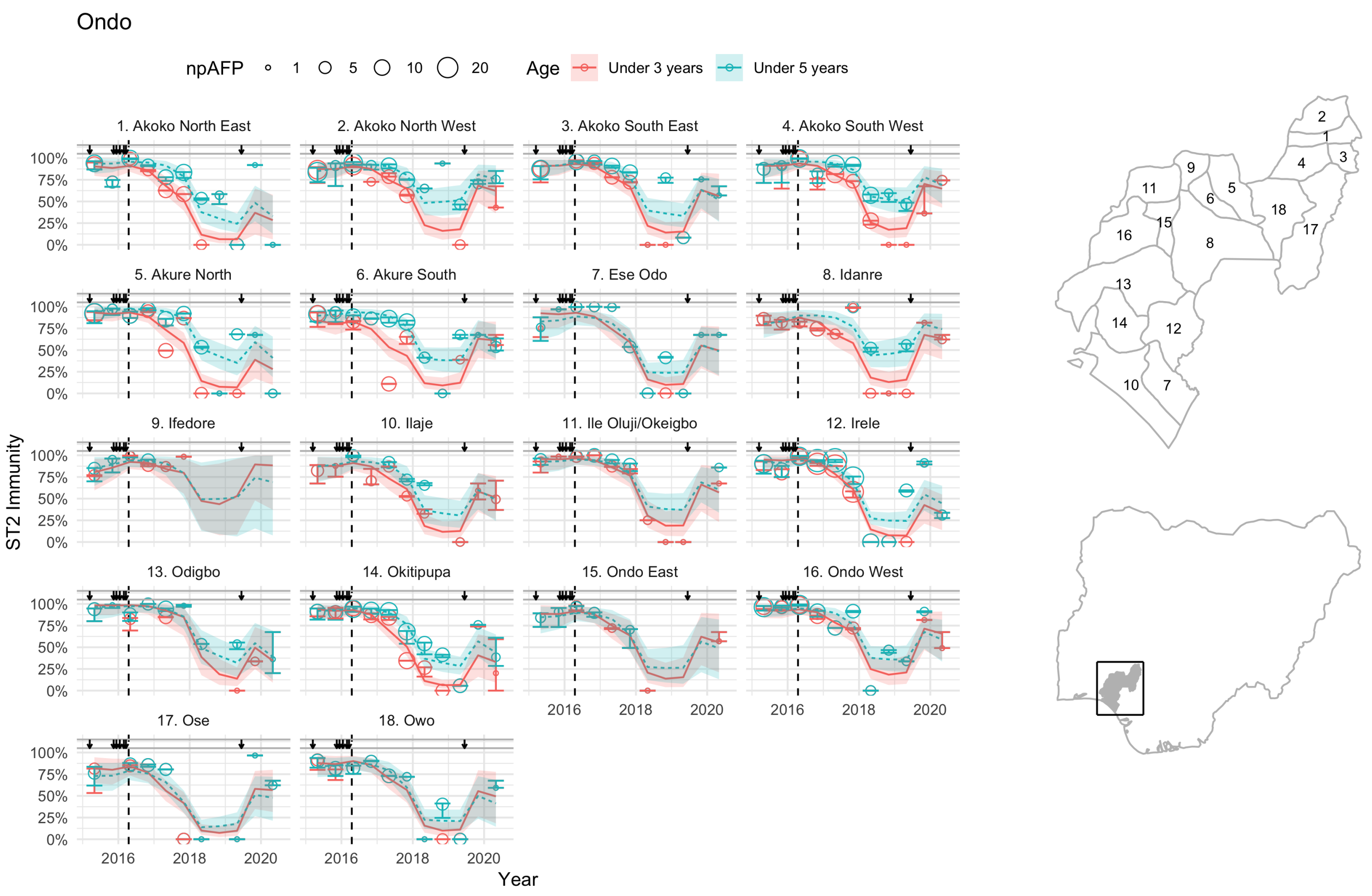

Figure S57: Type 2 population immunity from OPV in each district of Ondo, Nigeria in children under five (blue) and under three (red). Circles show median of bootstrapped crude immunity estimates, error bars show 2.5th and 97.5th percentiles of bootstrapped estimates. Size of circles indicate the number of non-polio AFP cases that each crude estimate is based on. Lines show median smoothed immunity estimate, transparent ribbons show 95% credible interval. Arrows show timing of tOPV (before withdrawal, dotted line) or mOPV2 SIAs (after withdrawal, dotted line). Height of arrows should the proportion of under-five population targeted in SIA. The publication of this map does not imply the expression of any opinion whatsoever on the part of WHO concerning the legal status of any territory, city or area or of its authorities, or concerning the delimitation of its frontiers or boundaries.

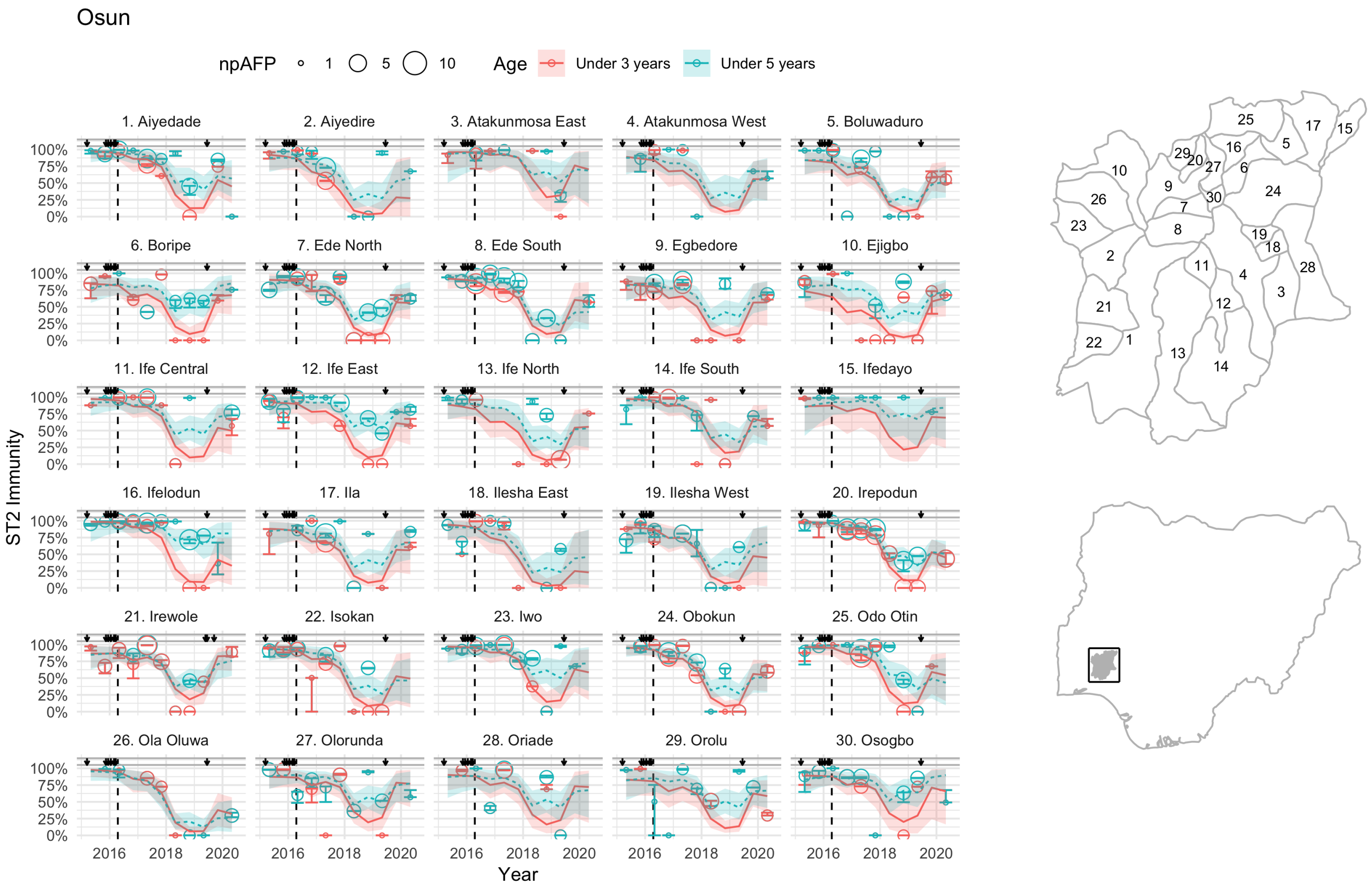

Figure S58: Type 2 population immunity from OPV in each district of Osun, Nigeria in children under five (blue) and under three (red). Circles show median of bootstrapped crude immunity estimates, error bars show 2.5th and 97.5th percentiles of bootstrapped estimates. Size of circles indicate the number of non-polio AFP cases that each crude estimate is based on. Lines show median smoothed immunity estimate, transparent ribbons show 95% credible interval. Arrows show timing of tOPV (before withdrawal, dotted line) or mOPV2 SIAs (after withdrawal, dotted line). Height of arrows should the proportion of under-five population targeted in SIA. The publication of this map does not imply the expression of any opinion whatsoever on the part of WHO concerning the legal status of any territory, city or area or of its authorities, or concerning the delimitation of its frontiers or boundaries.

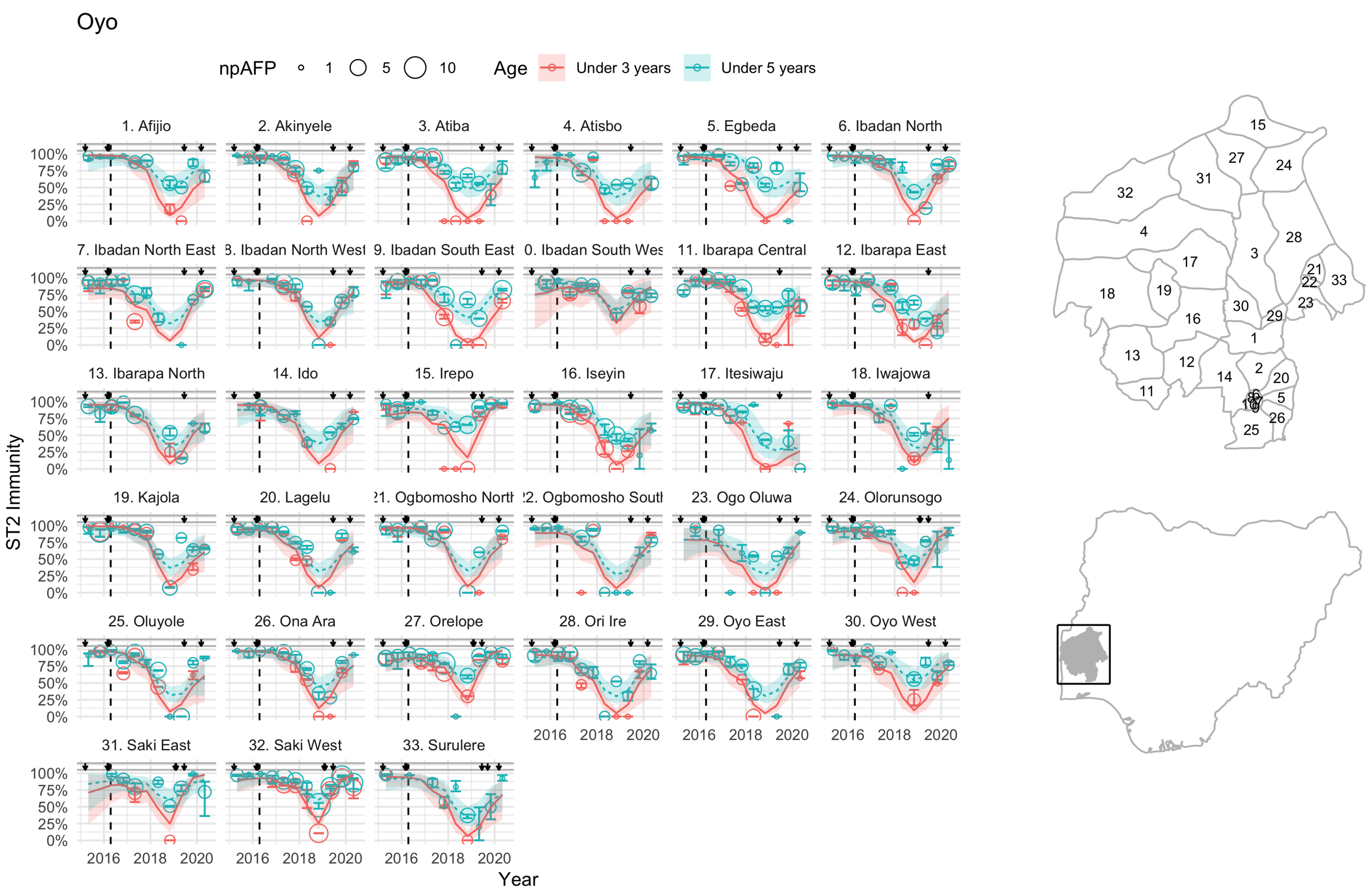

Figure S59: Type 2 population immunity from OPV in each district of Oyo, Nigeria in children under five (blue) and under three (red). Circles show median of bootstrapped crude immunity estimates, error bars show 2.5th and 97.5th percentiles of bootstrapped estimates. Size of circles indicate the number of non-polio AFP cases that each crude estimate is based on. Lines show median smoothed immunity estimate, transparent ribbons show 95% credible interval. Arrows show timing of tOPV (before withdrawal, dotted line) or mOPV2 SIAs (after withdrawal, dotted line). Height of arrows should the proportion of under-five population targeted in SIA. The publication of this map does not imply the expression of any opinion whatsoever on the part of WHO concerning the legal status of any territory, city or area or of its authorities, or concerning the delimitation of its frontiers or boundaries.

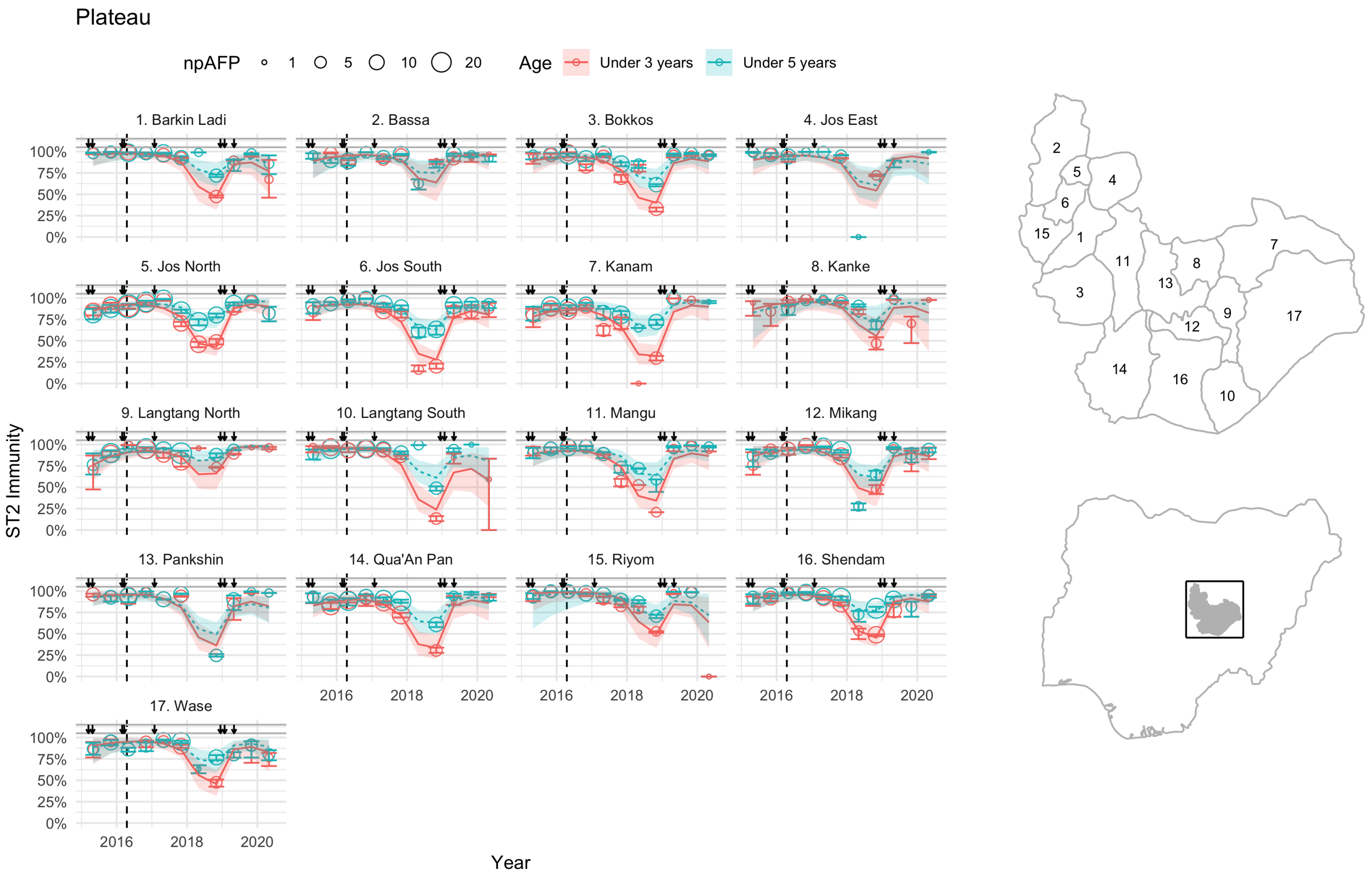

Figure S60: Type 2 population immunity from OPV in each district of Plateau, Nigeria in children under five (blue) and under three (red). Circles show median of bootstrapped crude immunity estimates, error bars show 2.5th and 97.5th percentiles of bootstrapped estimates. Size of circles indicate the number of non-polio AFP cases that each crude estimate is based on. Lines show median smoothed immunity estimate, transparent ribbons show 95% credible interval. Arrows show timing of tOPV (before withdrawal, dotted line) or mOPV2 SIAs (after withdrawal, dotted line). Height of arrows should the proportion of under-five population targeted in SIA. The publication of this map does not imply the expression of any opinion whatsoever on the part of WHO concerning the legal status of any territory, city or area or of its authorities, or concerning the delimitation of its frontiers or boundaries.

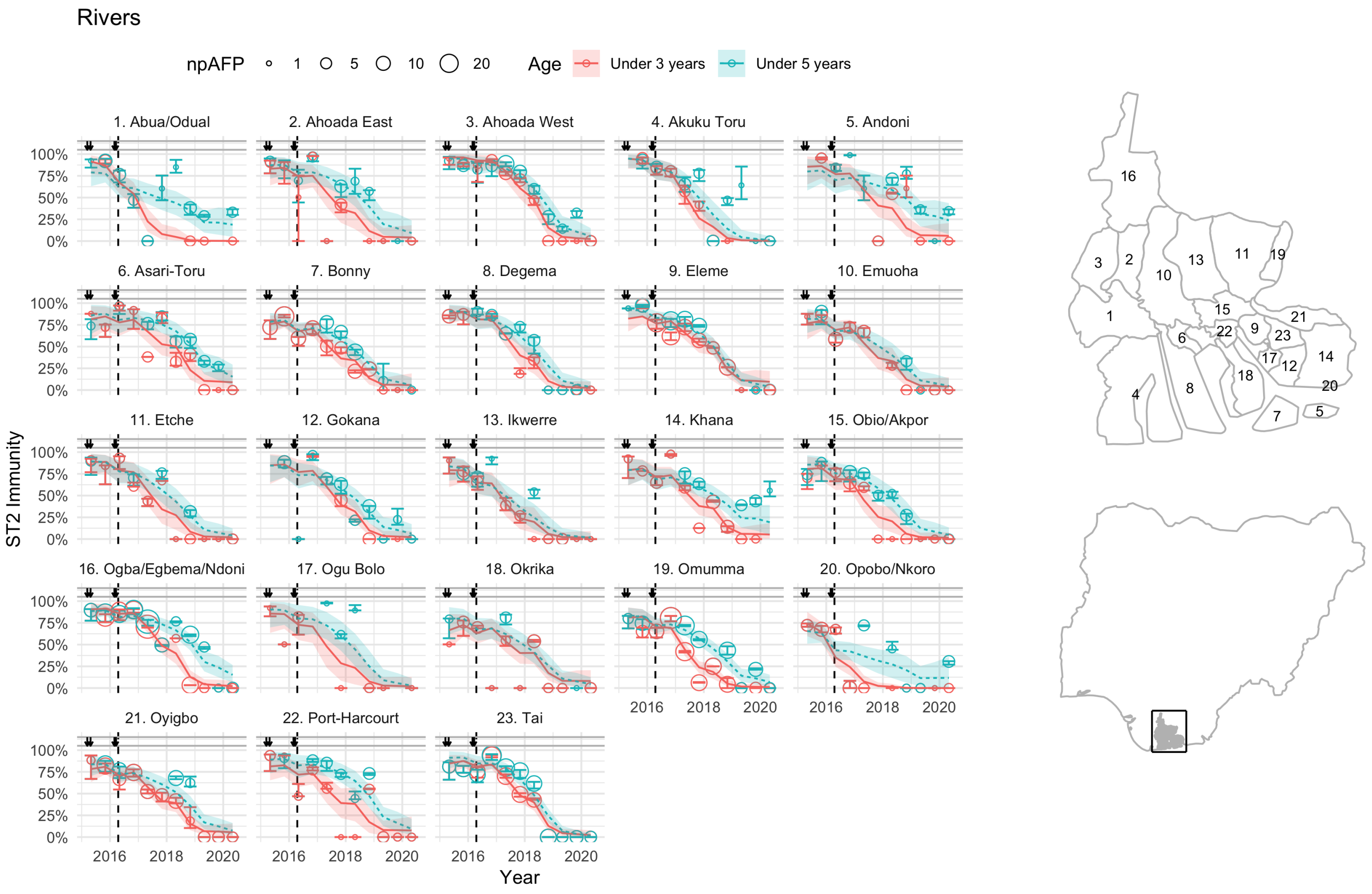

Figure S61: Type 2 population immunity from OPV in each district of Rivers, Nigeria in children under five (blue) and under three (red). Circles show median of bootstrapped crude immunity estimates, error bars show 2.5th and 97.5th percentiles of bootstrapped estimates. Size of circles indicate the number of non-polio AFP cases that each crude estimate is based on. Lines show median smoothed immunity estimate, transparent ribbons show 95% credible interval. Arrows show timing of tOPV (before withdrawal, dotted line) or mOPV2 SIAs (after withdrawal, dotted line). Height of arrows should the proportion of under-five population targeted in SIA. The publication of this map does not imply the expression of any opinion whatsoever on the part of WHO concerning the legal status of any territory, city or area or of its authorities, or concerning the delimitation of its frontiers or boundaries.

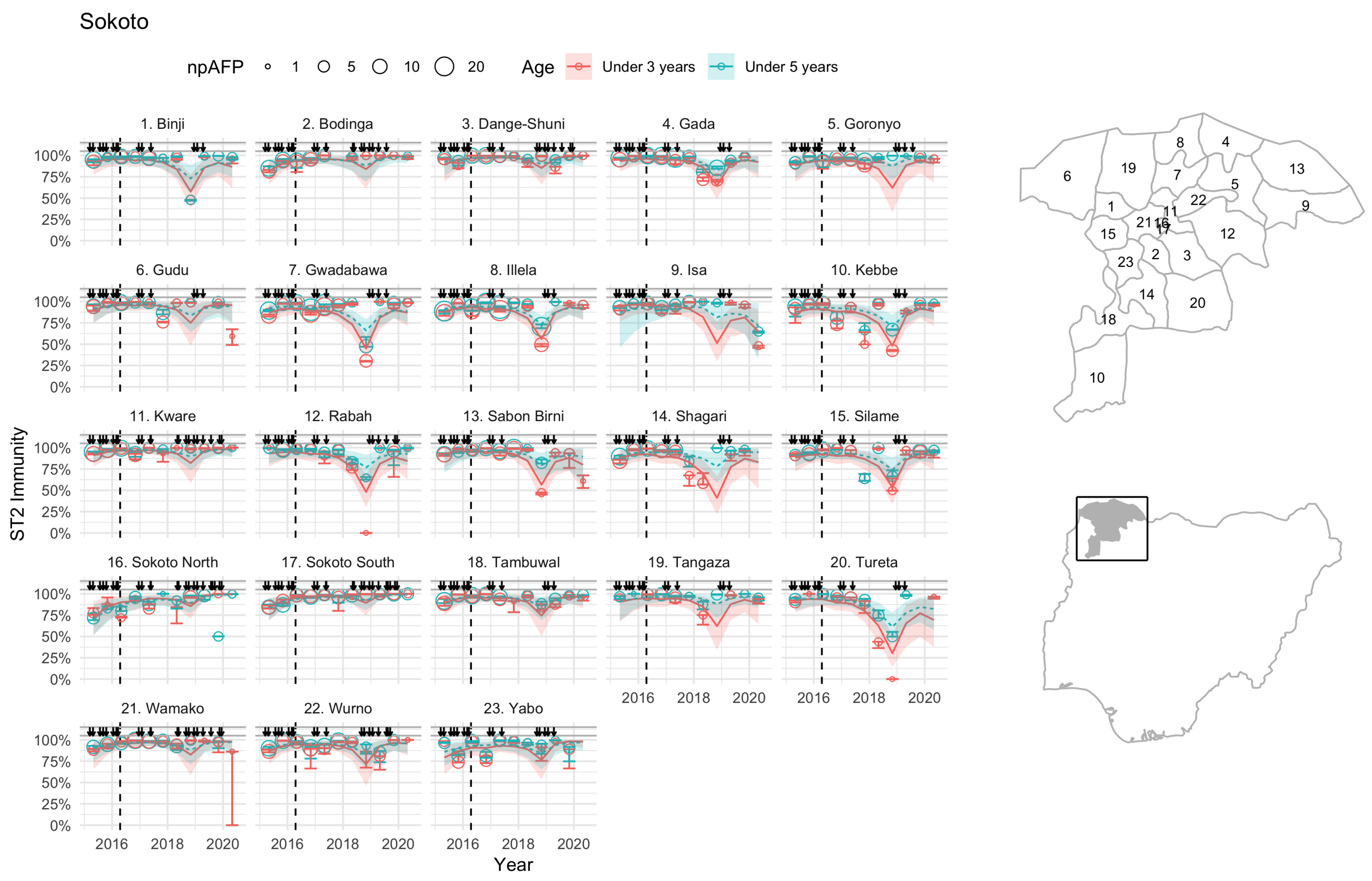

Figure S62: Type 2 population immunity from OPV in each district of Sokoto, Nigeria in children under five (blue) and under three (red). Circles show median of bootstrapped crude immunity estimates, error bars show 2.5th and 97.5th percentiles of bootstrapped estimates. Size of circles indicate the number of non-polio AFP cases that each crude estimate is based on. Lines show median smoothed immunity estimate, transparent ribbons show 95% credible interval. Arrows show timing of tOPV (before withdrawal, dotted line) or mOPV2 SIAs (after withdrawal, dotted line). Height of arrows should the proportion of under-five population targeted in SIA. The publication of this map does not imply the expression of any opinion whatsoever on the part of WHO concerning the legal status of any territory, city or area or of its authorities, or concerning the delimitation of its frontiers or boundaries.

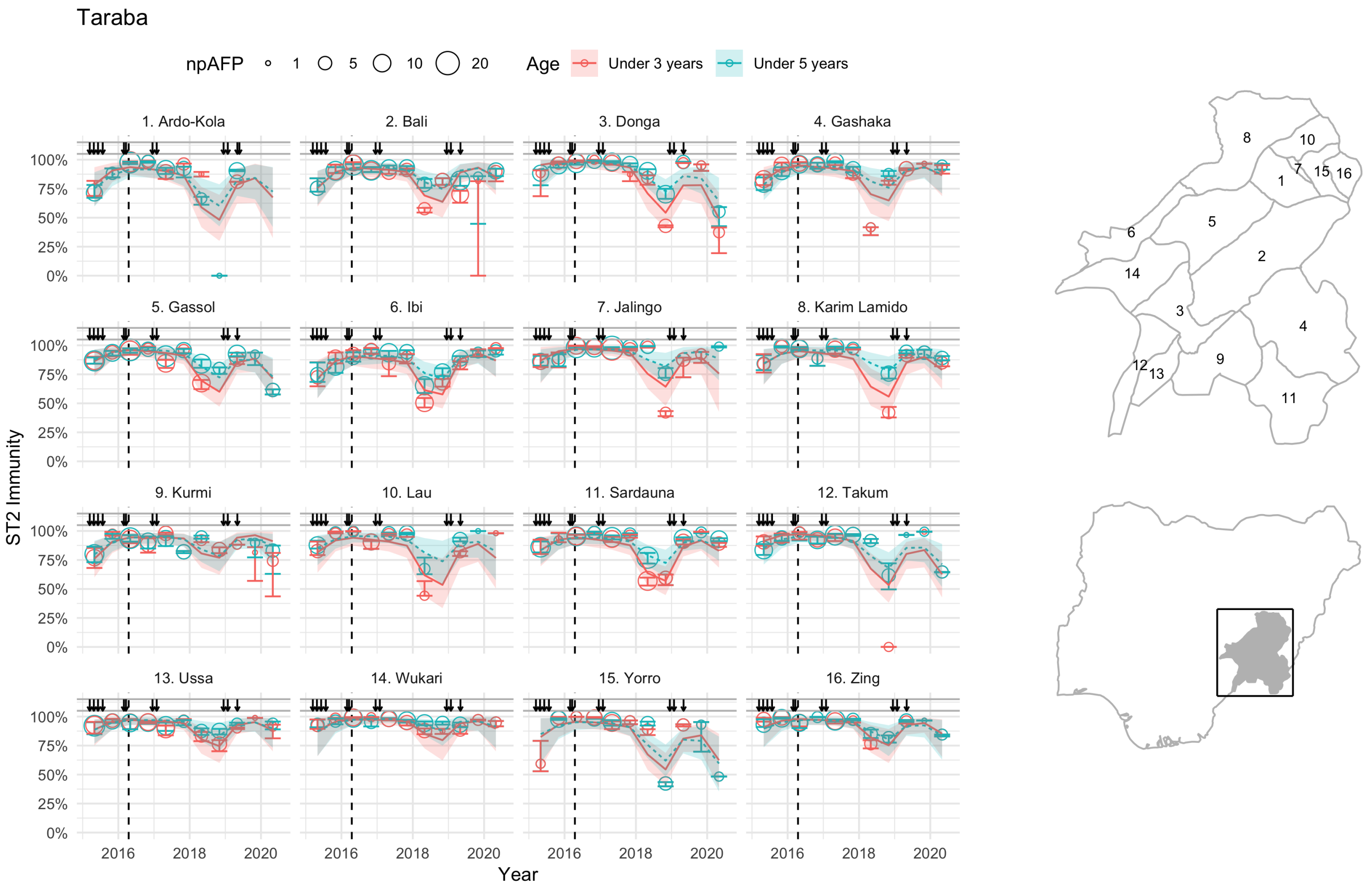

Figure S63: Type 2 population immunity from OPV in each district of Taraba, Nigeria in children under five (blue) and under three (red). Circles show median of bootstrapped crude immunity estimates, error bars show 2.5th and 97.5th percentiles of bootstrapped estimates. Size of circles indicate the number of non-polio AFP cases that each crude estimate is based on. Lines show median smoothed immunity estimate, transparent ribbons show 95% credible interval. Arrows show timing of tOPV (before withdrawal, dotted line) or mOPV2 SIAs (after withdrawal, dotted line). Height of arrows should the proportion of under-five population targeted in SIA. The publication of this map does not imply the expression of any opinion whatsoever on the part of WHO concerning the legal status of any territory, city or area or of its authorities, or concerning the delimitation of its frontiers or boundaries.

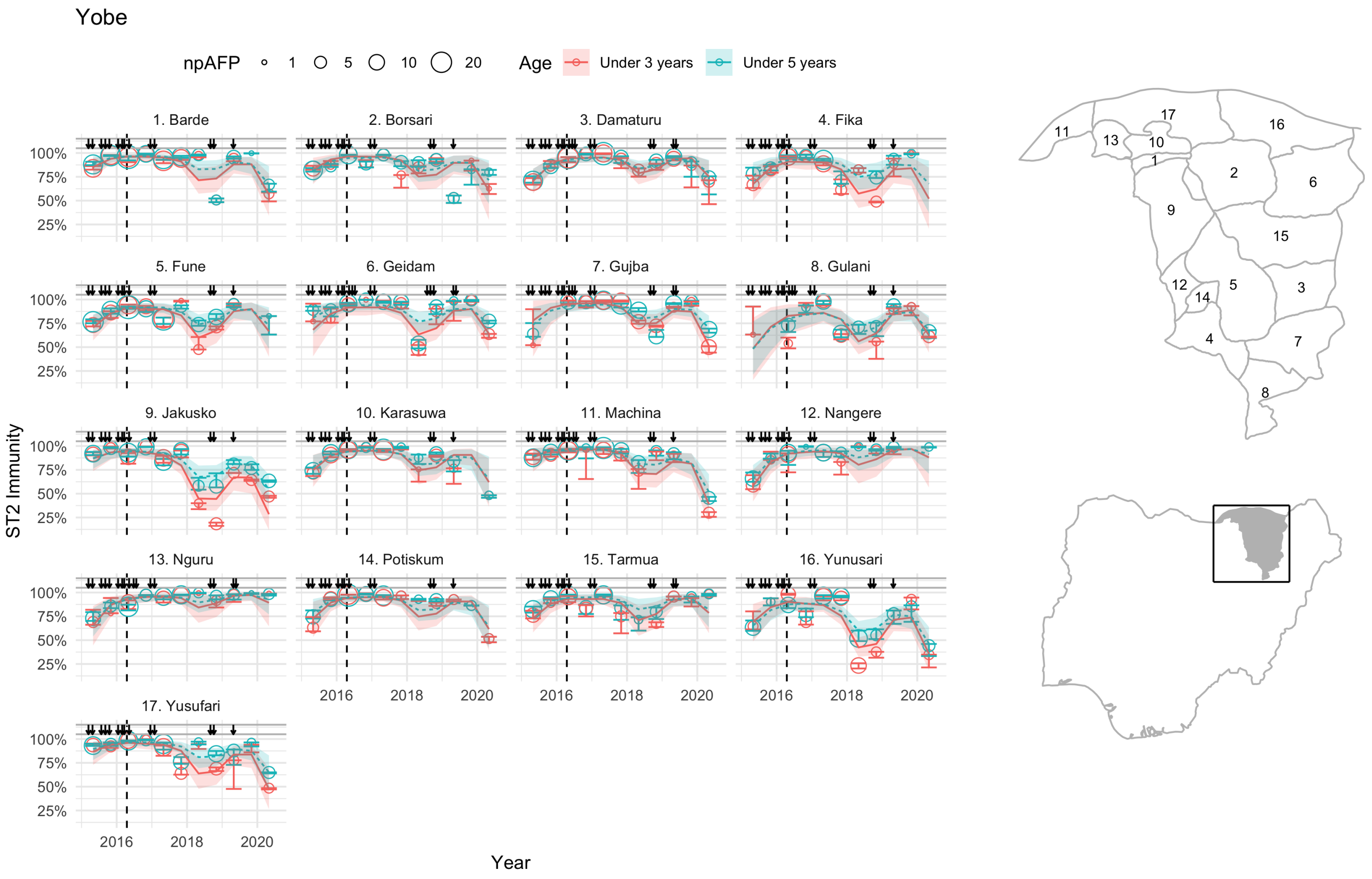

Figure S64: Type 2 population immunity from OPV in each district of Yobe, Nigeria in children under five (blue) and under three (red). Circles show median of bootstrapped crude immunity estimates, error bars show 2.5th and 97.5th percentiles of bootstrapped estimates. Size of circles indicate the number of non-polio AFP cases that each crude estimate is based on. Lines show median smoothed immunity estimate, transparent ribbons show 95% credible interval. Arrows show timing of tOPV (before withdrawal, dotted line) or mOPV2 SIAs (after withdrawal, dotted line). Height of arrows should the proportion of under-five population targeted in SIA. The publication of this map does not imply the expression of any opinion whatsoever on the part of WHO concerning the legal status of any territory, city or area or of its authorities, or concerning the delimitation of its frontiers or boundaries.

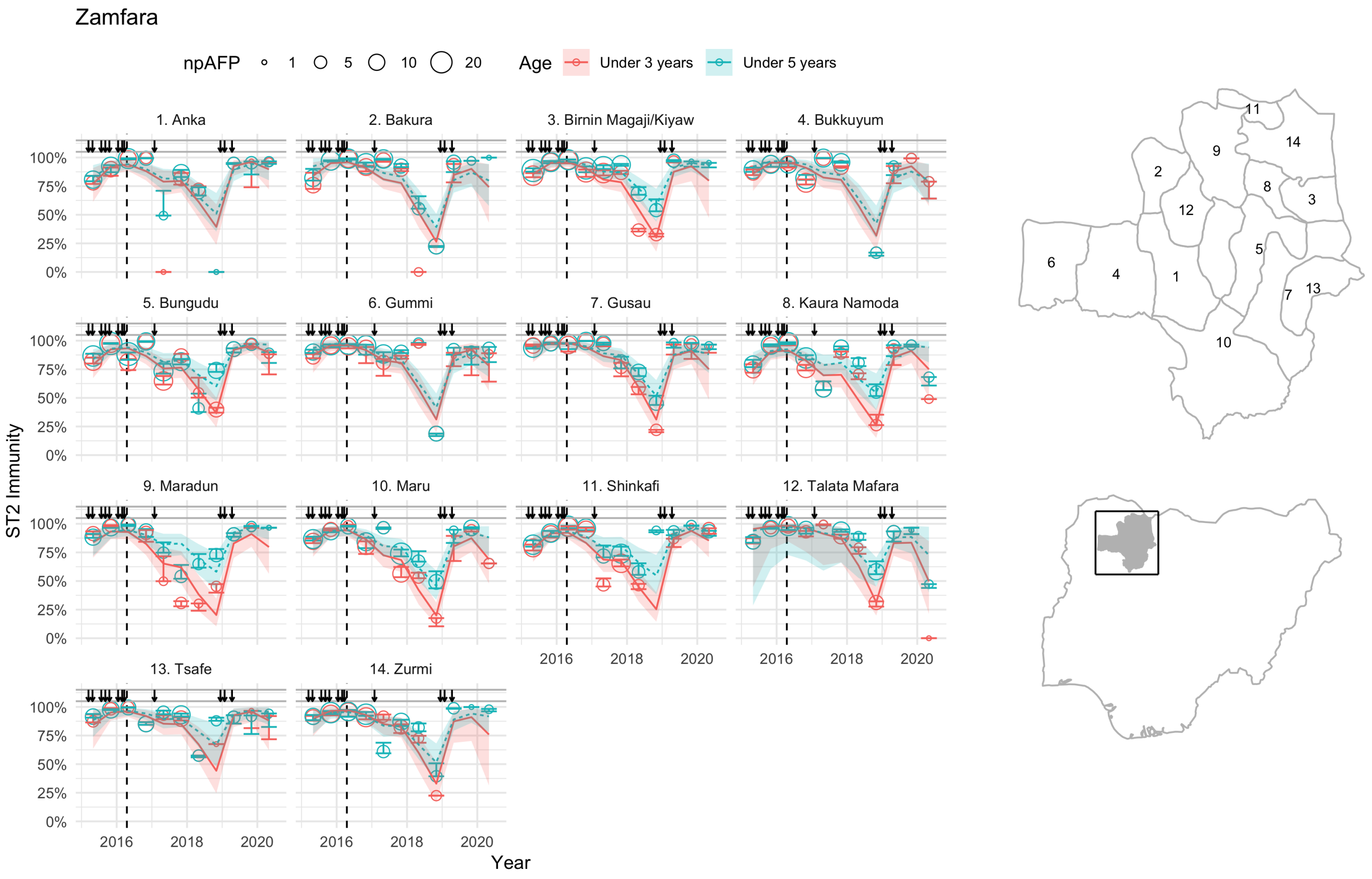

Figure S65: Type 2 population immunity from OPV in each district of Zamfara, Nigeria in children under five (blue) and under three (red). Circles show median of bootstrapped crude immunity estimates, error bars show 2.5th and 97.5th percentiles of bootstrapped estimates. Size of circles indicate the number of non-polio AFP cases that each crude estimate is based on. Lines show median smoothed immunity estimate, transparent ribbons show 95% credible interval. Arrows show timing of tOPV (before withdrawal, dotted line) or mOPV2 SIAs (after withdrawal, dotted line). Height of arrows should the proportion of under-five population targeted in SIA. The publication of this map does not imply the expression of any opinion whatsoever on the part of WHO concerning the legal status of any territory, city or area or of its authorities, or concerning the delimitation of its frontiers or boundaries.

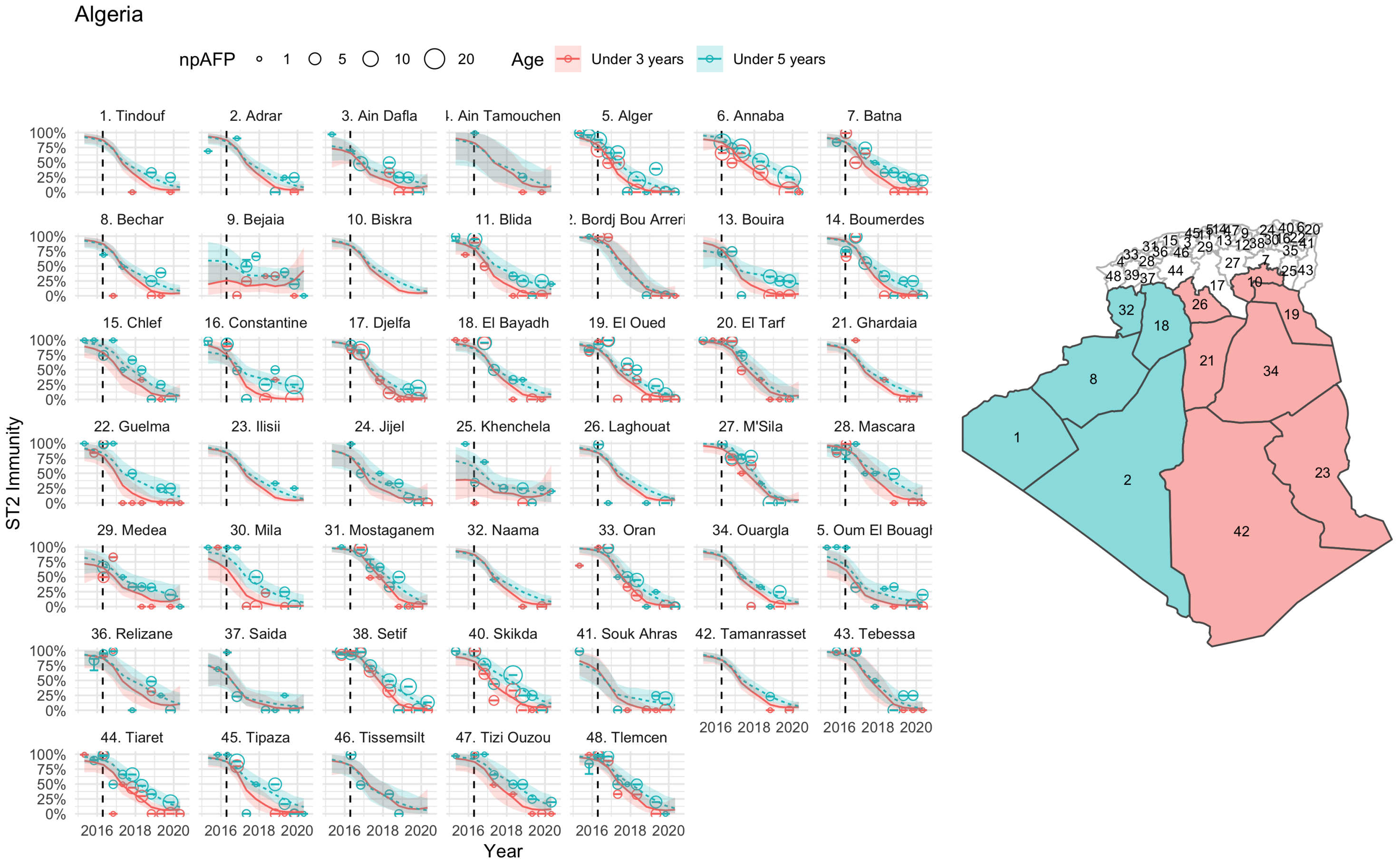

Figure S66: Type 2 population immunity from OPV in each province of Algeria in children under five (blue) and under three (red). Circles show median of bootstrapped crude immunity estimates, error bars show 2.5th and 97.5th percentiles of bootstrapped estimates. Size of circles indicate the number of non-polio AFP cases that each crude estimate is based on. Lines show median smoothed immunity estimate, transparent ribbons show 95% credible interval. Arrows show timing of tOPV (before withdrawal, dotted line) or mOPV2 SIAs (after withdrawal, dotted line). Height of arrows should the proportion of under-five population targeted in SIA. Shaded areas on map indicate grouped provinces. The publication of this map does not imply the expression of any opinion whatsoever on the part of WHO concerning the legal status of any territory, city or area or of its authorities, or concerning the delimitation of its frontiers or boundaries.

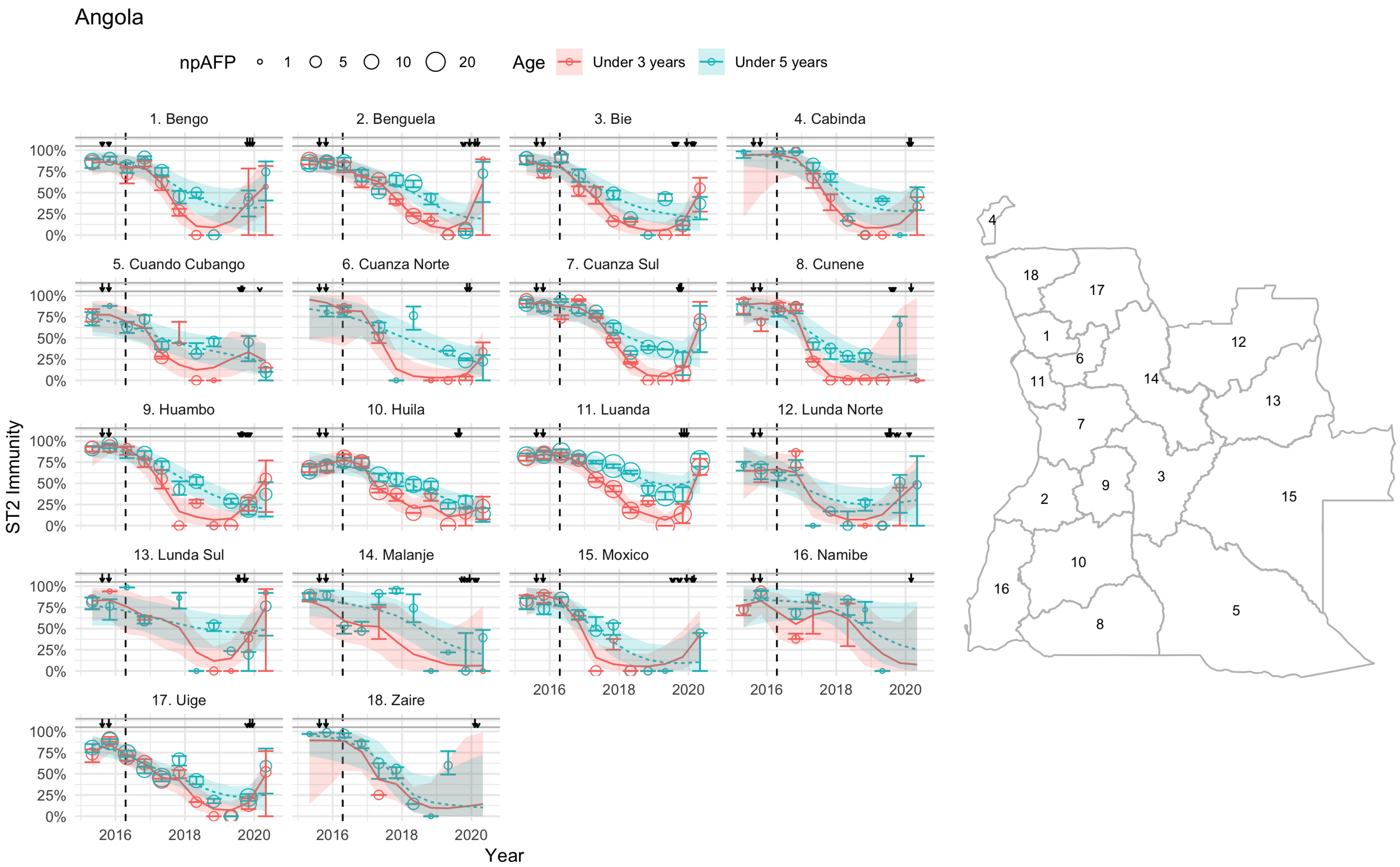

Figure S67: Type 2 population immunity from OPV in each province of Angola in children under five (blue) and under three (red). Circles show median of bootstrapped crude immunity estimates, error bars show 2.5th and 97.5th percentiles of bootstrapped estimates. Size of circles indicate the number of non-polio AFP cases that each crude estimate is based on. Lines show median smoothed immunity estimate, transparent ribbons show 95% credible interval. Arrows show timing of tOPV (before withdrawal, dotted line) or mOPV2 SIAs (after withdrawal, dotted line). Height of arrows should the proportion of under-five population targeted in SIA. The publication of this map does not imply the expression of any opinion whatsoever on the part of WHO concerning the legal status of any territory, city or area or of its authorities, or concerning the delimitation of its frontiers or boundaries.

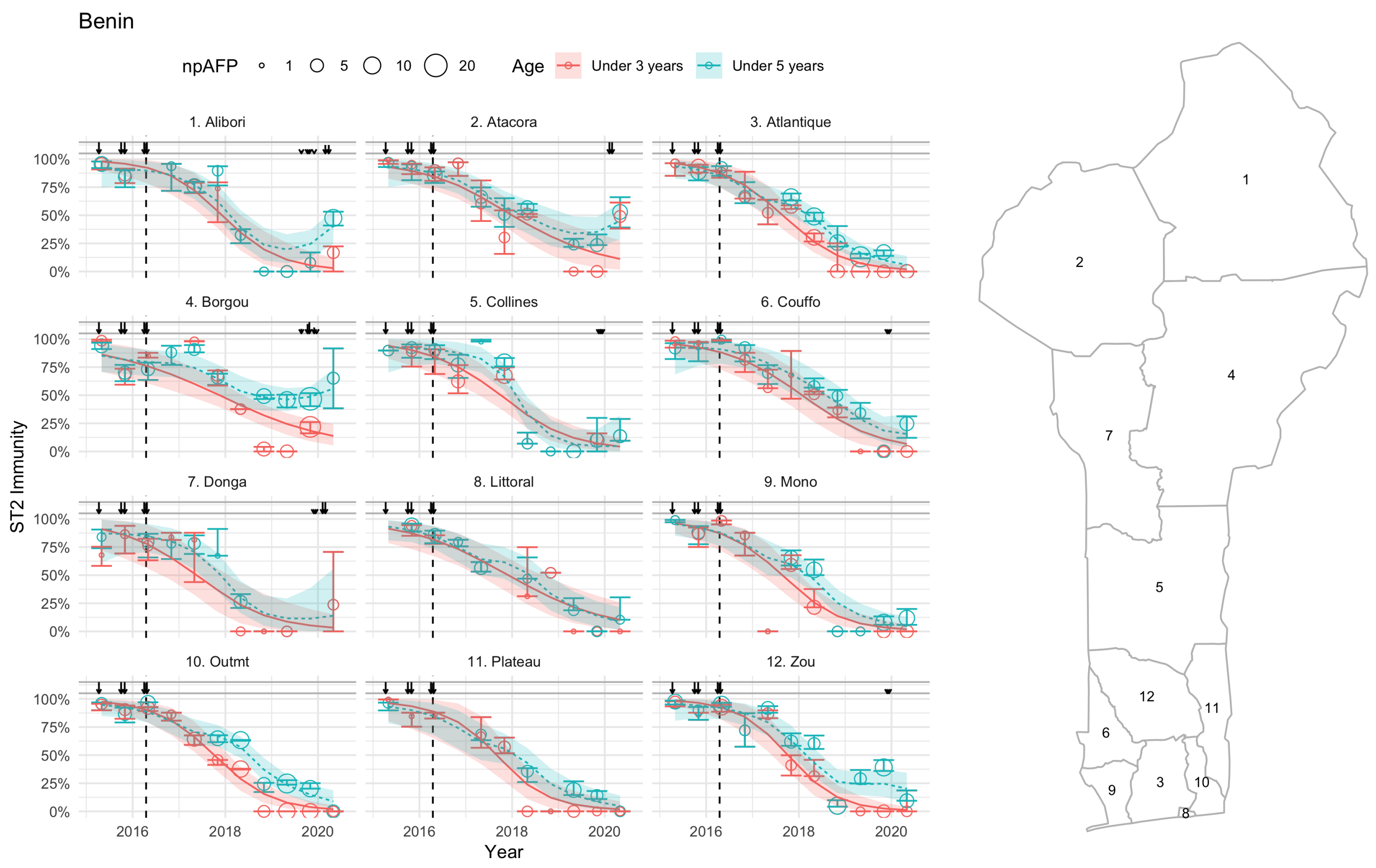

Figure S68: Type 2 population immunity from OPV in each province of Benin in children under five (blue) and under three (red). Circles show median of bootstrapped crude immunity estimates, error bars show 2.5th and 97.5th percentiles of bootstrapped estimates. Size of circles indicate the number of non-polio AFP cases that each crude estimate is based on. Lines show median smoothed immunity estimate, transparent ribbons show 95% credible interval. Arrows show timing of tOPV (before withdrawal, dotted line) or mOPV2 SIAs (after withdrawal, dotted line). Height of arrows should the proportion of under-five population targeted in SIA. The publication of this map does not imply the expression of any opinion whatsoever on the part of WHO concerning the legal status of any territory, city or area or of its authorities, or concerning the delimitation of its frontiers or boundaries.

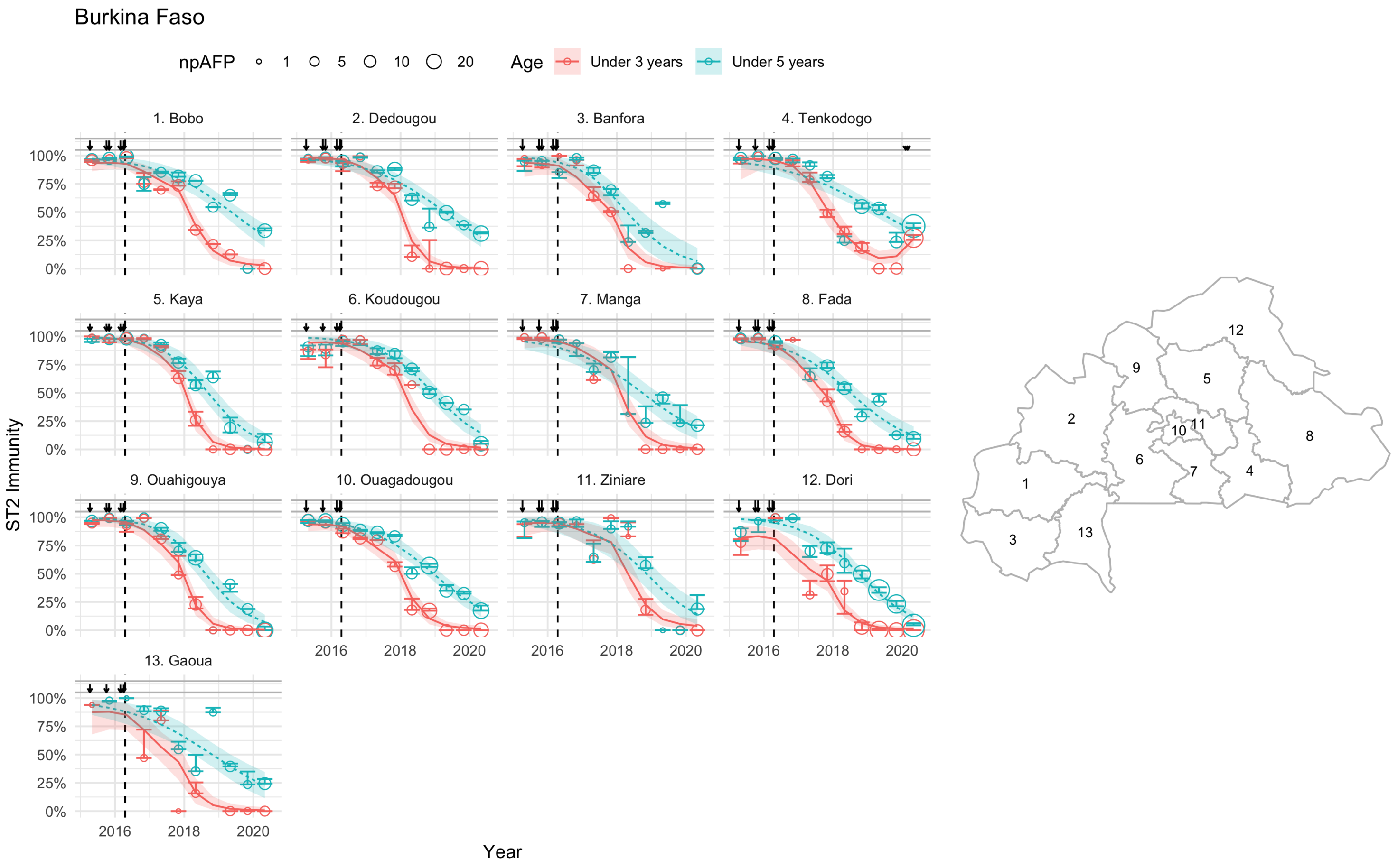

Figure S69: Type 2 population immunity from OPV in each province of Burkina Faso in children under five (blue) and under three (red). Circles show median of bootstrapped crude immunity estimates, error bars show 2.5th and 97.5th percentiles of bootstrapped estimates. Size of circles indicate the number of non-polio AFP cases that each crude estimate is based on. Lines show median smoothed immunity estimate, transparent ribbons show 95% credible interval. Arrows show timing of tOPV (before withdrawal, dotted line) or mOPV2 SIAs (after withdrawal, dotted line). Height of arrows should the proportion of under-five population targeted in SIA. The publication of this map does not imply the expression of any opinion whatsoever on the part of WHO concerning the legal status of any territory, city or area or of its authorities, or concerning the delimitation of its frontiers or boundaries.

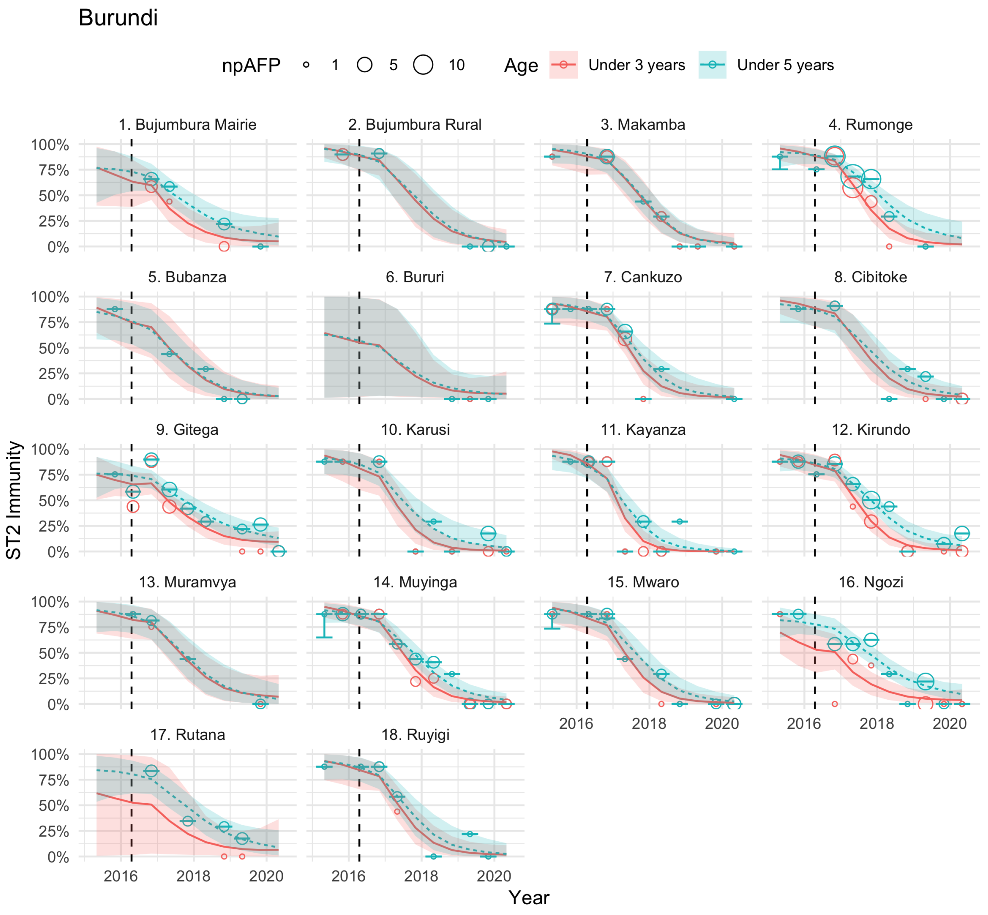

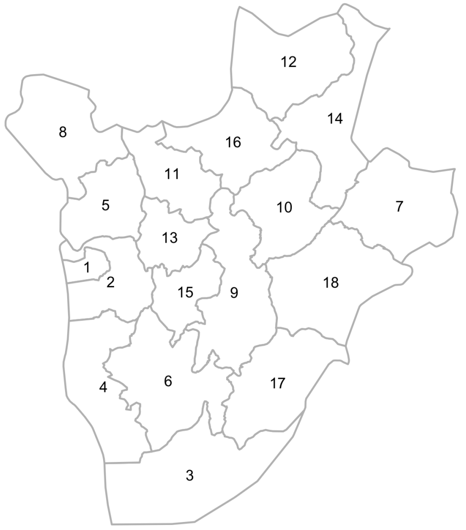

Figure S70: Type 2 population immunity from OPV in each province of Burundi in children under five (blue) and under three (red). Circles show median of bootstrapped crude immunity estimates, error bars show 2.5th and 97.5th percentiles of bootstrapped estimates. Size of circles indicate the number of non-polio AFP cases that each crude estimate is based on. Lines show median smoothed immunity estimate, transparent ribbons show 95% credible interval. Arrows show timing of tOPV (before withdrawal, dotted line) or mOPV2 SIAs (after withdrawal, dotted line). Height of arrows should the proportion of under-five population targeted in SIA. The publication of this map does not imply the expression of any opinion whatsoever on the part of WHO concerning the legal status of any territory, city or area or of its authorities, or concerning the delimitation of its frontiers or boundaries.

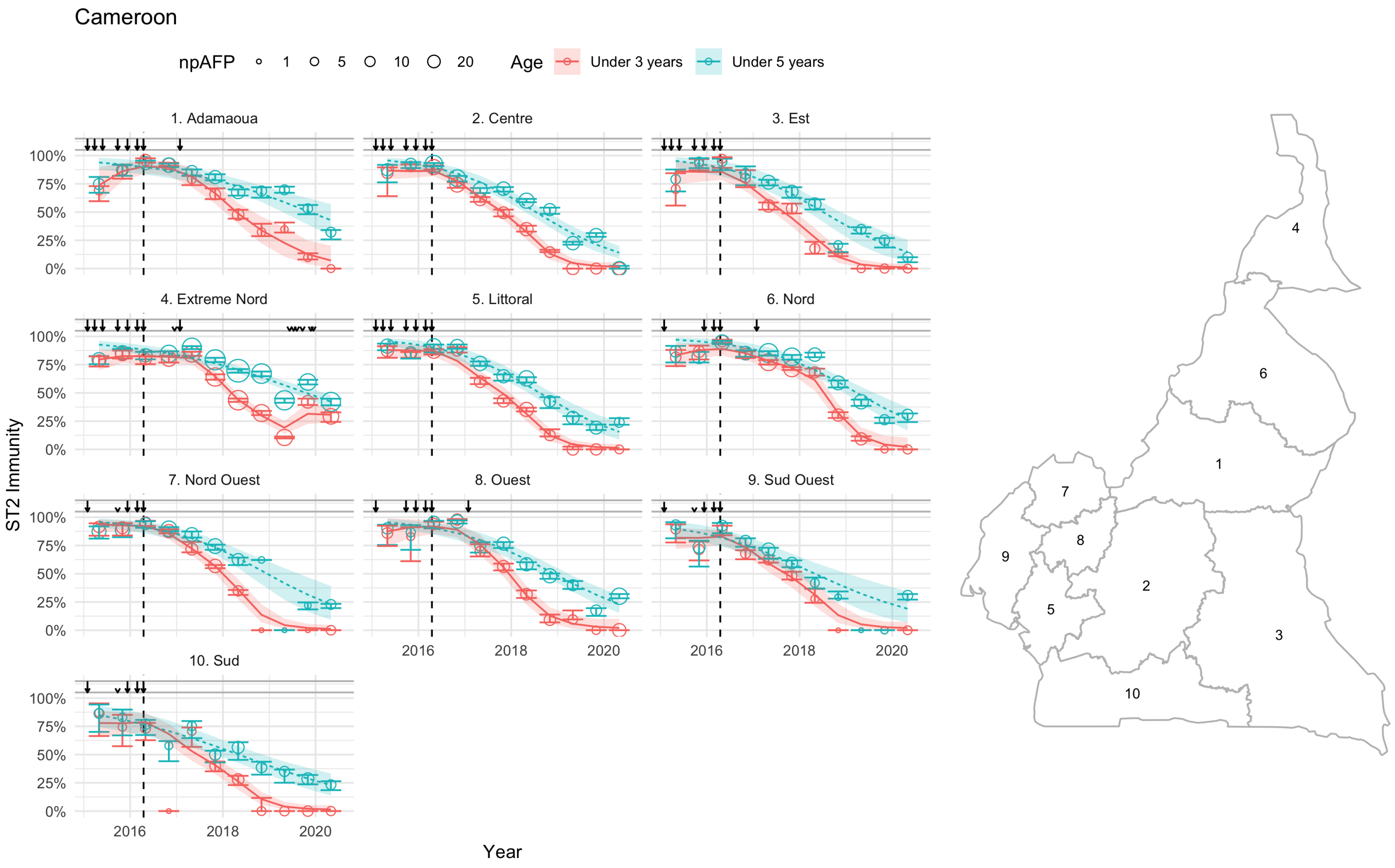

Figure S71: Type 2 population immunity from OPV in each province of Cameroon in children under five (blue) and under three (red). Circles show median of bootstrapped crude immunity estimates, error bars show 2.5th and 97.5th percentiles of bootstrapped estimates. Size of circles indicate the number of non-polio AFP cases that each crude estimate is based on. Lines show median smoothed immunity estimate, transparent ribbons show 95% credible interval. Arrows show timing of tOPV (before withdrawal, dotted line) or mOPV2 SIAs (after withdrawal, dotted line). Height of arrows should the proportion of under-five population targeted in SIA. The publication of this map does not imply the expression of any opinion whatsoever on the part of WHO concerning the legal status of any territory, city or area or of its authorities, or concerning the delimitation of its frontiers or boundaries.

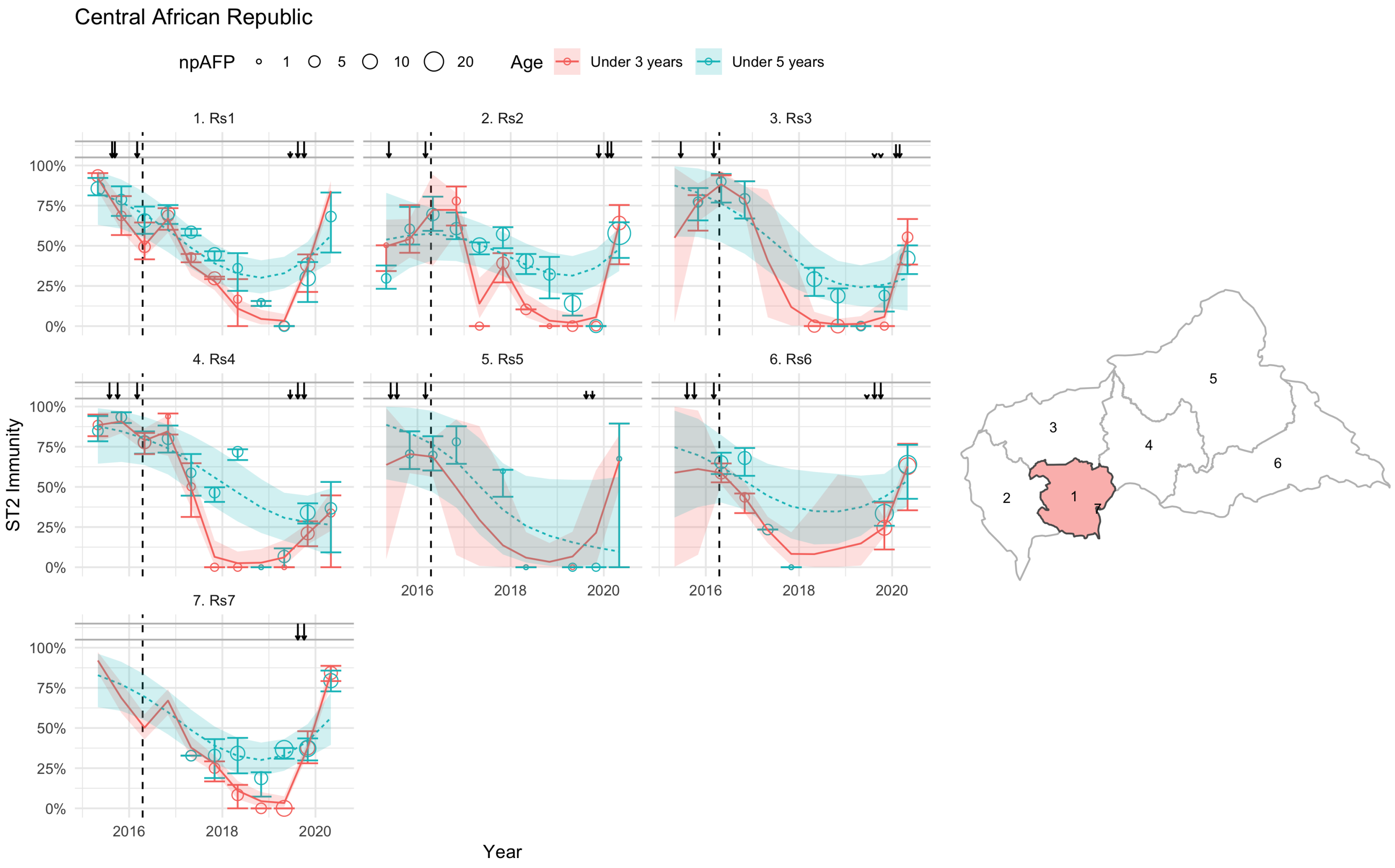

Figure S72: Type 2 population immunity from OPV in each province of Central African Republic in children under five (blue) and under three (red). Circles show median of bootstrapped crude immunity estimates, error bars show 2.5th and 97.5th percentiles of bootstrapped estimates. Size of circles indicate the number of non-polio AFP cases that each crude estimate is based on. Lines show median smoothed immunity estimate, transparent ribbons show 95% credible interval. Arrows show timing of tOPV (before withdrawal, dotted line) or mOPV2 SIAs (after withdrawal, dotted line). Height of arrows should the proportion of under-five population targeted in SIA. Shaded areas on map indicate grouped provinces. The publication of this map does not imply the expression of any opinion whatsoever on the part of WHO concerning the legal status of any territory, city or area or of its authorities, or concerning the delimitation of its frontiers or boundaries.

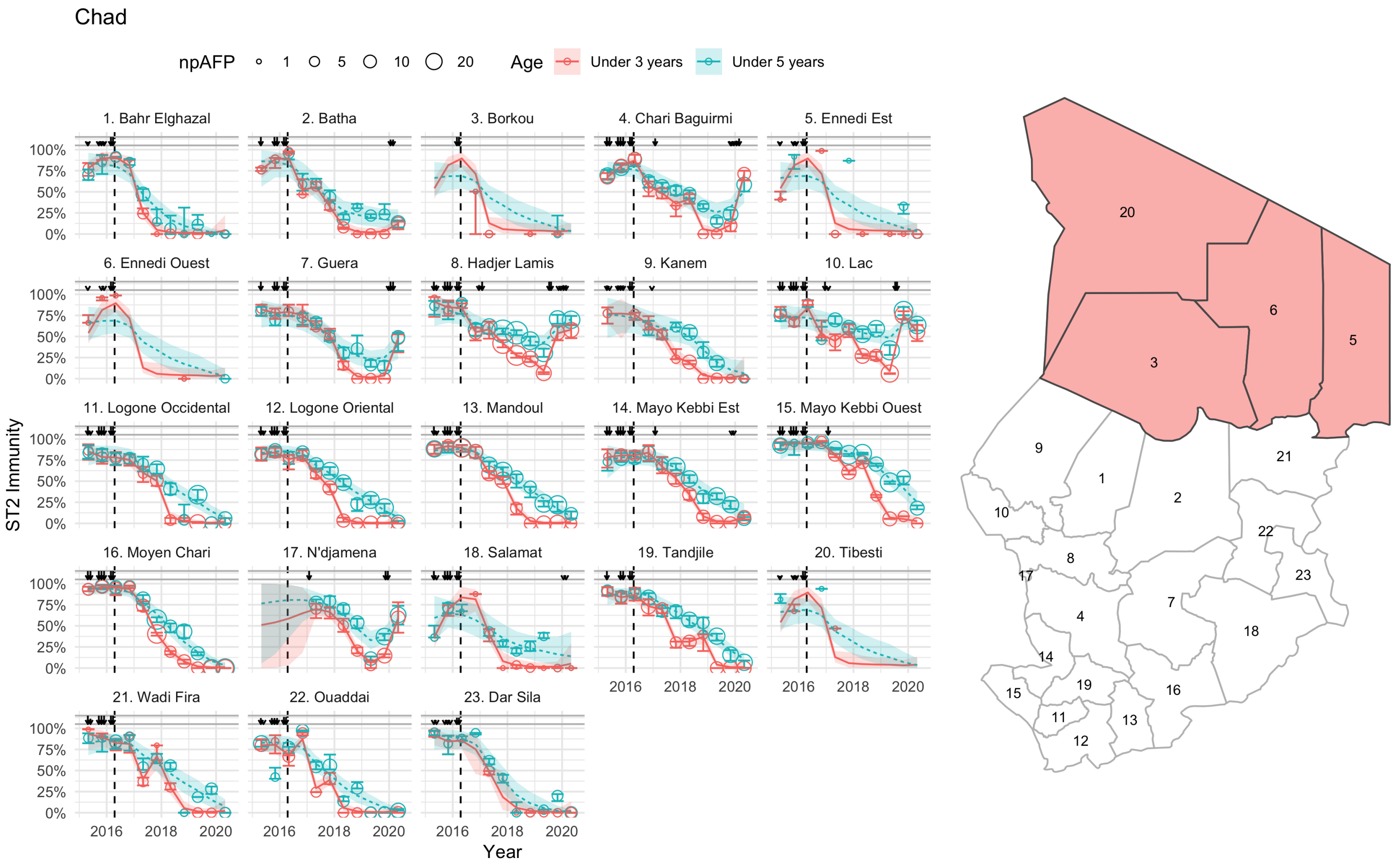

Figure S73: Type 2 population immunity from OPV in each province of Chad in children under five (blue) and under three (red). Circles show median of bootstrapped crude immunity estimates, error bars show 2.5th and 97.5th percentiles of bootstrapped estimates. Size of circles indicate the number of non-polio AFP cases that each crude estimate is based on. Lines show median smoothed immunity estimate, transparent ribbons show 95% credible interval. Arrows show timing of tOPV (before withdrawal, dotted line) or mOPV2 SIAs (after withdrawal, dotted line). Height of arrows should the proportion of under-five population targeted in SIA. Shaded areas on map indicate grouped provinces. The publication of this map does not imply the expression of any opinion whatsoever on the part of WHO concerning the legal status of any territory, city or area or of its authorities, or concerning the delimitation of its frontiers or boundaries.

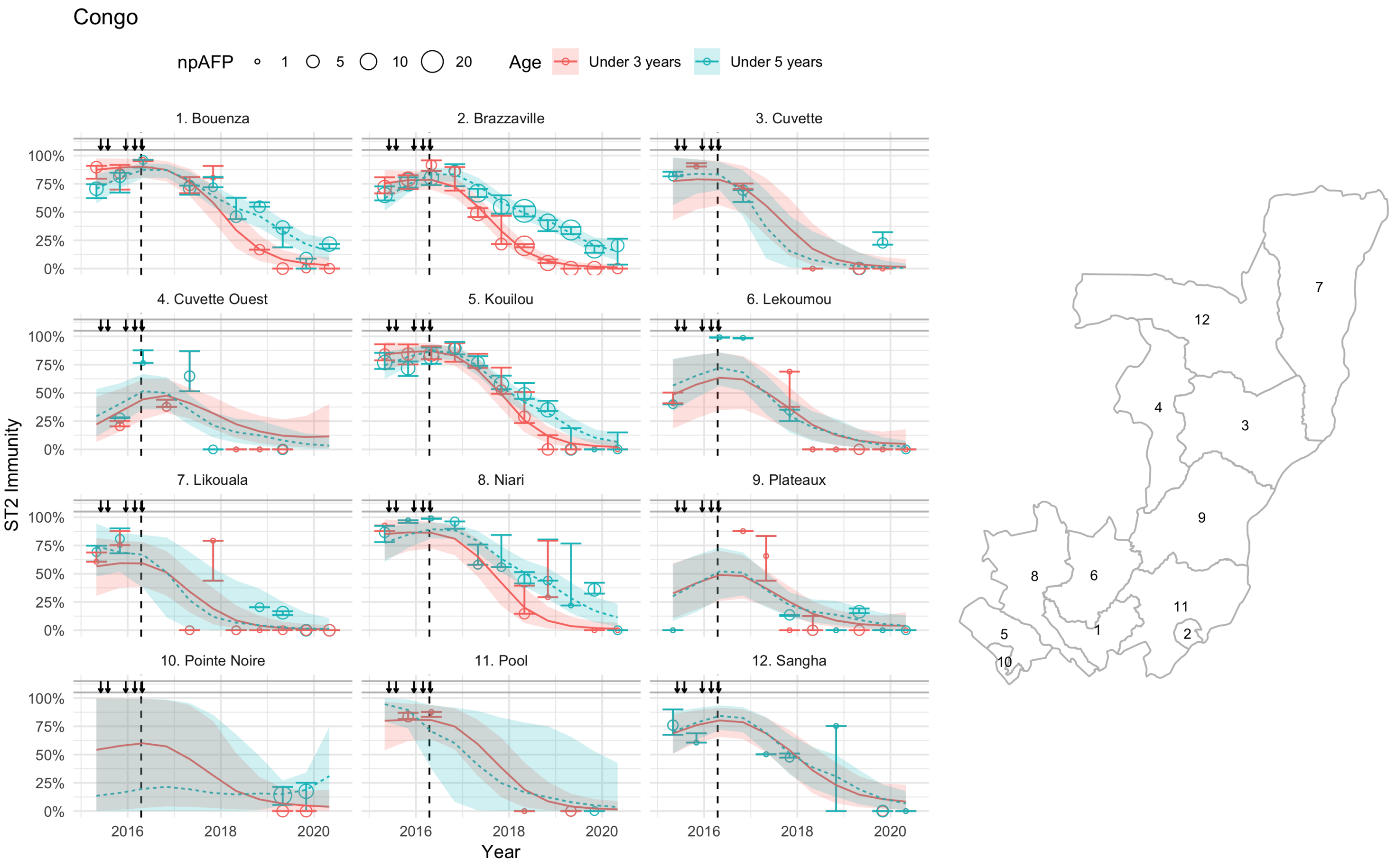

Figure S74: Type 2 population immunity from OPV in each province of Congo in children under five (blue) and under three (red). Circles show median of bootstrapped crude immunity estimates, error bars show 2.5th and 97.5th percentiles of bootstrapped estimates. Size of circles indicate the number of non-polio AFP cases that each crude estimate is based on. Lines show median smoothed immunity estimate, transparent ribbons show 95% credible interval. Arrows show timing of tOPV (before withdrawal, dotted line) or mOPV2 SIAs (after withdrawal, dotted line). Height of arrows should the proportion of under-five population targeted in SIA. The publication of this map does not imply the expression of any opinion whatsoever on the part of WHO concerning the legal status of any territory, city or area or of its authorities, or concerning the delimitation of its frontiers or boundaries.

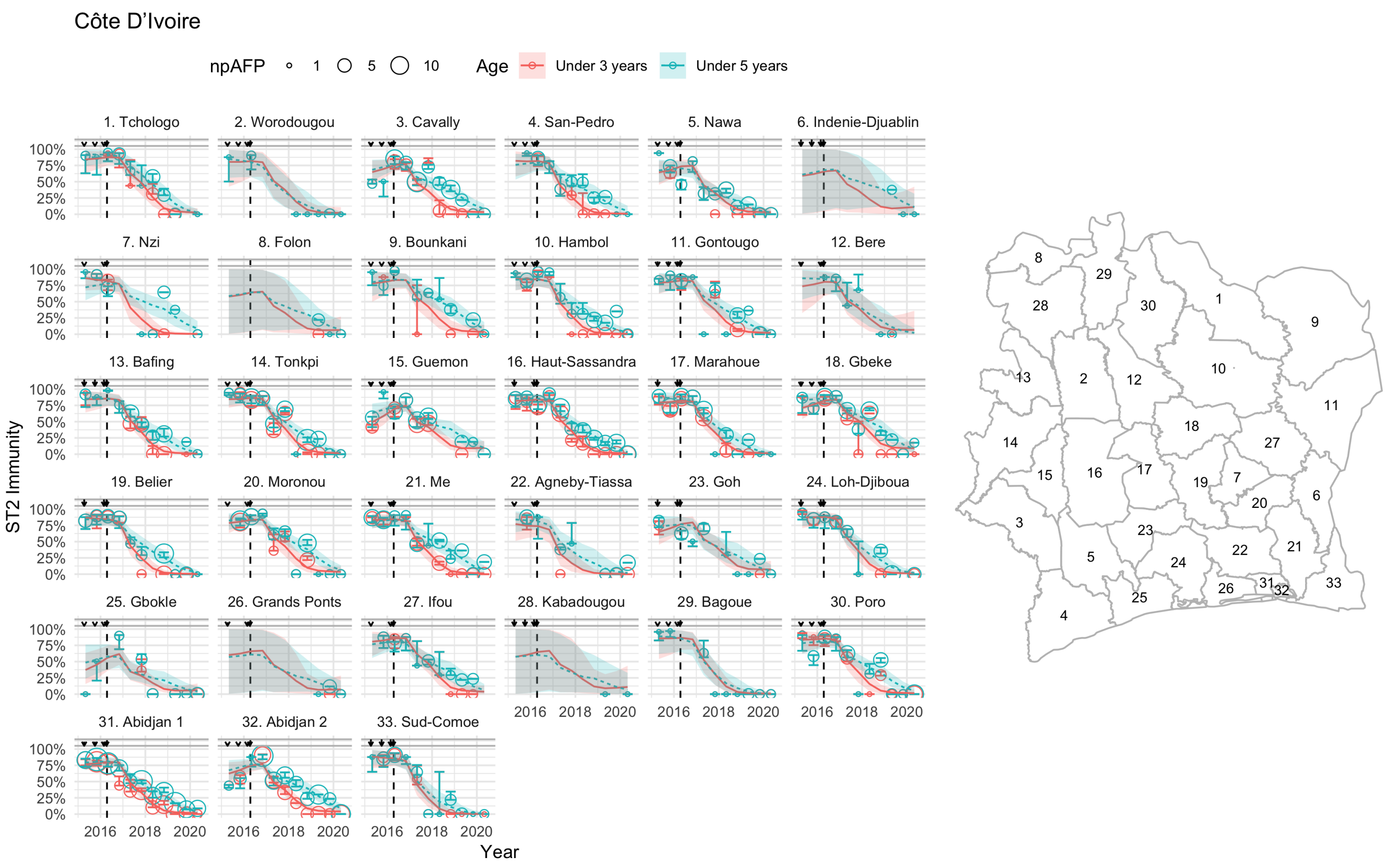

Figure S75: Type 2 population immunity from OPV in each province of Cote d'Ivoire in children under five (blue) and under three (red). Circles show median of bootstrapped crude immunity estimates, error bars show 2.5th and 97.5th percentiles of bootstrapped estimates. Size of circles indicate the number of non-polio AFP cases that each crude estimate is based on. Lines show median smoothed immunity estimate, transparent ribbons show 95% credible interval. Arrows show timing of tOPV (before withdrawal, dotted line) or mOPV2 SIAs (after withdrawal, dotted line). Height of arrows should the proportion of under-five population targeted in SIA. The publication of this map does not imply the expression of any opinion whatsoever on the part of WHO concerning the legal status of any territory, city or area or of its authorities, or concerning the delimitation of its frontiers or boundaries.

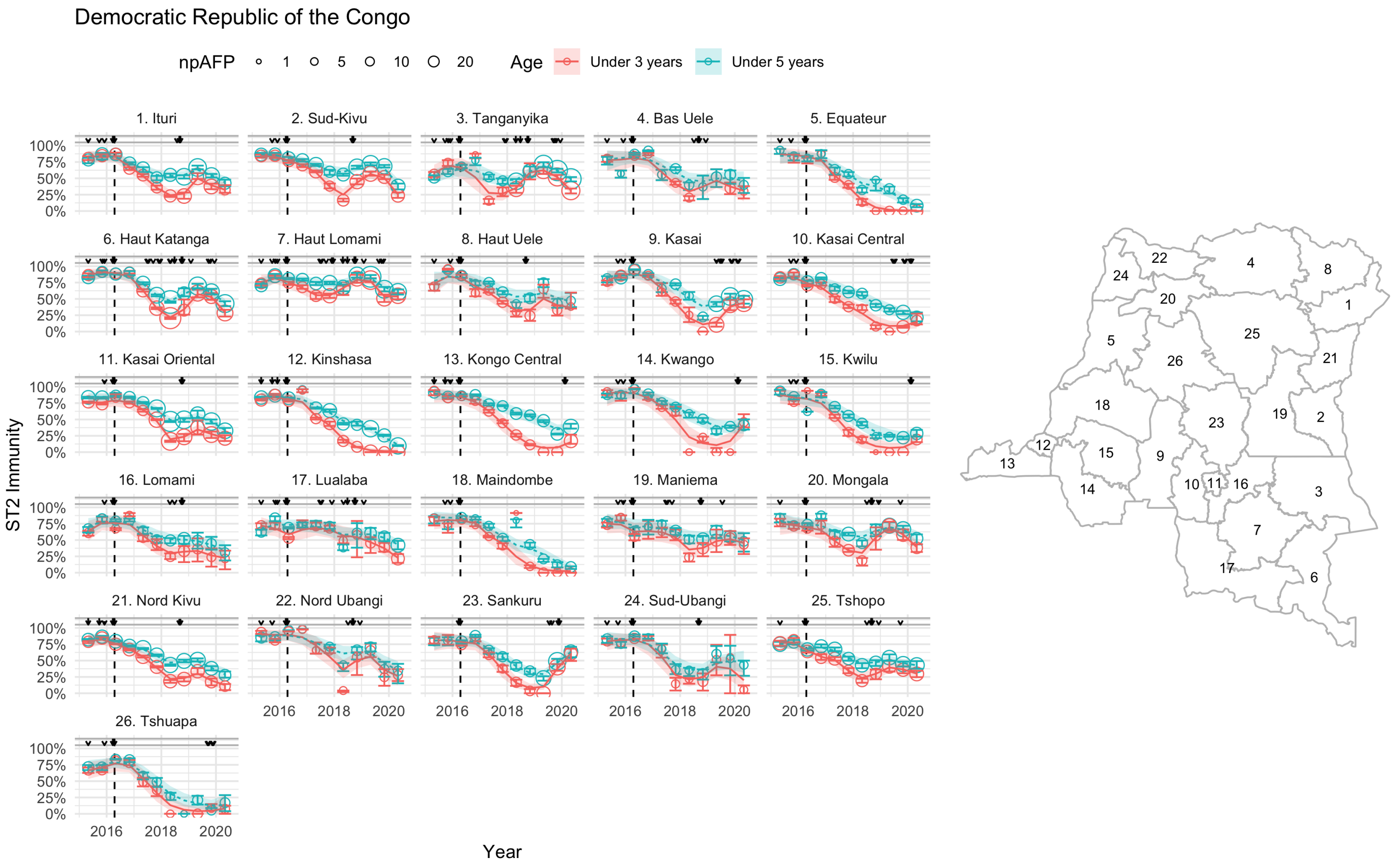

Figure S76: Type 2 population immunity from OPV in each province of Democratic Republic of the Congo in children under five (blue) and under three (red). Circles show median of bootstrapped crude immunity estimates, error bars show 2.5th and 97.5th percentiles of bootstrapped estimates. Size of circles indicate the number of non-polio AFP cases that each crude estimate is based on. Lines show median smoothed immunity estimate, transparent ribbons show 95% credible interval. Arrows show timing of tOPV (before withdrawal, dotted line) or mOPV2 SIAs (after withdrawal, dotted line). Height of arrows should the proportion of under-five population targeted in SIA. The publication of this map does not imply the expression of any opinion whatsoever on the part of WHO concerning the legal status of any territory, city or area or of its authorities, or concerning the delimitation of its frontiers or boundaries.

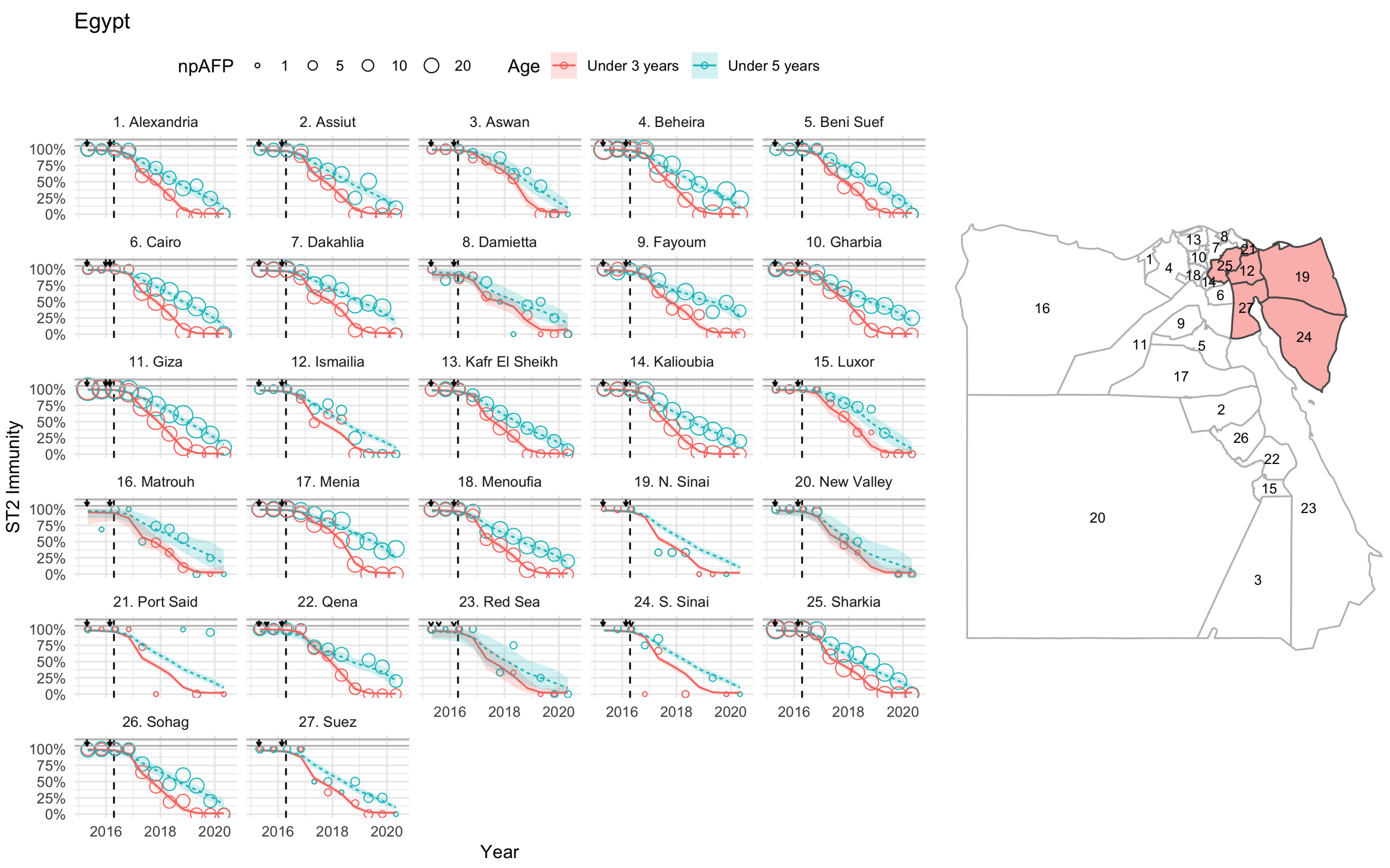

Figure S77: Type 2 population immunity from OPV in each province of Egypt in children under five (blue) and under three (red). Circles show median of bootstrapped crude immunity estimates, error bars show 2.5th and 97.5th percentiles of bootstrapped estimates. Size of circles indicate the number of non-polio AFP cases that each crude estimate is based on. Lines show median smoothed immunity estimate, transparent ribbons show 95% credible interval. Arrows show timing of tOPV (before withdrawal, dotted line) or mOPV2 SIAs (after withdrawal, dotted line). Height of arrows should the proportion of under-five population targeted in SIA. Shaded areas on map indicate grouped provinces. The publication of this map does not imply the expression of any opinion whatsoever on the part of WHO concerning the legal status of any territory, city or area or of its authorities, or concerning the delimitation of its frontiers or boundaries.

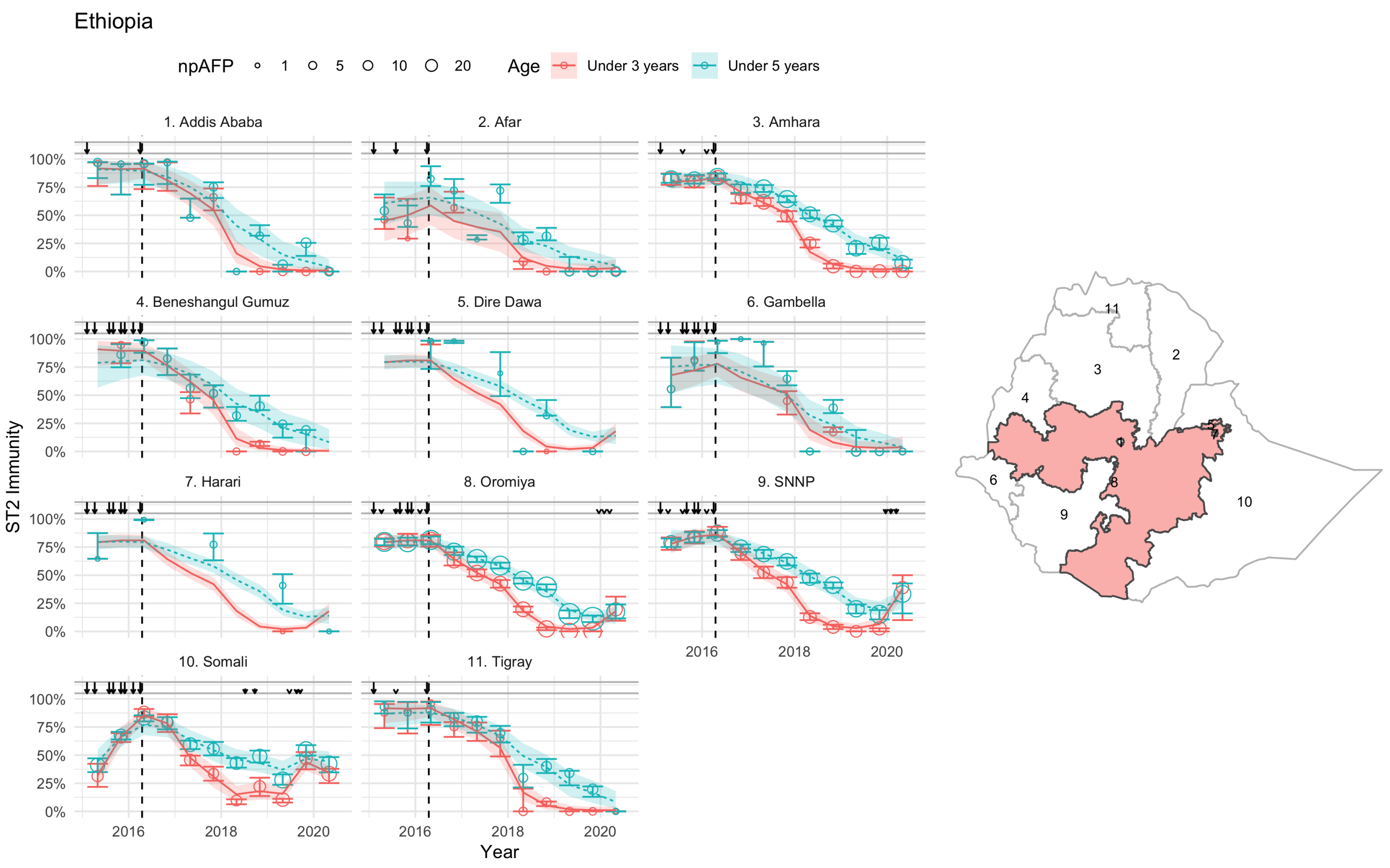

Figure S78: Type 2 population immunity from OPV in each province of Ethiopia in children under five (blue) and under three (red). Circles show median of bootstrapped crude immunity estimates, error bars show 2.5th and 97.5th percentiles of bootstrapped estimates. Size of circles indicate the number of non-polio AFP cases that each crude estimate is based on. Lines show median smoothed immunity estimate, transparent ribbons show 95% credible interval. Arrows show timing of tOPV (before withdrawal, dotted line) or mOPV2 SIAs (after withdrawal, dotted line). Height of arrows should the proportion of under-five population targeted in SIA. Shaded areas on map indicate grouped provinces. The publication of this map does not imply the expression of any opinion whatsoever on the part of WHO concerning the legal status of any territory, city or area or of its authorities, or concerning the delimitation of its frontiers or boundaries.

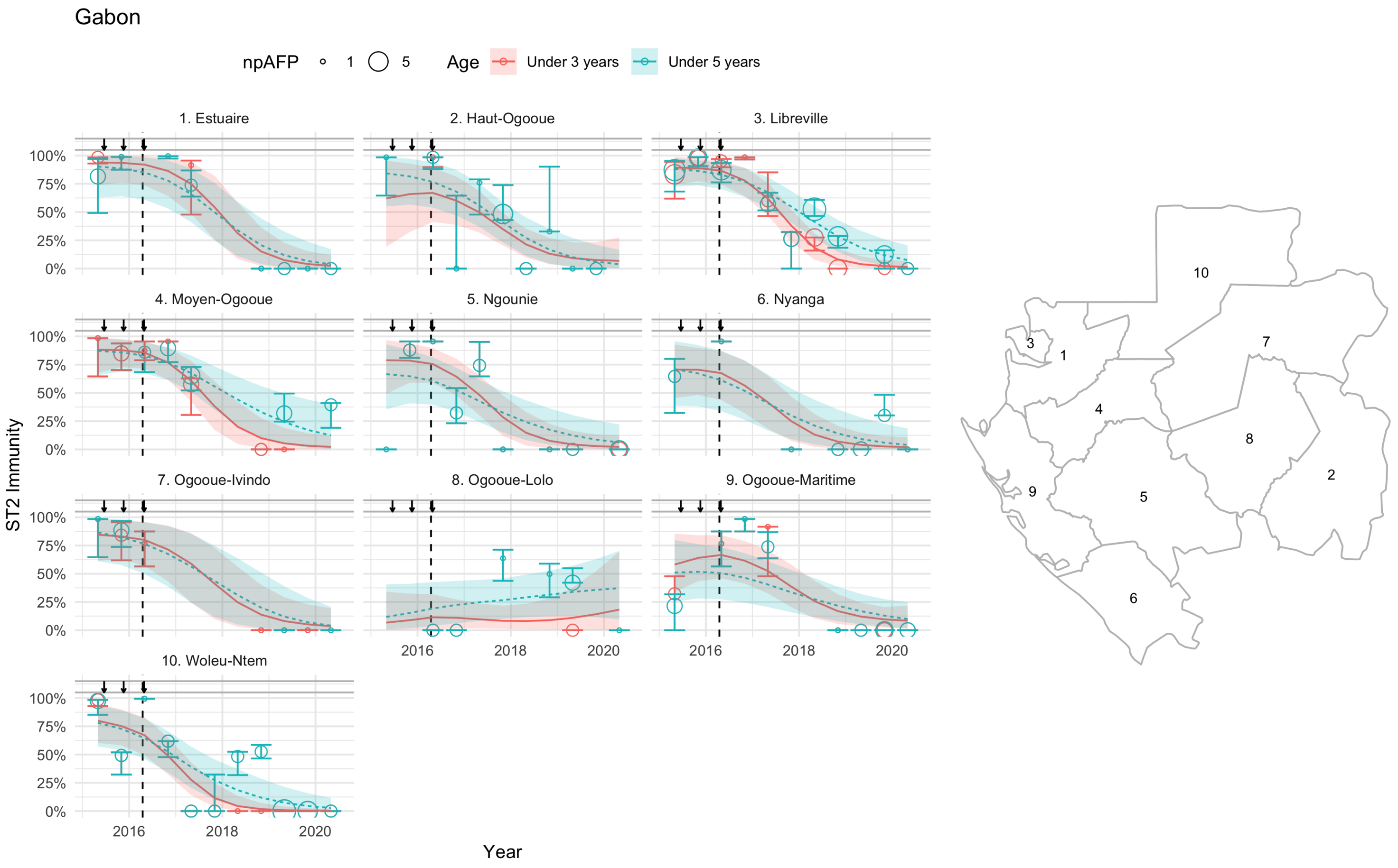

Figure S79: Type 2 population immunity from OPV in each province of Gabon in children under five (blue) and under three (red). Circles show median of bootstrapped crude immunity estimates, error bars show 2.5th and 97.5th percentiles of bootstrapped estimates. Size of circles indicate the number of non-polio AFP cases that each crude estimate is based on. Lines show median smoothed immunity estimate, transparent ribbons show 95% credible interval. Arrows show timing of tOPV (before withdrawal, dotted line) or mOPV2 SIAs (after withdrawal, dotted line). Height of arrows should the proportion of under-five population targeted in SIA. The publication of this map does not imply the expression of any opinion whatsoever on the part of WHO concerning the legal status of any territory, city or area or of its authorities, or concerning the delimitation of its frontiers or boundaries.

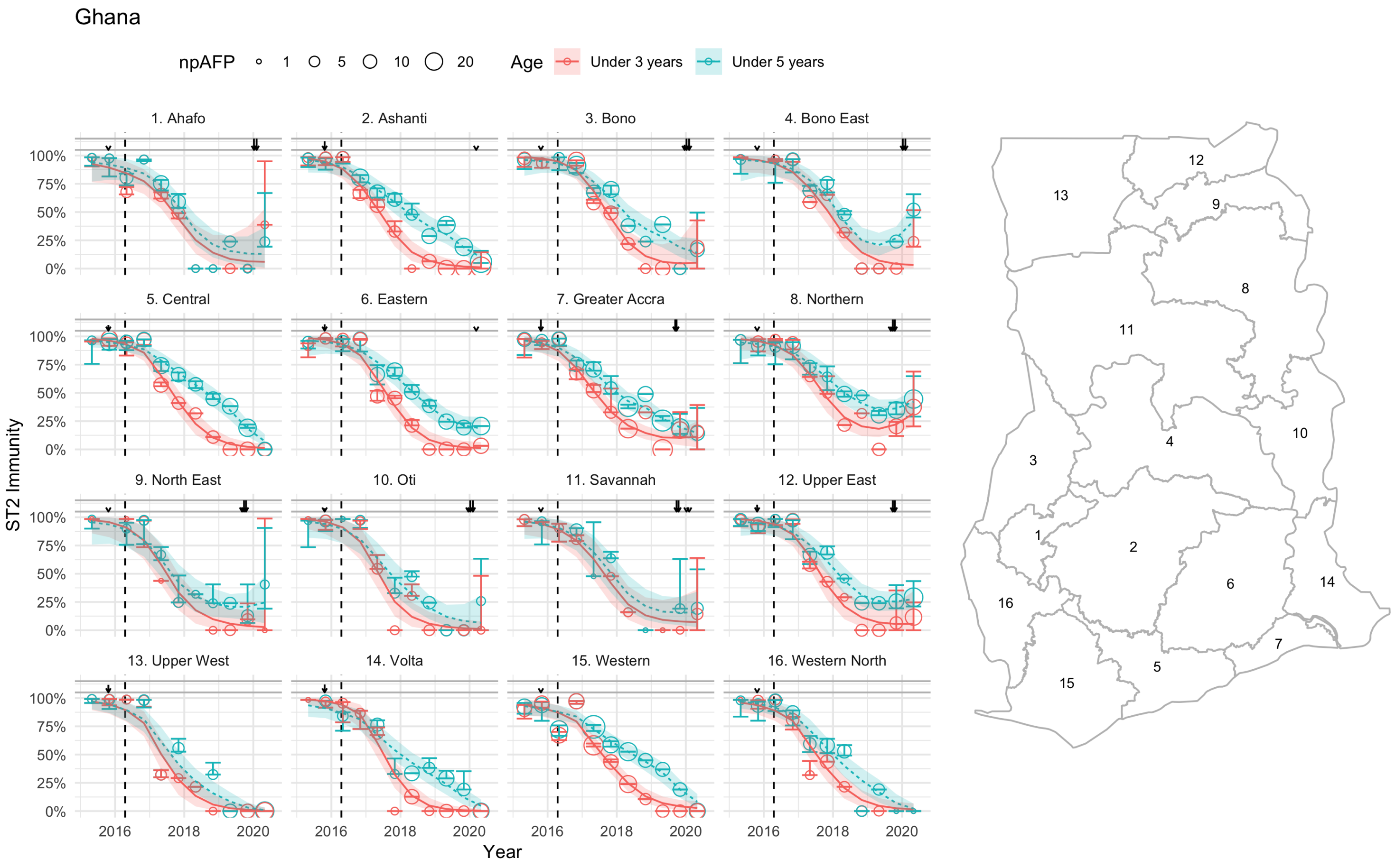

Figure S80: Type 2 population immunity from OPV in each province of Ghana in children under five (blue) and under three (red). Circles show median of bootstrapped crude immunity estimates, error bars show 2.5th and 97.5th percentiles of bootstrapped estimates. Size of circles indicate the number of non-polio AFP cases that each crude estimate is based on. Lines show median smoothed immunity estimate, transparent ribbons show 95% credible interval. Arrows show timing of tOPV (before withdrawal, dotted line) or mOPV2 SIAs (after withdrawal, dotted line). Height of arrows should the proportion of under-five population targeted in SIA. The publication of this map does not imply the expression of any opinion whatsoever on the part of WHO concerning the legal status of any territory, city or area or of its authorities, or concerning the delimitation of its frontiers or boundaries.

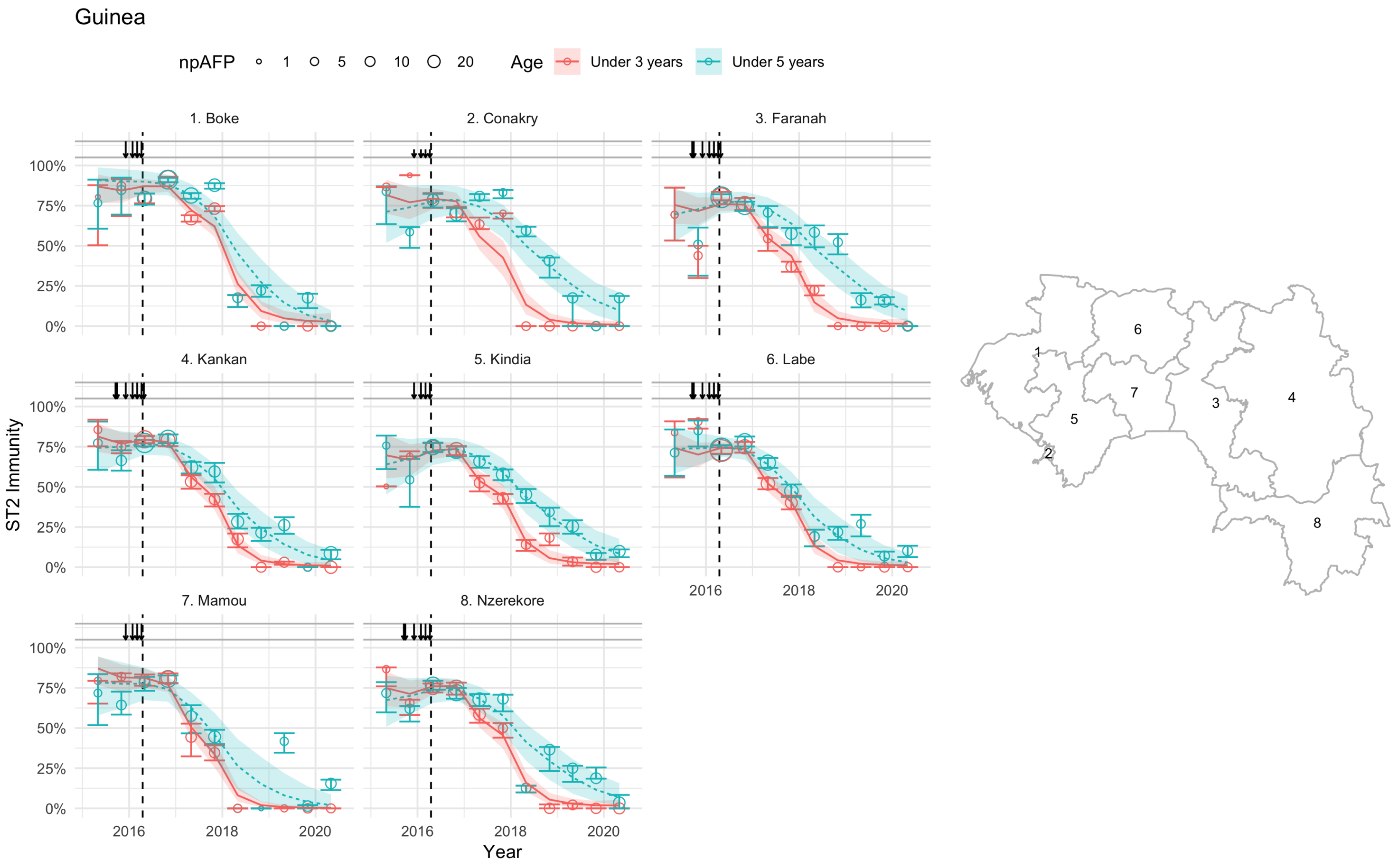

Figure S81: Type 2 population immunity from OPV in each province of Guinea in children under five (blue) and under three (red). Circles show median of bootstrapped crude immunity estimates, error bars show 2.5th and 97.5th percentiles of bootstrapped estimates. Size of circles indicate the number of non-polio AFP cases that each crude estimate is based on. Lines show median smoothed immunity estimate, transparent ribbons show 95% credible interval. Arrows show timing of tOPV (before withdrawal, dotted line) or mOPV2 SIAs (after withdrawal, dotted line). Height of arrows should the proportion of under-five population targeted in SIA. The publication of this map does not imply the expression of any opinion whatsoever on the part of WHO concerning the legal status of any territory, city or area or of its authorities, or concerning the delimitation of its frontiers or boundaries.

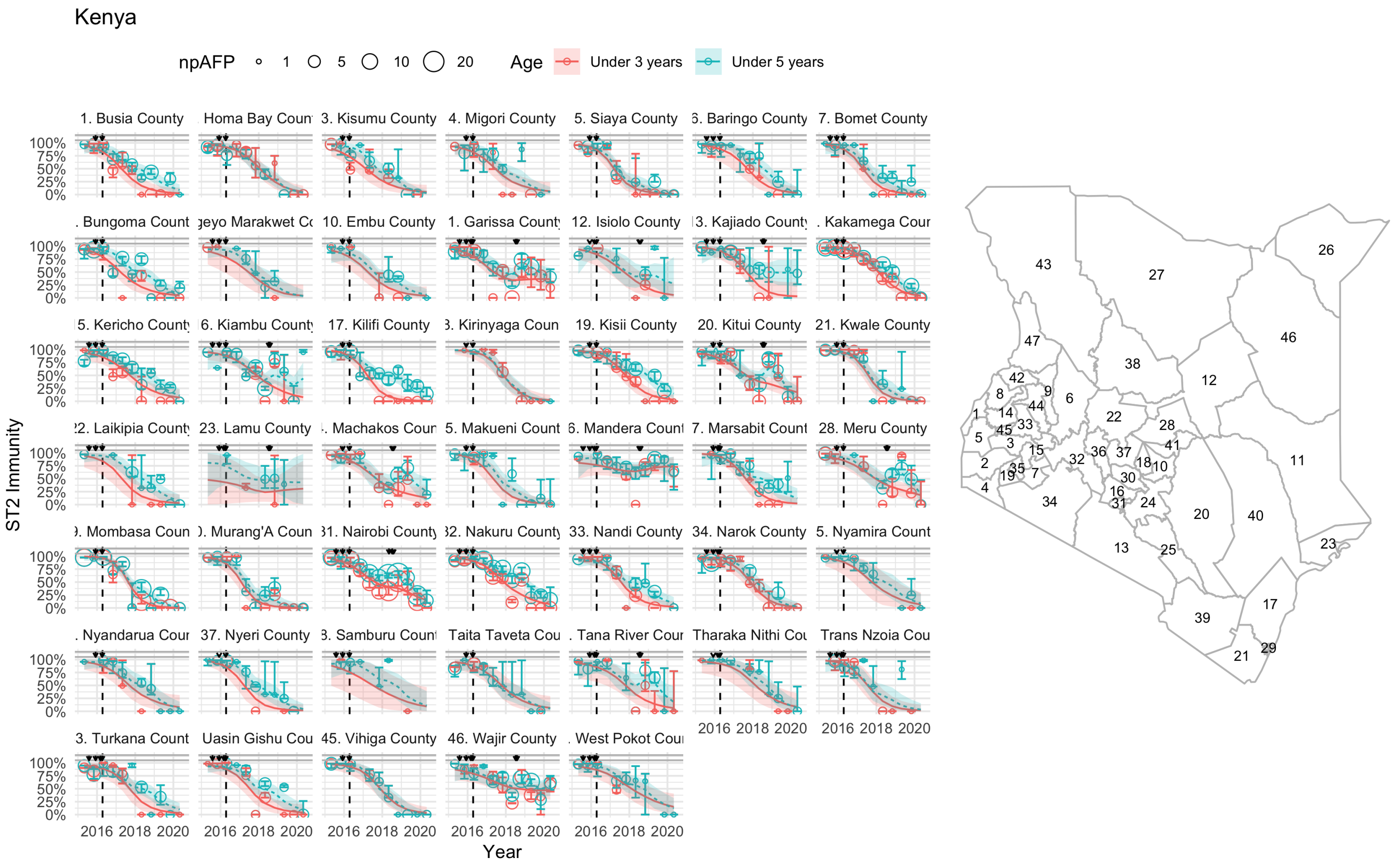

Figure S82: Type 2 population immunity from OPV in each province of Kenya in children under five (blue) and under three (red). Circles show median of bootstrapped crude immunity estimates, error bars show 2.5th and 97.5th percentiles of bootstrapped estimates. Size of circles indicate the number of non-polio AFP cases that each crude estimate is based on. Lines show median smoothed immunity estimate, transparent ribbons show 95% credible interval. Arrows show timing of tOPV (before withdrawal, dotted line) or mOPV2 SIAs (after withdrawal, dotted line). Height of arrows should the proportion of under-five population targeted in SIA. The publication of this map does not imply the expression of any opinion whatsoever on the part of WHO concerning the legal status of any territory, city or area or of its authorities, or concerning the delimitation of its frontiers or boundaries.

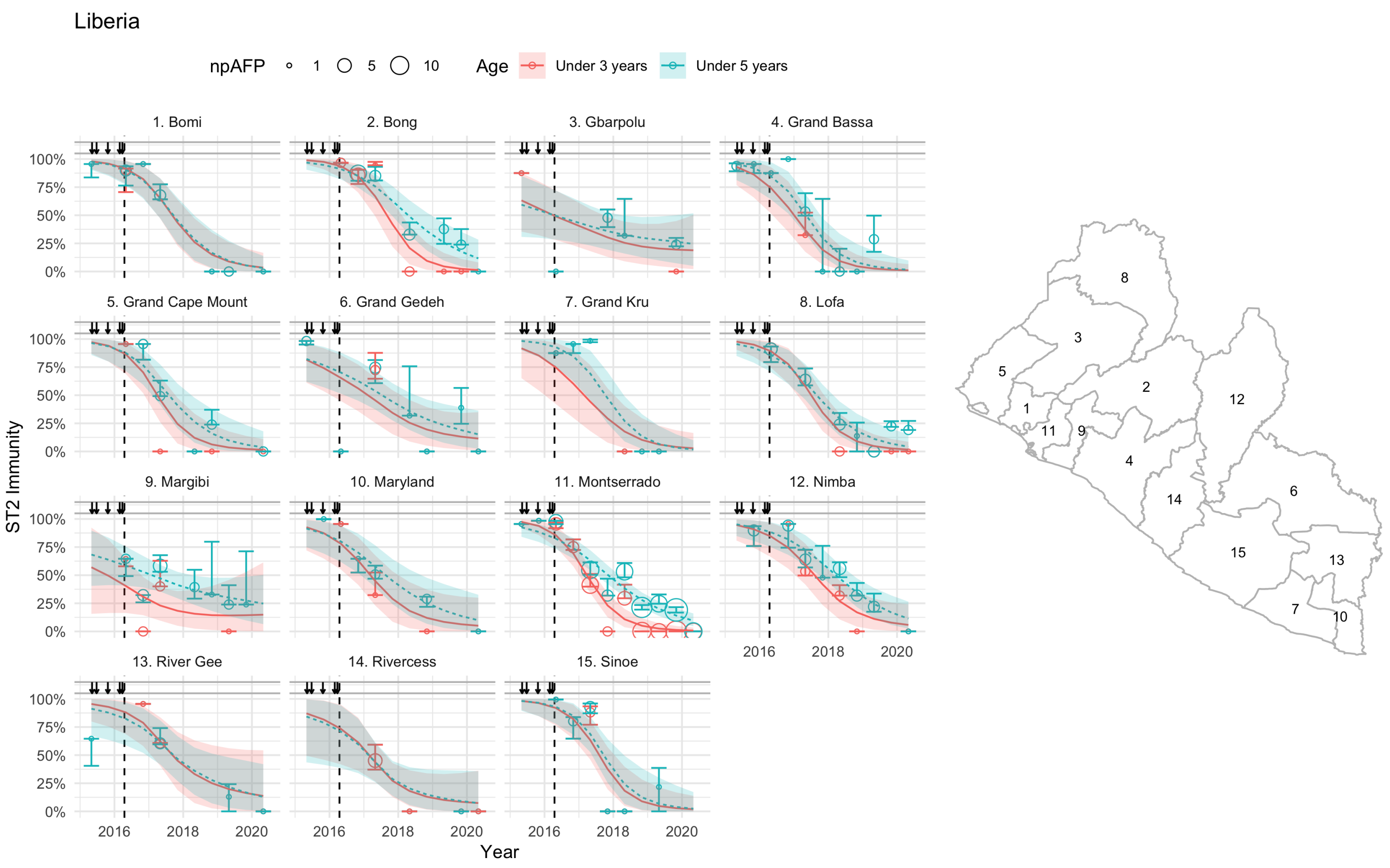

Figure S83: Type 2 population immunity from OPV in each province of Liberia in children under five (blue) and under three (red). Circles show median of bootstrapped crude immunity estimates, error bars show 2.5th and 97.5th percentiles of bootstrapped estimates. Size of circles indicate the number of non-polio AFP cases that each crude estimate is based on. Lines show median smoothed immunity estimate, transparent ribbons show 95% credible interval. Arrows show timing of tOPV (before withdrawal, dotted line) or mOPV2 SIAs (after withdrawal, dotted line). Height of arrows should the proportion of under-five population targeted in SIA. The publication of this map does not imply the expression of any opinion whatsoever on the part of WHO concerning the legal status of any territory, city or area or of its authorities, or concerning the delimitation of its frontiers or boundaries.

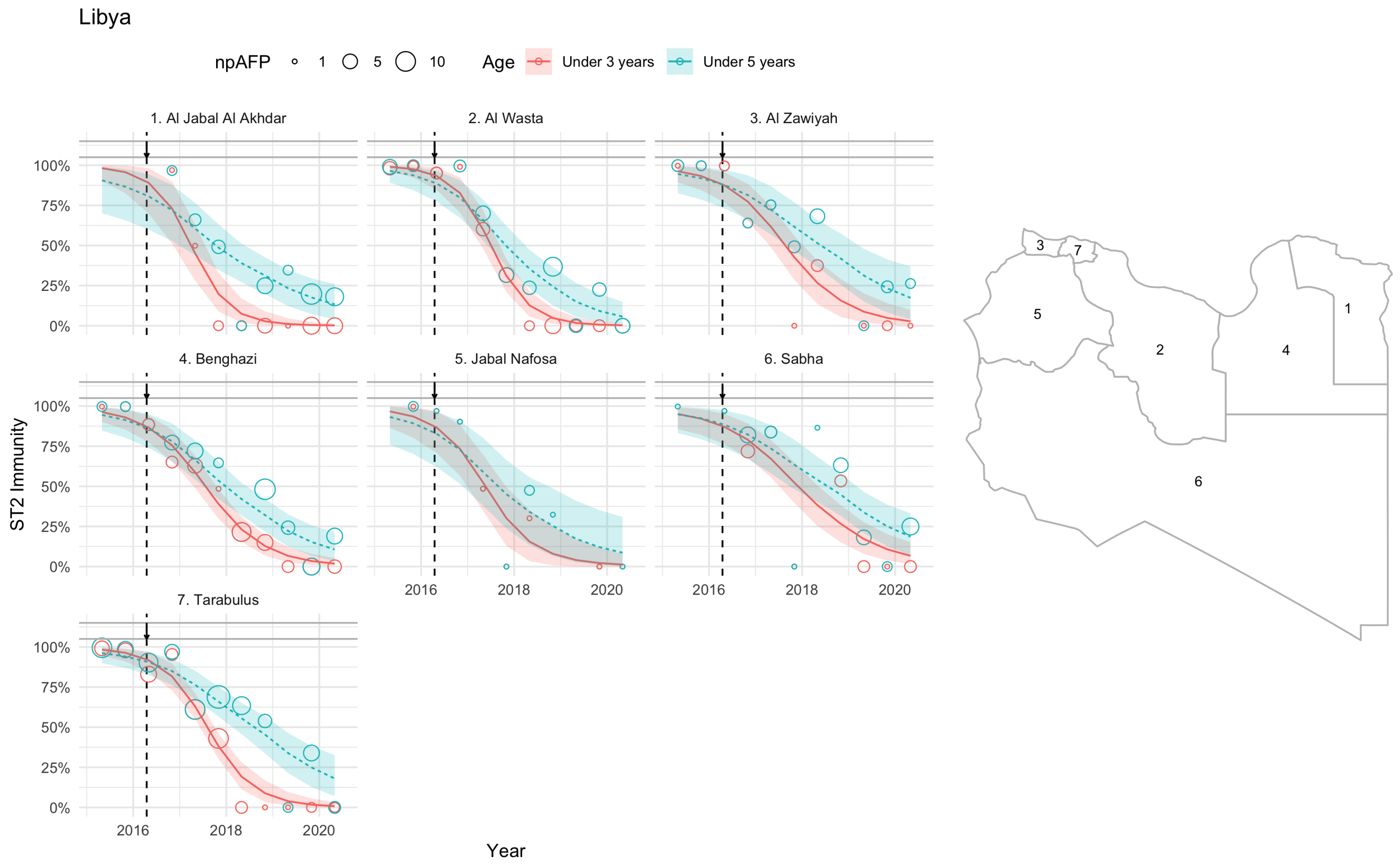

Figure S84: Type 2 population immunity from OPV in each province of Libya in children under five (blue) and under three (red). Circles show median of bootstrapped crude immunity estimates, error bars show 2.5th and 97.5th percentiles of bootstrapped estimates. Size of circles indicate the number of non-polio AFP cases that each crude estimate is based on. Lines show median smoothed immunity estimate, transparent ribbons show 95% credible interval. Arrows show timing of tOPV (before withdrawal, dotted line) or mOPV2 SIAs (after withdrawal, dotted line). Height of arrows should the proportion of under-five population targeted in SIA. The publication of this map does not imply the expression of any opinion whatsoever on the part of WHO concerning the legal status of any territory, city or area or of its authorities, or concerning the delimitation of its frontiers or boundaries.

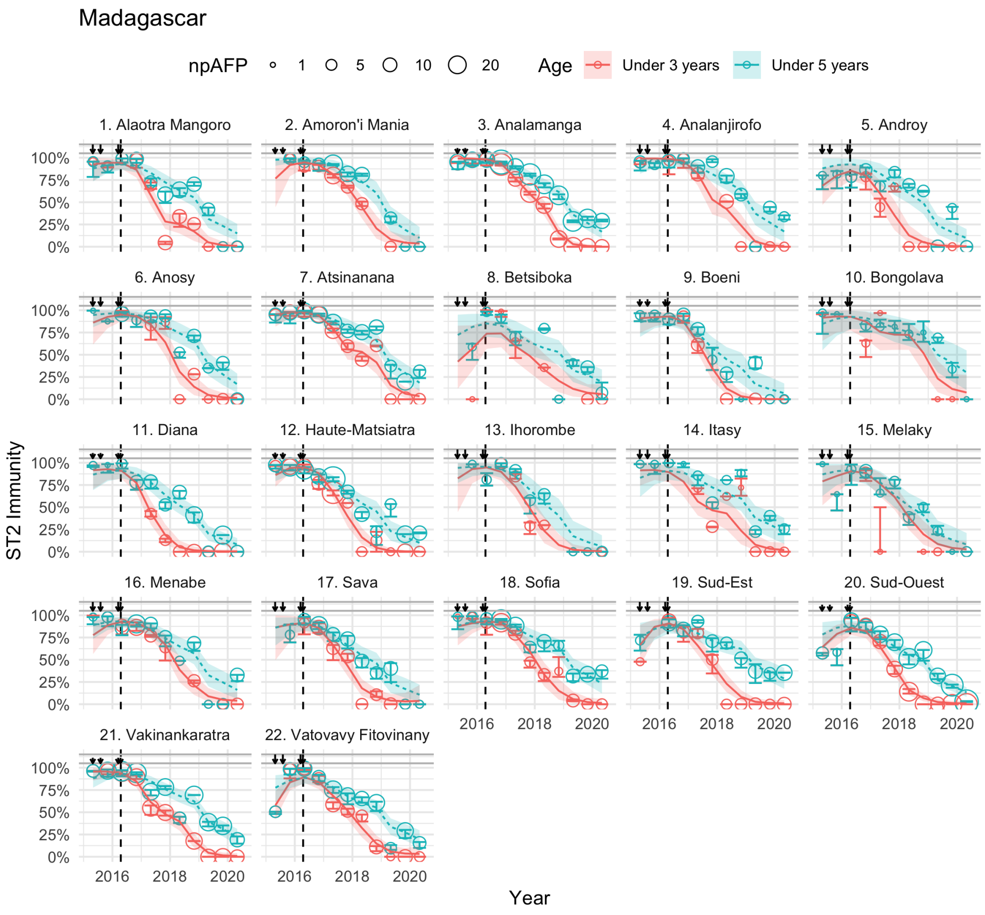

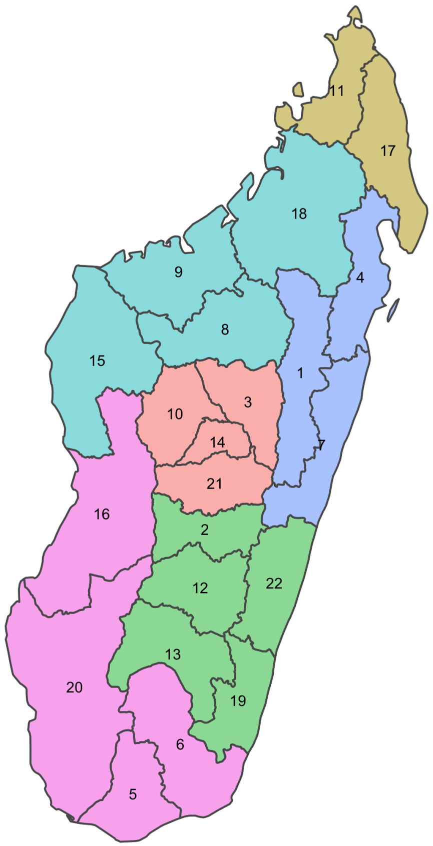

Figure S85: Type 2 population immunity from OPV in each province of Madagascar in children under five (blue) and under three (red). Circles show median of bootstrapped crude immunity estimates, error bars show 2.5th and 97.5th percentiles of bootstrapped estimates. Size of circles indicate the number of non-polio AFP cases that each crude estimate is based on. Lines show median smoothed immunity estimate, transparent ribbons show 95% credible interval. Arrows show timing of tOPV (before withdrawal, dotted line) or mOPV2 SIAs (after withdrawal, dotted line). Height of arrows should the proportion of under-five population targeted in SIA. Shaded areas on map indicate grouped provinces. The publication of this map does not imply the expression of any opinion whatsoever on the part of WHO concerning the legal status of any territory, city or area or of its authorities, or concerning the delimitation of its frontiers or boundaries.

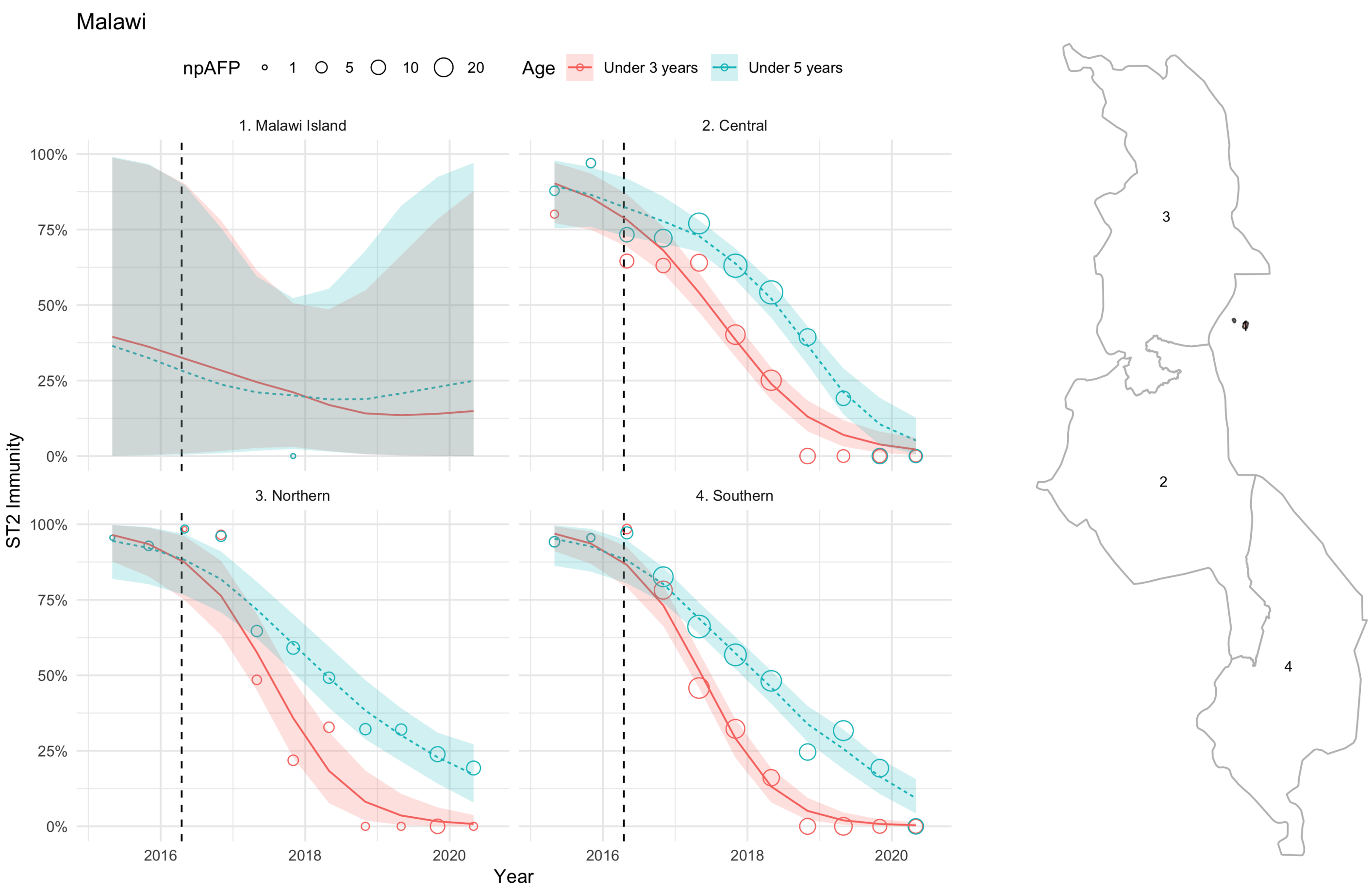

Figure S86: Type 2 population immunity from OPV in each province of Malawi in children under five (blue) and under three (red). Circles show median of bootstrapped crude immunity estimates, error bars show 2.5th and 97.5th percentiles of bootstrapped estimates. Size of circles indicate the number of non-polio AFP cases that each crude estimate is based on. Lines show median smoothed immunity estimate, transparent ribbons show 95% credible interval. Arrows show timing of tOPV (before withdrawal, dotted line) or mOPV2 SIAs (after withdrawal, dotted line). Height of arrows should the proportion of under-five population targeted in SIA. Shaded areas on map indicate grouped provinces. The publication of this map does not imply the expression of any opinion whatsoever on the part of WHO concerning the legal status of any territory, city or area or of its authorities, or concerning the delimitation of its frontiers or boundaries.

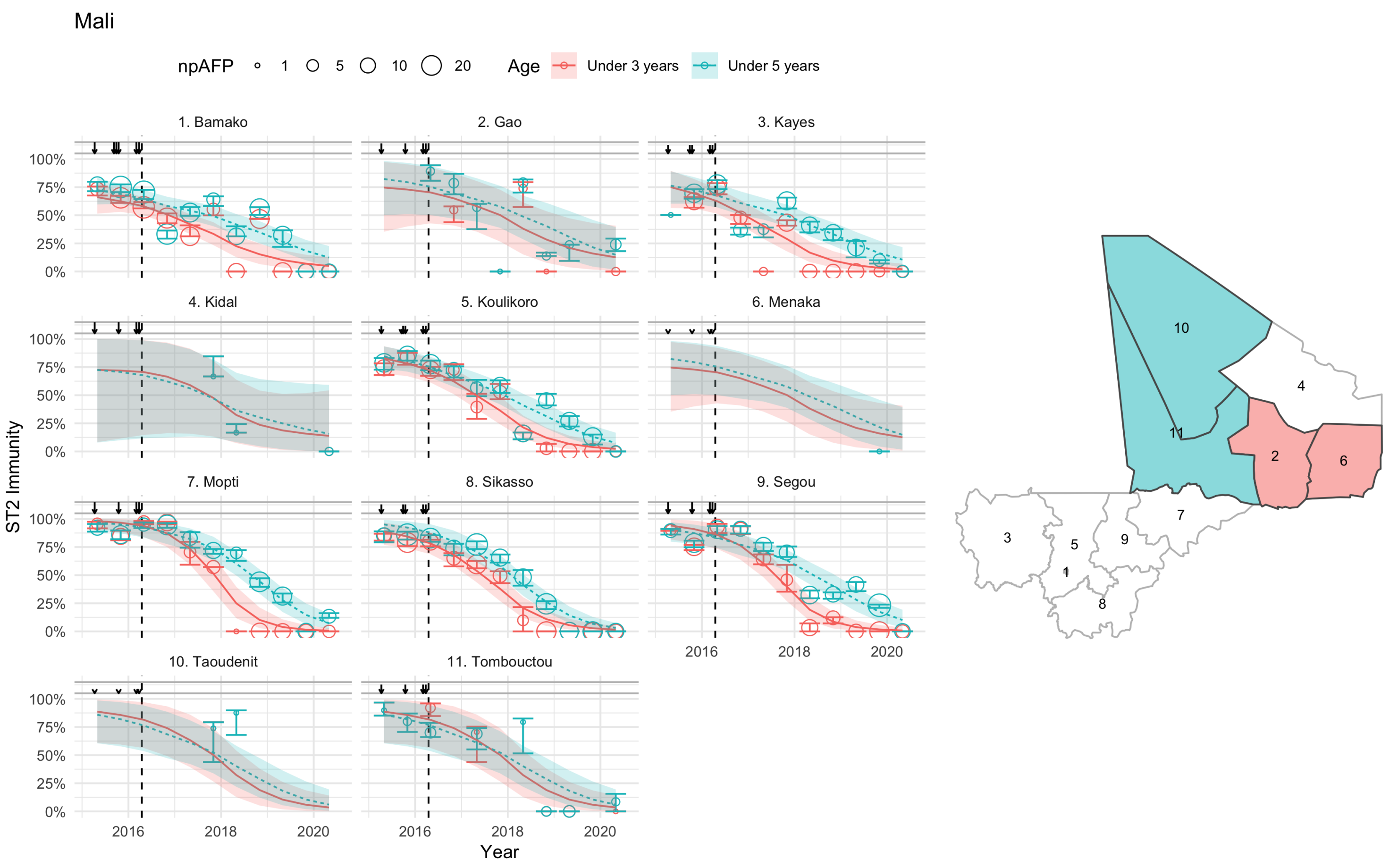

Figure S87: Type 2 population immunity from OPV in each province of Mali in children under five (blue) and under three (red). Circles show median of bootstrapped crude immunity estimates, error bars show 2.5th and 97.5th percentiles of bootstrapped estimates. Size of circles indicate the number of non-polio AFP cases that each crude estimate is based on. Lines show median smoothed immunity estimate, transparent ribbons show 95% credible interval. Arrows show timing of tOPV (before withdrawal, dotted line) or mOPV2 SIAs (after withdrawal, dotted line). Height of arrows should the proportion of under-five population targeted in SIA. Shaded areas on map indicate grouped provinces. The publication of this map does not imply the expression of any opinion whatsoever on the part of WHO concerning the legal status of any territory, city or area or of its authorities, or concerning the delimitation of its frontiers or boundaries.

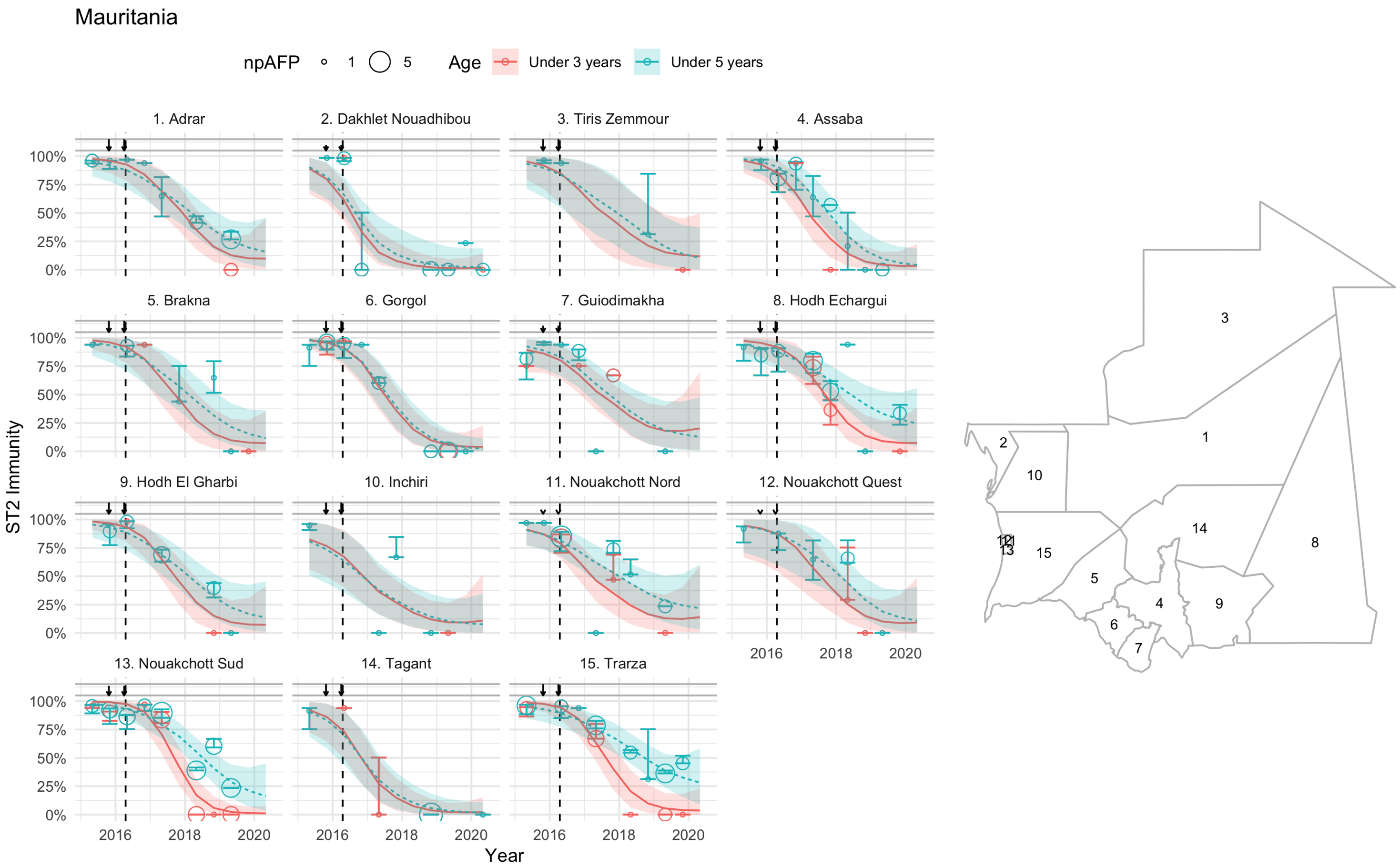

Figure S88: Type 2 population immunity from OPV in each province of Mauritania in children under five (blue) and under three (red). Circles show median of bootstrapped crude immunity estimates, error bars show 2.5th and 97.5th percentiles of bootstrapped estimates. Size of circles indicate the number of non-polio AFP cases that each crude estimate is based on. Lines show median smoothed immunity estimate, transparent ribbons show 95% credible interval. Arrows show timing of tOPV (before withdrawal, dotted line) or mOPV2 SIAs (after withdrawal, dotted line). Height of arrows should the proportion of under-five population targeted in SIA. The publication of this map does not imply the expression of any opinion whatsoever on the part of WHO concerning the legal status of any territory, city or area or of its authorities, or concerning the delimitation of its frontiers or boundaries.

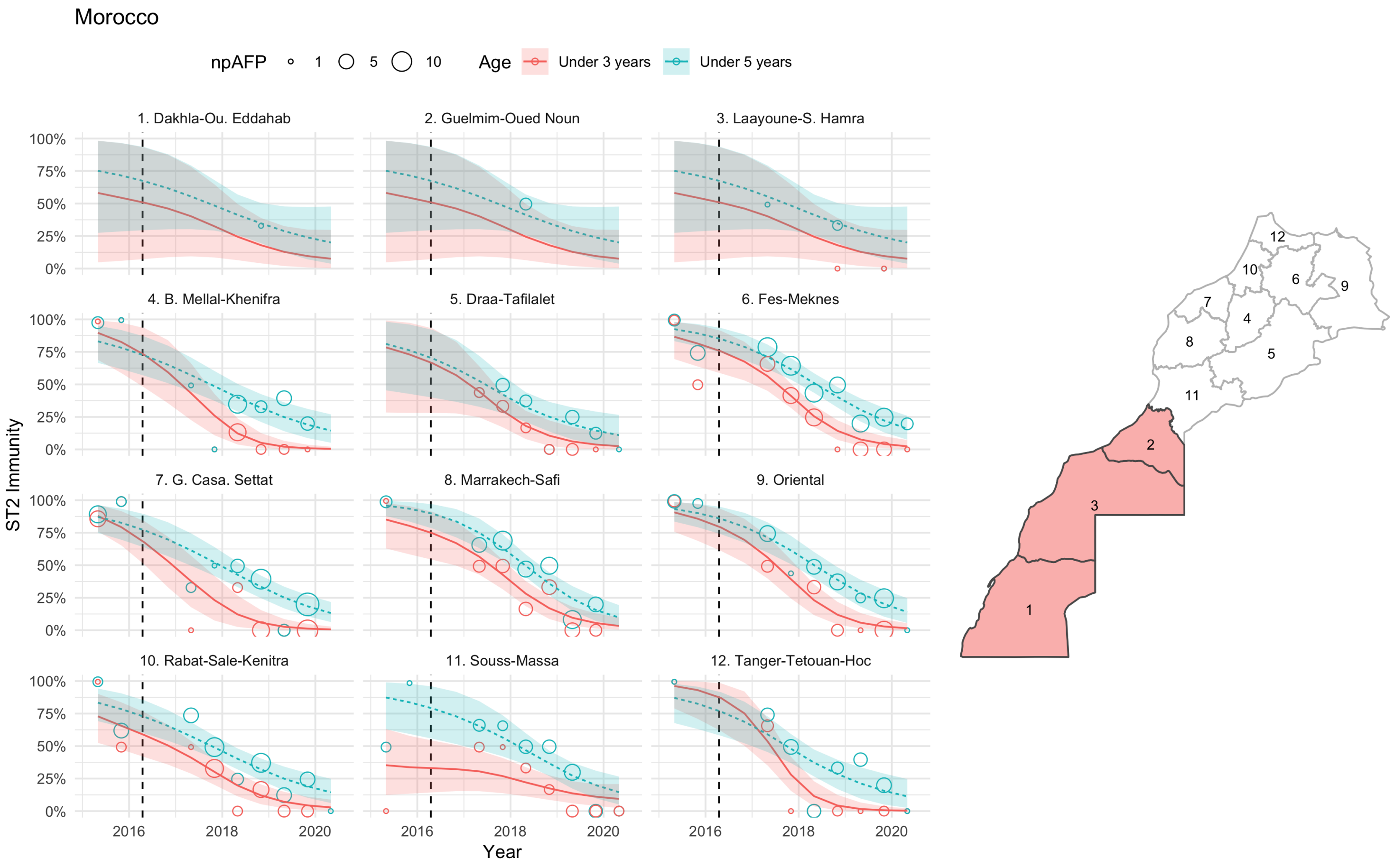

Figure S89: Type 2 population immunity from OPV in each province of Morocco in children under five (blue) and under three (red). Circles show median of bootstrapped crude immunity estimates, error bars show 2.5th and 97.5th percentiles of bootstrapped estimates. Size of circles indicate the number of non-polio AFP cases that each crude estimate is based on. Lines show median smoothed immunity estimate, transparent ribbons show 95% credible interval. Arrows show timing of tOPV (before withdrawal, dotted line) or mOPV2 SIAs (after withdrawal, dotted line). Height of arrows should the proportion of under-five population targeted in SIA. Shaded areas on map indicate grouped provinces. The publication of this map does not imply the expression of any opinion whatsoever on the part of WHO concerning the legal status of any territory, city or area or of its authorities, or concerning the delimitation of its frontiers or boundaries.

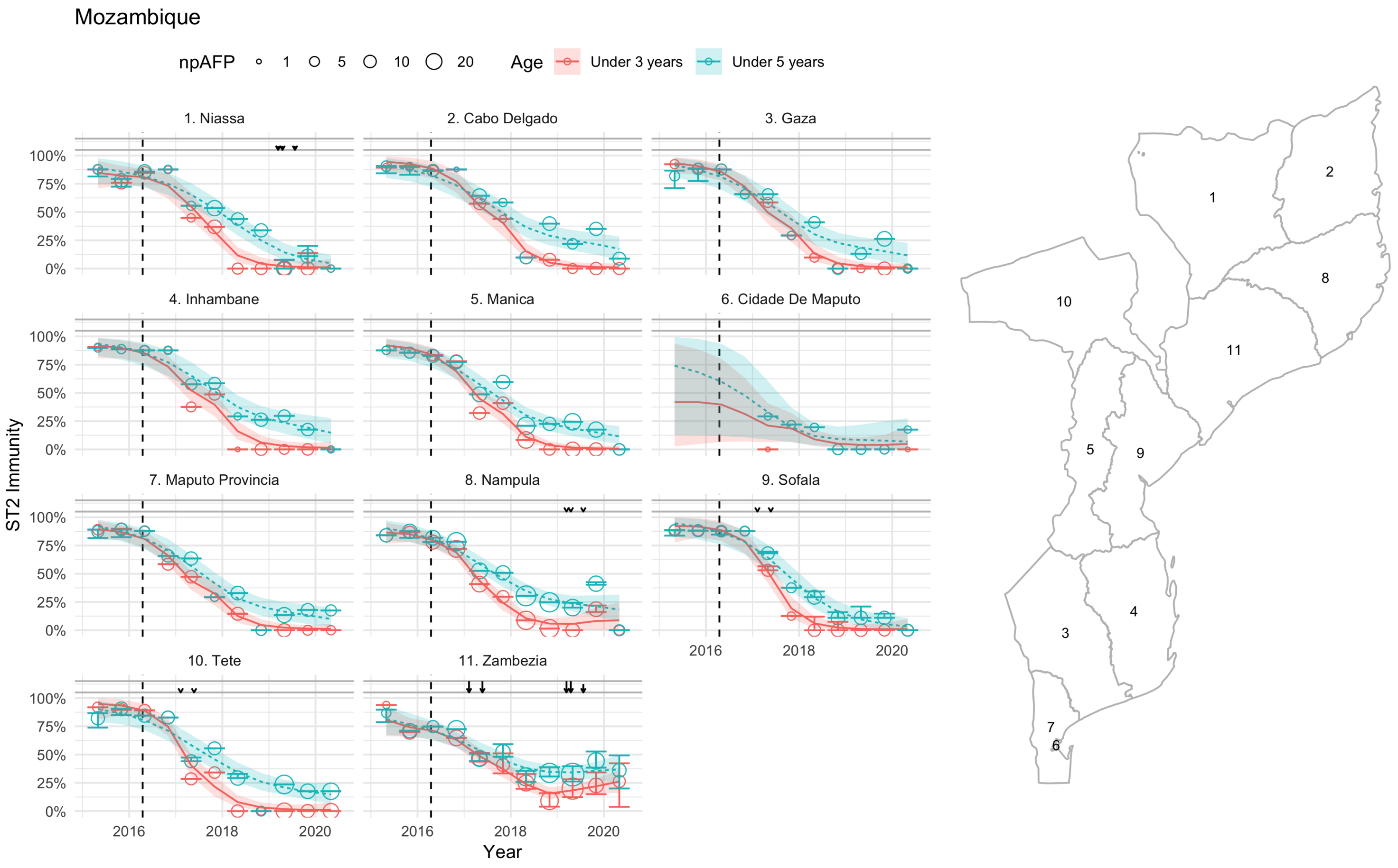

Figure S90: Type 2 population immunity from OPV in each province of Mozambique in children under five (blue) and under three (red). Circles show median of bootstrapped crude immunity estimates, error bars show 2.5th and 97.5th percentiles of bootstrapped estimates. Size of circles indicate the number of non-polio AFP cases that each crude estimate is based on. Lines show median smoothed immunity estimate, transparent ribbons show 95% credible interval. Arrows show timing of tOPV (before withdrawal, dotted line) or mOPV2 SIAs (after withdrawal, dotted line). Height of arrows should the proportion of under-five population targeted in SIA. Shaded areas on map indicate grouped provinces. The publication of this map does not imply the expression of any opinion whatsoever on the part of WHO concerning the legal status of any territory, city or area or of its authorities, or concerning the delimitation of its frontiers or boundaries.

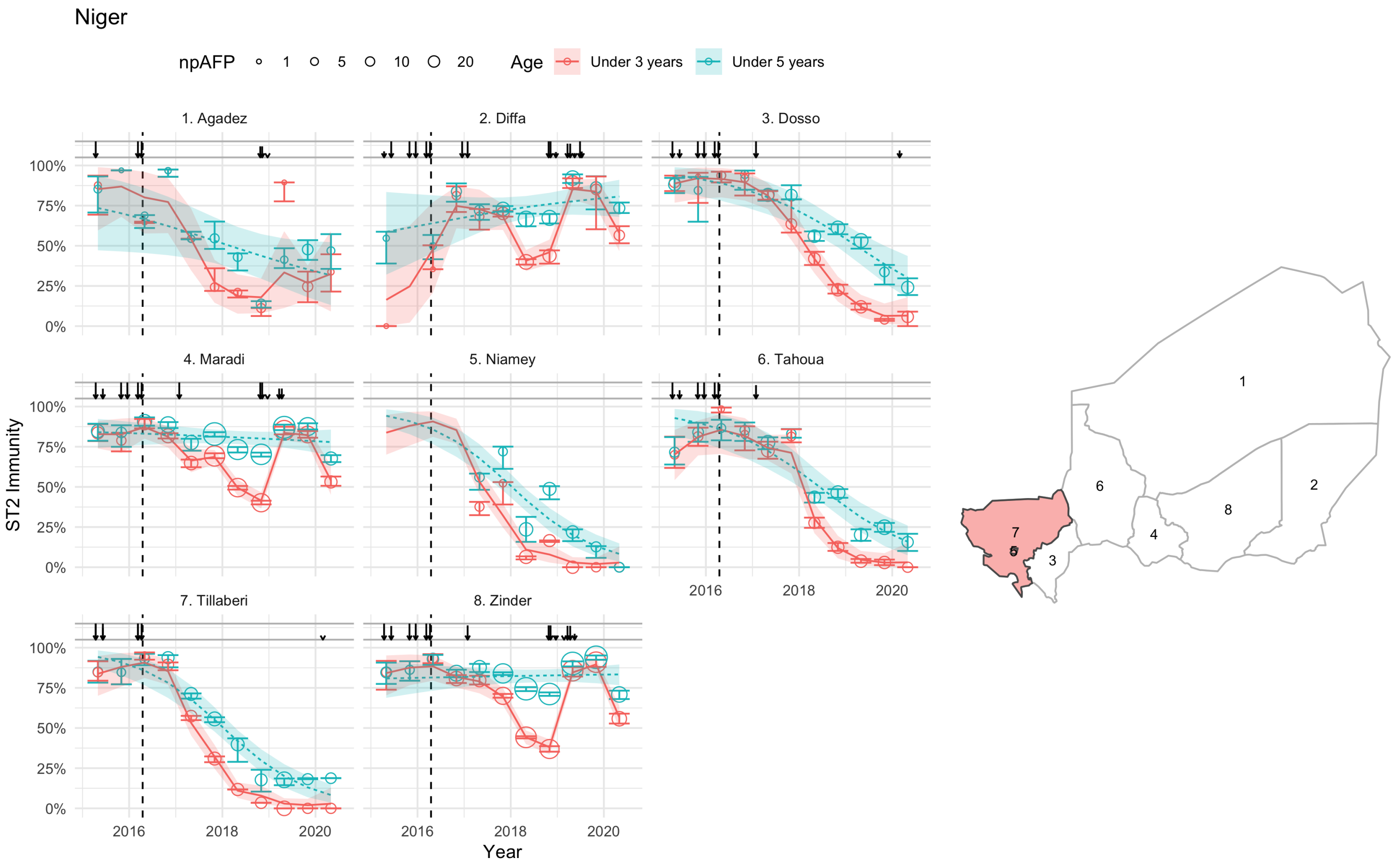

Figure S91: Type 2 population immunity from OPV in each province of Niger in children under five (blue) and under three (red). Circles show median of bootstrapped crude immunity estimates, error bars show 2.5th and 97.5th percentiles of bootstrapped estimates. Size of circles indicate the number of non-polio AFP cases that each crude estimate is based on. Lines show median smoothed immunity estimate, transparent ribbons show 95% credible interval. Arrows show timing of tOPV (before withdrawal, dotted line) or mOPV2 SIAs (after withdrawal, dotted line). Height of arrows should the proportion of under-five population targeted in SIA. Shaded areas on map indicate grouped provinces. The publication of this map does not imply the expression of any opinion whatsoever on the part of WHO concerning the legal status of any territory, city or area or of its authorities, or concerning the delimitation of its frontiers or boundaries.

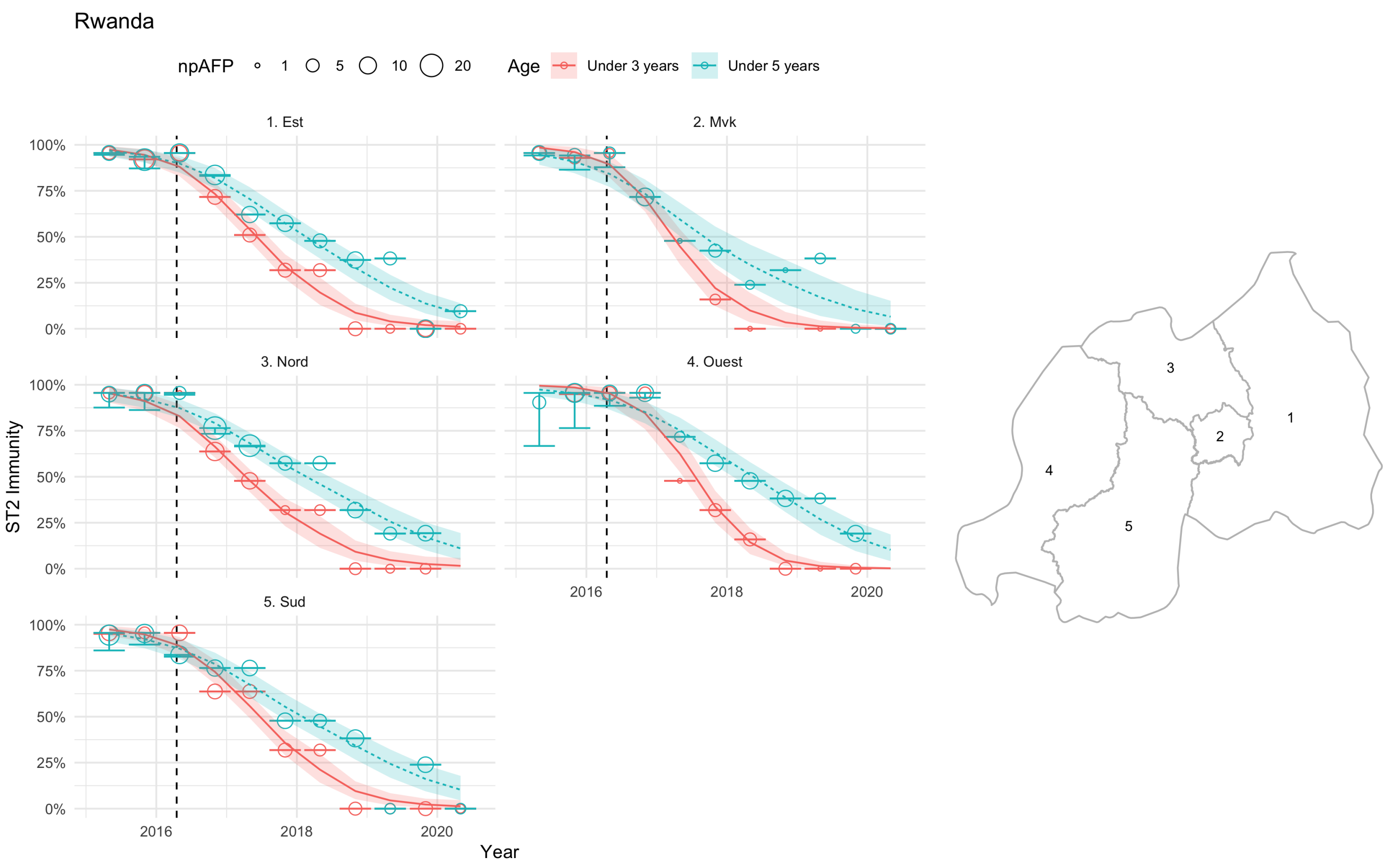

Figure S92: Type 2 population immunity from OPV in each province of Rwanda in children under five (blue) and under three (red). Circles show median of bootstrapped crude immunity estimates, error bars show 2.5th and 97.5th percentiles of bootstrapped estimates. Size of circles indicate the number of non-polio AFP cases that each crude estimate is based on. Lines show median smoothed immunity estimate, transparent ribbons show 95% credible interval. Arrows show timing of tOPV (before withdrawal, dotted line) or mOPV2 SIAs (after withdrawal, dotted line). Height of arrows should the proportion of under-five population targeted in SIA. The publication of this map does not imply the expression of any opinion whatsoever on the part of WHO concerning the legal status of any territory, city or area or of its authorities, or concerning the delimitation of its frontiers or boundaries.

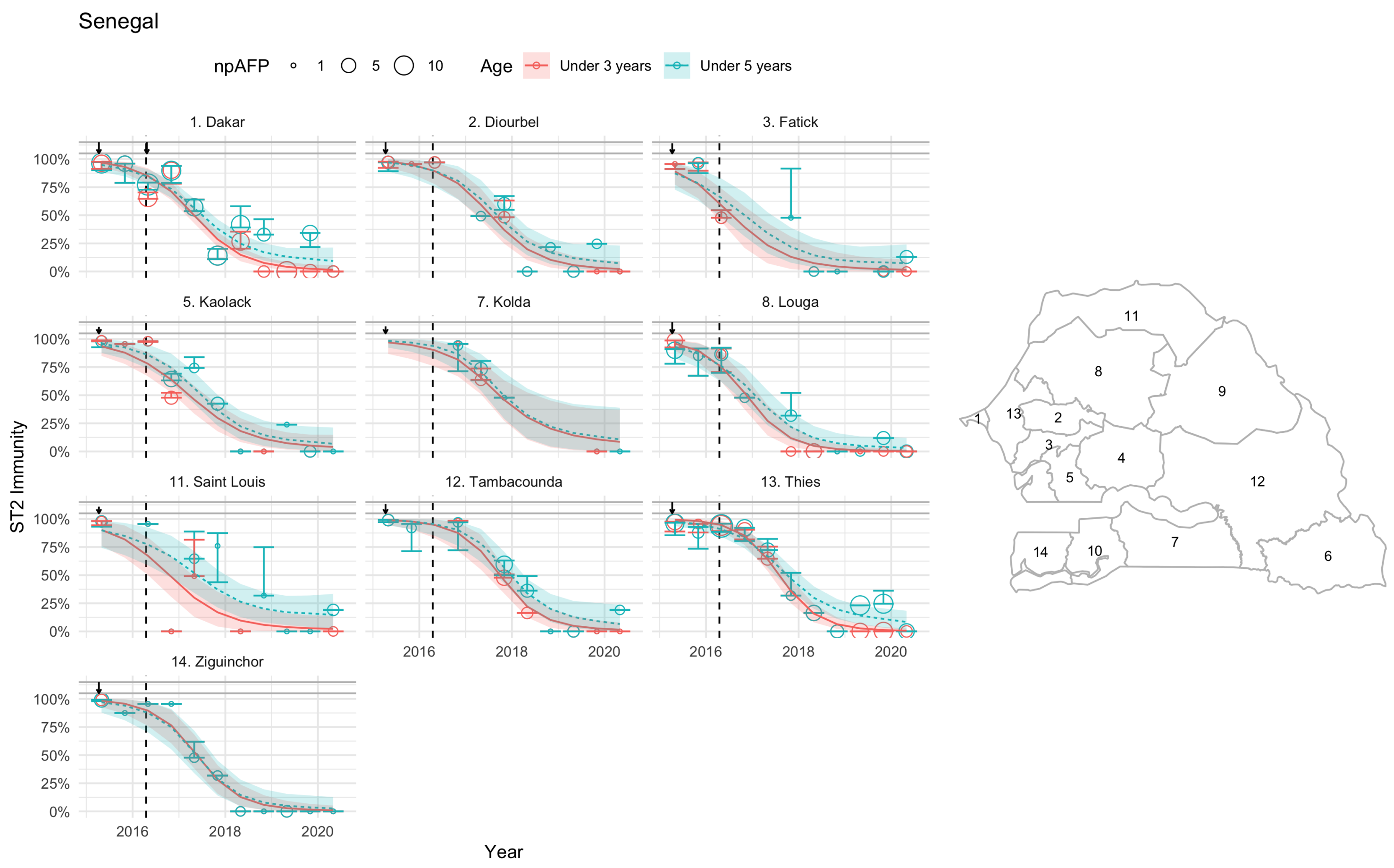

Figure S93: Type 2 population immunity from OPV in each province of Senegal in children under five (blue) and under three (red). Circles show median of bootstrapped crude immunity estimates, error bars show 2.5th and 97.5th percentiles of bootstrapped estimates. Size of circles indicate the number of non-polio AFP cases that each crude estimate is based on. Lines show median smoothed immunity estimate, transparent ribbons show 95% credible interval. Arrows show timing of tOPV (before withdrawal, dotted line) or mOPV2 SIAs (after withdrawal, dotted line). Height of arrows should the proportion of under-five population targeted in SIA. The publication of this map does not imply the expression of any opinion whatsoever on the part of WHO concerning the legal status of any territory, city or area or of its authorities, or concerning the delimitation of its frontiers or boundaries.

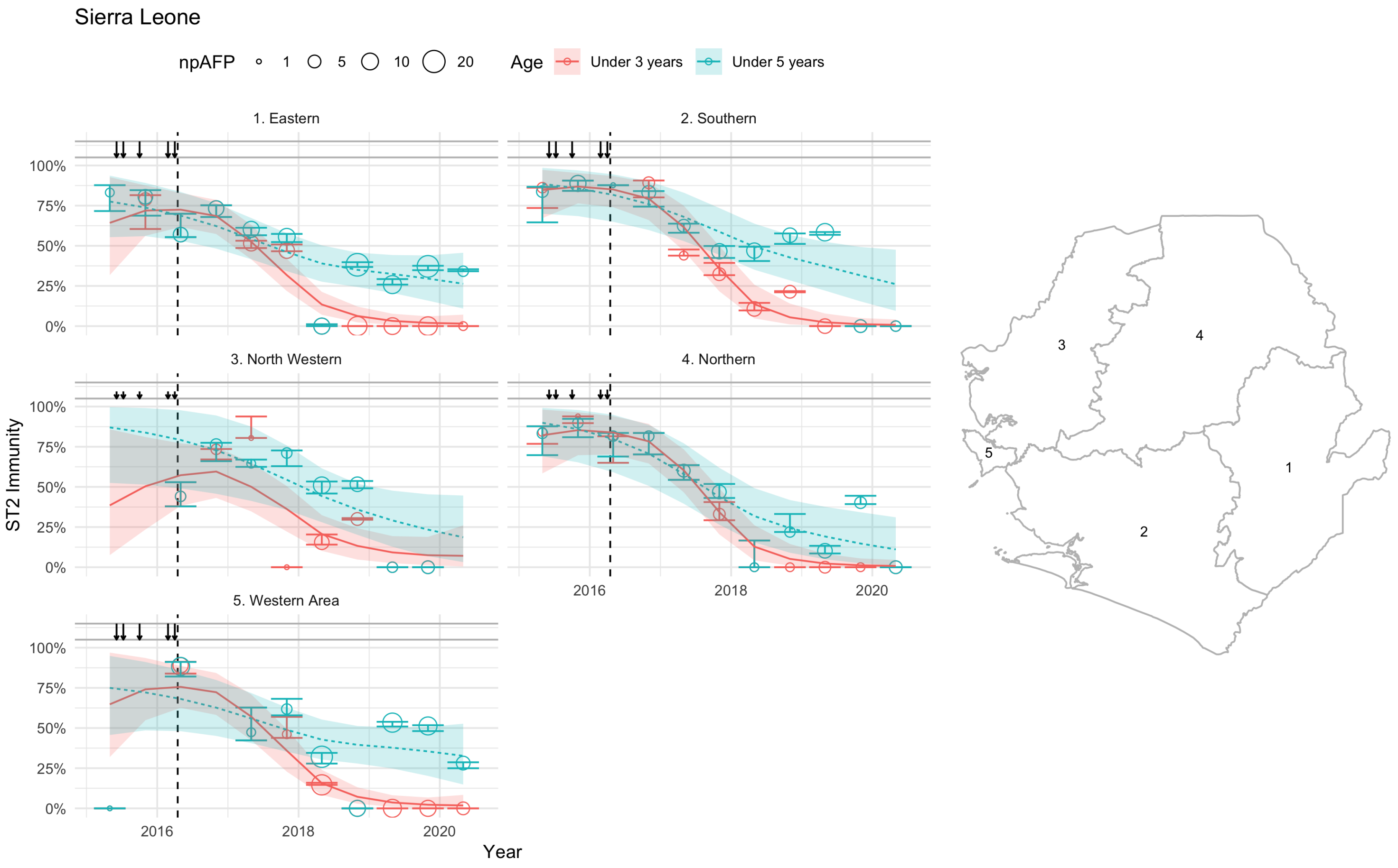

Figure S94: Type 2 population immunity from OPV in each province of Sierra Leone in children under five (blue) and under three (red). Circles show median of bootstrapped crude immunity estimates, error bars show 2.5th and 97.5th percentiles of bootstrapped estimates. Size of circles indicate the number of non-polio AFP cases that each crude estimate is based on. Lines show median smoothed immunity estimate, transparent ribbons show 95% credible interval. Arrows show timing of tOPV (before withdrawal, dotted line) or mOPV2 SIAs (after withdrawal, dotted line). Height of arrows should the proportion of under-five population targeted in SIA. The publication of this map does not imply the expression of any opinion whatsoever on the part of WHO concerning the legal status of any territory, city or area or of its authorities, or concerning the delimitation of its frontiers or boundaries.

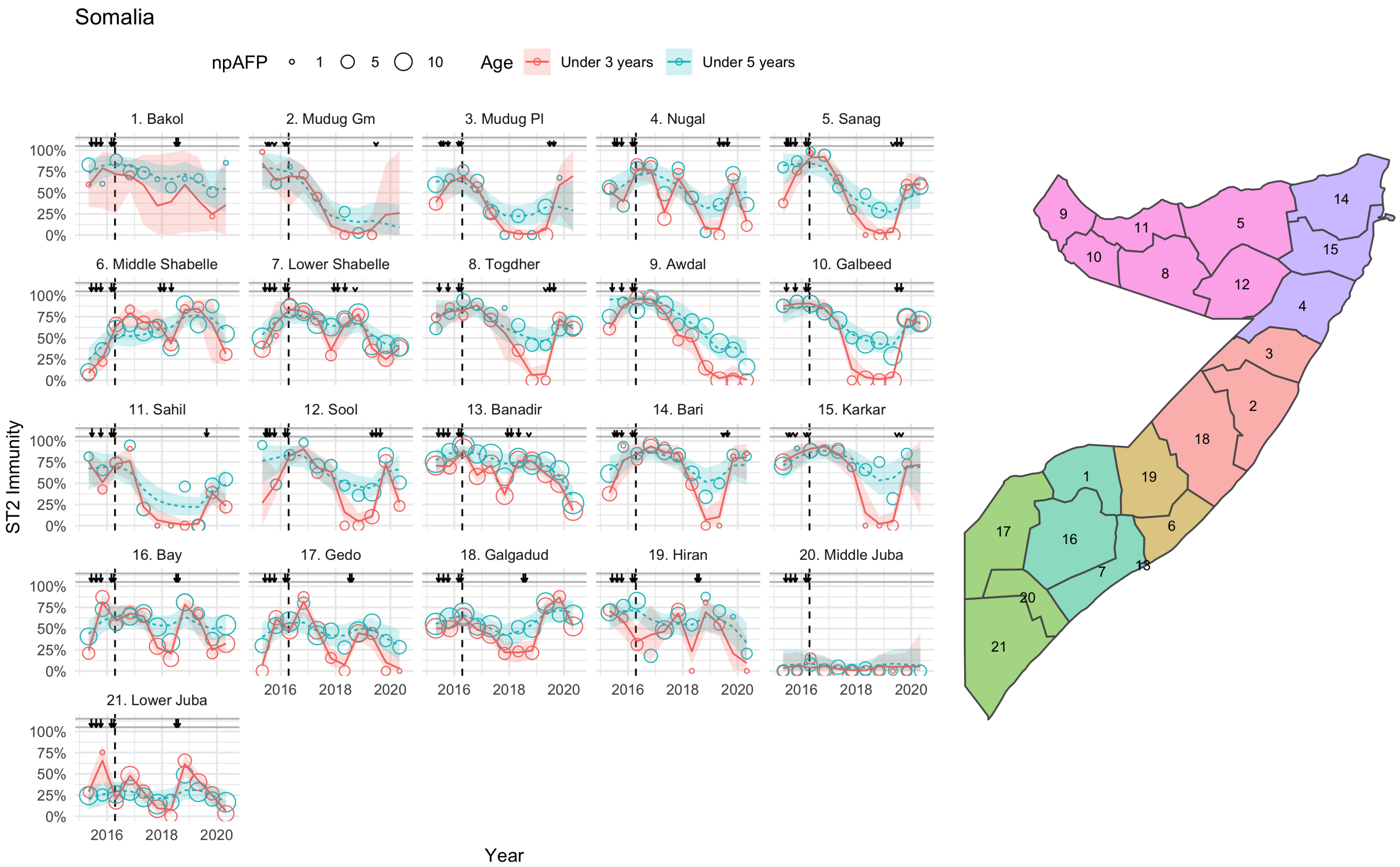

Figure S95: Type 2 population immunity from OPV in each province of Somalia in children under five (blue) and under three (red). Circles show median of bootstrapped crude immunity estimates, error bars show 2.5th and 97.5th percentiles of bootstrapped estimates. Size of circles indicate the number of non-polio AFP cases that each crude estimate is based on. Lines show median smoothed immunity estimate, transparent ribbons show 95% credible interval. Arrows show timing of tOPV (before withdrawal, dotted line) or mOPV2 SIAs (after withdrawal, dotted line). Height of arrows should the proportion of under-five population targeted in SIA. Shaded areas on map indicate grouped provinces. The publication of this map does not imply the expression of any opinion whatsoever on the part of WHO concerning the legal status of any territory, city or area or of its authorities, or concerning the delimitation of its frontiers or boundaries.

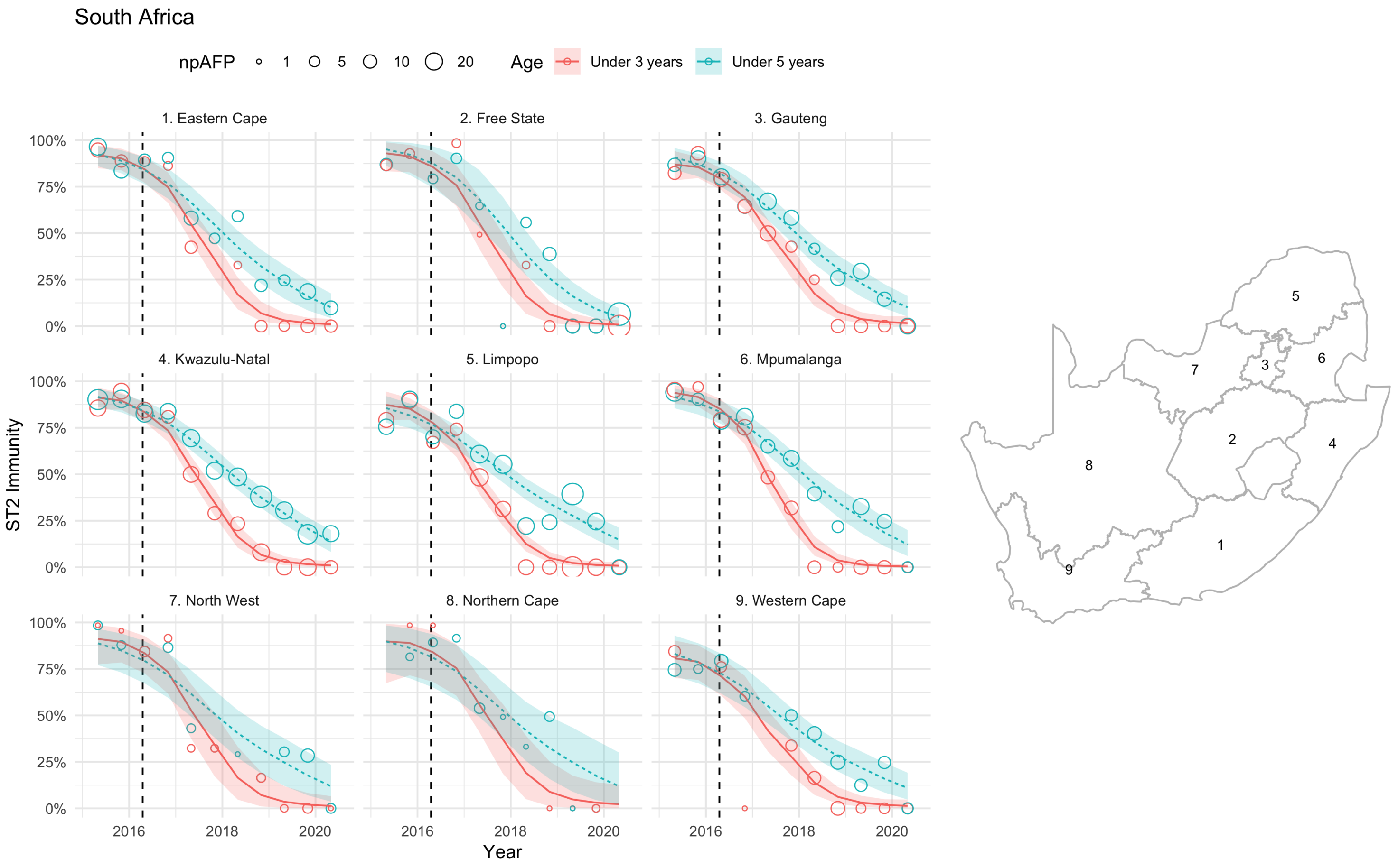

Figure S96: Type 2 population immunity from OPV in each province of South Africa in children under five (blue) and under three (red). Circles show median of bootstrapped crude immunity estimates, error bars show 2.5th and 97.5th percentiles of bootstrapped estimates. Size of circles indicate the number of non-polio AFP cases that each crude estimate is based on. Lines show median smoothed immunity estimate, transparent ribbons show 95% credible interval. Arrows show timing of tOPV (before withdrawal, dotted line) or mOPV2 SIAs (after withdrawal, dotted line). Height of arrows should the proportion of under-five population targeted in SIA. The publication of this map does not imply the expression of any opinion whatsoever on the part of WHO concerning the legal status of any territory, city or area or of its authorities, or concerning the delimitation of its frontiers or boundaries.

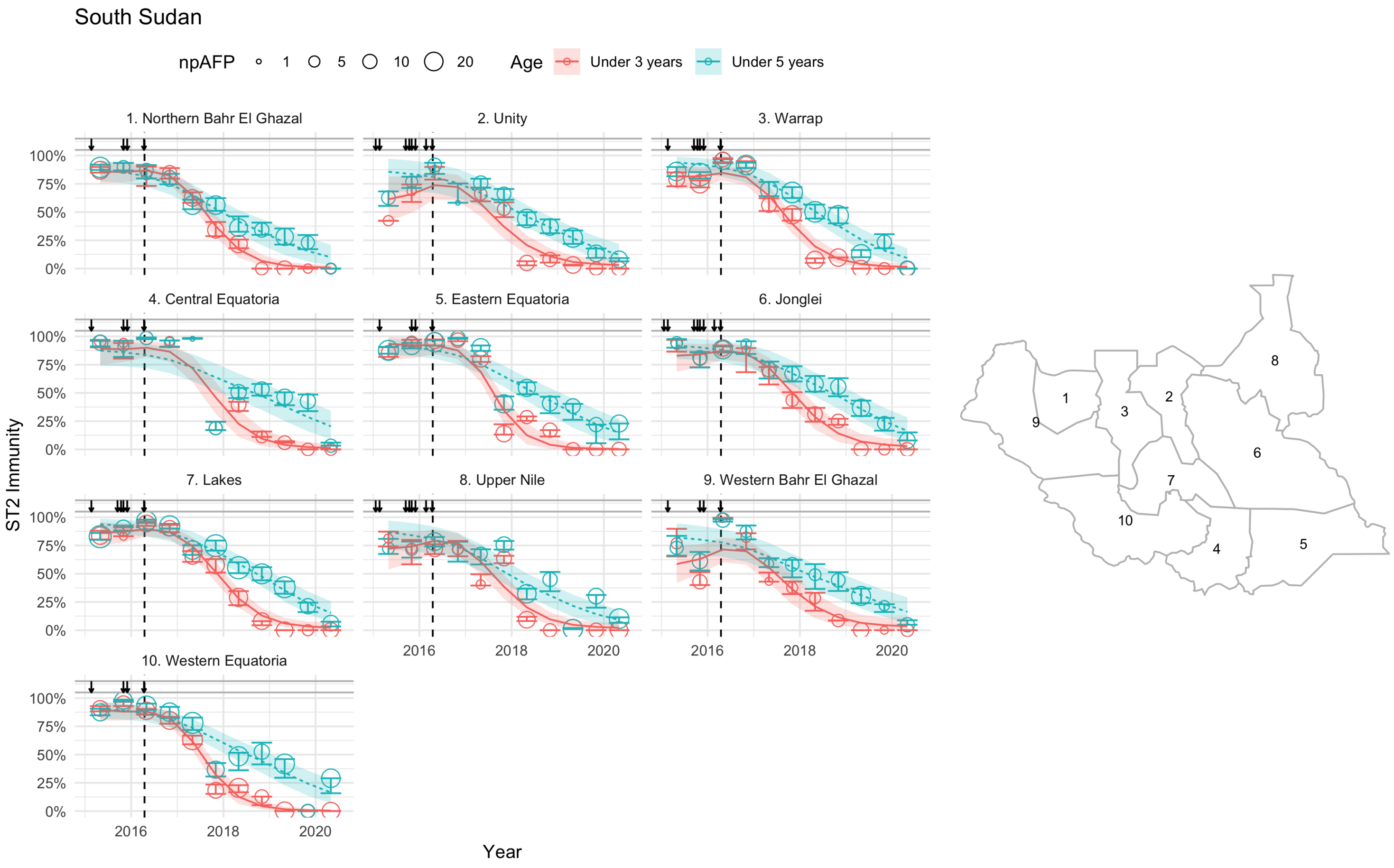

Figure S97: Type 2 population immunity from OPV in each province of South Sudan in children under five (blue) and under three (red). Circles show median of bootstrapped crude immunity estimates, error bars show 2.5th and 97.5th percentiles of bootstrapped estimates. Size of circles indicate the number of non-polio AFP cases that each crude estimate is based on. Lines show median smoothed immunity estimate, transparent ribbons show 95% credible interval. Arrows show timing of tOPV (before withdrawal, dotted line) or mOPV2 SIAs (after withdrawal, dotted line). Height of arrows should the proportion of under-five population targeted in SIA. The publication of this map does not imply the expression of any opinion whatsoever on the part of WHO concerning the legal status of any territory, city or area or of its authorities, or concerning the delimitation of its frontiers or boundaries.

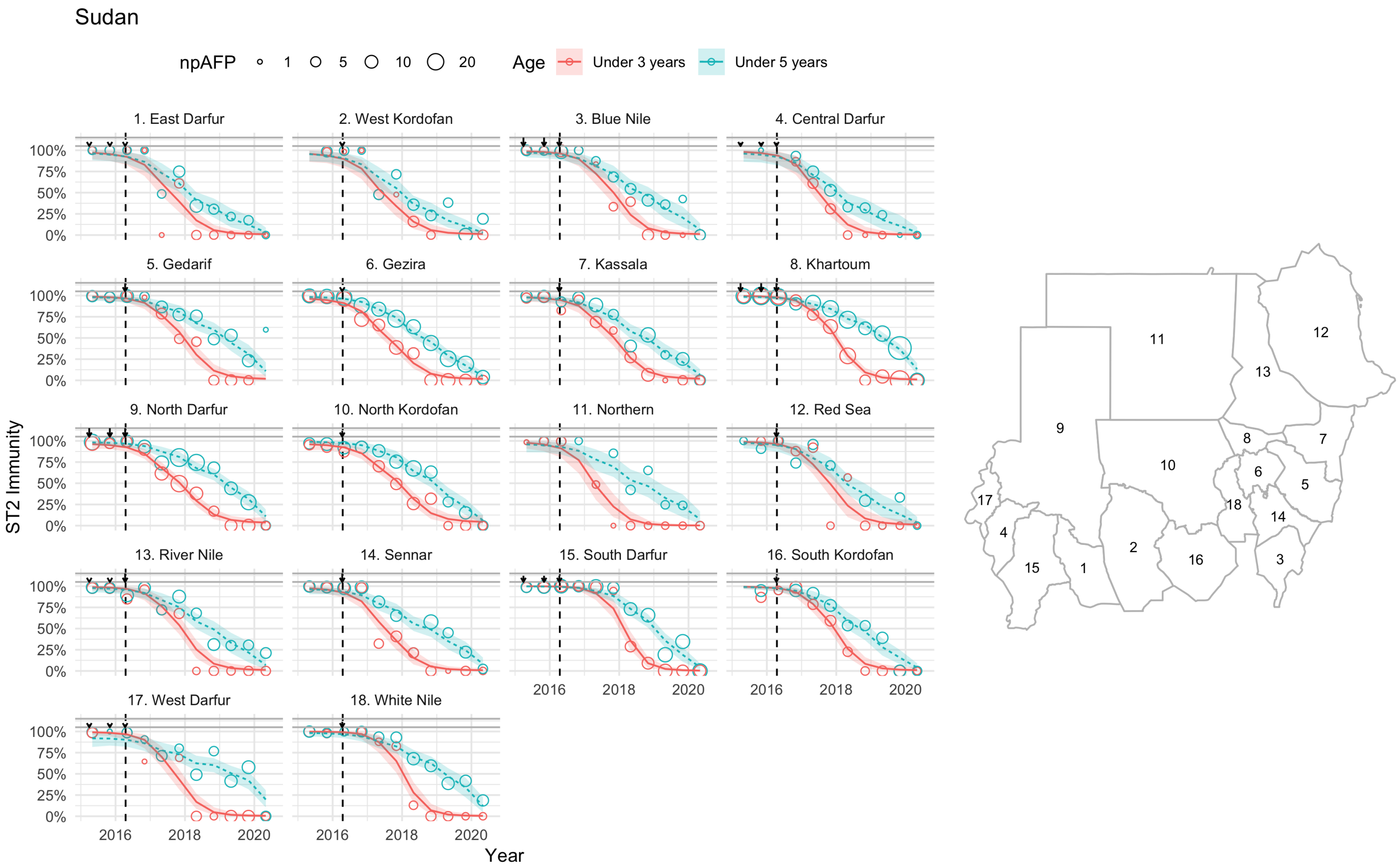

Figure S98: Type 2 population immunity from OPV in each province of Sudan in children under five (blue) and under three (red). Circles show median of bootstrapped crude immunity estimates, error bars show 2.5th and 97.5th percentiles of bootstrapped estimates. Size of circles indicate the number of non-polio AFP cases that each crude estimate is based on. Lines show median smoothed immunity estimate, transparent ribbons show 95% credible interval. Arrows show timing of tOPV (before withdrawal, dotted line) or mOPV2 SIAs (after withdrawal, dotted line). Height of arrows should the proportion of under-five population targeted in SIA. The publication of this map does not imply the expression of any opinion whatsoever on the part of WHO concerning the legal status of any territory, city or area or of its authorities, or concerning the delimitation of its frontiers or boundaries.

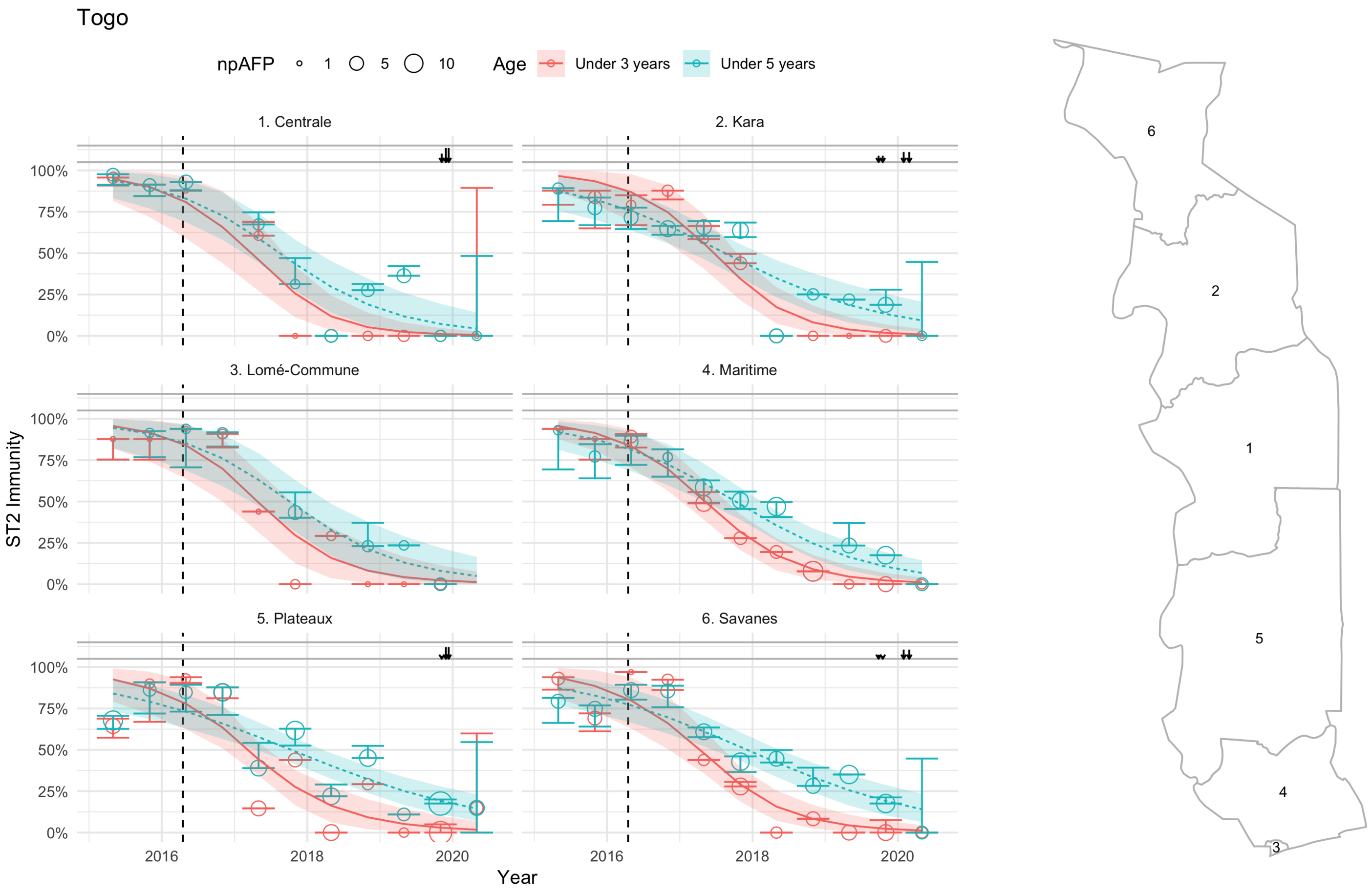

Figure S99: Type 2 population immunity from OPV in each province of Togo in children under five (blue) and under three (red). Circles show median of bootstrapped crude immunity estimates, error bars show 2.5th and 97.5th percentiles of bootstrapped estimates. Size of circles indicate the number of non-polio AFP cases that each crude estimate is based on. Lines show median smoothed immunity estimate, transparent ribbons show 95% credible interval. Arrows show timing of tOPV (before withdrawal, dotted line) or mOPV2 SIAs (after withdrawal, dotted line). Height of arrows should the proportion of under-five population targeted in SIA. The publication of this map does not imply the expression of any opinion whatsoever on the part of WHO concerning the legal status of any territory, city or area or of its authorities, or concerning the delimitation of its frontiers or boundaries.

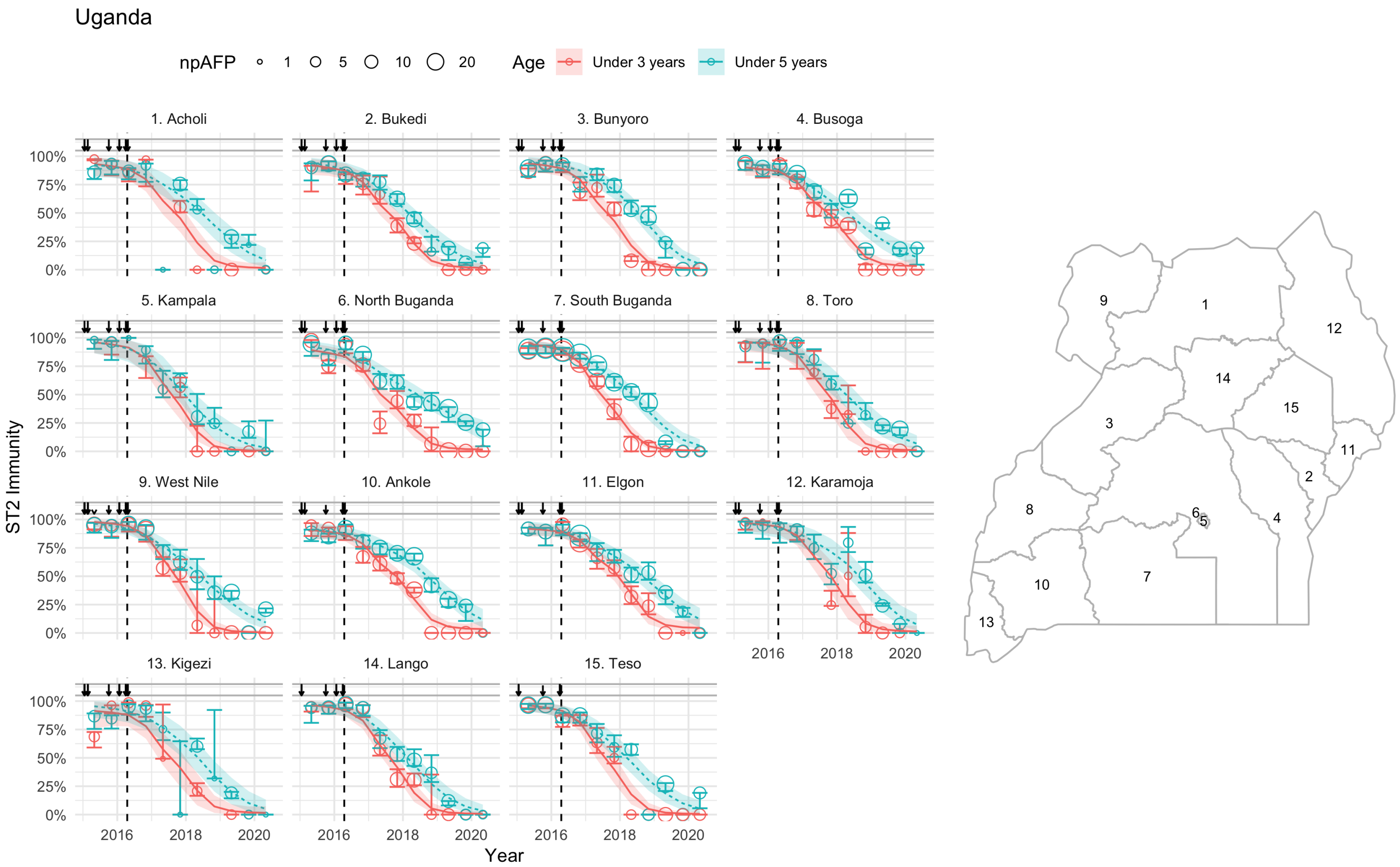

Figure S100: Type 2 population immunity from OPV in each province of Uganda in children under five (blue) and under three (red). Circles show median of bootstrapped crude immunity estimates, error bars show 2.5th and 97.5th percentiles of bootstrapped estimates. Size of circles indicate the number of non-polio AFP cases that each crude estimate is based on. Lines show median smoothed immunity estimate, transparent ribbons show 95% credible interval. Arrows show timing of tOPV (before withdrawal, dotted line) or mOPV2 SIAs (after withdrawal, dotted line). Height of arrows should the proportion of under-five population targeted in SIA. The publication of this map does not imply the expression of any opinion whatsoever on the part of WHO concerning the legal status of any territory, city or area or of its authorities, or concerning the delimitation of its frontiers or boundaries.

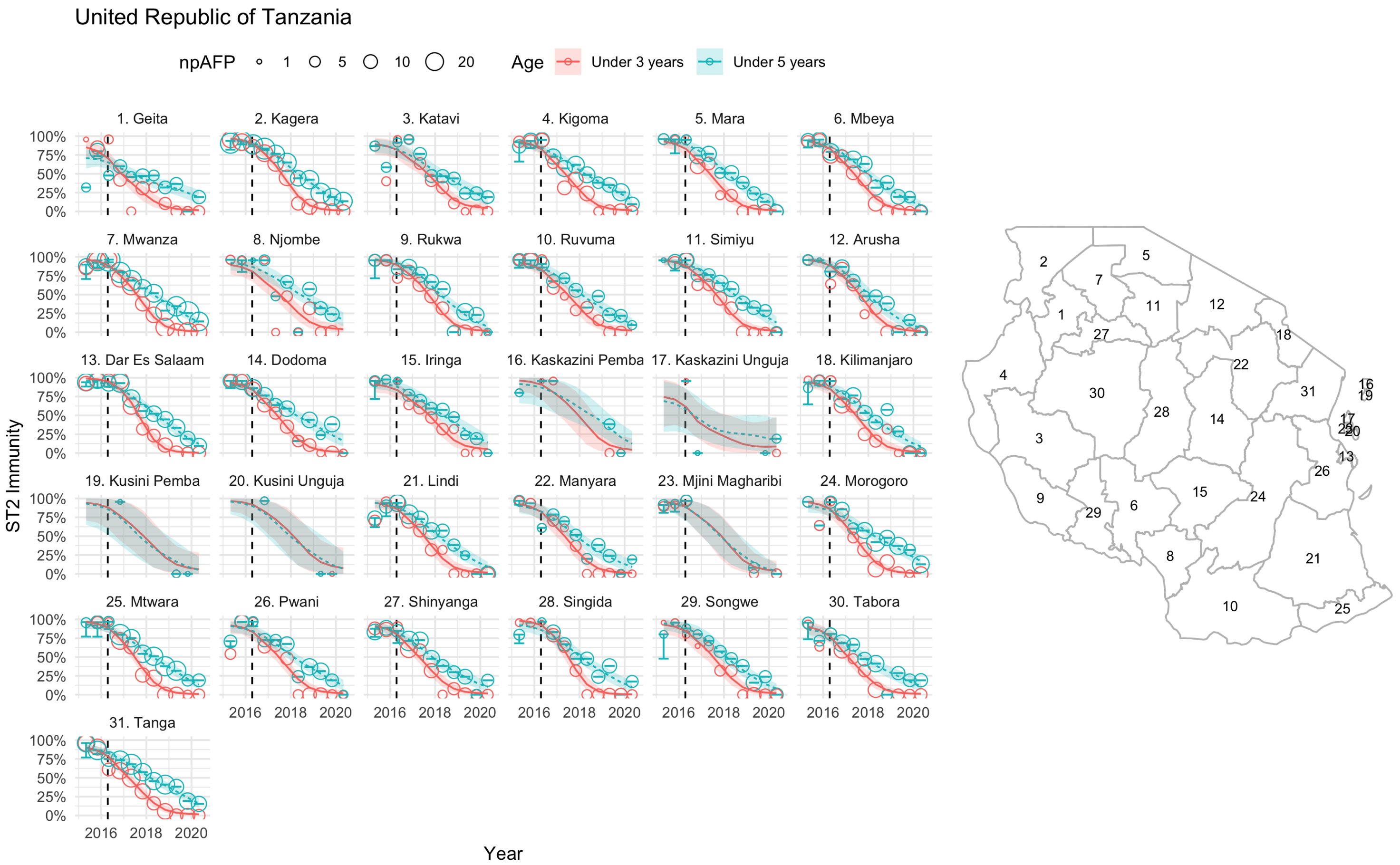

Figure S101: Type 2 population immunity from OPV in each province of Tanzania in children under five (blue) and under three (red). Circles show median of bootstrapped crude immunity estimates, error bars show 2.5th and 97.5th percentiles of bootstrapped estimates. Size of circles indicate the number of non-polio AFP cases that each crude estimate is based on. Lines show median smoothed immunity estimate, transparent ribbons show 95% credible interval. Arrows show timing of tOPV (before withdrawal, dotted line) or mOPV2 SIAs (after withdrawal, dotted line). Height of arrows should the proportion of under-five population targeted in SIA. The publication of this map does not imply the expression of any opinion whatsoever on the part of WHO concerning the legal status of any territory, city or area or of its authorities, or concerning the delimitation of its frontiers or boundaries.

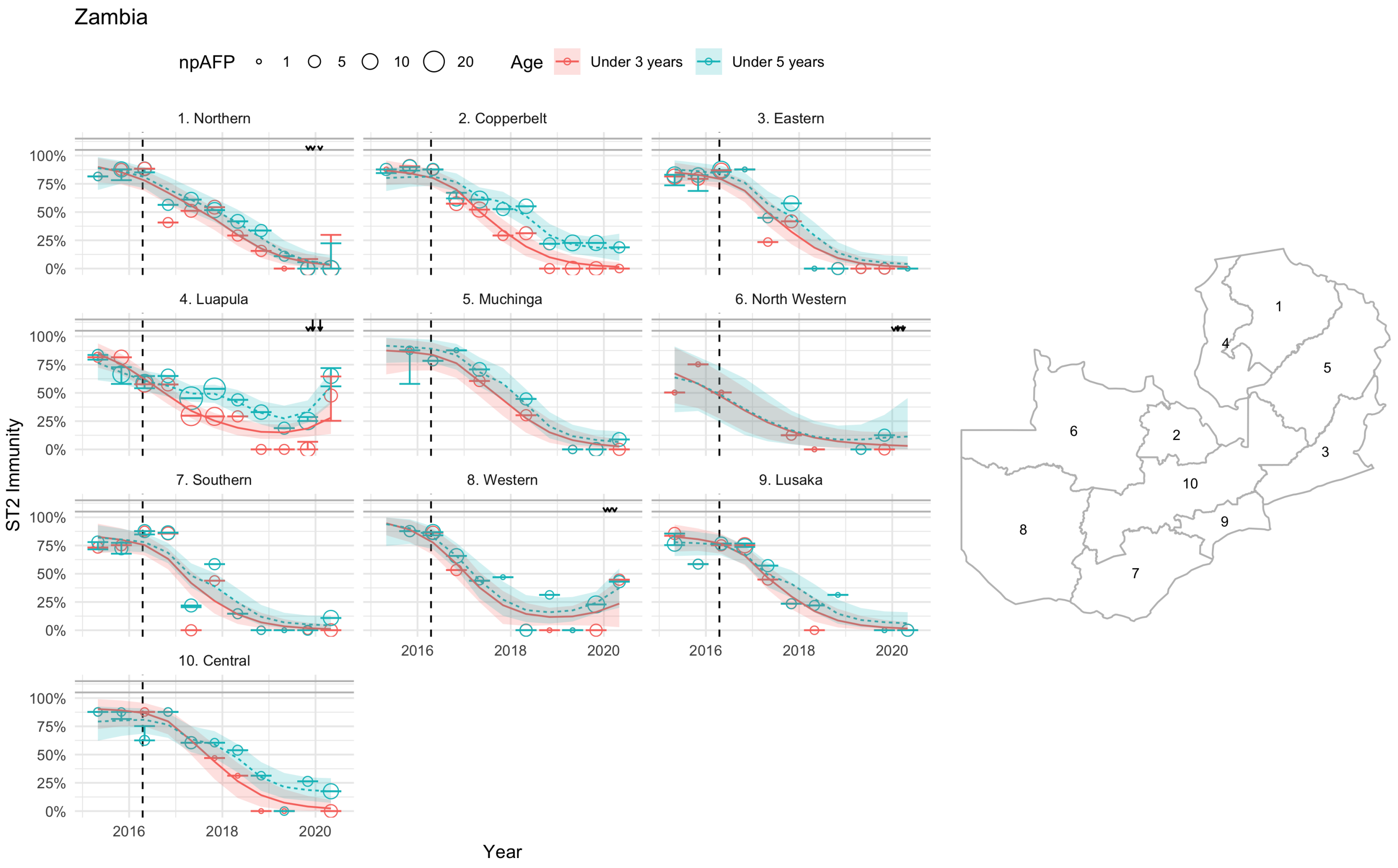

Figure S102: Type 2 population immunity from OPV in each province of Zambia in children under five (blue) and under three (red). Circles show median of bootstrapped crude immunity estimates, error bars show 2.5th and 97.5th percentiles of bootstrapped estimates. Size of circles indicate the number of non-polio AFP cases that each crude estimate is based on. Lines show median smoothed immunity estimate, transparent ribbons show 95% credible interval. Arrows show timing of tOPV (before withdrawal, dotted line) or mOPV2 SIAs (after withdrawal, dotted line). Height of arrows should the proportion of under-five population targeted in SIA. The publication of this map does not imply the expression of any opinion whatsoever on the part of WHO concerning the legal status of any territory, city or area or of its authorities, or concerning the delimitation of its frontiers or boundaries.

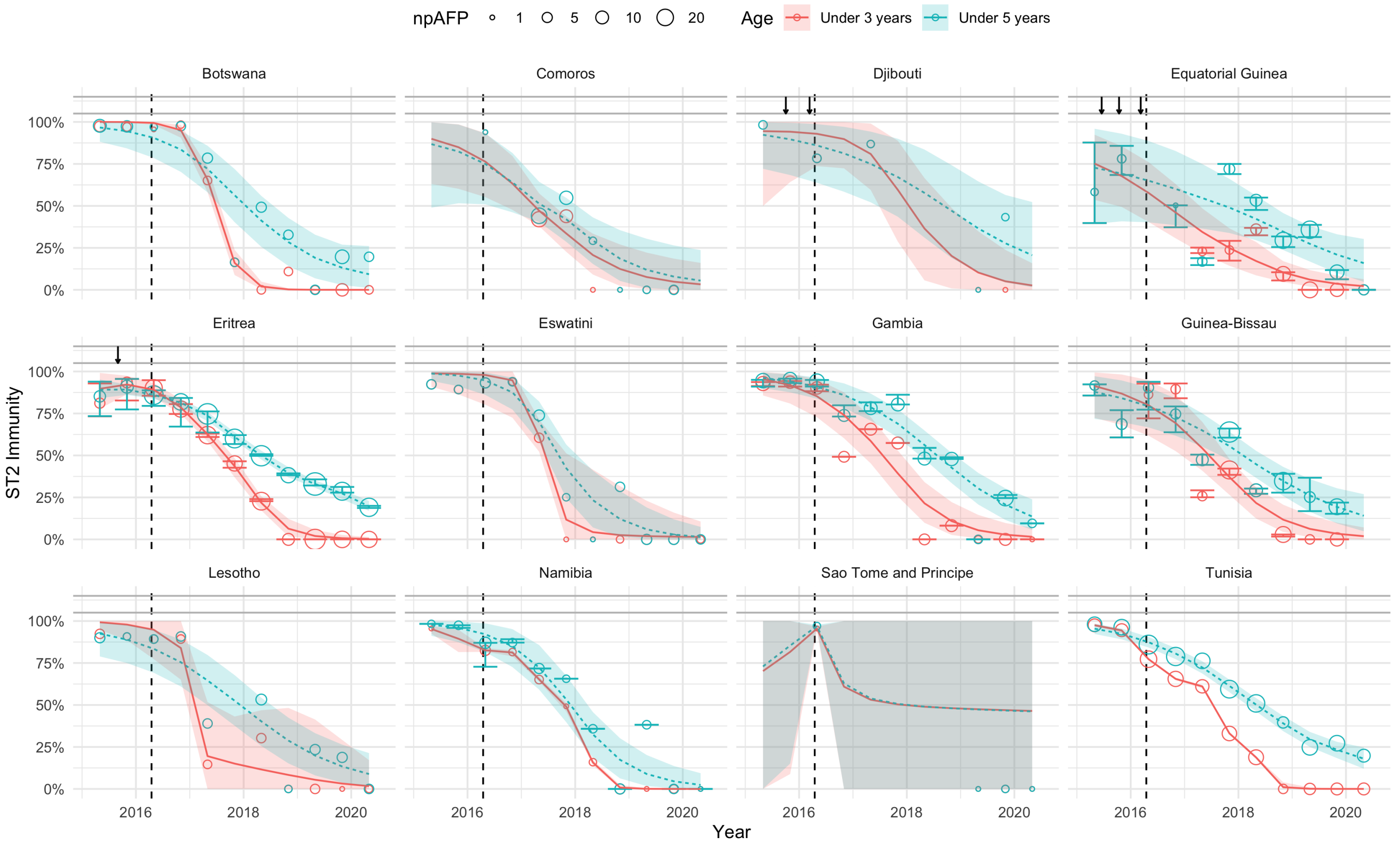

Figure S103: Type 2 population immunity from OPV in Botswana, Comoros, Djibouti, Equatorial Guinea, Eritrea, Eswatini, the Gambia, Guinea-Bissau, Lesotho, Namibia, Sao Tome and Principe, and Tunisia in children under five (blue) and under three (red). Circles show median of bootstrapped crude immunity estimates, error bars show 2.5th and 97.5th percentiles of bootstrapped estimates. Size of circles indicate the number of non-polio AFP cases that each crude estimate is based on. Lines show median smoothed immunity estimate, transparent ribbons show 95% credible interval. Arrows show timing of tOPV (before withdrawal, dotted line) or mOPV2 SIAs (after withdrawal, dotted line). Height of arrows should the proportion of under-five population targeted in SIA. The publication of this map does not imply the expression of any opinion whatsoever on the part of WHO concerning the legal status of any territory, city or area or of its authorities, or concerning the delimitation of its frontiers or boundaries.
